# Rhodium-catalysed C(*sp*^2^)–C(*sp*^2^) bond formation via C–H/C–F activation

**DOI:** 10.1038/ncomms8472

**Published:** 2015-06-17

**Authors:** Panpan Tian, Chao Feng, Teck-Peng Loh

**Affiliations:** 1 Hefei National Laboratory for Physical Sciences at the Microscale and Department of Chemistry, University of Science and Technology of China, Hefei 230026, China; 2 Division of Chemistry and Biological Chemistry, Nanyang Technological University, 50 Nanyang Avenue, Singapore 639798, Singapore

## Abstract

Fluoroalkenes represent a class of privileged structural motifs, which found widespread use in medicinal chemistry. However, the synthetic access to fluoroalkenes was much underdeveloped with previous reported methods suffering from either low step economy or harsh reaction conditions. Here we present a Rh^III^-catalysed tandem C–H/C–F activation for the synthesis of (hetero)arylated monofluoroalkenes. The use of readily available *gem*-difluoroalkenes as electrophiles provides a highly efficient and operationally simple method for the introduction of α-fluoroalkenyl motifs onto (hetero)arenes under oxidant-free conditions. Furthermore, the employment of alcoholic solvent and the *in-situ* generated hydrogen fluoride are found to be beneficial in this transformation, indicating the possibility of the involvement of hydrogen bond activation mode with regards to the C–F bond cleavage step.

## Abstract (web summary)

Fluoroalkenes are found widely in biologically active compounds, but their introduction can be difficult or laborious. Here, the authors report a C–H/C–F activation strategy to introduce monofluoroalkenes into organic molecules in one step with good to excellent yields.

## Graphical abstract

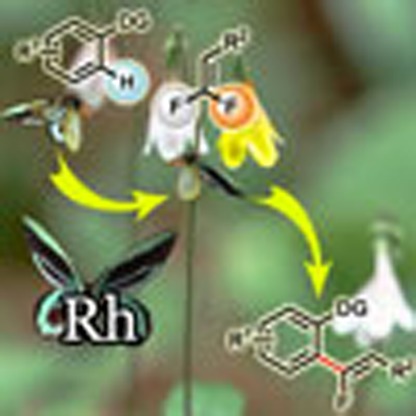

Fluorine, ‘small atom with a big ego', due to its intrinsic properties such as small size and high electronegativity in comparison with other halogen atoms, has played a key role in all fields of science^1^,^2^. More specifically, the incorporation of fluorine or fluorine-containing structural motifs into organic molecule brings about substantial improvement in its bioactivity and provides unique chemical and physical properties, thus enabling the widespread use of this strategy in the field of medicinal chemistry^3^,^4^,^5^. In this context, fluoroalkenes represent a class of very important molecules owing to their biological properties and also their synthetic potential in synthetic organic chemistry^6^,^7^. Furthermore, as favourable peptide bond mimetics, both electrostatically and geometrically, as well as their resistant nature to enzymatic degradation, the fluoroalkene structural motifs have been attracting increasing interest in medicinal chemistry and drug-discovery research^8^,^9^,^10^,^11^,^12^. Albeit their great importance, compared with the development of analogous fluorination and trifluoromethylation methodologies^13^,^14^,^15^,^16^,^17^,^18^, the synthetic access to alkenyl fluorides remains largely underdeveloped, with most of the reported protocols suffering from the need of substrate pre-activation or using non-readily available starting materials, low regio- or stereo-selectivity and poor functional group tolerance due to the employment of sensitive reagents^19^,^20^,^21^. By taking advantage of the Pd/Cu-catalysed C–H activation strategy, Hoarau and colleagues^22^,^23^ reported elegant works on the fluoroalkenylation of heteroarenes either through C–H/C–Br or C–H/CO_2_H couplings (Fig. 1d). Notwithstanding the advance attained, the development of a new synthetic method, which streamlines the access to fluoroalkene motifs using readily available building blocks while avoiding substrate pre-activation steps, would still be of meaningful importance in both the synthetic organic chemistry and pharmaceuticals development.

With explosive advancements achieved in the past decade, the directing group assisted C–H bond activation has emerged as a powerful and competent tool, which not only result in fundamental changes in the retrosynthesis but more importantly represents the state-of-the-art in the organic synthesis and shows the direction of development beyond what traditional synthetic methodologies could bring about^24^,^25^. As a subclass, the Rh(III)-catalysed C–H functionalization is booming rapidly in the recent years^26^,^27^,^28^,^29^,^30^. Although a diverse range of synthetically useful transformations have already been attained in this context, the application of Rh(III)-catalysed C–H activation in the fluorine chemistry is unprecedented and more importantly the employment of C(*sp*^2^)-X as electrophilic coupling partner is totally unrecognized in high valent rhodium catalysis^31^,^32^. Therefore, the development of a Rh(III)-catalysed C–H functionalization protocol that enables the easy access of biologically relevant fluoroalkenes is highly desirable and the feasibility of such protocol was based on the following considerations: (i) Rh(III)-catalysed alkenylation has evolved to be a competent and reliable method for the introduction of olefin segments, although stoichiometric amount of external oxidant was always required to fulfill the redox demand (Fig. 1a)^33^,^34^; (ii) notwithstanding its high dissociation energy, the C–F bond could be activated by transition metal or through the formation of hydrogen bond, provided that suitable hydrogen bond donor is present (Fig. 1b)^35^,^36^,^37^,^38^,^39^,^40^; (iii) *gem*-difluoroalkenes represent a class of appealing synthetic intermediates with the C–C double bond being highly polarized because of the electronegativity of fluorine and also the repulsion effect stemming from its unpaired electrons^41^,^42^. Furthermore, it is well-known that hetero-nucleophiles could undergo facile nucleophilic addition or substitution reactions under basic conditions (Fig. 1c)^43^,^44^,^45^,^46^. We reasoned that the putative carbocation character of *gem*-difluoroalkenes is of critical importance in ensuring the regioselectivity of carborhodationic step and the employment of hydrogen-bond donor would result in the activation of C–F bond. Assuming the viability of our proposal, there comes with an affiliated bonus, as no external oxidant was required because of the redox neutral property of this transformation^32^. With our ongoing interest in rhodium and fluorine chemistry^47^,^48^,^49^, herein we would like to present the chelation-assisted C–H activation strategy for the direct incorporation of the α-fluoroalkenyl unit using *gem*-difluoroalkene as the fluoroalkene donor (Fig. 1e)^50^.

## Results

### Reaction condition optimization for **3aa**

To test our hypothesis, the cross-coupling between 1-(2, 2-difluorovinyl)-4-methoxybenzene (**2a**) and 1-(pyrimidin-2-yl)-1*H*-indole (**1a**) was selected as the model reaction. After examination of a considerable variety of reaction parameters, we were rather pleased to find that the anticipated fluoroalkenylation product could be obtained in 89% yield when using [RhCp*(CH_3_CN)_3_](SbF_6_)_2_ as the catalyst and methanol as solvent, and at 80 °C for 16 h (see Supplementary Tables 1–3 for details of reaction optimization). In accordance with our hypothesis mentioned above, this reaction occurred in an excellent regio- and stereo-selective manner due to the intrinsic property of *gem*-difluoro substituents. Considering the fact that hydrogen fluoride (HF) itself is a strong hydrogen-bond donor, which could provide a resultant stabilization energy in the cleavage of C(*sp*^3^)–F bond process, as well as to evaluate whether such an interaction was present and in turn facilitated our reaction process, control experiments with external HF sequesters were conducted^37^. As expected, the addition of exogenous bases such as NaHCO_3_, Na_2_CO_3_ and 2,6-*di*-*tert*-butyl-4-methylpyridine for the neutralization of the HF generated in the reaction was proved to be detrimental to this transformation. Taking this phenomenon together with the superiority of alcoholic solvents into consideration, it is reasonable to assume that the activation of C–F bond through hydrogen-bond formation is involved and proved to be crucial for the execution of this protocol. Furthermore, the hydroarylation product arising from the protonation of carborhodation intermediate was not observed throughout the whole reaction, implying the kinetic favourability of the *β*-defluorination step^51^.

### Substrate scope

Having obtained the optimized reaction conditions, the issues with respect to functional group tolerance and scope of *gem*-difluoroalkene was thus addressed and the results were summarized in Table 1. In general, with respect to aryl substituted *gem*-difluoroalkenes, both electron-donating and electron-withdrawing functional groups, regardless of the substitution patterns, on the arene moiety were all well tolerated, affording the desired products in high to excellent yield. When electron-rich substituents such as Me or OMe are present in the *ortho*, *meta* or *para* positions, the reaction proceeded well, leading to the formation of the desired products with the yields ranging from 69% to 91% (**3ab**–**3ae**). However, when **2f** was used as a substrate, the reaction afforded product **3af** in 46% yield, which may be attributed to the competitive chelation effect of nitrogen substituent in this case. Substrates with synthetically useful functional groups such as NO_2_, CF_3_, Ac, CO_2_Me and CN (**2g**–**2k**) participated in this reaction efficiently, to deliver the fluoroalkenylation products in high to excellent yields. Furthermore, we were pleased to find that halogen substituents were also well tolerated in this reaction, which offers the opportunity for further synthetic elaborations (**2l**–**2p**). The benzo[*d*][1,3]dioxole and naphthalyl derived substrates **2q**, **2r** were also amenable to this reaction conditions to deliver the desired products in 91% and 94% yield, respectively. Notably, heterocycle based *gem*-difluoroalkenes, such as thiophene (**2s**) and benzo[*b*]thiophene (**2t**), also proved to be viable substrates, leading to valuable products in high yields. Pleasingly, this protocol also accommodates the alkyl derived *gem*-difluoroakenes, and when **2u** was employed as substrate the desired product **2au** was obtained in 81% yield.

The reaction generality of indole derivatives was subsequently investigated and the results were summarized in Table 2. It was found that electron-releasing groups, regardless of their positions on the substrates, were proved to be beneficial, leading to the desired products in excellent yields (**3ba**–**3fa**). Again, halogen atoms were nicely tolerated (**3ga**–**3ka**). Substituents with strong electron-withdrawing ability have somewhat deleterious effect on this reaction and the impact became more severe with regard to 5-CN-based indole substrate **1m**, which afforded the coupling product **3ma** in low yield. Furthermore, it was realized that this protocol was not restricted to indole-derived heterocycles as demonstrated in the cases of **1n** to **1p**. It needs to be noted that the switch to trifluoroethanol as solvent was proved to be critical for achieving high reactivity when pyrrole substrates **1n** and **1o** were employed^32^. The superiority of CF_3_CH_2_OH as the reaction solvent in these cases was attributed to its ability of acting as a better hydrogen-bond donor than MeOH. When 2-(thiophen-2-yl)pyrimidine **1p** was used, the reaction also worked well to afford the desired product (**3pa**) in 72% yield. To further challenge the reaction scope of this reaction, 2-arylpyridine derivatives were subsequently examined. We were pleased to find that when simple 2-phenyl pyridine was tested, the desired product **5aa** was obtained in synthetically useful yield. For substrates containing *ortho*-substituents, such as **4b**–**4c**, the reaction efficiency was slightly decreased because of the steric hindrance involved, whereas in the cases of **4d** and **4e**, which have *meta*-substituents, the reaction proceeded selectively on the sterically more accessible site to afford the desired products in good yields. In accordance with the solvent effect encountered in the cases of **3na** and **3oa** formations, the use of trifluoroethanol as solvent was shown to be beneficial with respect to the reaction efficiency when using substrates containing electron-withdrawing groups, although at the expense of high selectivity for *mono*-fluoroalkenylations in some cases (**5ga**–**5ja**). Furthermore, substrates that possess substituents on the pyridine ring also engaged well in this reaction to furnish the desired products in moderate to good yields (**5ka** and **5na**). Finally, 2-phenylpyrimidine and benzo[*h*]quinoline were all viable substrates and participated in this reaction nicely to produce the desired products in high yields (**5la**–**5ma**).

To further extend the reaction scope and to gain more insight into the limitation of this reaction, substrates that do not contain strong chelation-directing groups, such as pyridine and pyrimidine, were examined and the results were presented in Table 3. Although at the present stage ketone, imine and anilide proved to be not suitable substrates, benzamide derivatives were found to be effective coupling partners, especially the Ts-imide analogues, which provide the desired fluoroalkenylation products in moderate yields, with slight modification of the reaction condition. Specifically, Ts-imide substrates with either electron-donating or electron-withdrawing groups reacted smoothly under modified reaction condition to deliver the desired products (**7aa**–**7ga**). The *ortho*-substitute was nicely tolerated without any deleterious effect on the reaction efficacy being observed compared with the *para*-analogue (**7aa** versus **7ba**). When substrates that contain other potential chelation groups, such as ester and carbamate were employed, the reaction selectively occurred on the *ortho*-position of Ts-imide directing group (**7fa** and **7ga**). It should be pointed out that thiophene-derived substrate also worked efficiently to produce the product **7ha** in synthetically useful yield. In contrast to the effectiveness of using Ts-imide as directing group, when the reaction was carried out with *N,N*-diisopropylbenzamides as substrates, the reaction tend to afford the desired products in relatively low yields, which was due to the low conversion of starting materials (**7ia**–**7la**).

### Synthetic elaboration and mechanistic investigation

**Figure 2:**
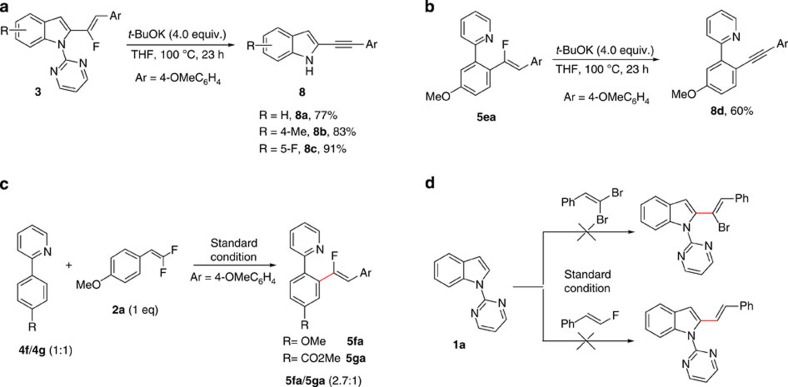
Control experiments and synthetic application. (**a**) Base-promoted dehydrofluoration for the synthesis of 2-alkynyl indole derivatives. (**b**) Base-promoted dehydrofluoration for the synthesis of pyridine derivative. (**c**) Competitive reaction between **4f** and **4g**. (**d**) Control reactions between **1a** and *gem*-dibromoalkene and *mono*-fluoroalkene.

To further showcase the synthetic applicability of these fluoroalkenylation products, base-promoted dehydrofluoration protocol for the synthesis of alkynes were attempted. After the examination of a variety of reaction conditions, we were delighted to find that the alkyne product **8a** could be isolated in 77% yield when **3aa** was treated with 4 equivalent of *t*-BuOK in a solution of tetrahydrofuran (THF) at 100 °C for 23 h. With this strategy, 2-alkynyl indoles **8b** and **8c** were readily obtained in excellent yield (Fig. 2a). Furthermore, the *t*-BuOK-promoted dehydrofluoration was also proved to be effective for the synthesis of 2-(2-(alkynyl)phenyl)pyridine derivatives and when **5ea** was employed, the desired product **8d** was generated in 60% yield (Fig. 2b). To gain insight into the electronic bias of the C–H activation step, competition experiments between **4f**/**4g** was examined. When an equimolar mixture of **4f**, **4g** and **2a** were subjected to the optimized reaction condition, the desired products **5fa** and **5ga** were formed in the ratio of 2.7:1, thus indicating that electron-rich substrate was more favoured in the C–H activation step in this reaction (Fig. 2c). To determine the role of *gem*-difluoro substituents in this reaction, substrates with *gem*-dibromo and *mono*-fluoro substituents were subjected to the optimized reaction condition; however, neither led to the formation of the desired products. These experiments clearly demonstrated that the *gem*-difluoro substituents was indispensable for the activation of the C–C double bond, which is in full agreement with our hypothesis (Fig. 2d).

## Discussion

A highly effective rhodium(III)-catalysed α-fluoroalkenylation of (hetero)arenes through C–H/C–F bond activations was developed. This reaction proceeds smoothly and stereoselectively under base- and oxidant-free reaction conditions to deliver a diverse range of synthetically useful and pharmaceutically relevant *cis*-alkenyl fluorides in good yields. Furthermore, the involvement of activation of the C–F bond through hydrogen-bond formation is crucial for the success of this transformation. Last but not the least, the success of incorporation of α-fluoroalkenyl unit onto (hetero)arenes through C–H/C–F activation manifold was in principle amenable to be extended to other processes wherein the key intermediates arylrhodiums were not formed by C–H activation, provided that each elemental reaction step was compatible and balanced, thereby offering a more generalized and versatile protocol for the synthesis of functionalized fluoroalkene derivatives.

## Methods

### Materials

For 1H, 13C NMR spectra of compounds in this manuscript, see Supplementary Figs 1–66. For optimization of reaction conditions, see Supplementary Tables 1–3. For details of the synthetic procedures, see Supplementary Methods.

### Syntheses of products **3** and **5**

An oven-dried 10-ml Schlenk tube with a magnetic stirring bar was charged with **1** or **4** (0.1 mmol), [RhCp*(CH_3_CN)_3_](SbF_6_)_2_ (3.3 mg, 0.004 mmol), **2** (0.15 mmol) in sequence, followed by adding anhydrous MeOH (0.5 ml) through syringe. The reaction tube was sealed with Teflon-coated screw cap and the reaction solution was stirred at 80 °C for 16 h. After cooling the reaction mixture to room temperature and removing the solvent *in vacuo*, the resulting residue was purified by silica gel column chromatography to afford the desired product **3** or **5**.

## Additional information

**How to cite this article:** Tian, P. *et al.* Rhodium-catalysed C(*sp*^2^)–C(*sp*^2^) bond formation via C–H/C–F activation. *Nat. Commun.* 6:7472 doi: 10.1038/ncomms8472 (2015).

## Supplementary Material

Supplementary Information Supplementary Figures 1-66, Supplementary Tables 1-3, Supplementary Methods and Supplementary References

## Figures and Tables

**Figure 1:**
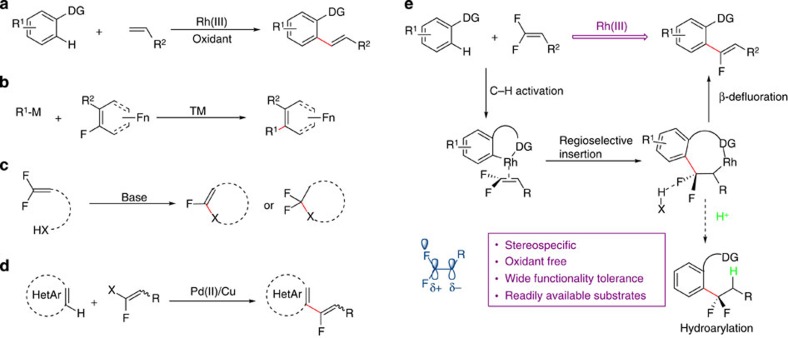
Proposed method of rhodium-catalysed α-fluoroalkenylation. (**a**) Oxidative alkenylation through Rh(III)-catalysed C–H activation. (**b**) Transition-metal-catalysed C–C bond formation through C–F activation. M, metal; TM, transition metal. (**c**) Base-promoted inter- or intramolecular nucleophilic addition or substitution of *gem*-difluoroalkenes with heteronucleophiles. X, hetero atom. (**d**) Pd/Cu-catalysed C–H fluoroalkenylation of heteroarenes. X, Br or CO_2_H. (**e**) In this report, oxidant-free Rh(III)-catalysed α-fluoroalkenylation of (hetero)arenes. The hydrogen bonding interaction is believed to promote the cleavage of C–F bond, which, in turn, renders this reaction redox neutral. DG, directing group.

**Table 1:**
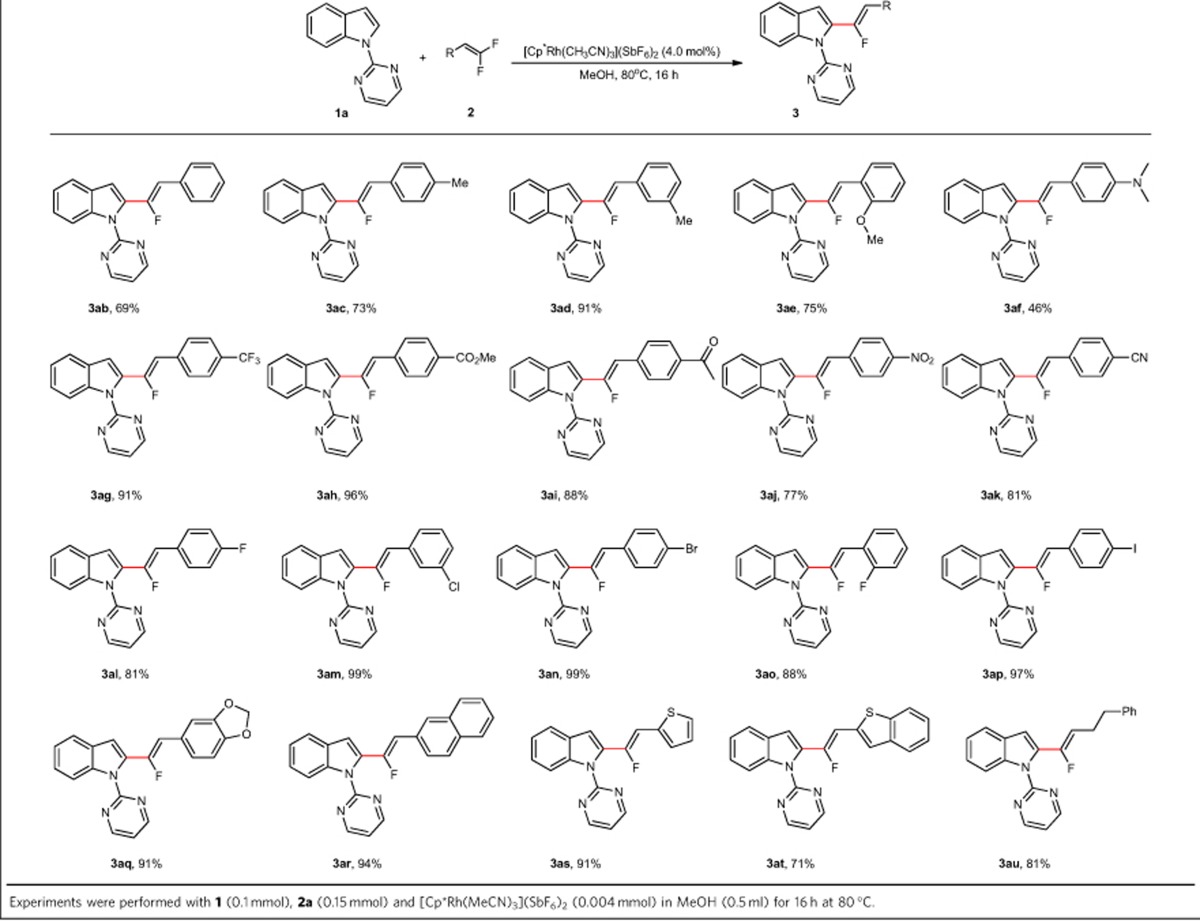
Scope of gem-difluoroalkene for the C–H/C–F activation reaction.

**Table 2:**
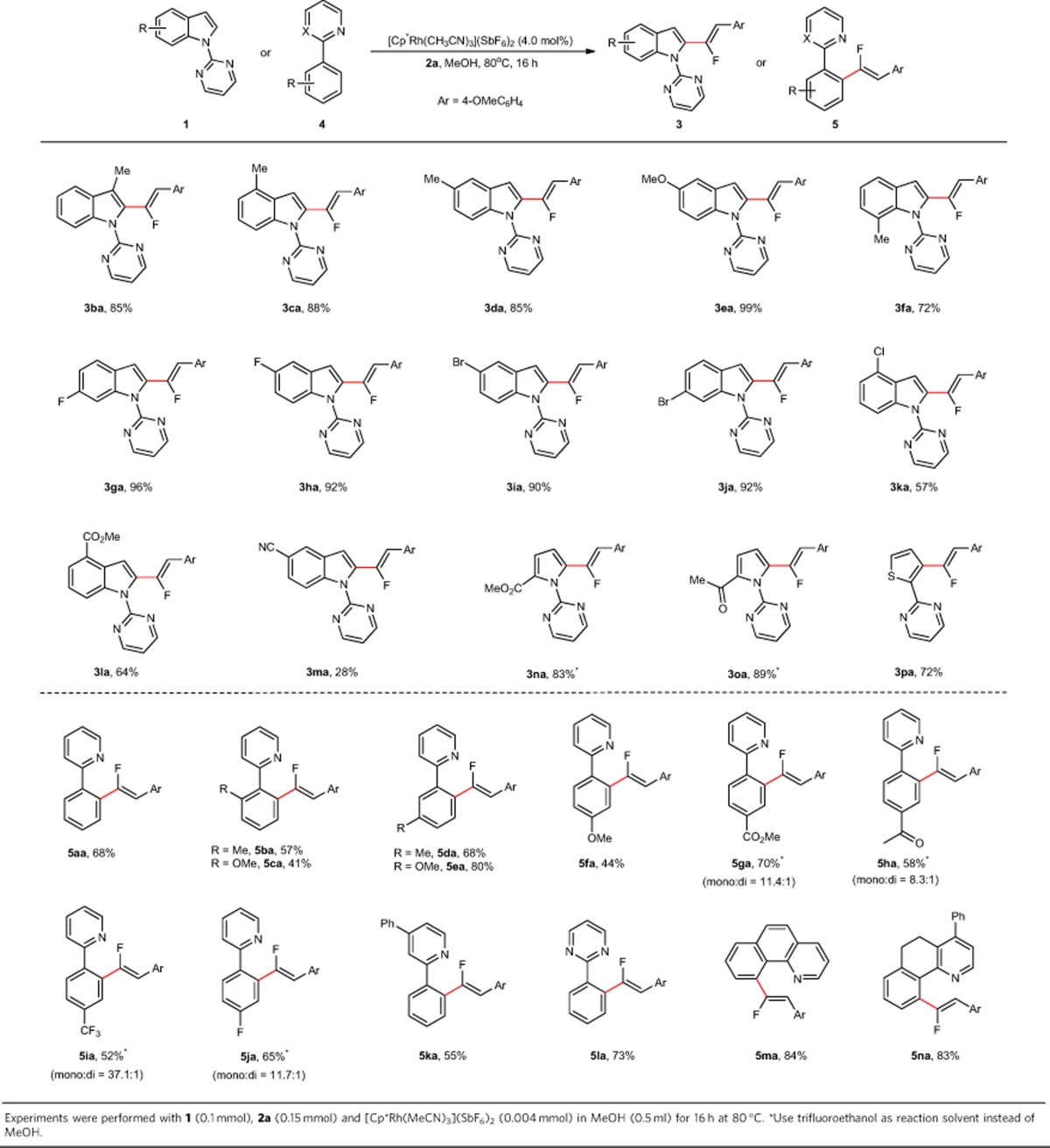
Scope of indole and 2-arylpyridine derivatives in the C–H/C–F activation reaction.

**Table 3:**
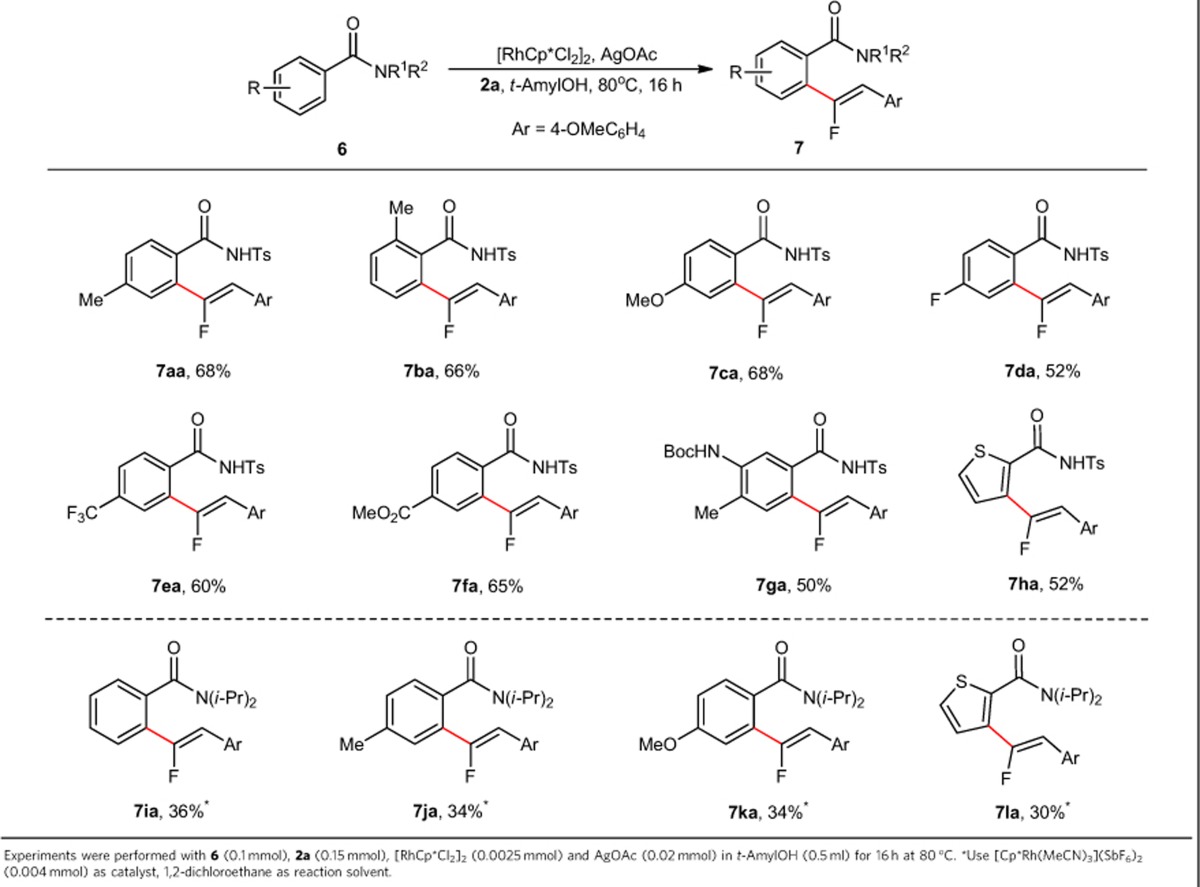
Scope of benzamide in the C–H/C–F activation reaction.
