# Supplementary material for: Rhodium-catalysed C(sp2)–C(sp2) bond formation via C–H/C–F activation
Source: Nat Commun. 2015 Jun 17;6:7472. doi: 10.1038/ncomms8472 (PMC4557390; doi:10.1038/ncomms8472)
Supplement: Supplementary Information — Supplementary Figures 1-66, Supplementary Tables 1-3, Supplementary Methods and Supplementary References [file ncomms8472-s1.pdf]

Supplementary Figures

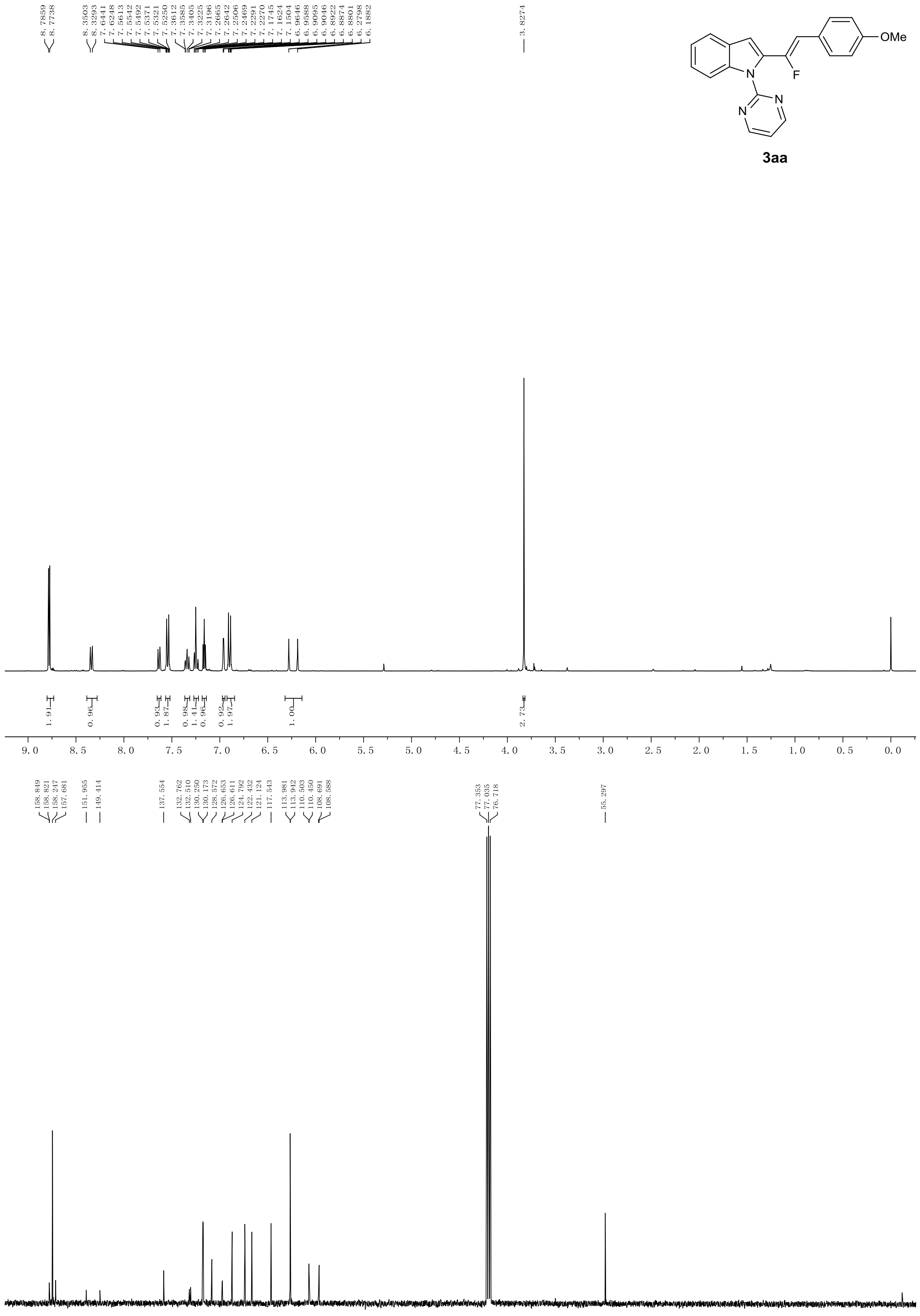

Supplementary Figure 1. ^{1}H and ^{13}C NMR spectra for product 3aa

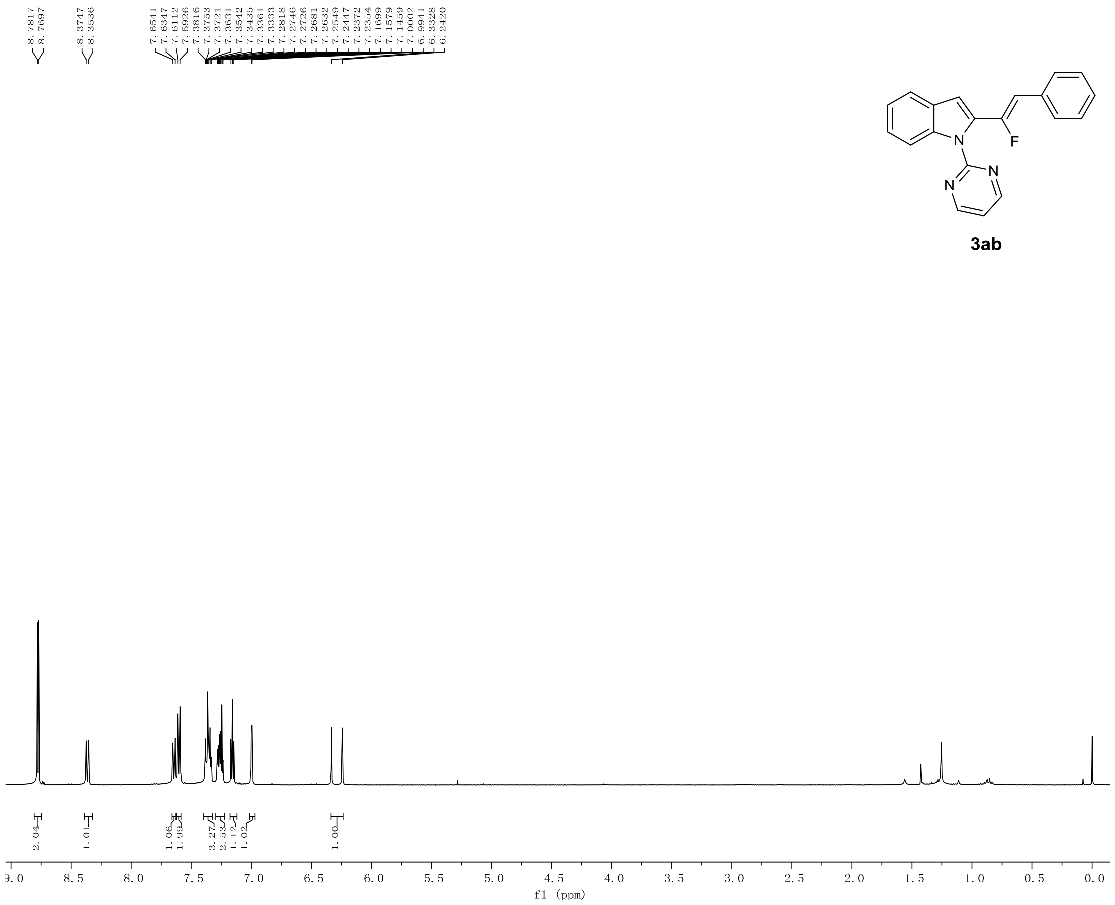

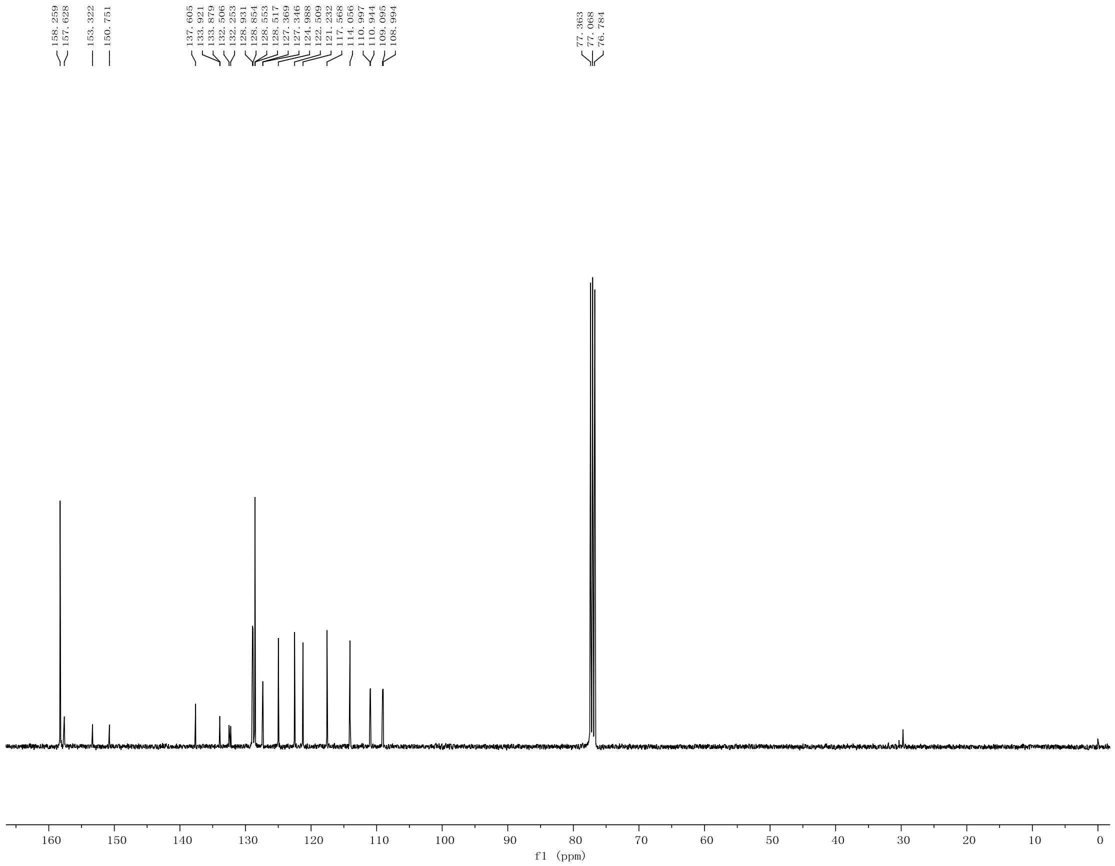

Supplementary Figure 2. ^{1}H and ^{13}C NMR spectra for product 3ab

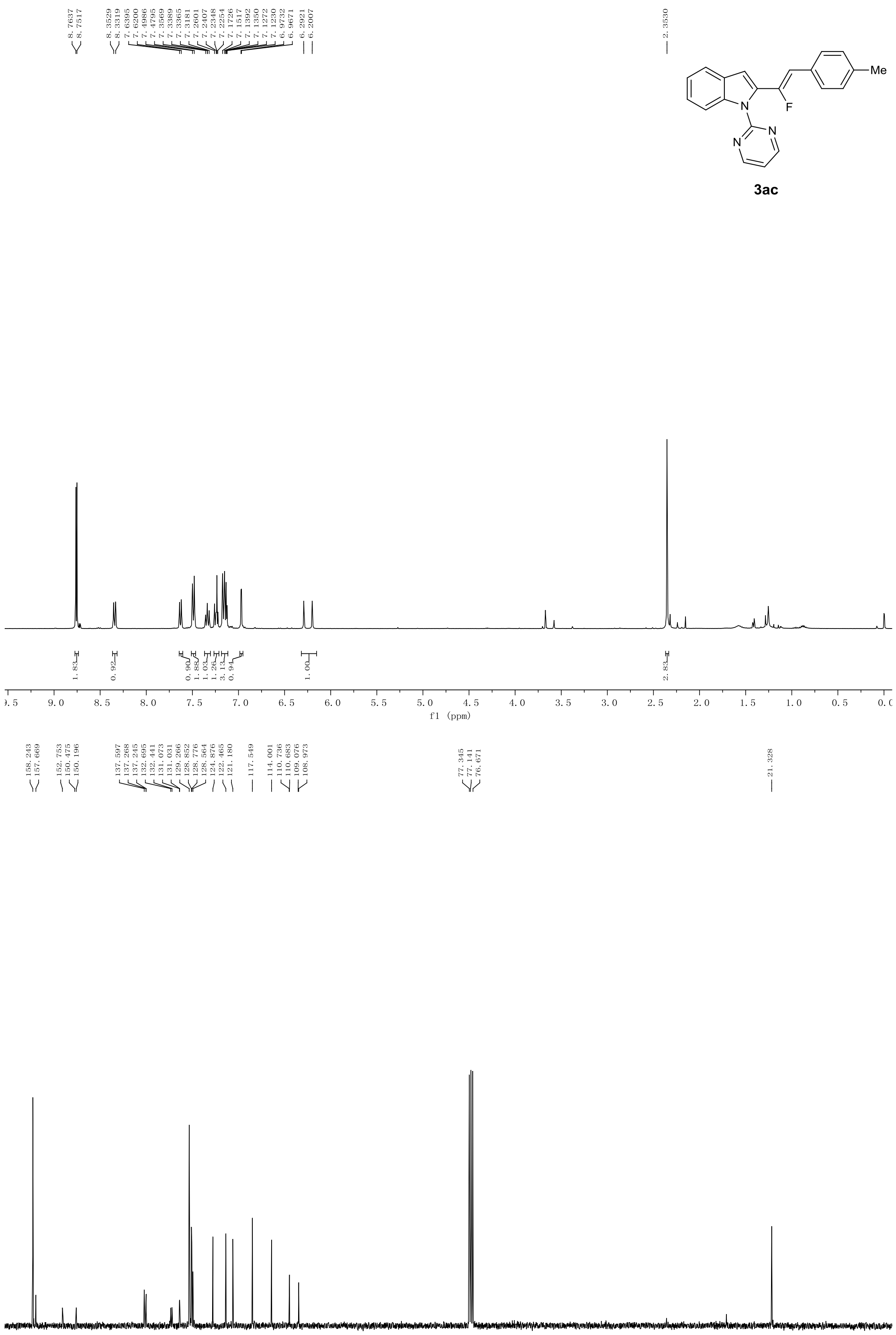

Supplementary Figure 3. ^{1}H and ^{13}C NMR spectra for product 3ac

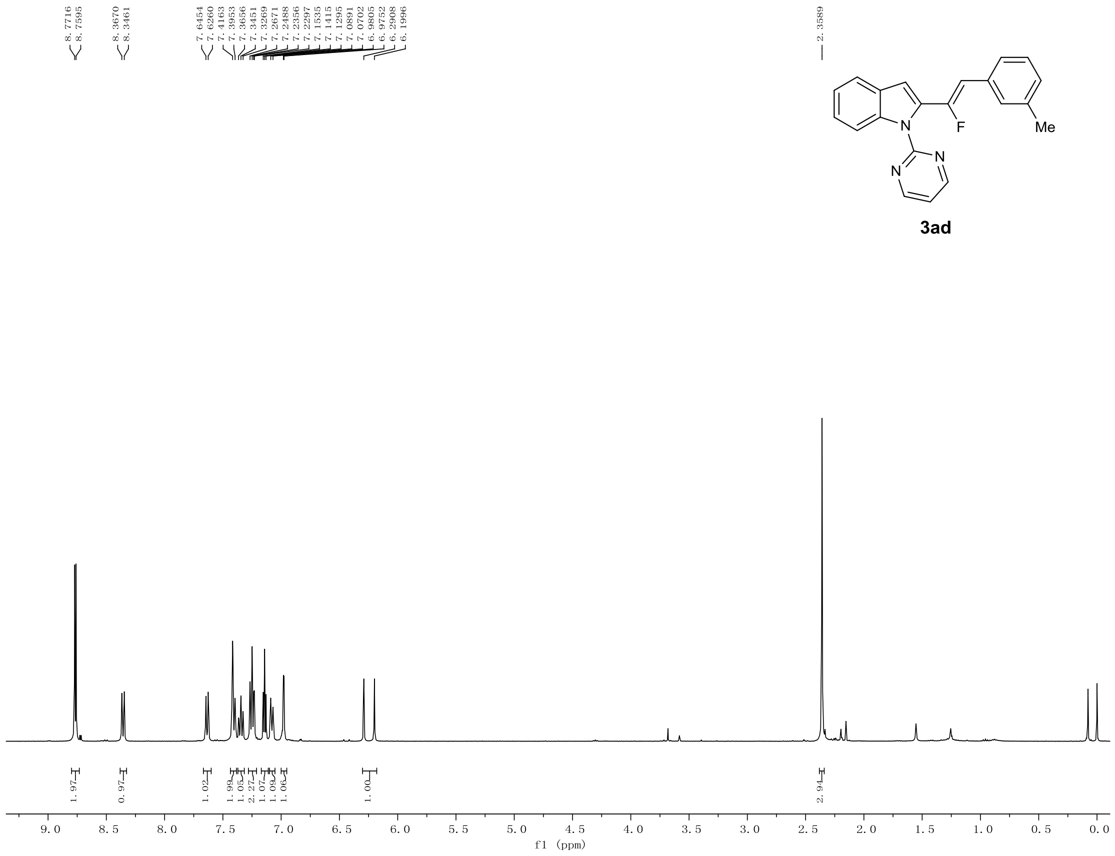

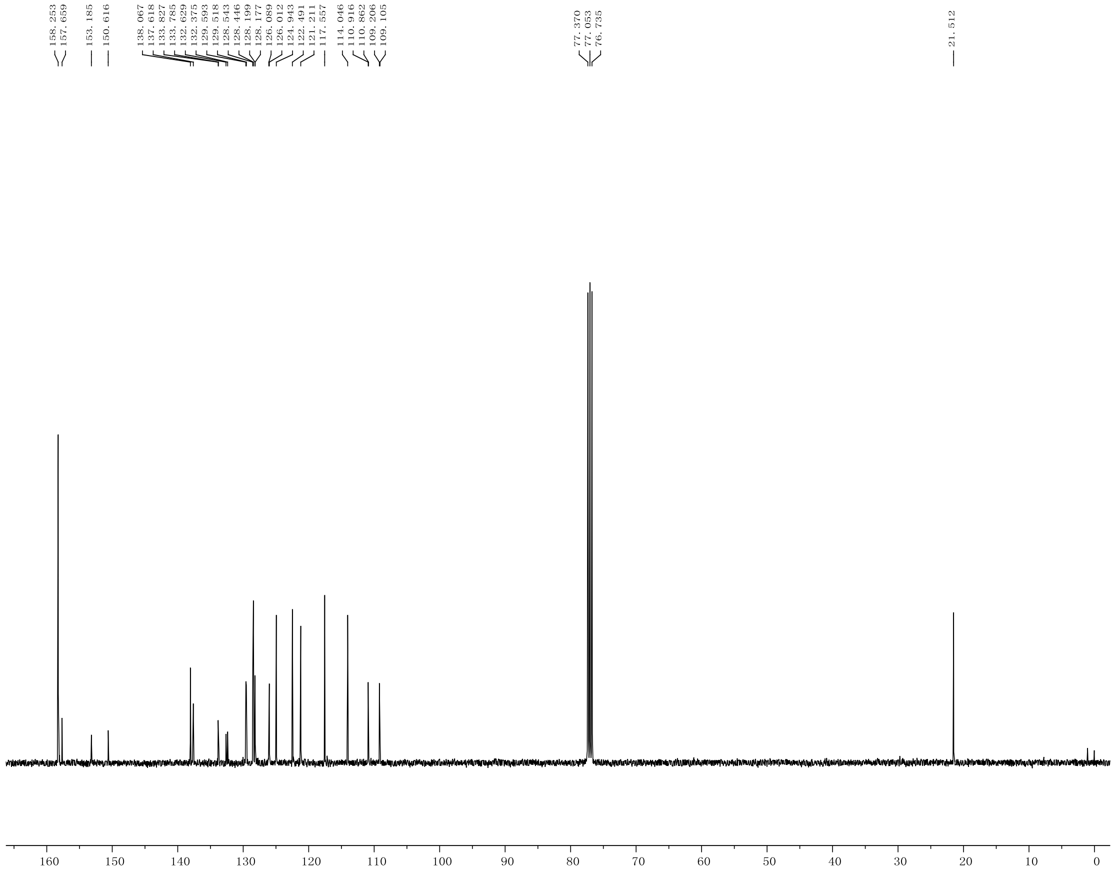

Supplementary Figure 4. ^{1}H and ^{13}C NMR spectra for product 3ad

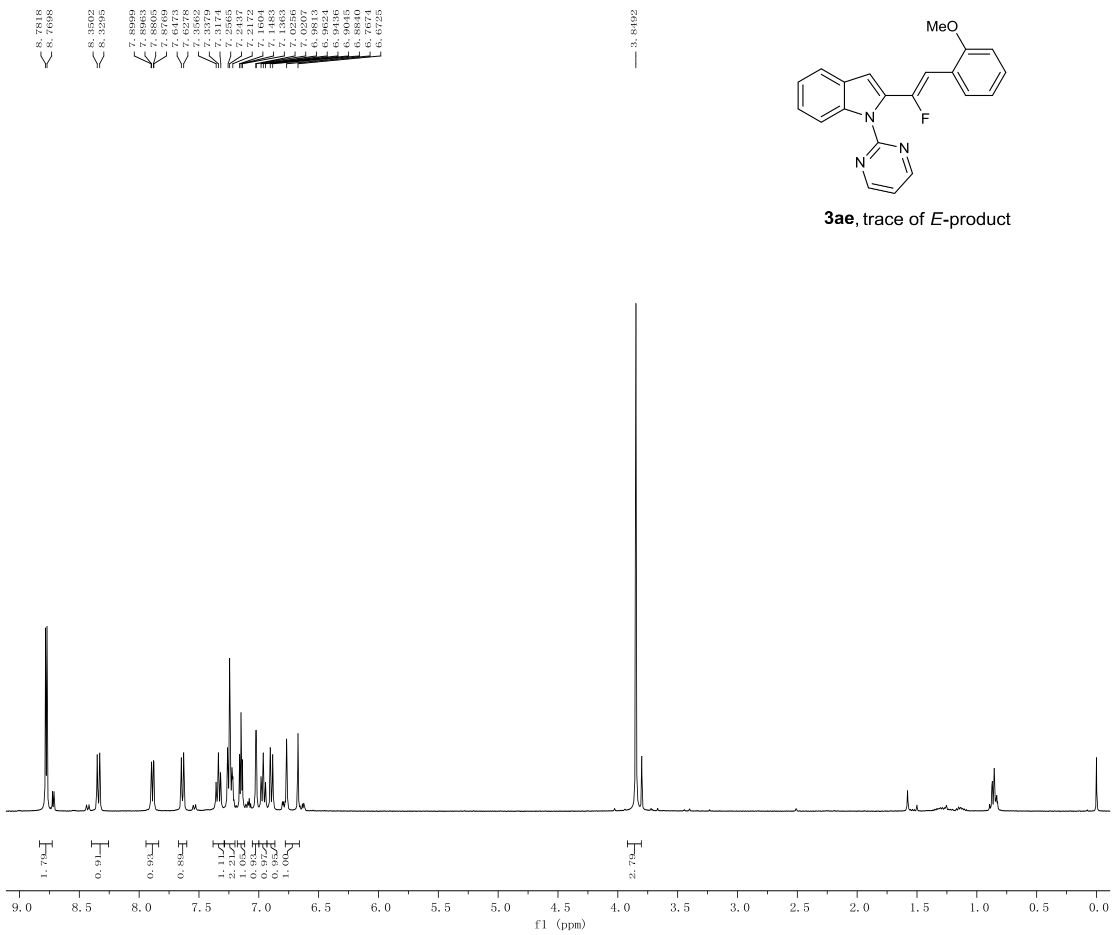

3ae, trace of *E*-product

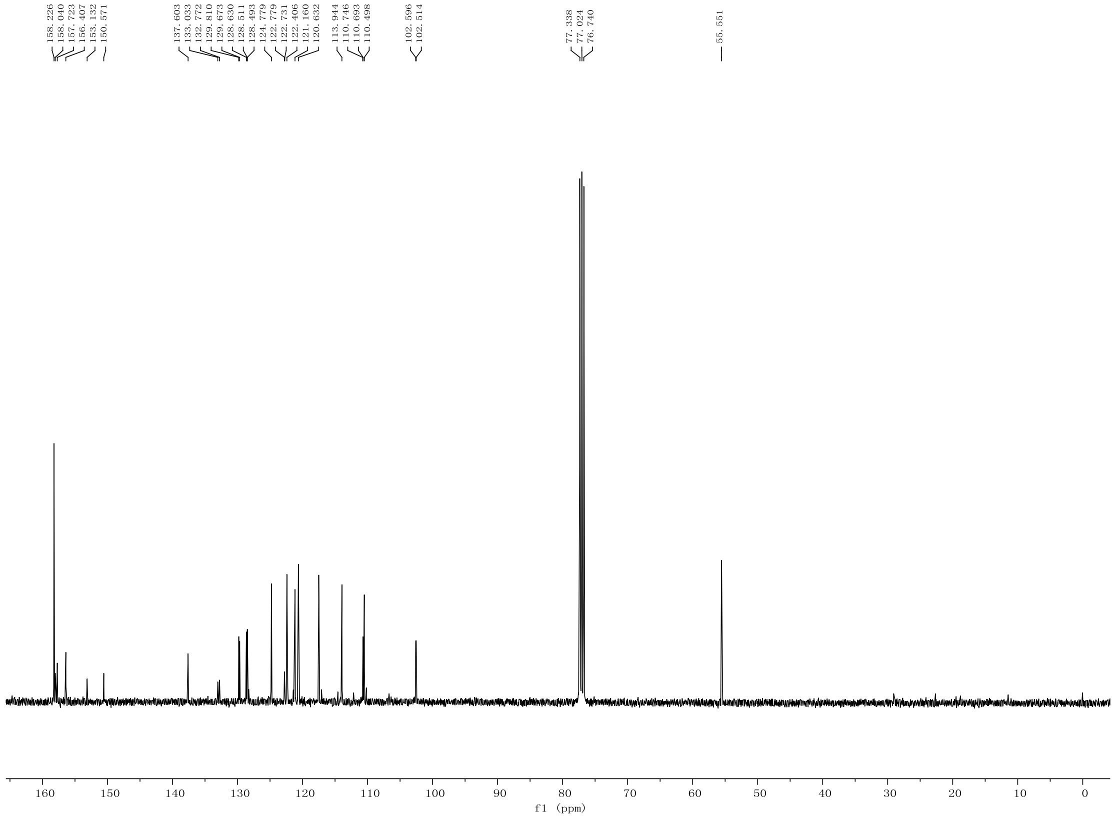

Supplementary Figure 5. ^{1}H and ^{13}C NMR spectra for product 3ae

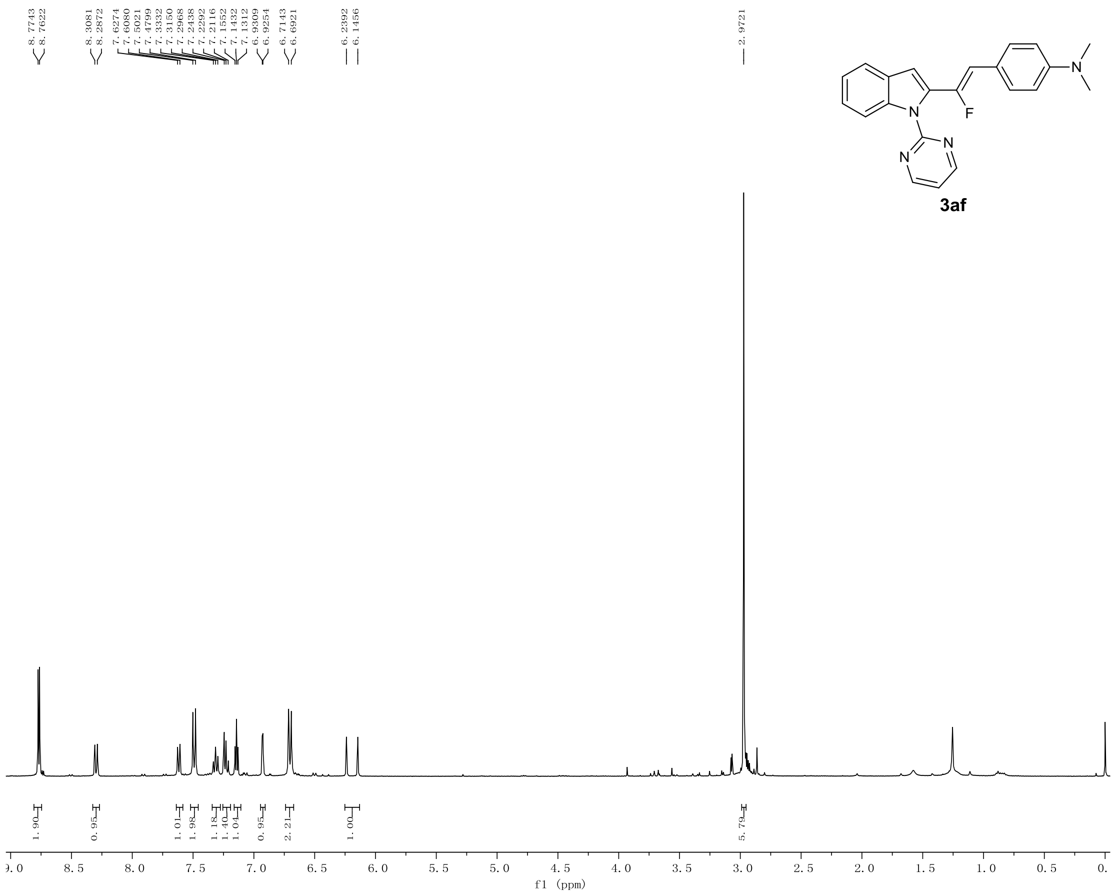

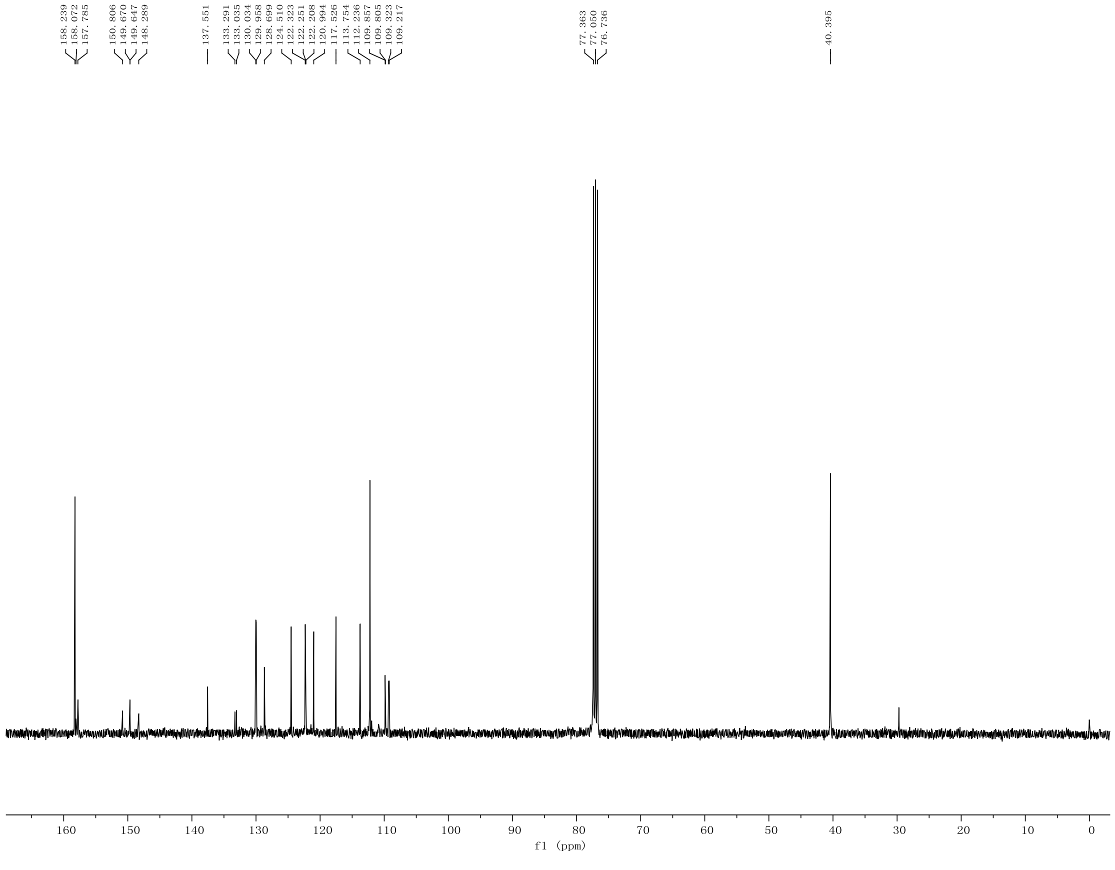

Supplementary Figure 6. **^{1}H and ^{13}C NMR spectra for product 3af**

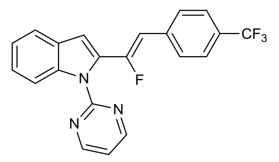

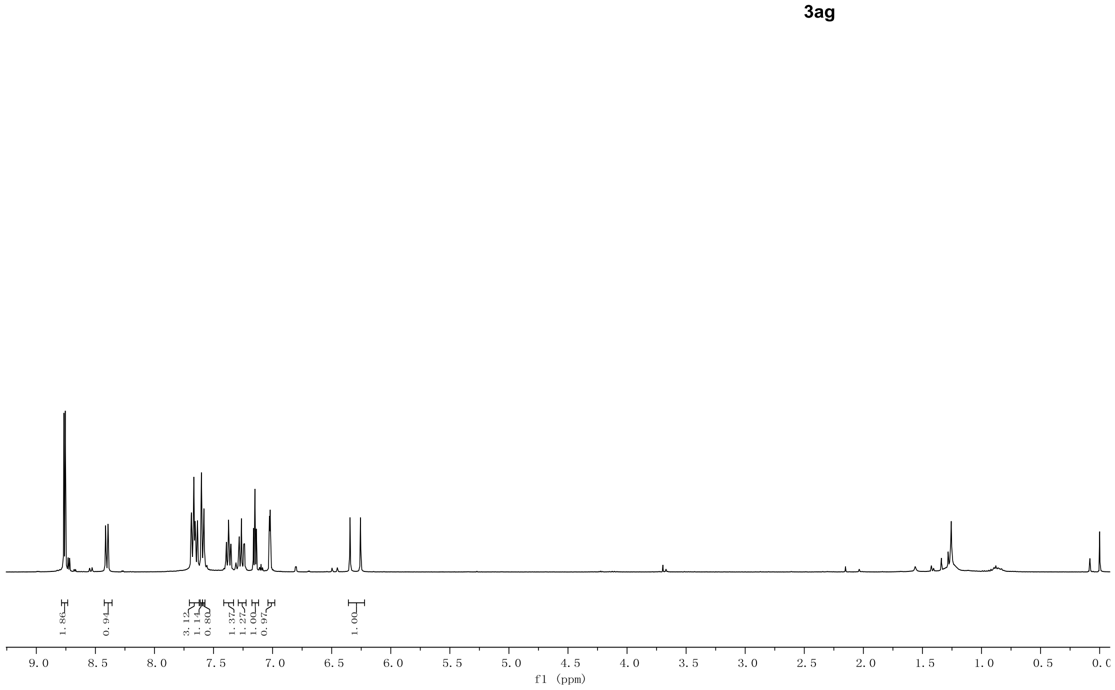

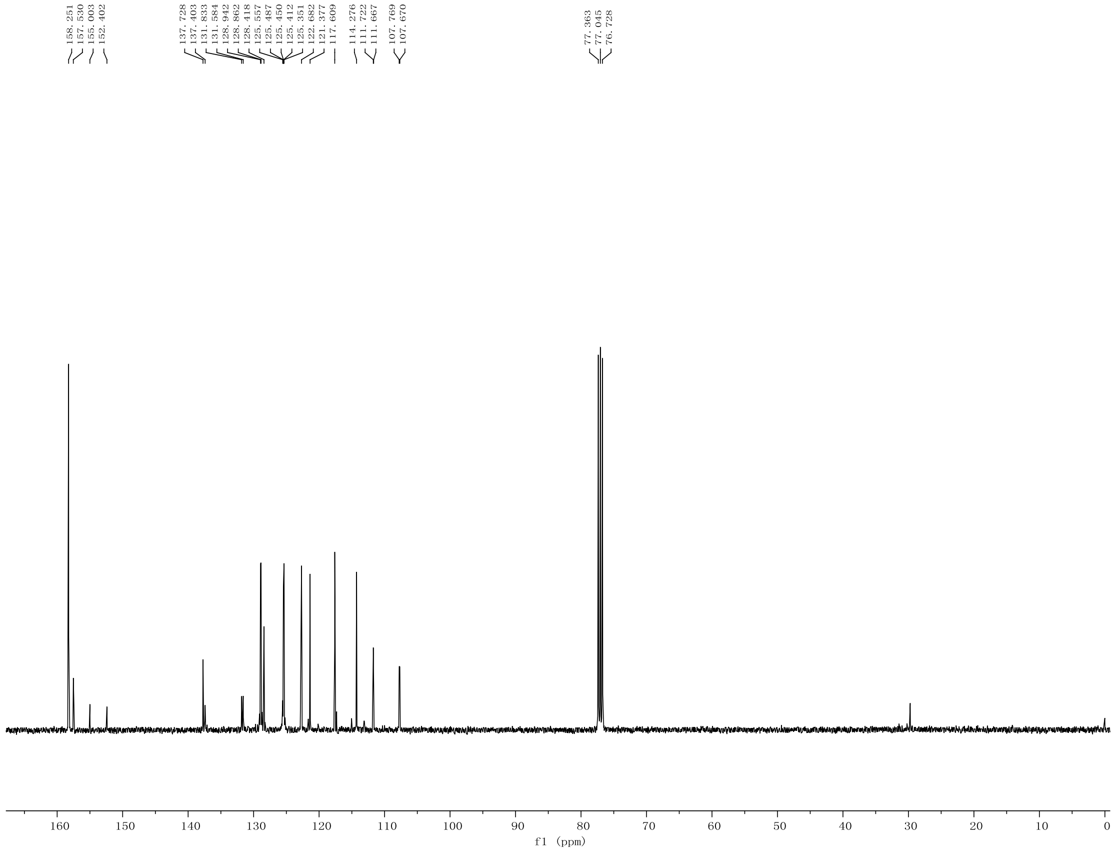

Supplementary Figure 7. ^{1}H and ^{13}C NMR spectra for product 3ag

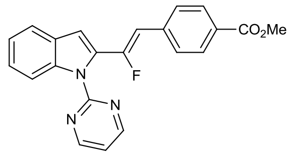

3ah

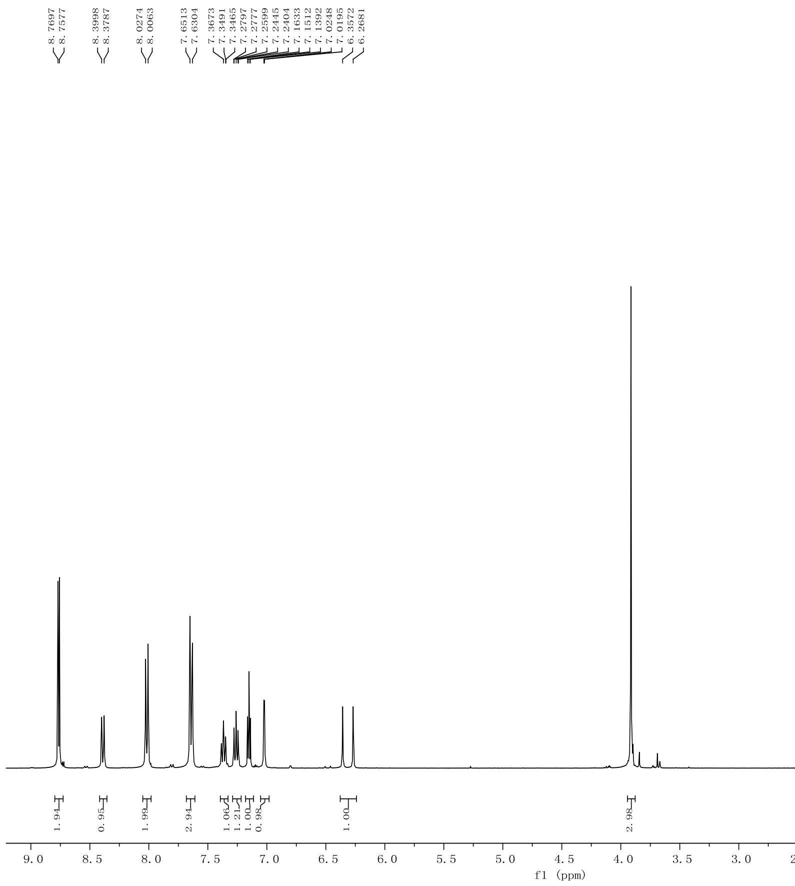

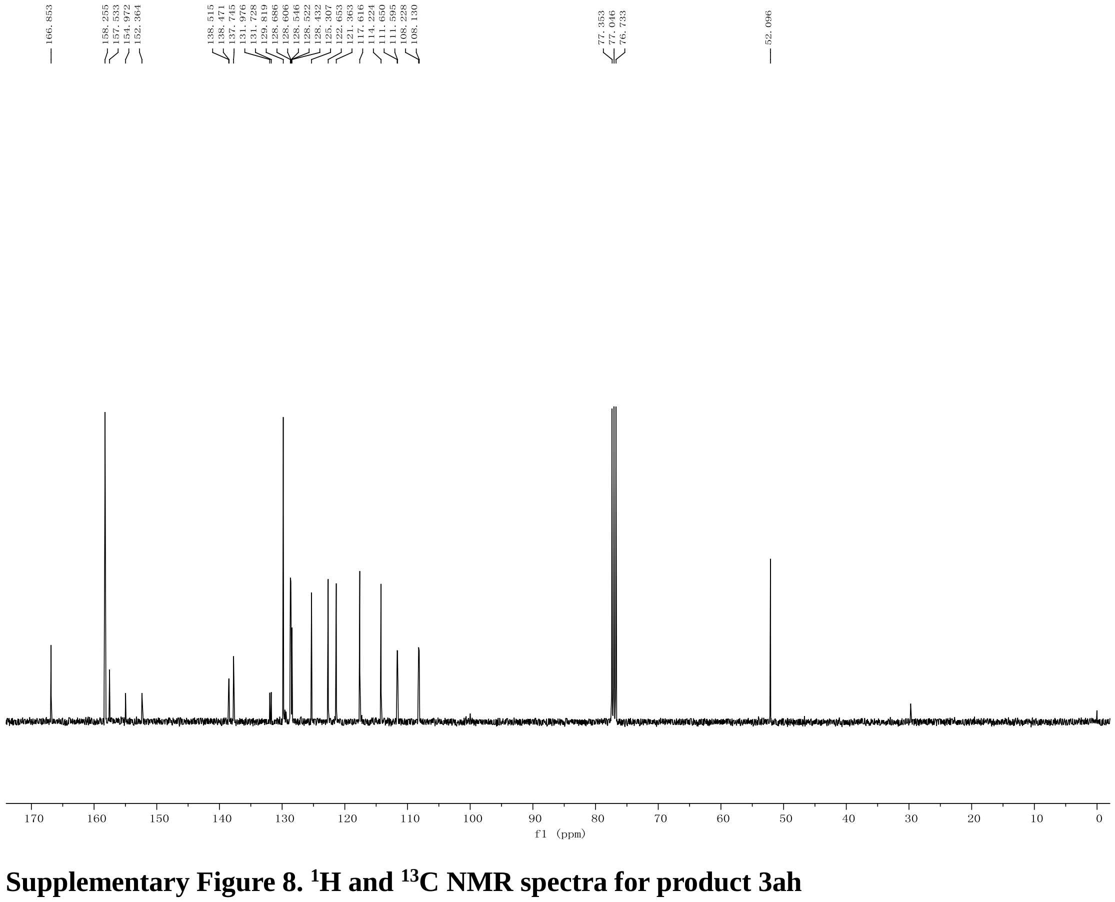

Supplementary Figure 8. ^{1}H and ^{13}C NMR spectra for product 3ah

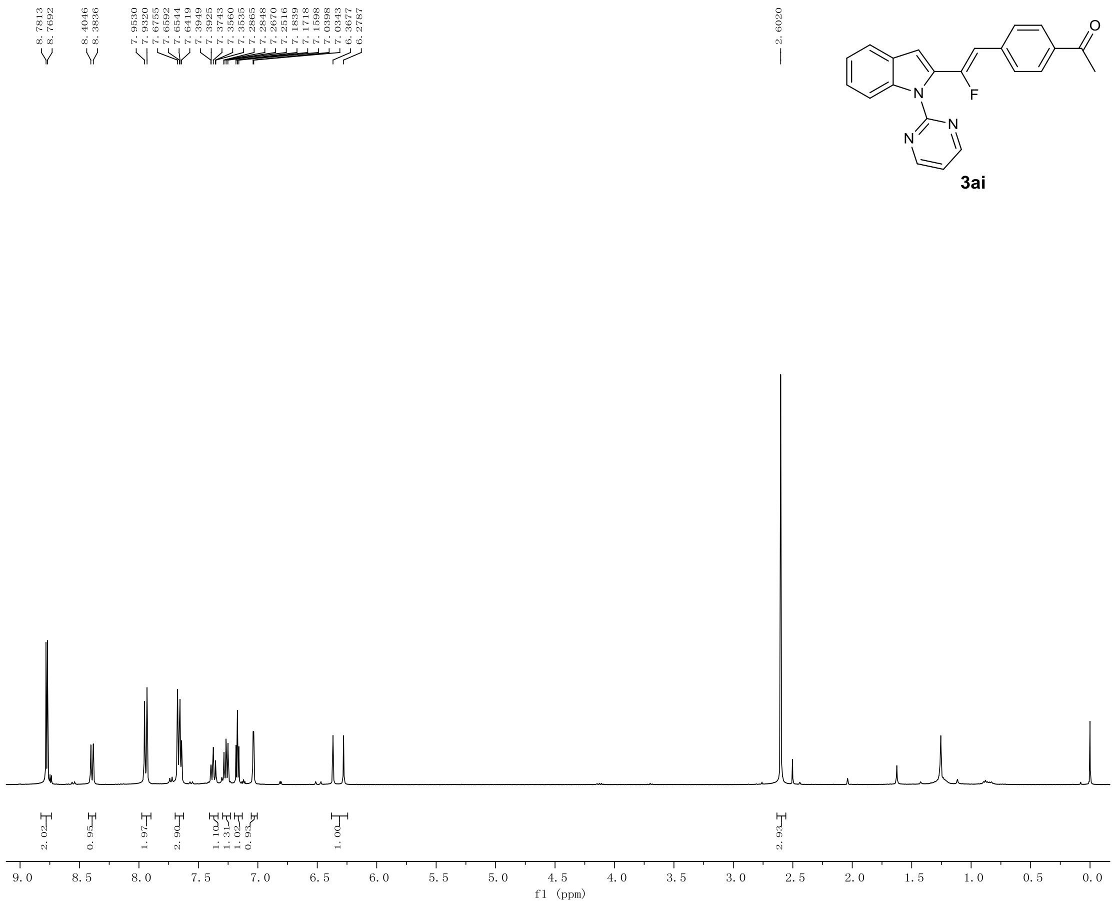

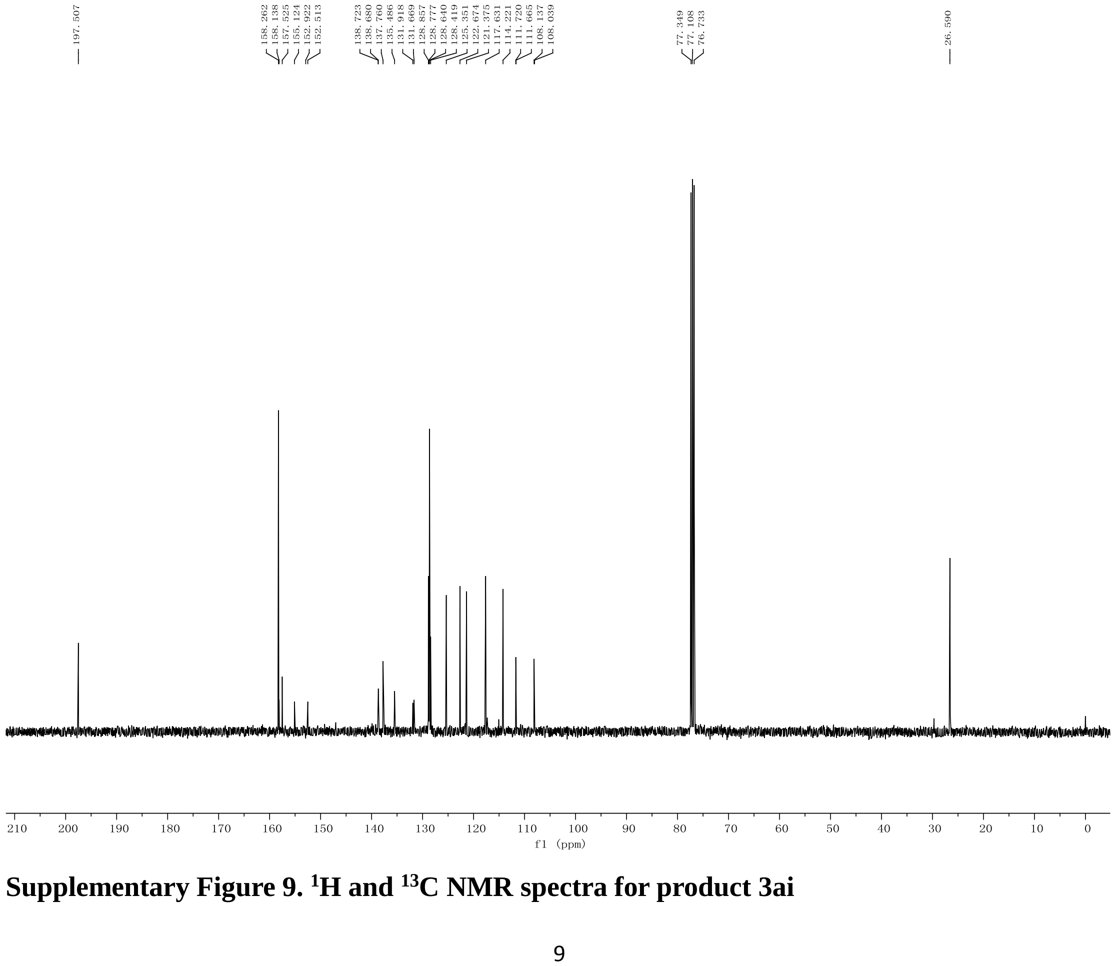

Supplementary Figure 9. ^{1}H and ^{13}C NMR spectra for product 3ai

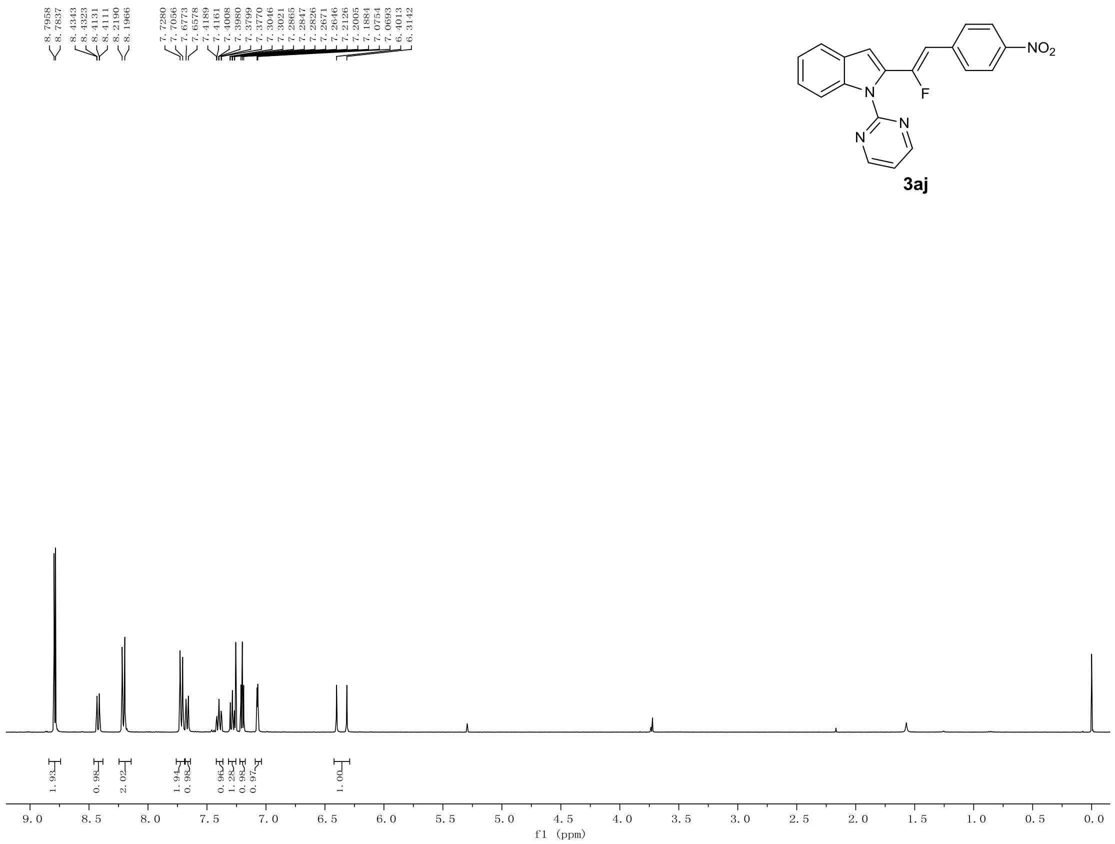

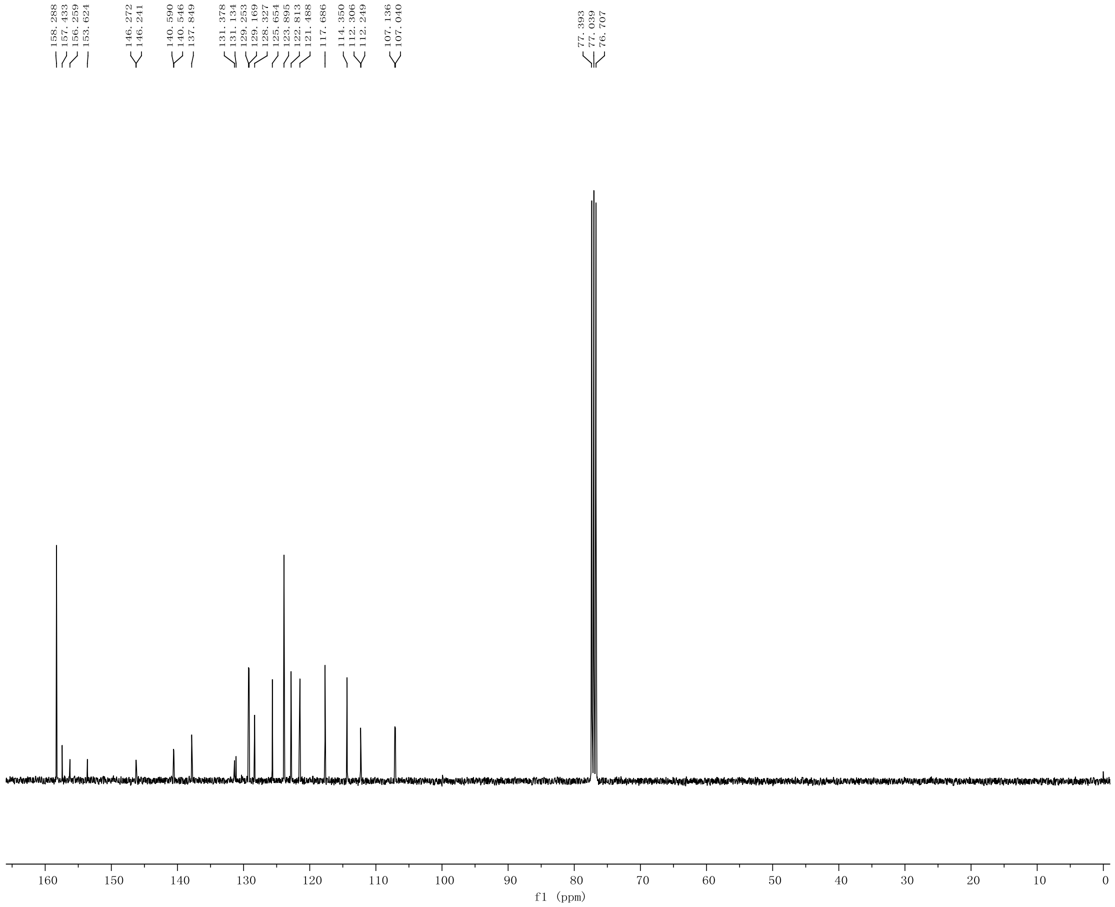

Supplementary Figure 10. ^{1}H and ^{13}C NMR spectra for product 3aj

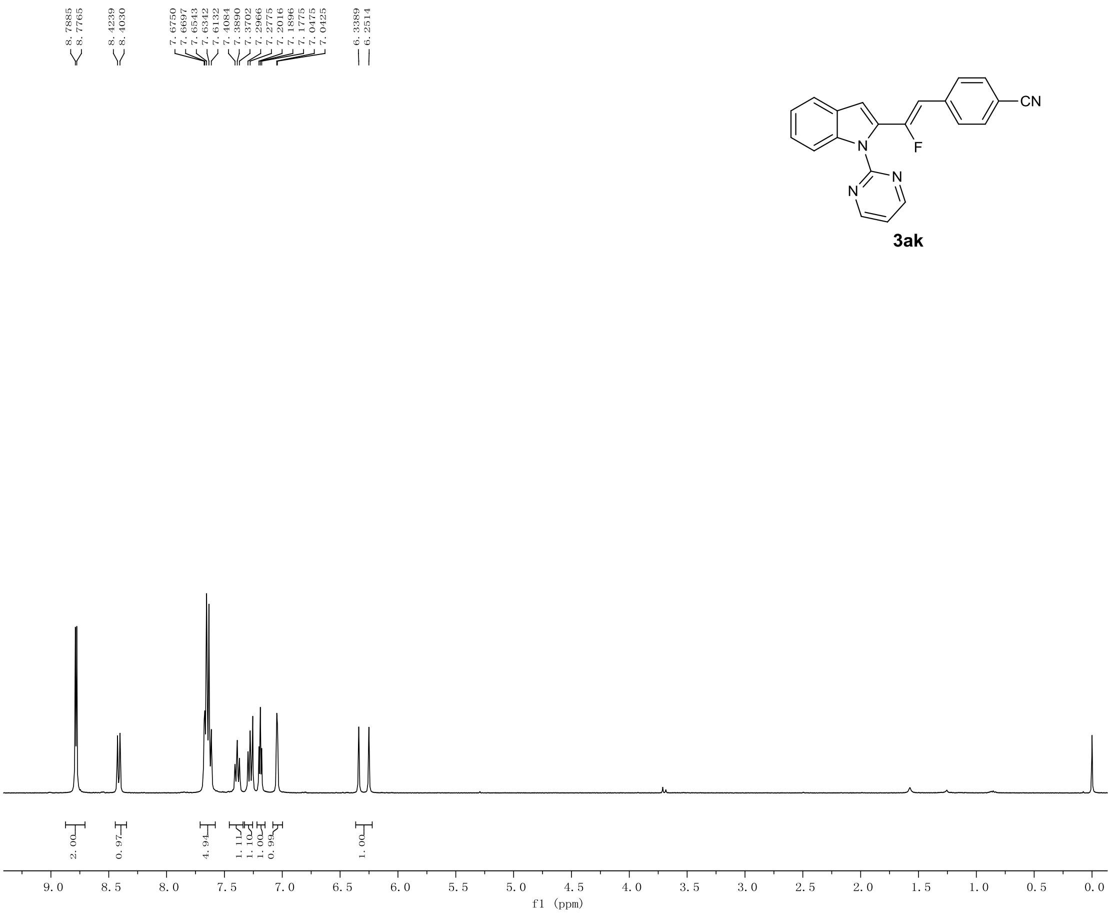

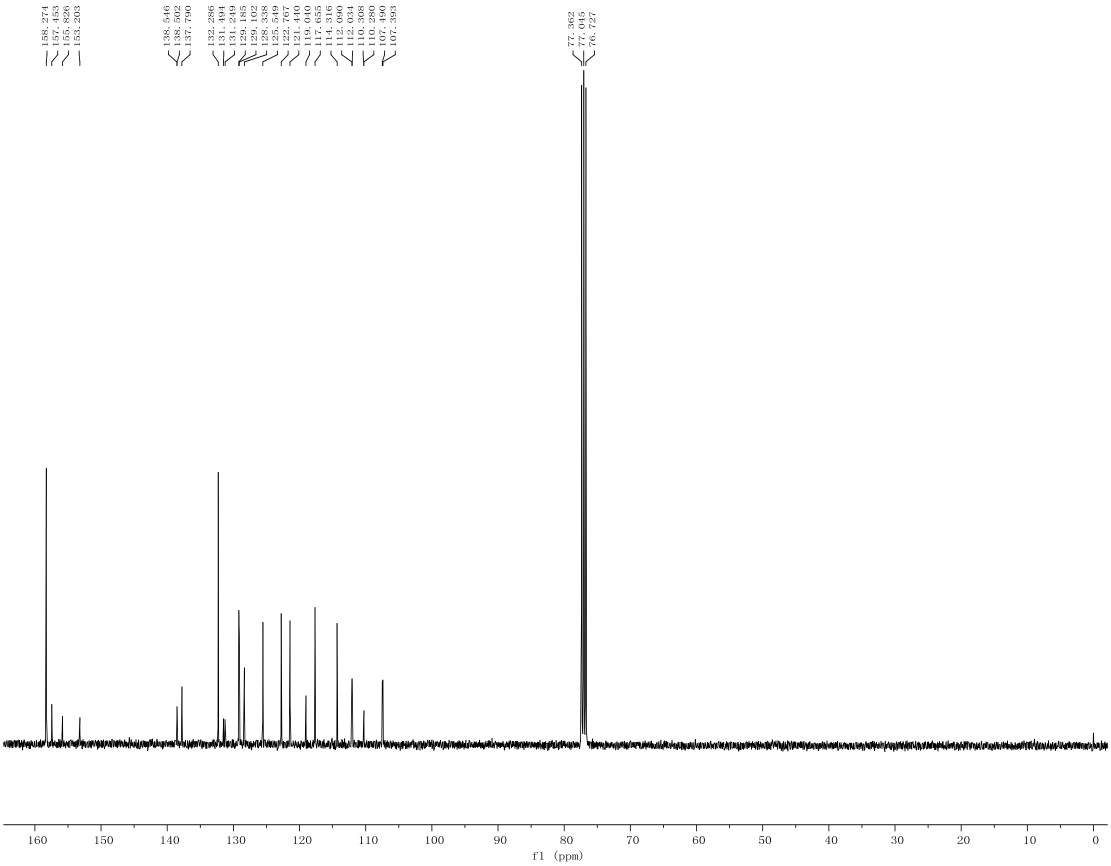

Supplementary Figure 11. ^{1}H and ^{13}C NMR spectra for product 3ak

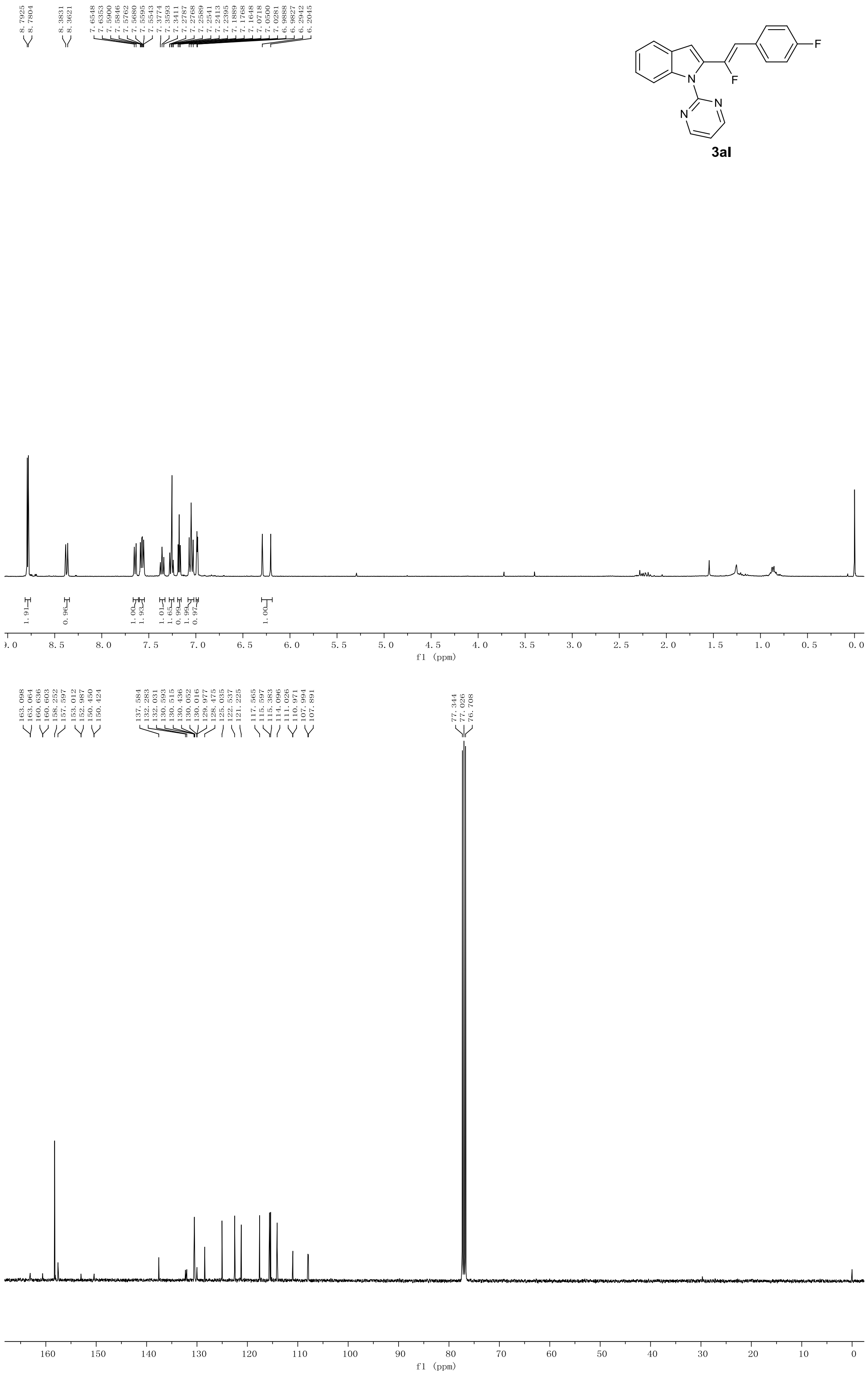

3al

Supplementary Figure 12. ^{1}H and ^{13}C NMR spectra for product 3al

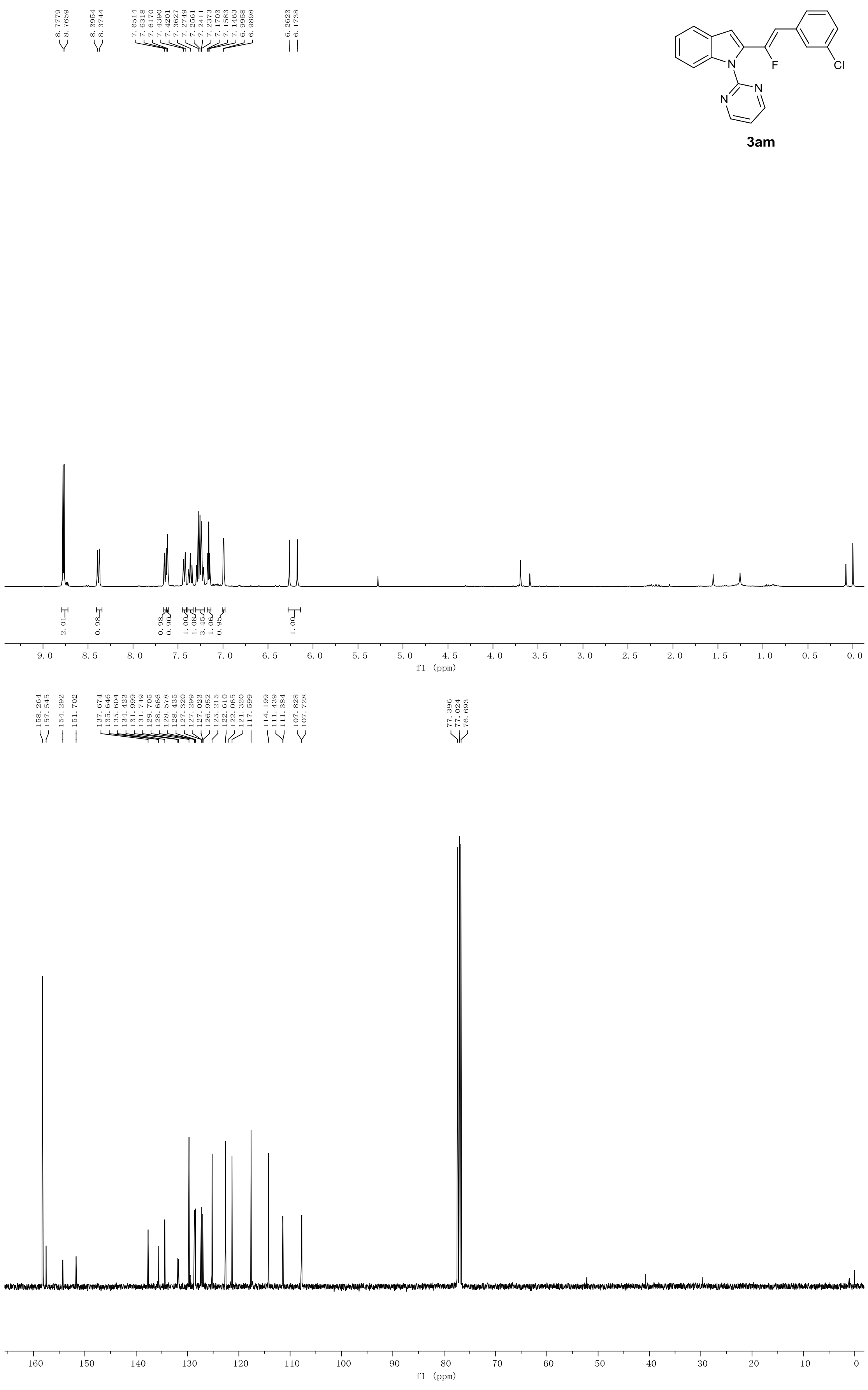

Supplementary Figure 13. ^{1}H and ^{13}C NMR spectra for product 3am

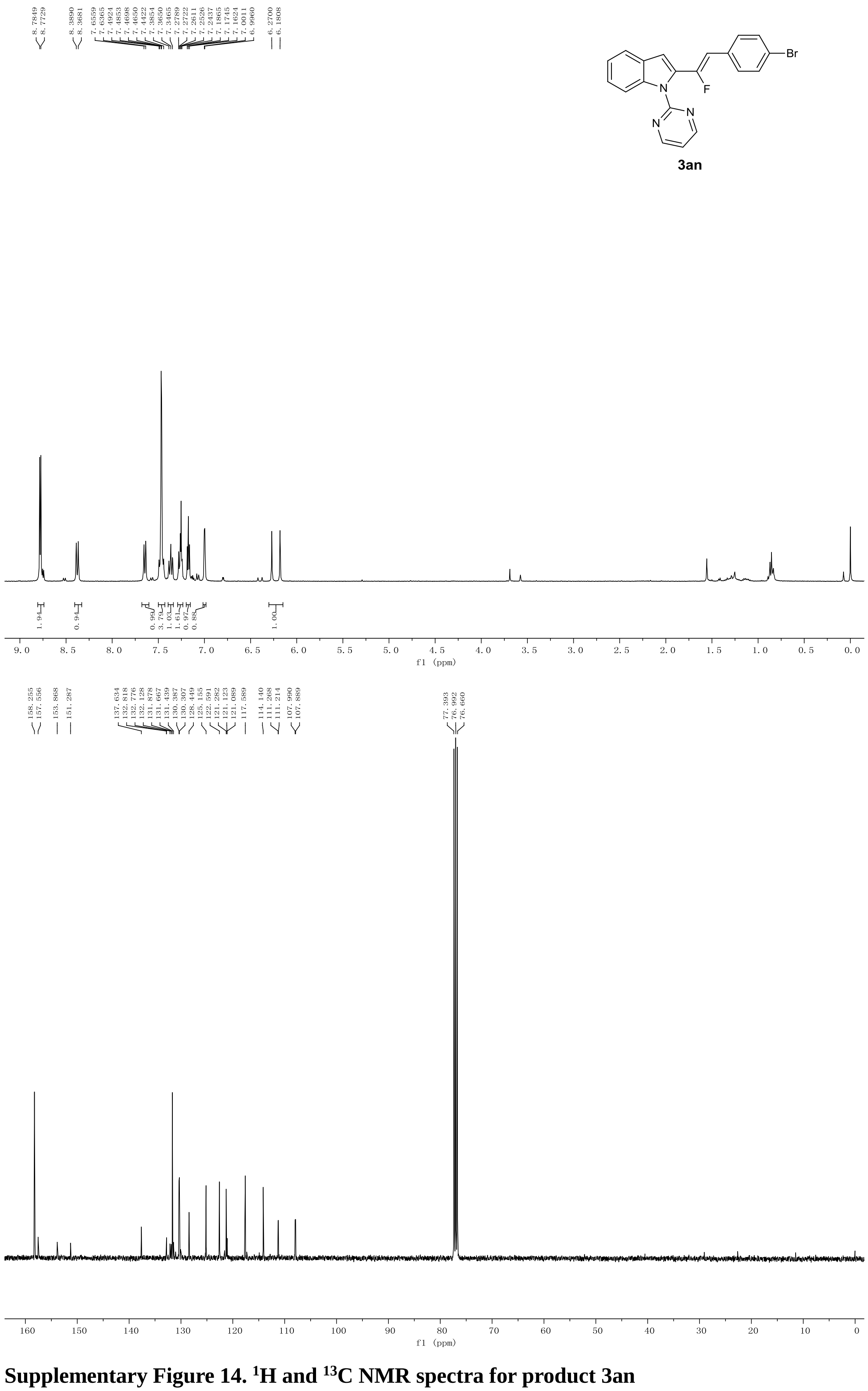

Supplementary Figure 14. ^{1}H and ^{13}C NMR spectra for product 3an

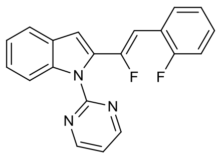

3ao

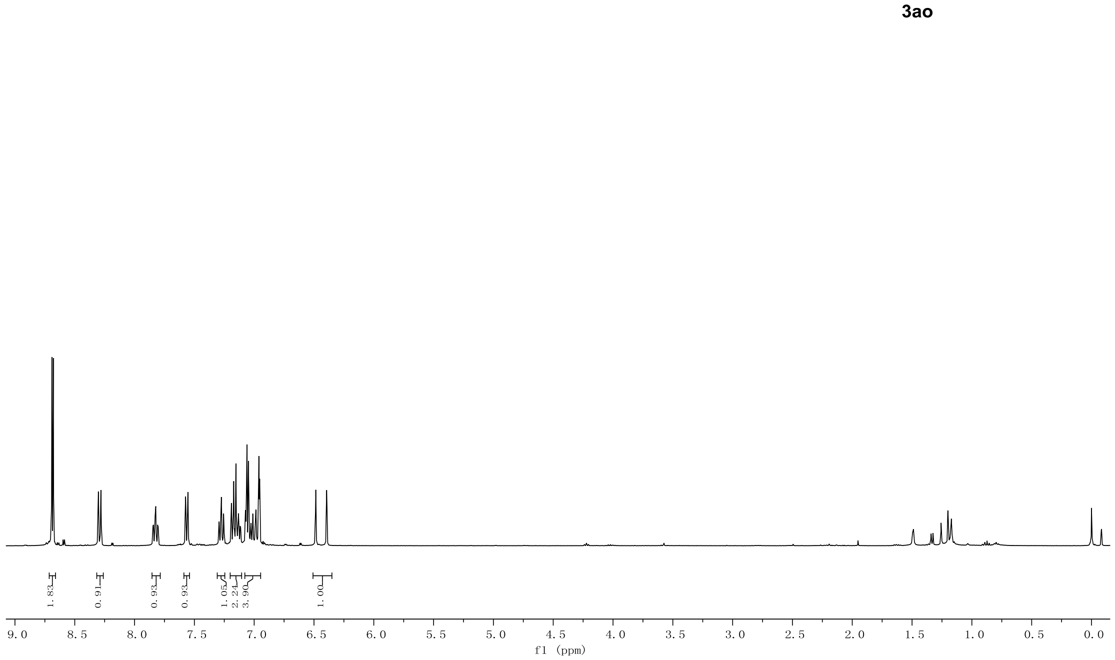

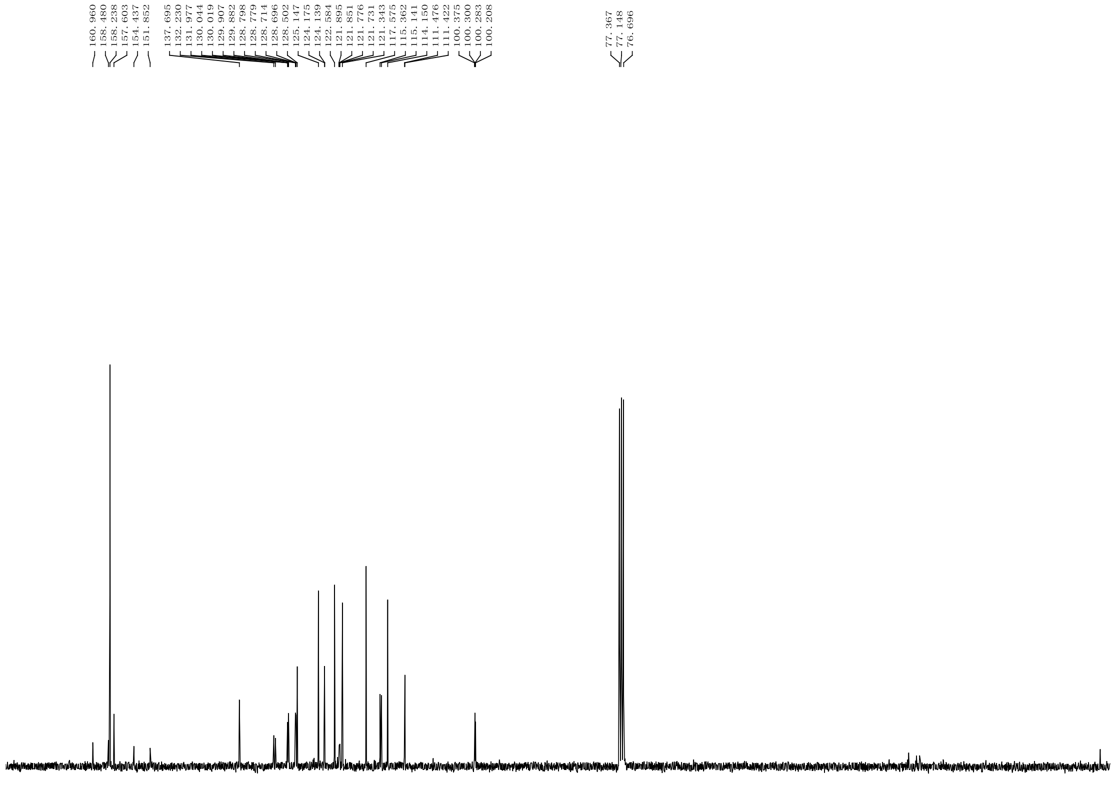

Supplementary Figure 15. ^{1}H and ^{13}C NMR spectra for product 3ao

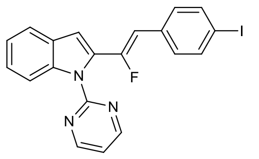

3ap

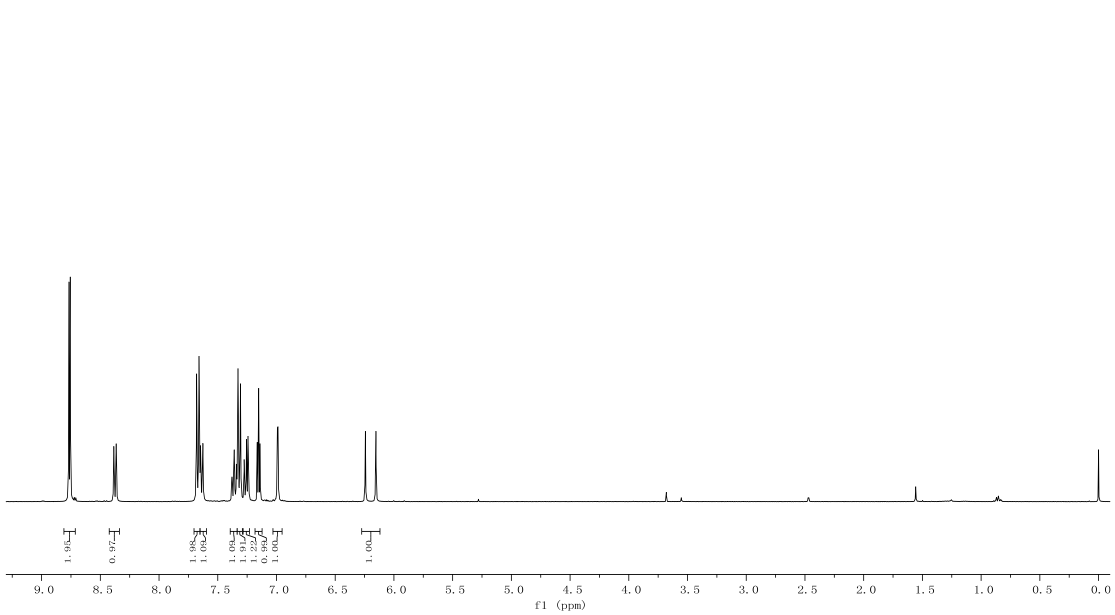

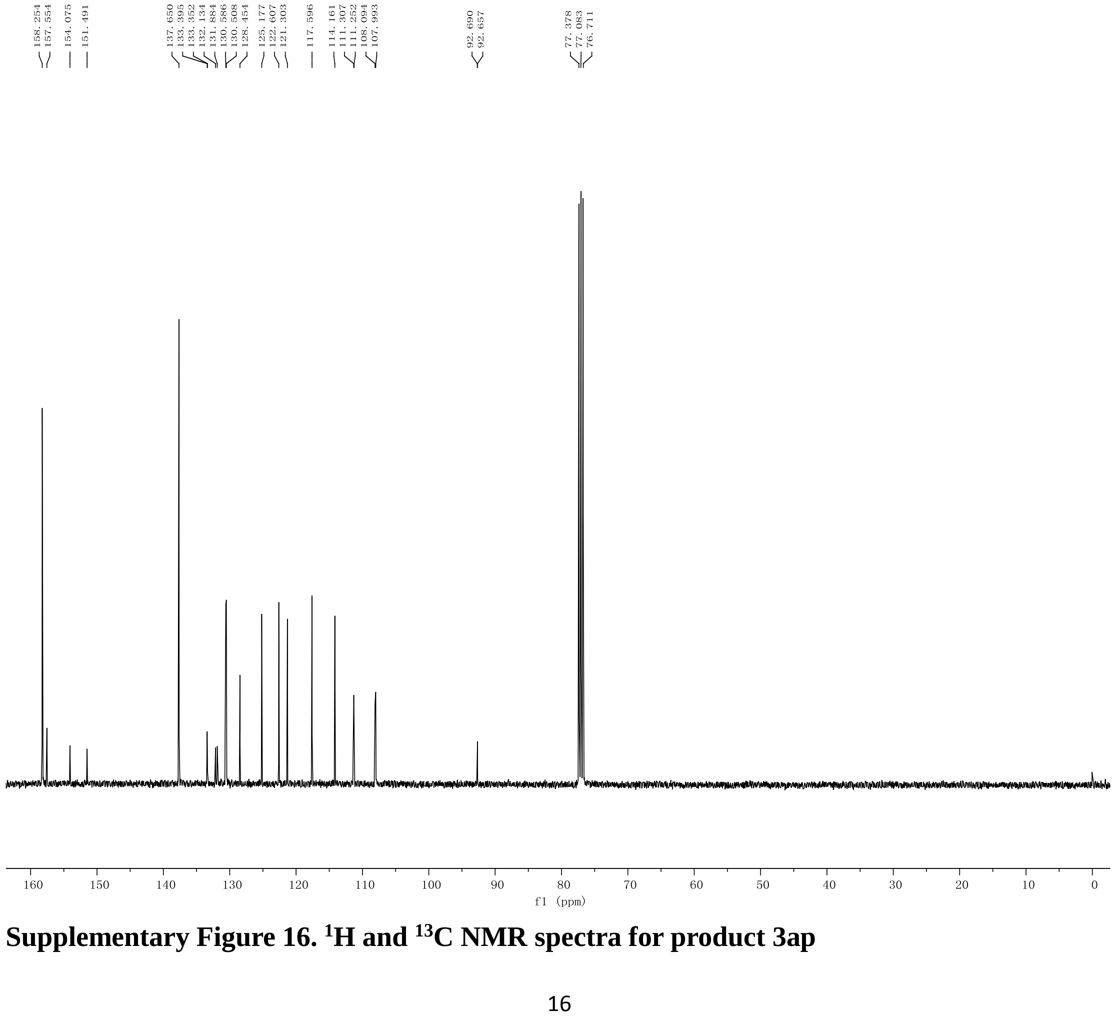

Supplementary Figure 16. ^{1}H and ^{13}C NMR spectra for product 3ap

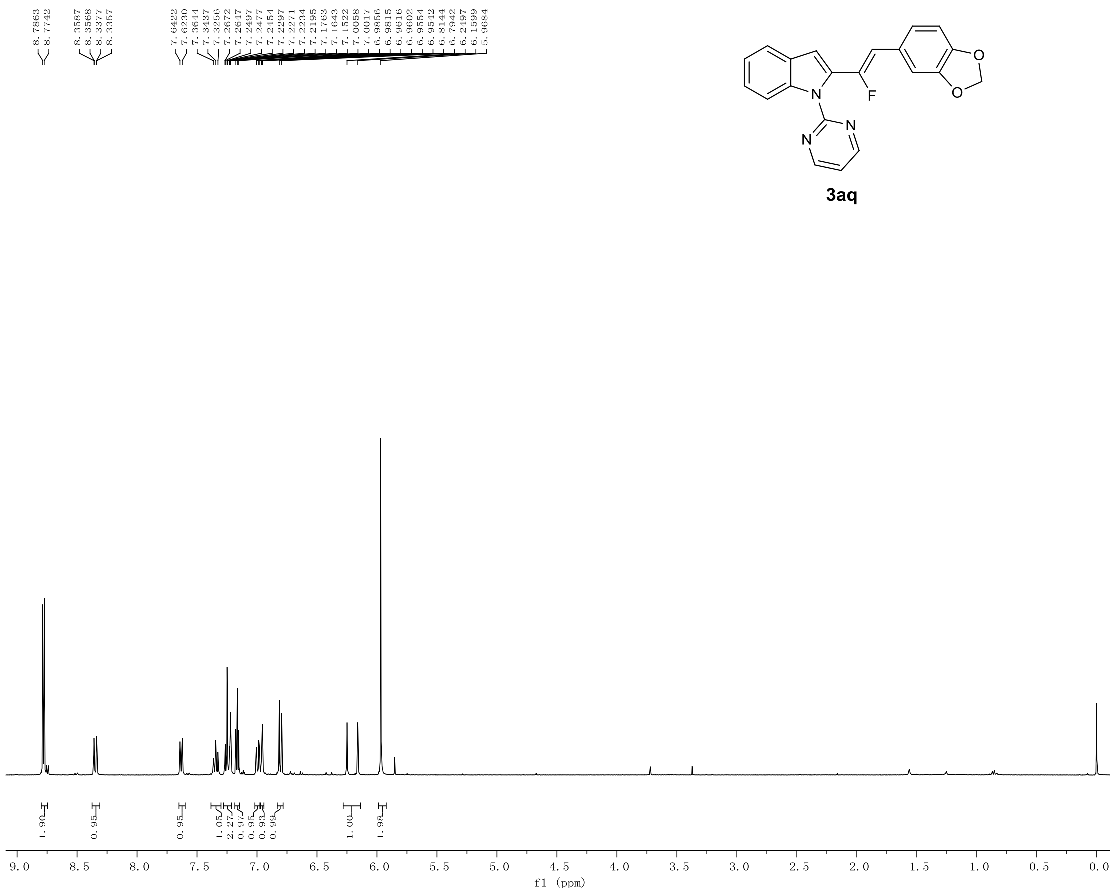

3aq

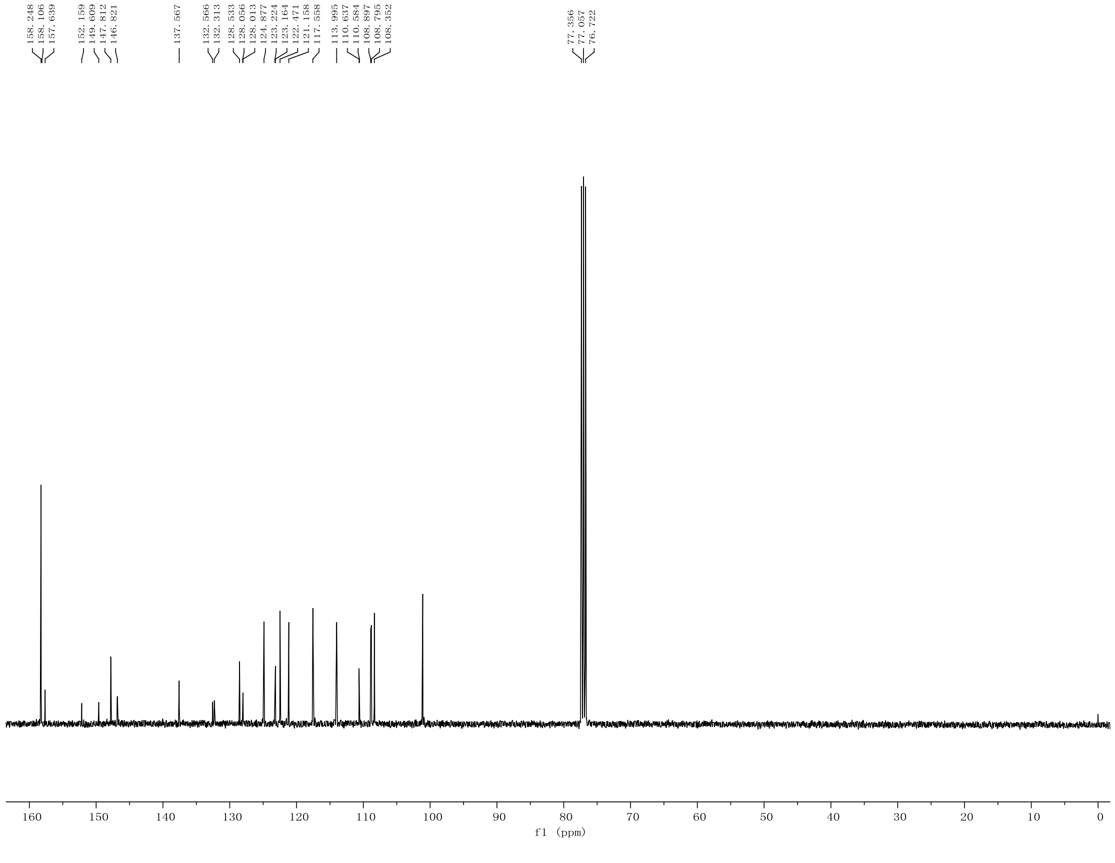

Supplementary Figure 17. ^{1}H and ^{13}C NMR spectra for product 3aq

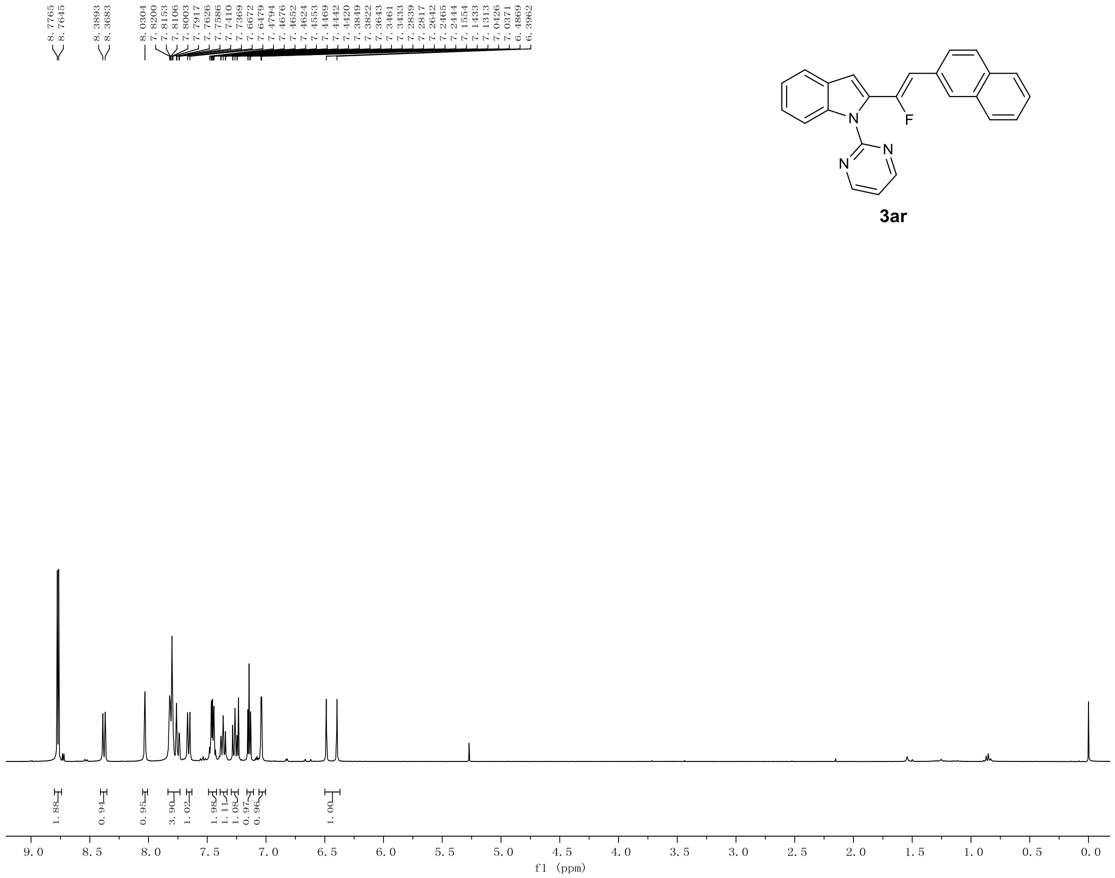

3ar

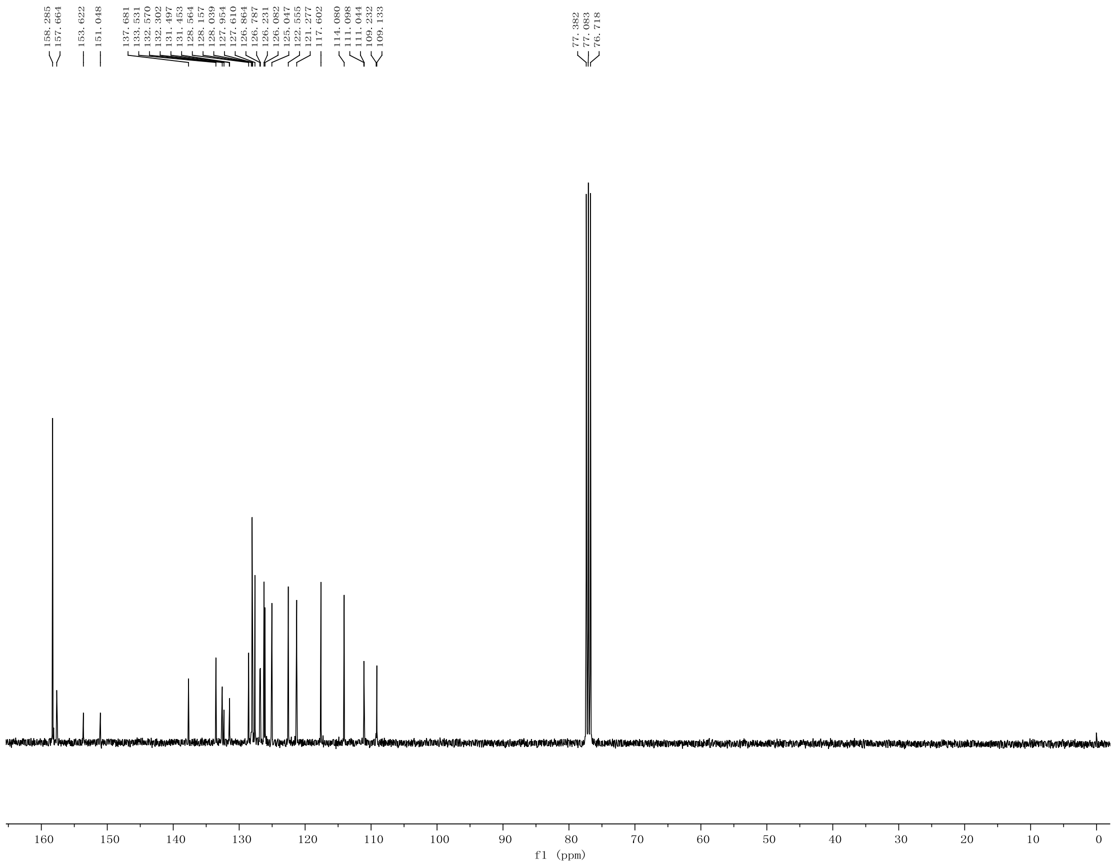

Supplementary Figure 18. ^{1}H and ^{13}C NMR spectra for product 3ar

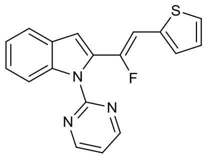

3as

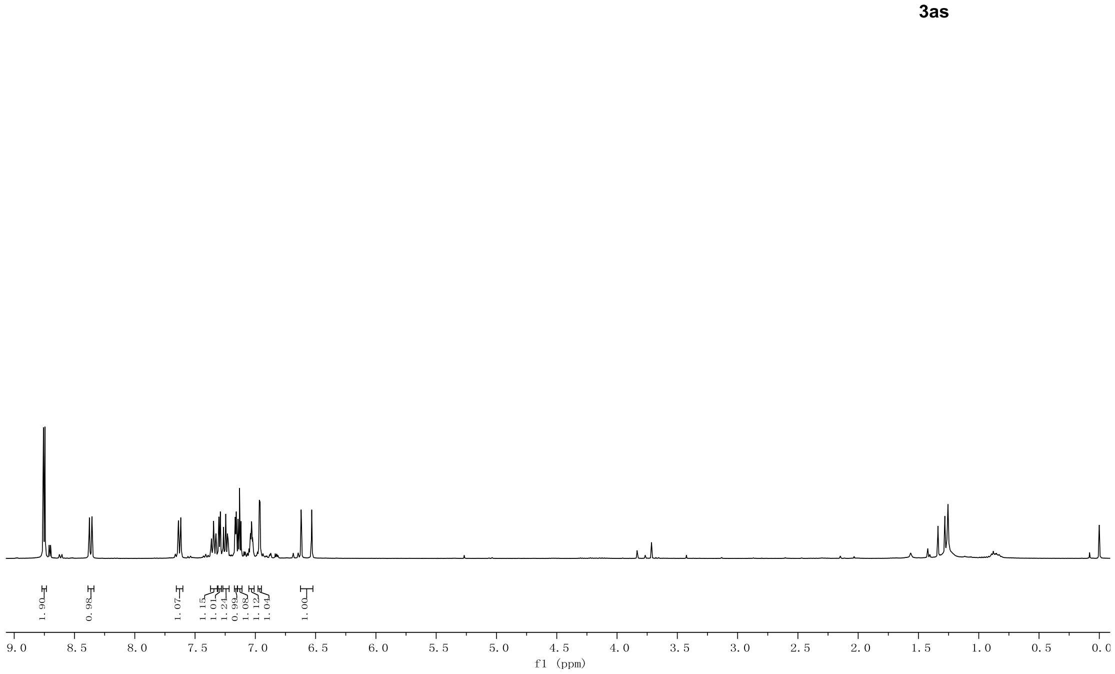

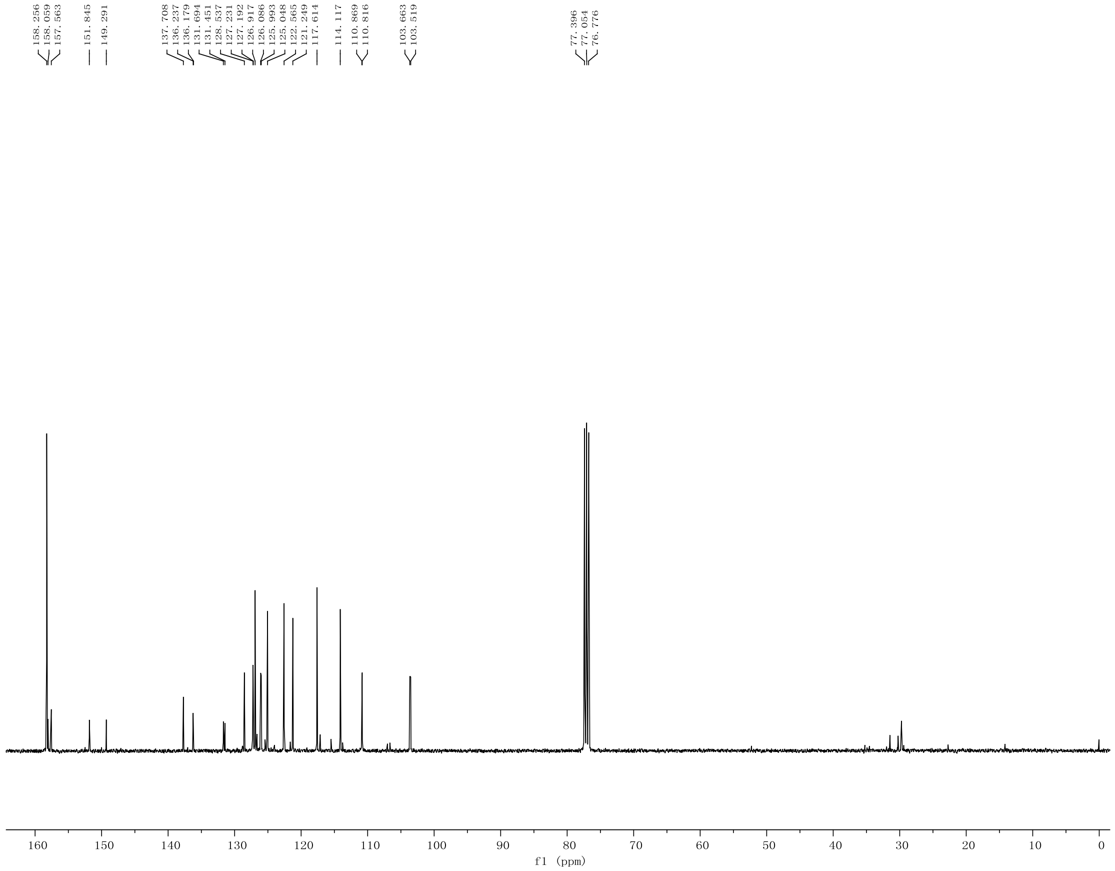

Supplementary Figure 19. ^{1}H and ^{13}C NMR spectra for product 3as

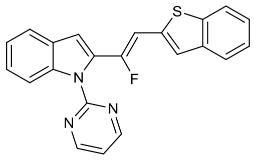

3at

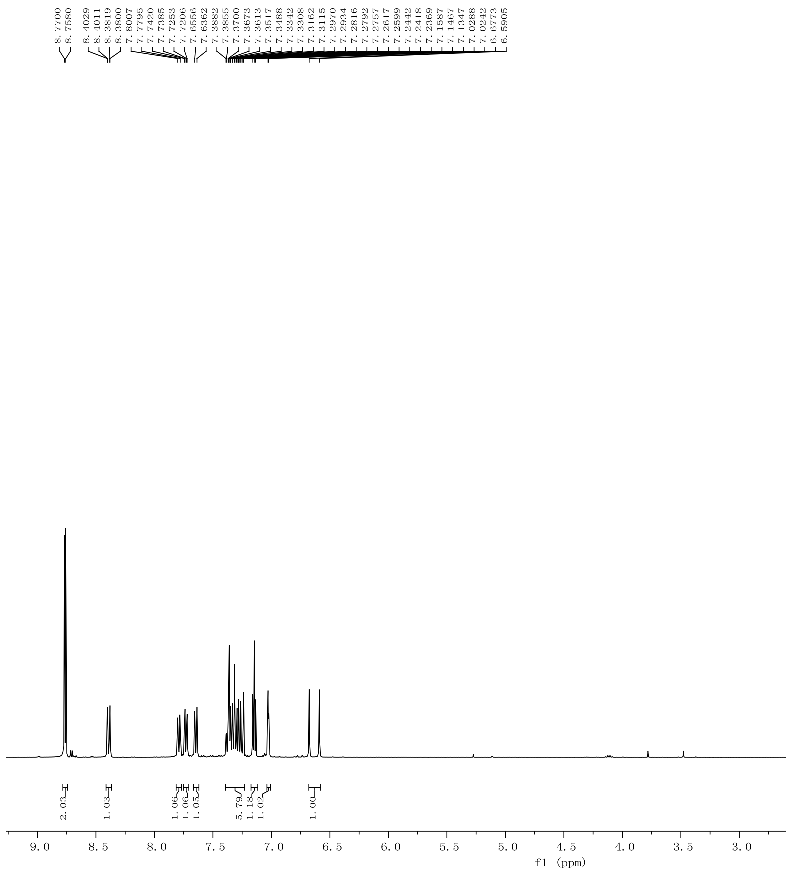

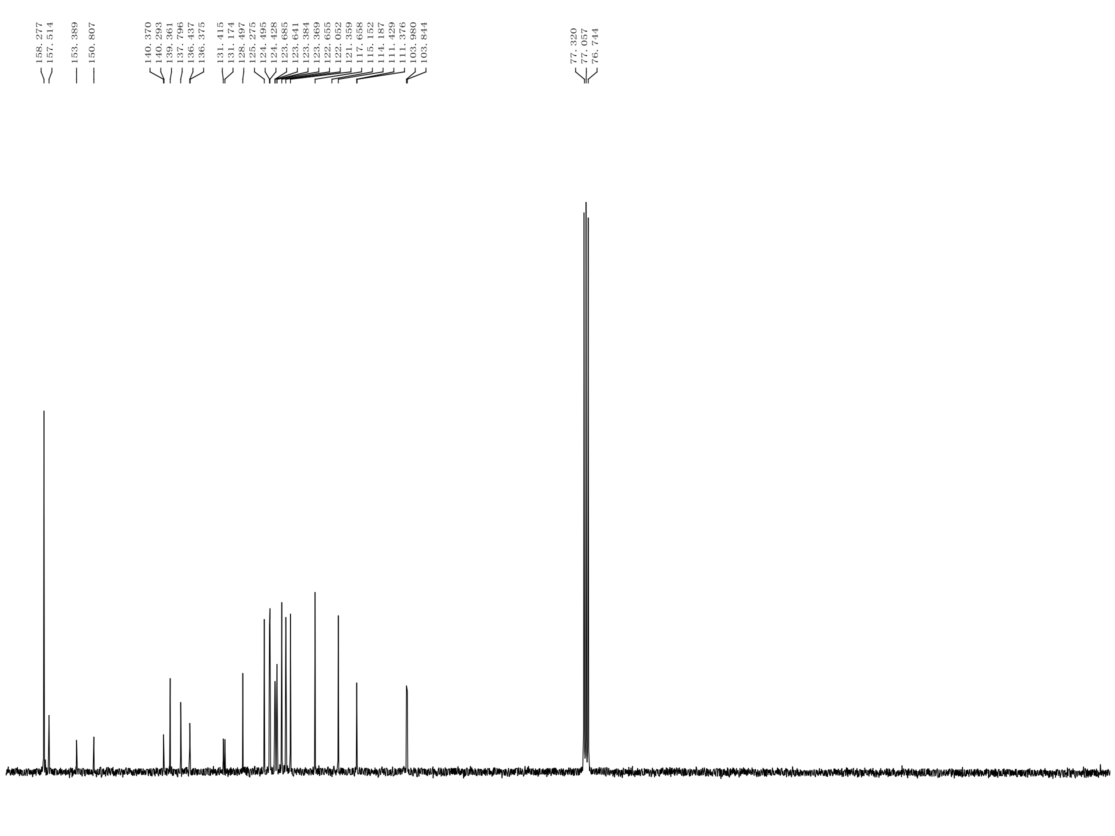

Supplementary Figure 20. ^{1}H and ^{13}C NMR spectra for product 3at

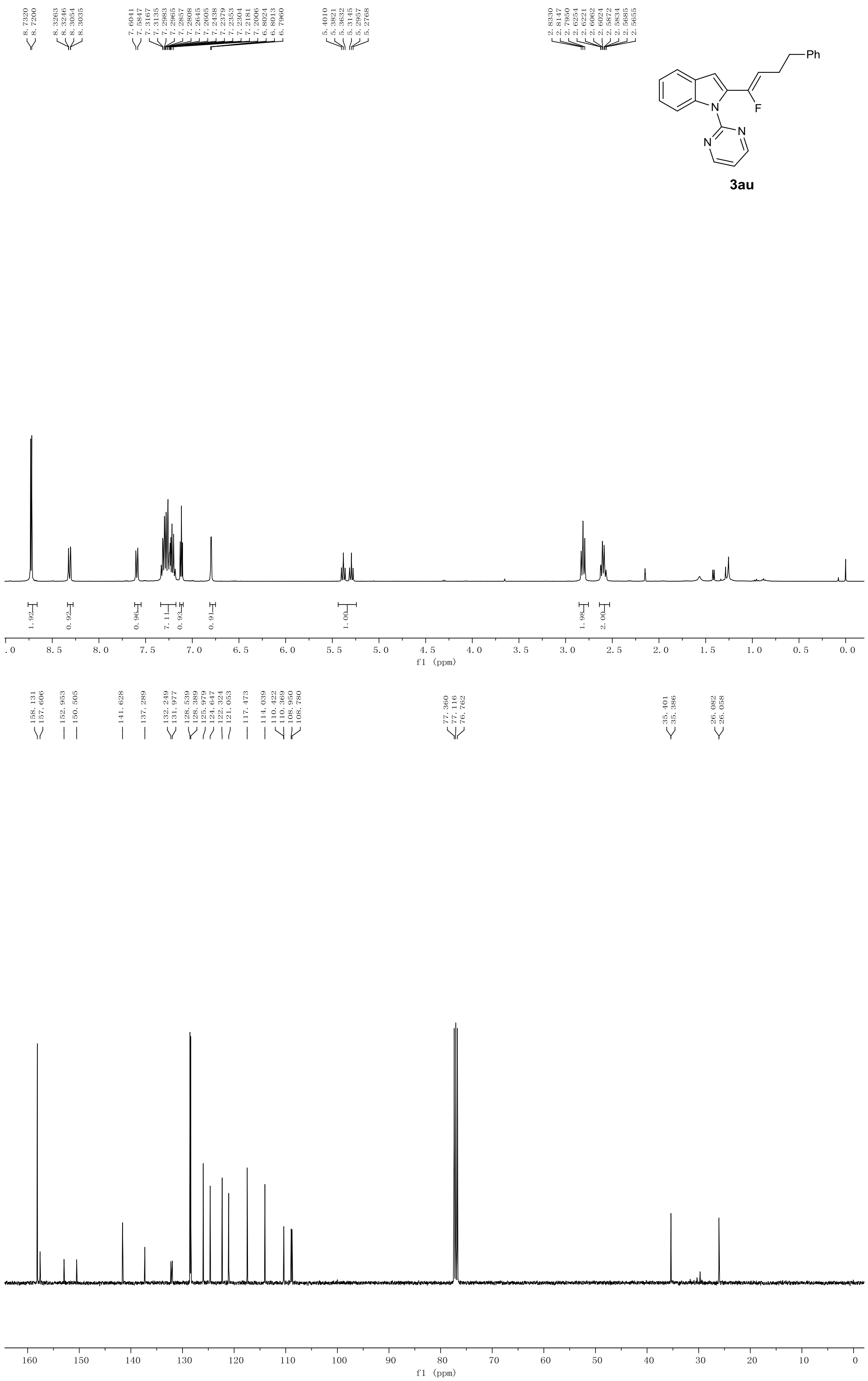

Supplementary Figure 21. ^{1}H and ^{13}C NMR spectra for product 3au

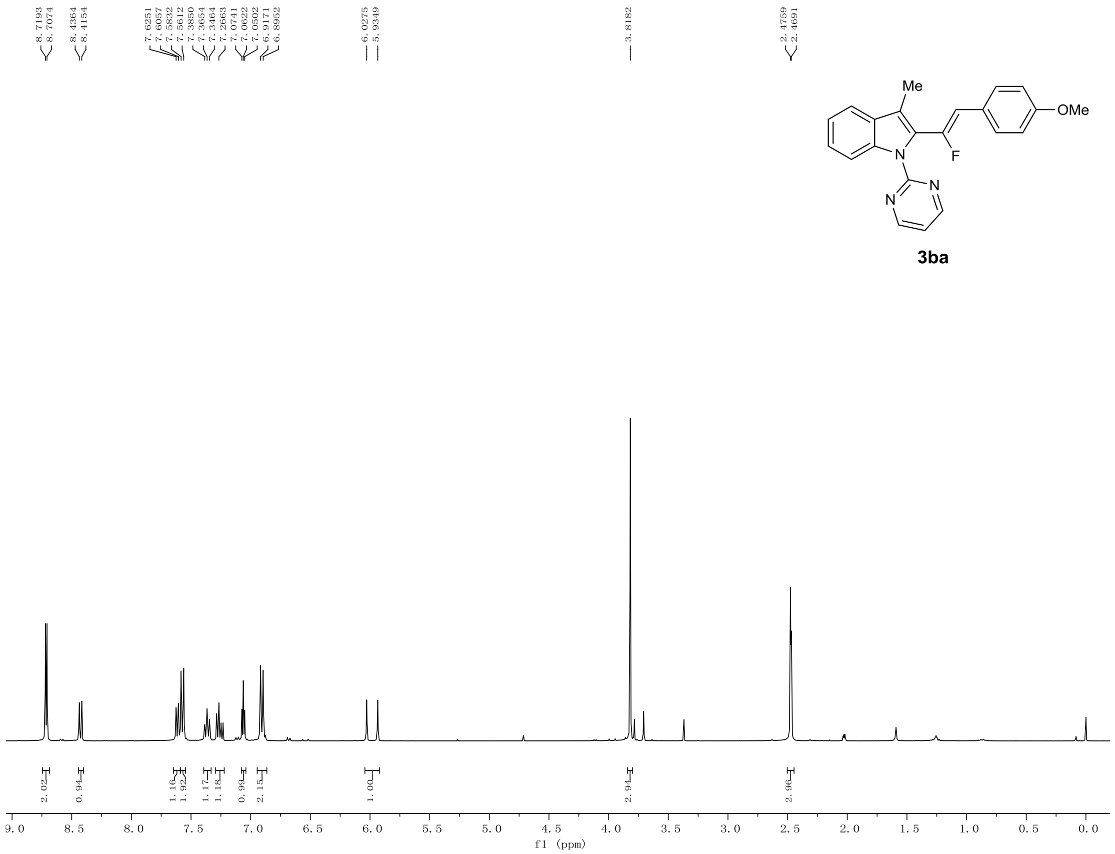

3ba

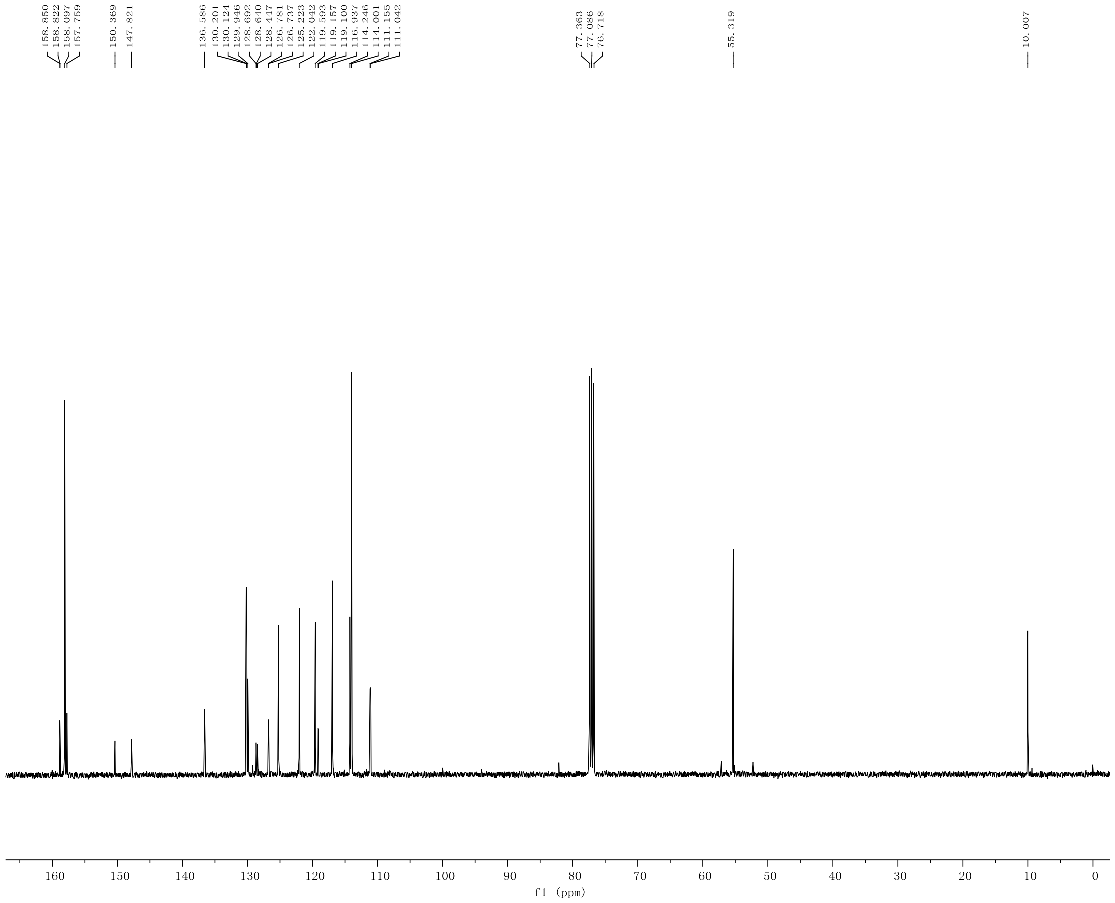

Supplementary Figure 22. ^{1}H and ^{13}C NMR spectra for product 3ba

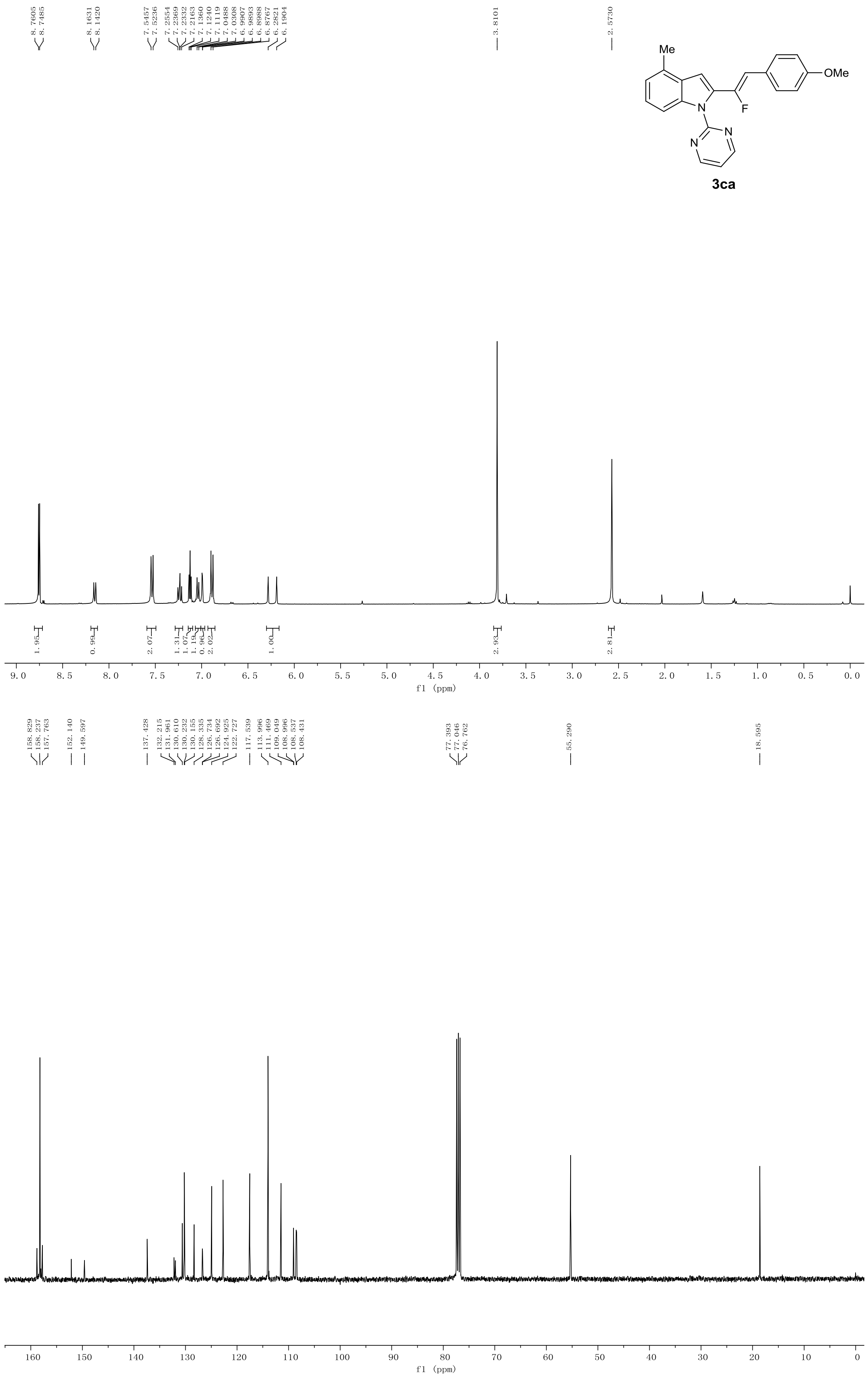

Supplementary Figure 23. ^{1}H and ^{13}C NMR spectra for product 3ca

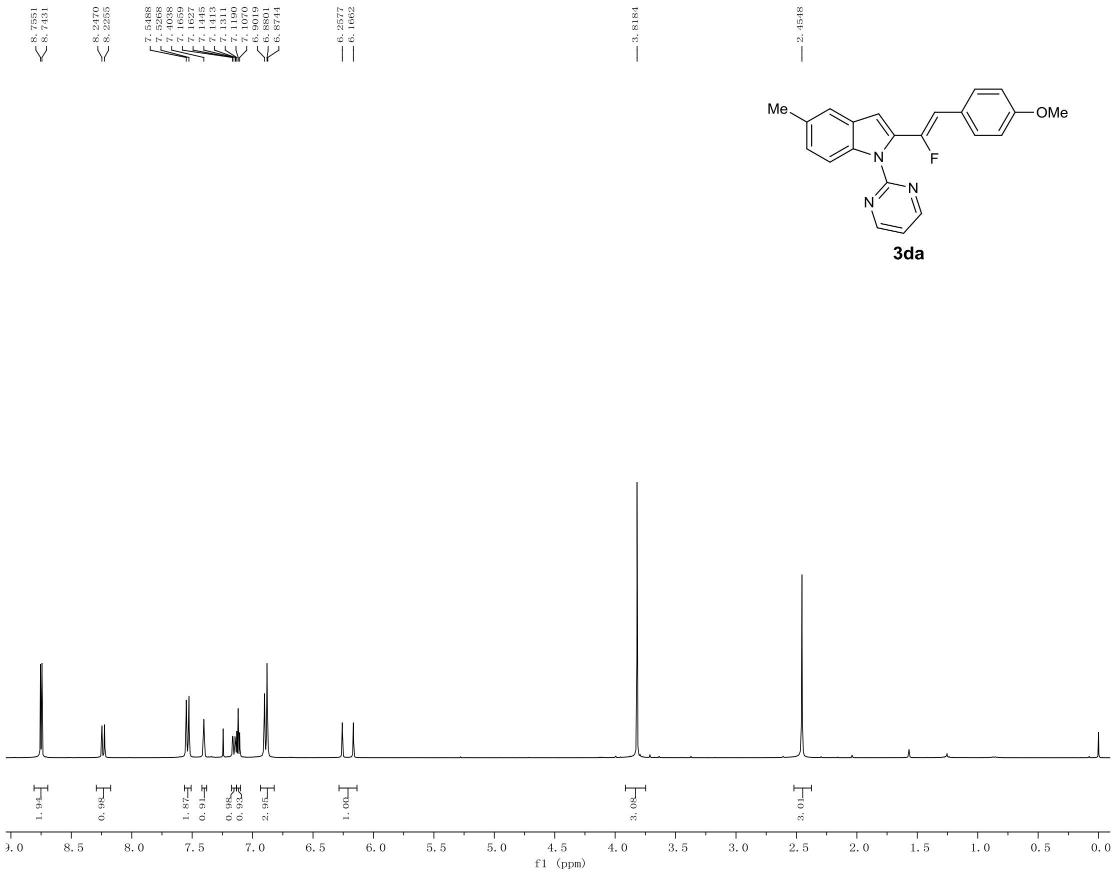

3da

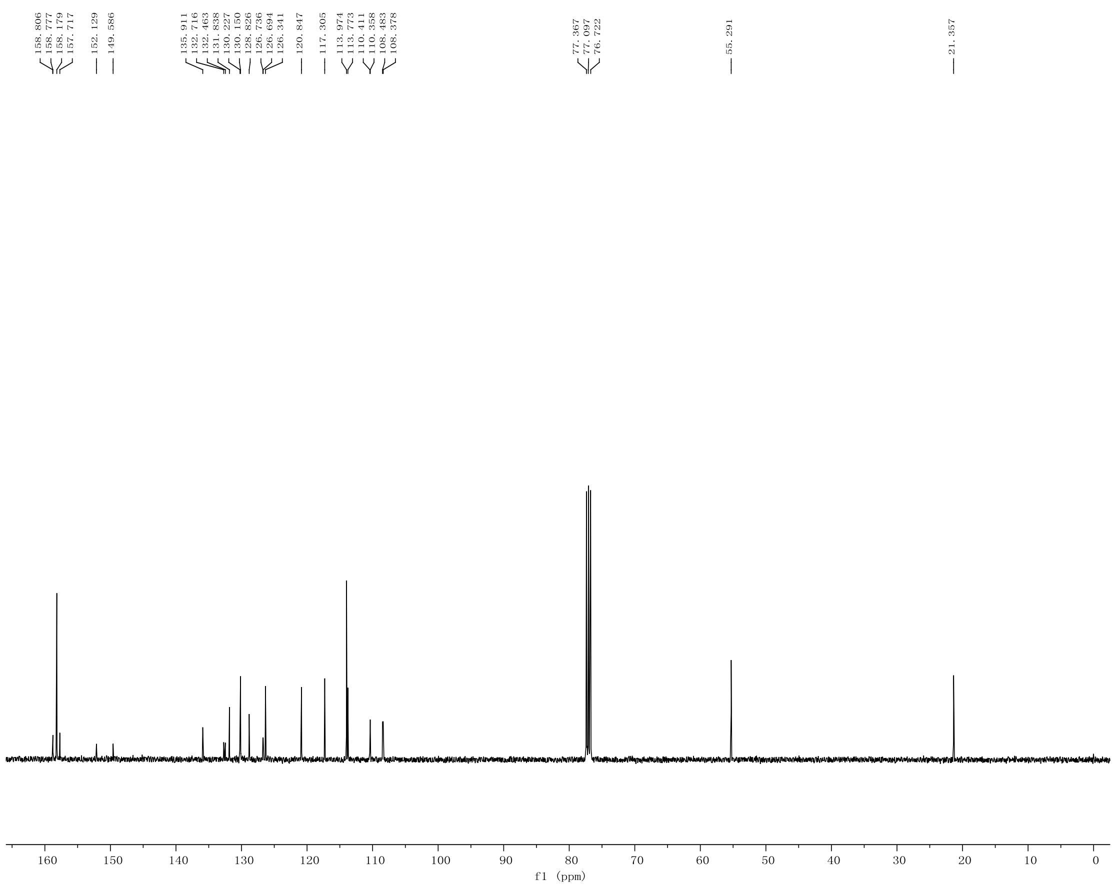

Supplementary Figure 24. ^{1}H and ^{13}C NMR spectra for product 3da

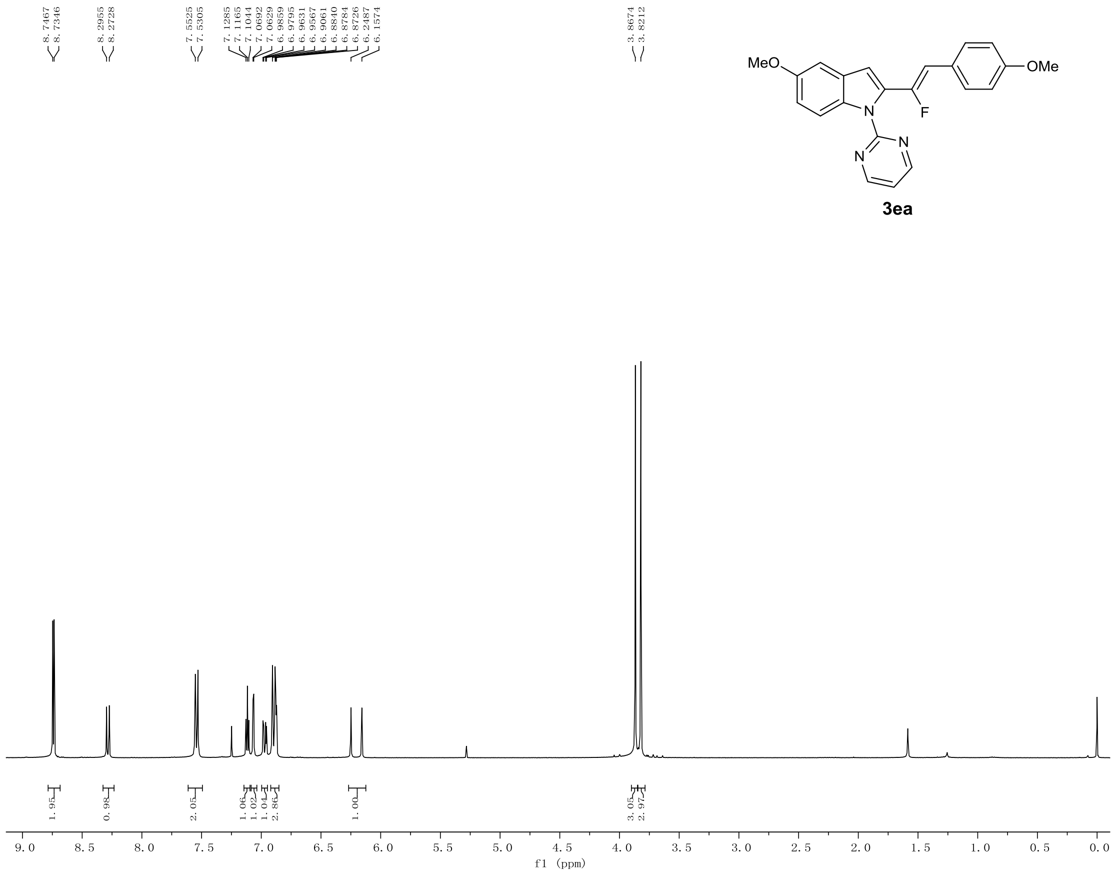

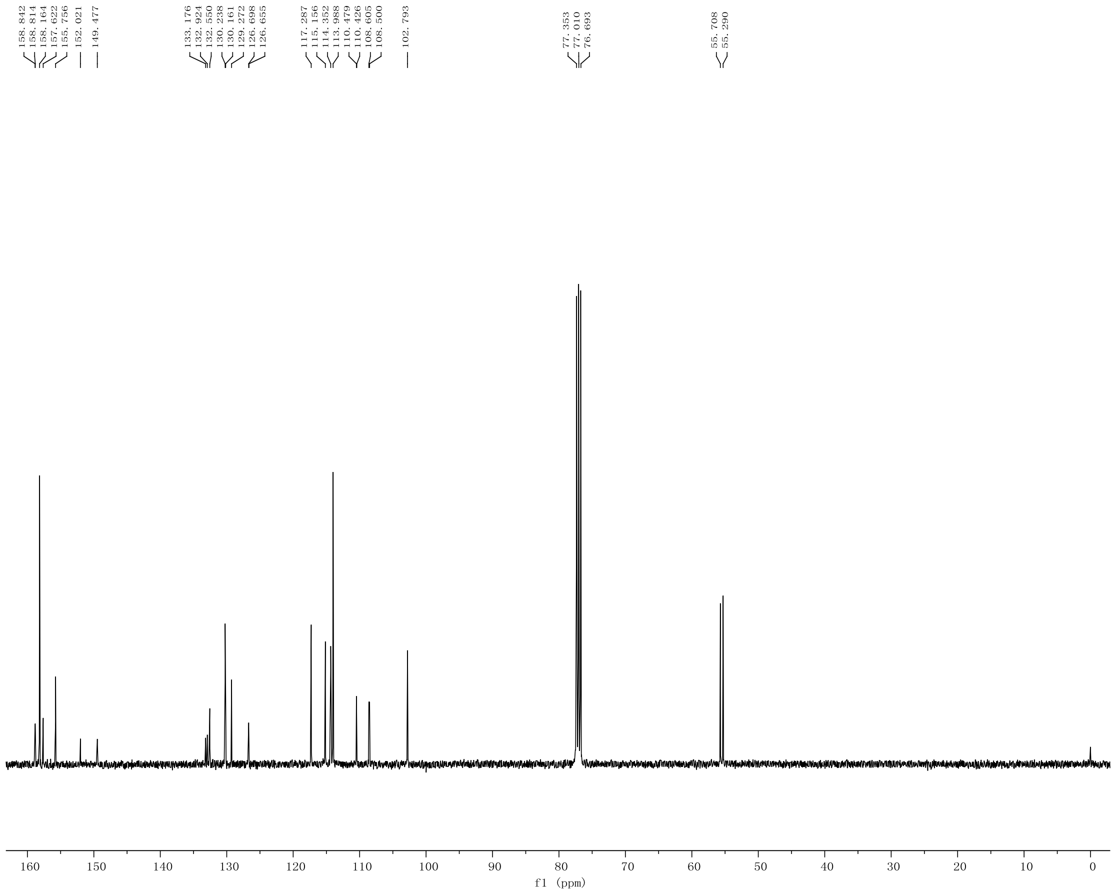

Supplementary Figure 25. ^1H and ^{13}C NMR spectra for product 3ea

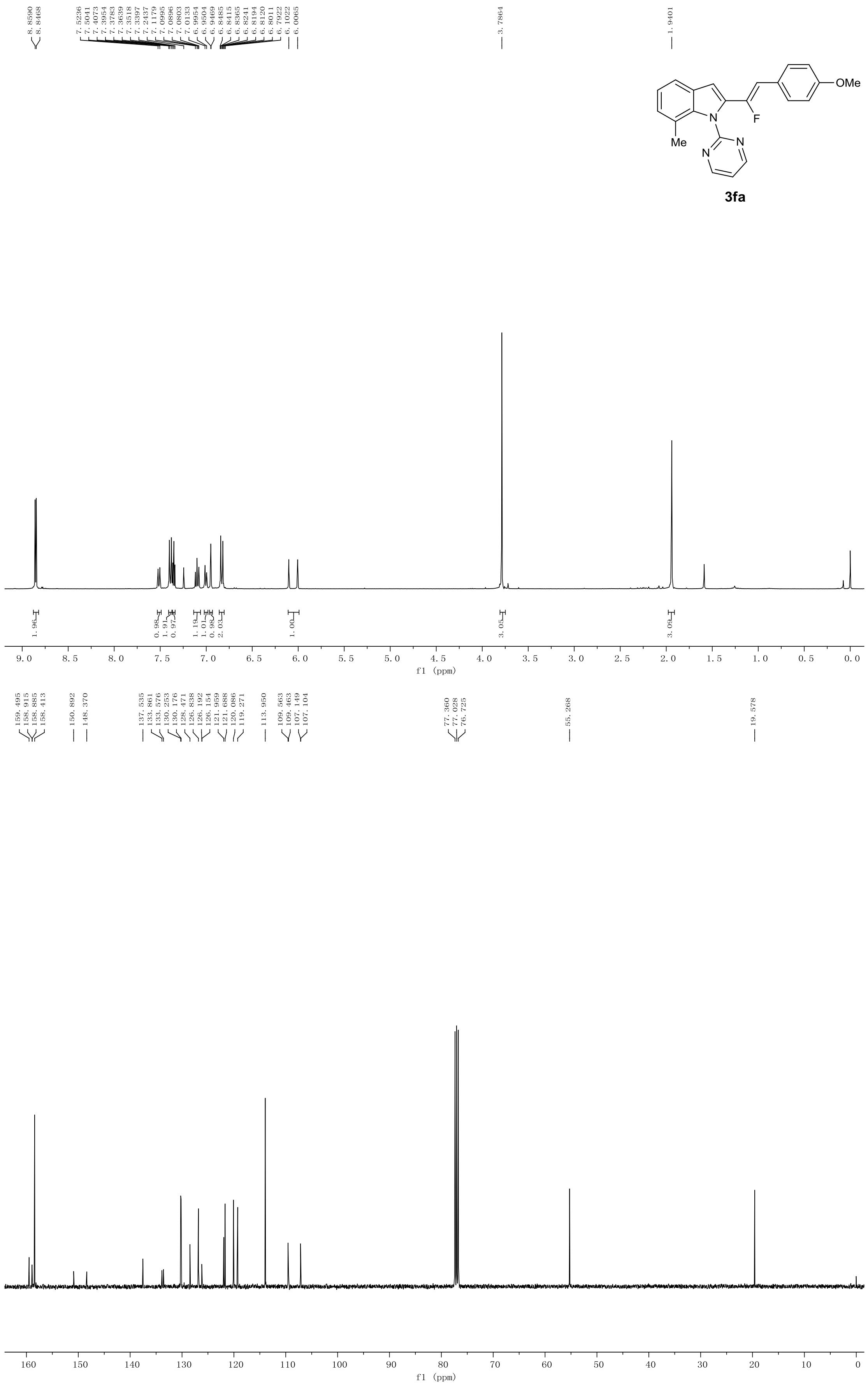

Supplementary Figure 26. ^{1}H and ^{13}C NMR spectra for product 3fa

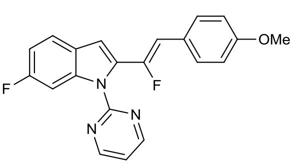

3ga

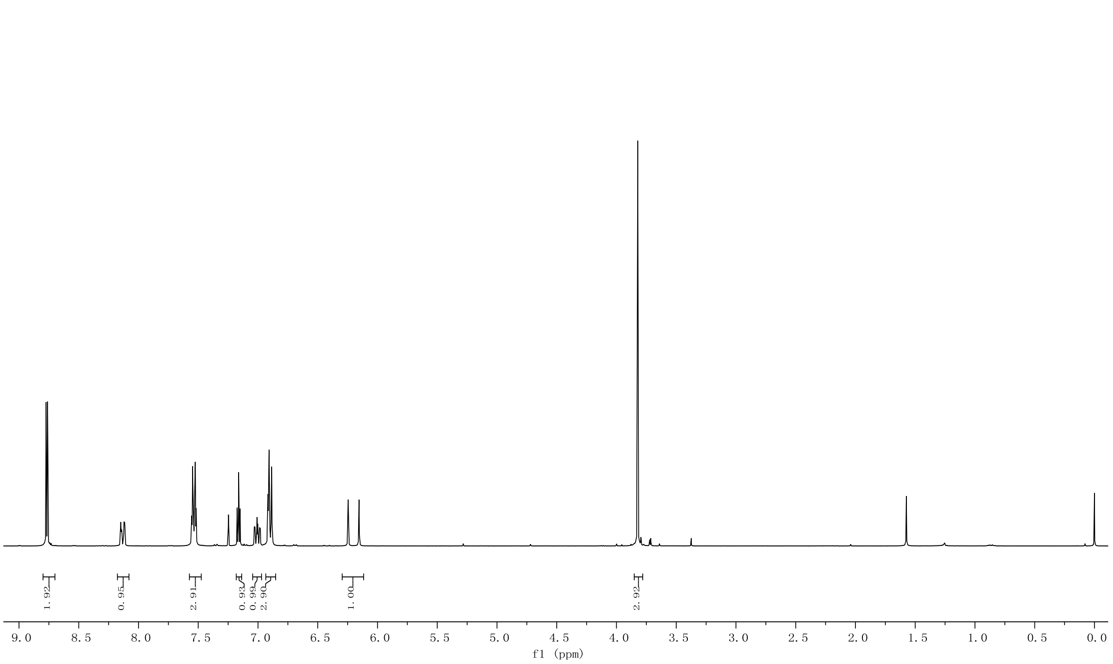

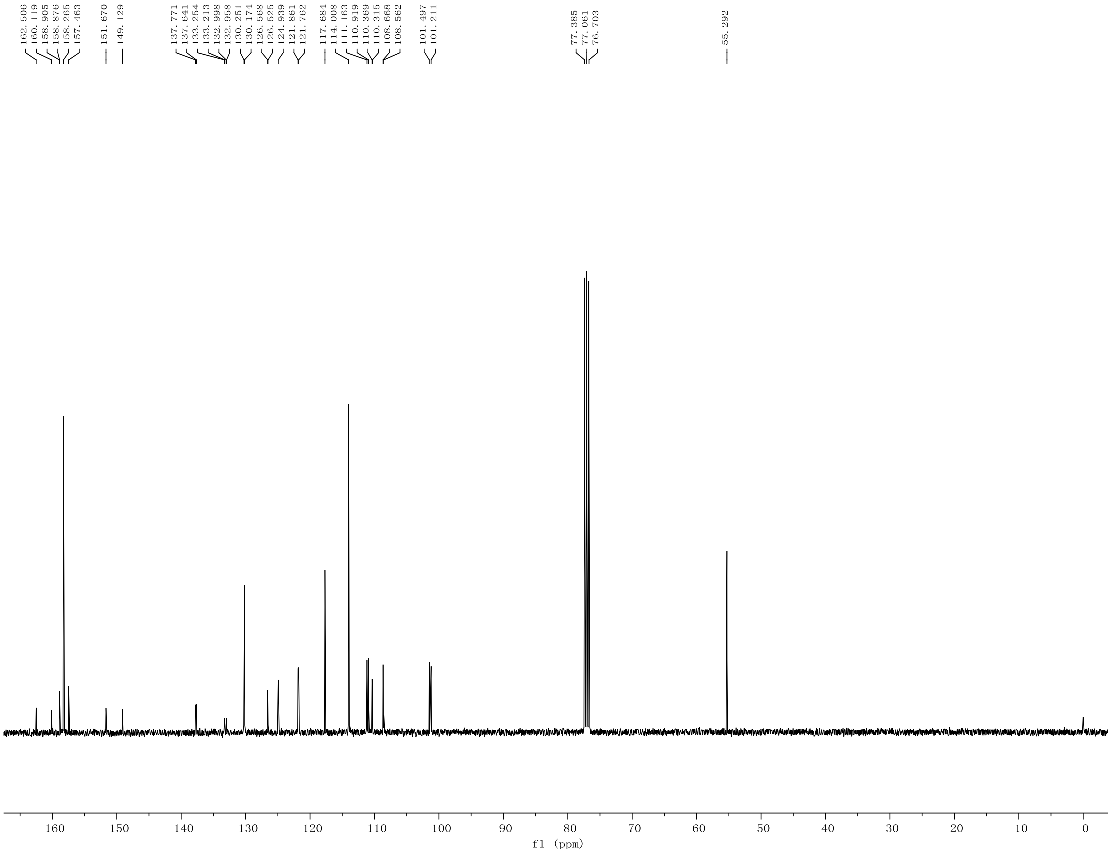

Supplementary Figure 27. ^{1}H and ^{13}C NMR spectra for product 3ga

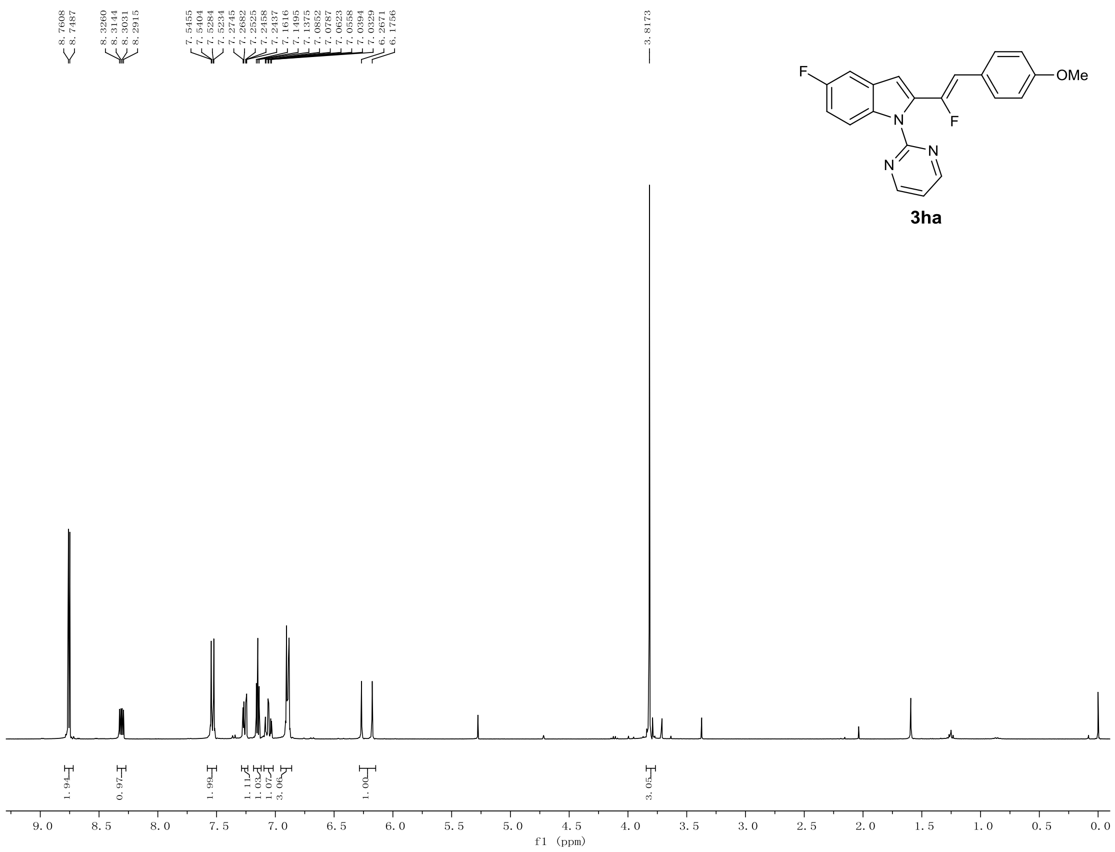

3ha

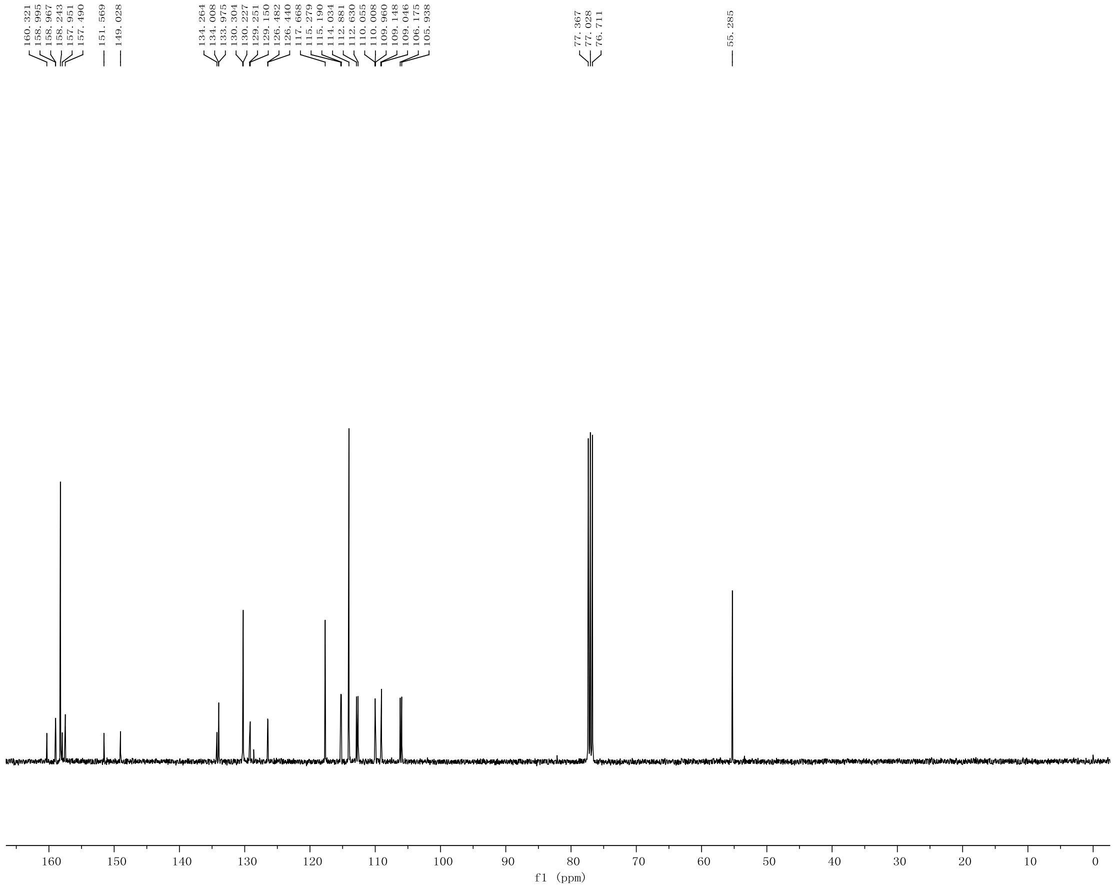

Supplementary Figure 28. ^{1}H and ^{13}C NMR spectra for product 3ha

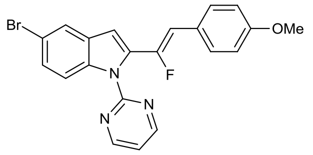

3ia

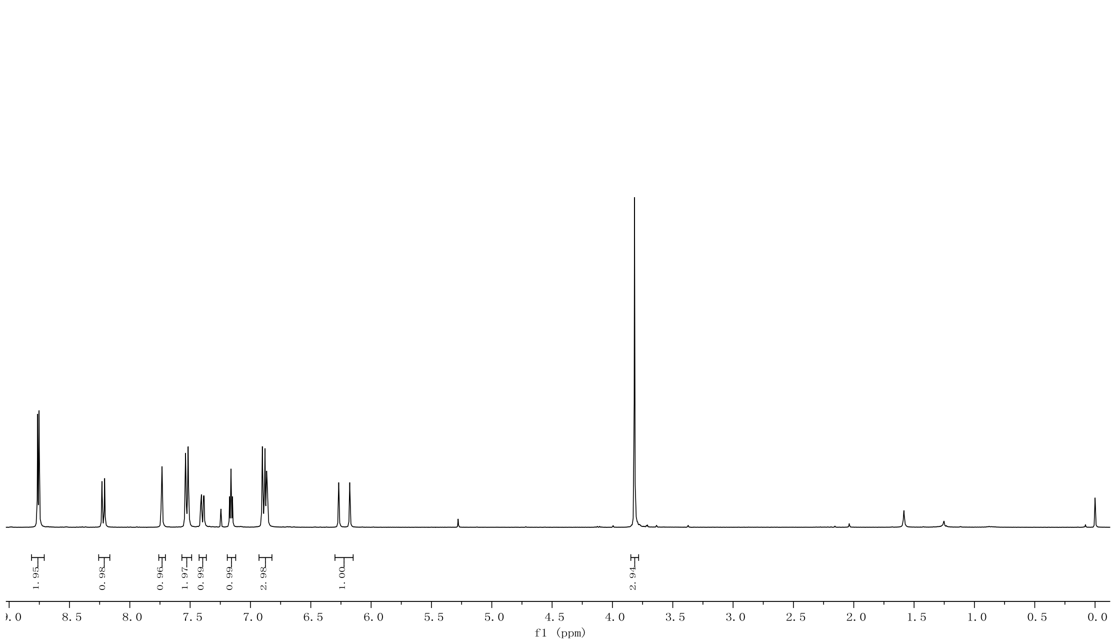

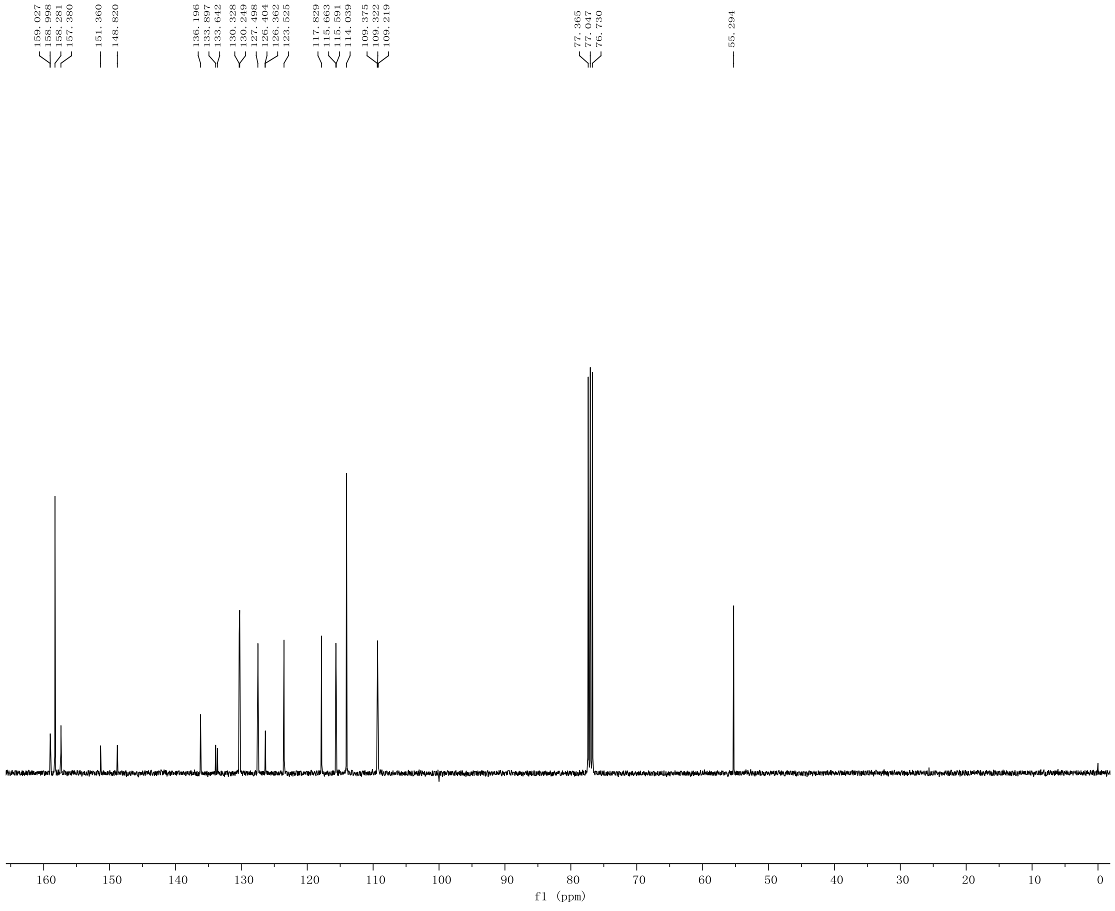

Supplementary Figure 29. ^{1}H and ^{13}C NMR spectra for product 3ia

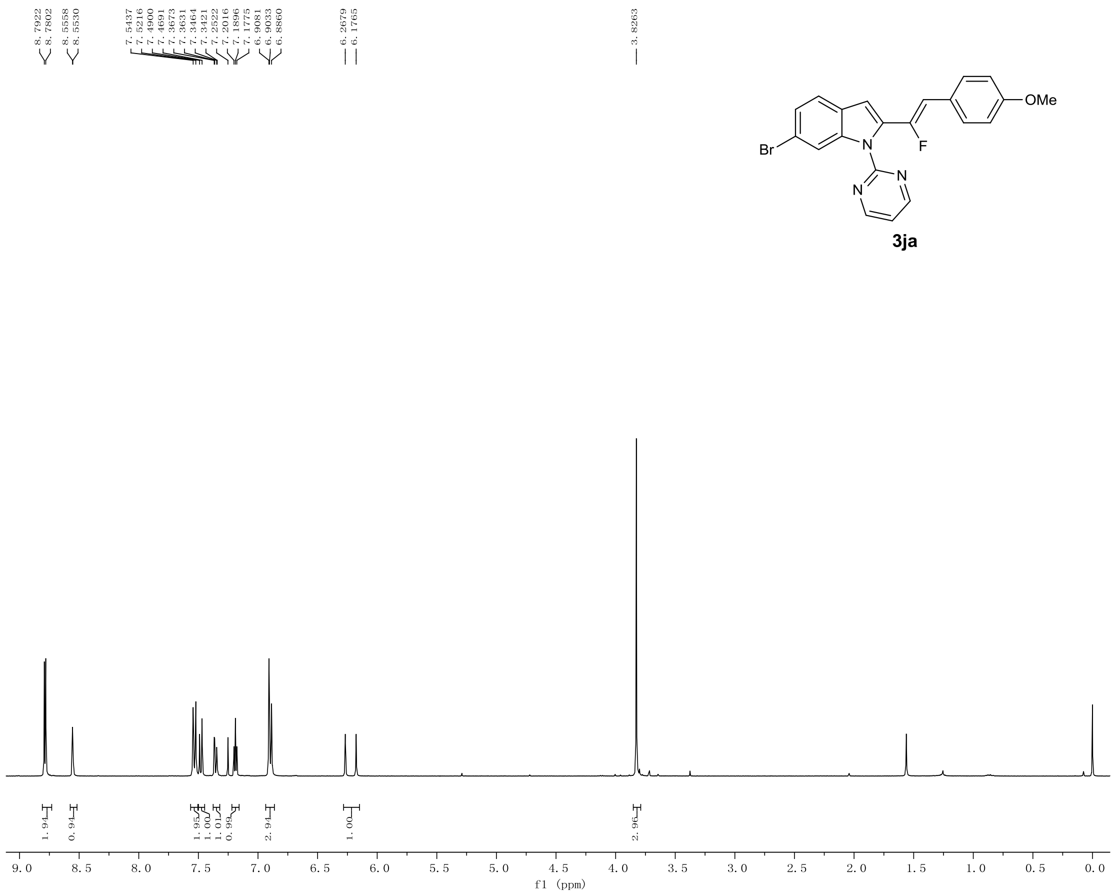

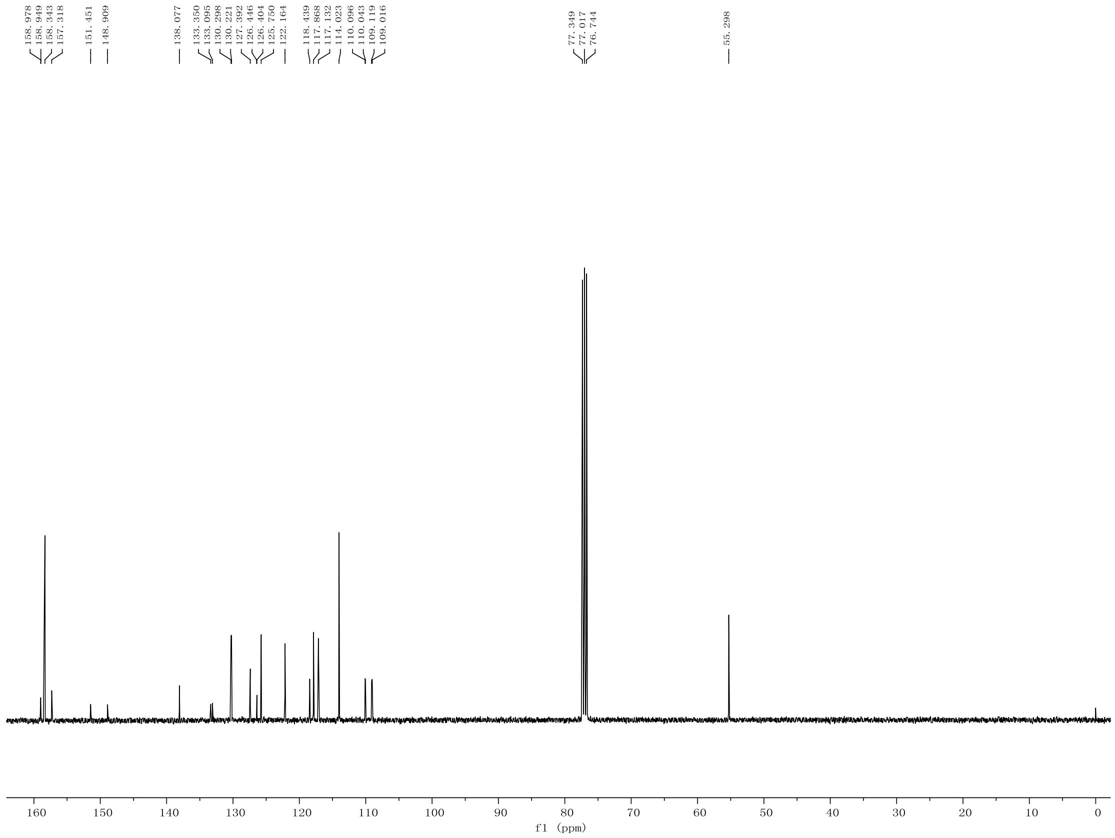

Supplementary Figure 30. ^{1}H and ^{13}C NMR spectra for product 3ja

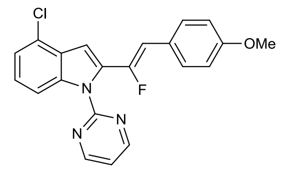

3ka

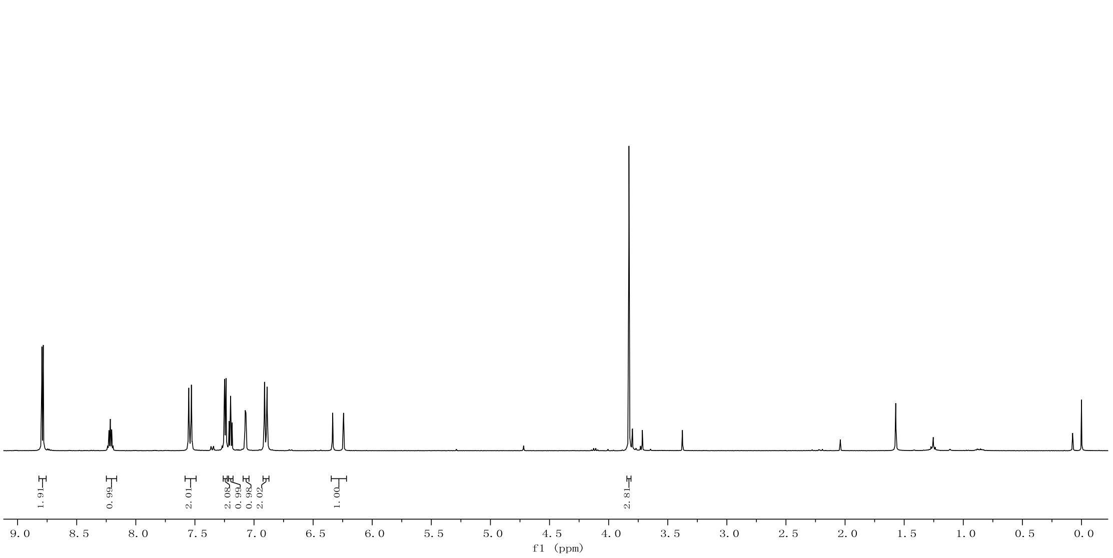

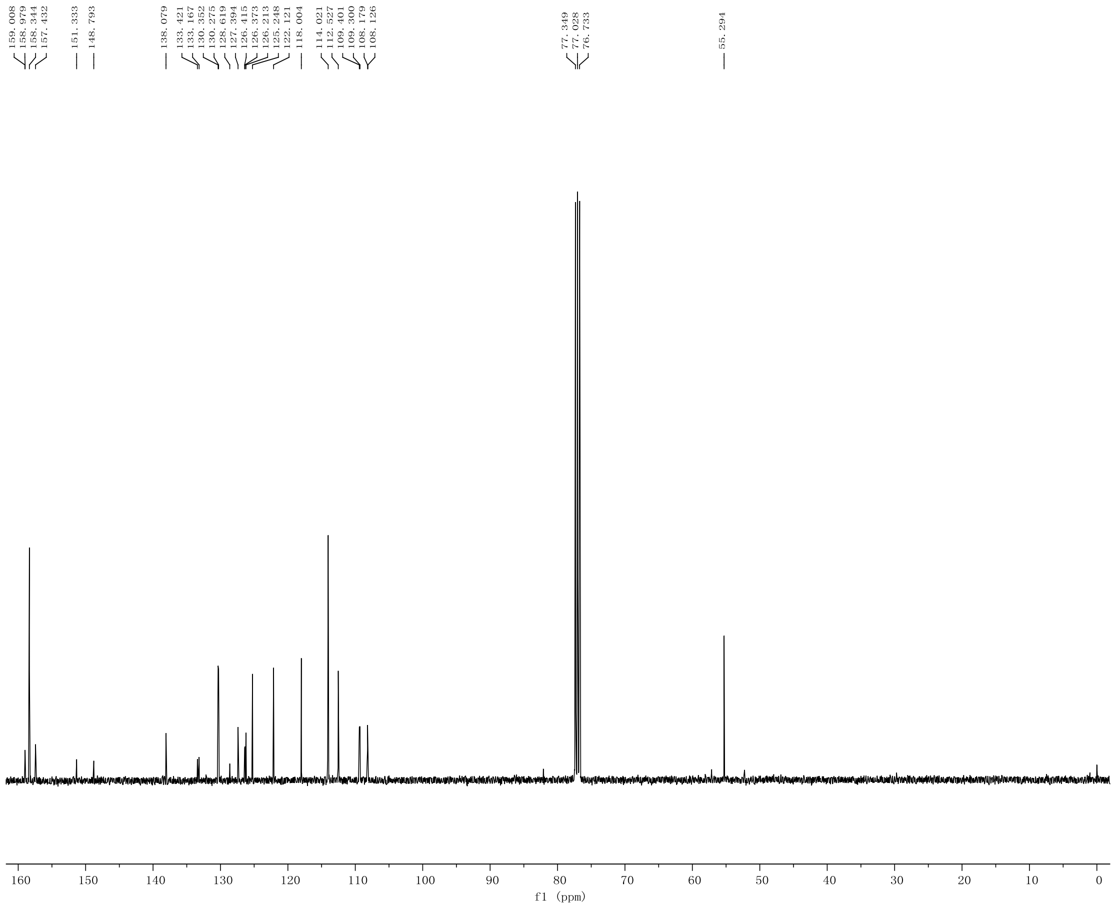

Supplementary Figure 31. ^{1}H and ^{13}C NMR spectra for product 3ka

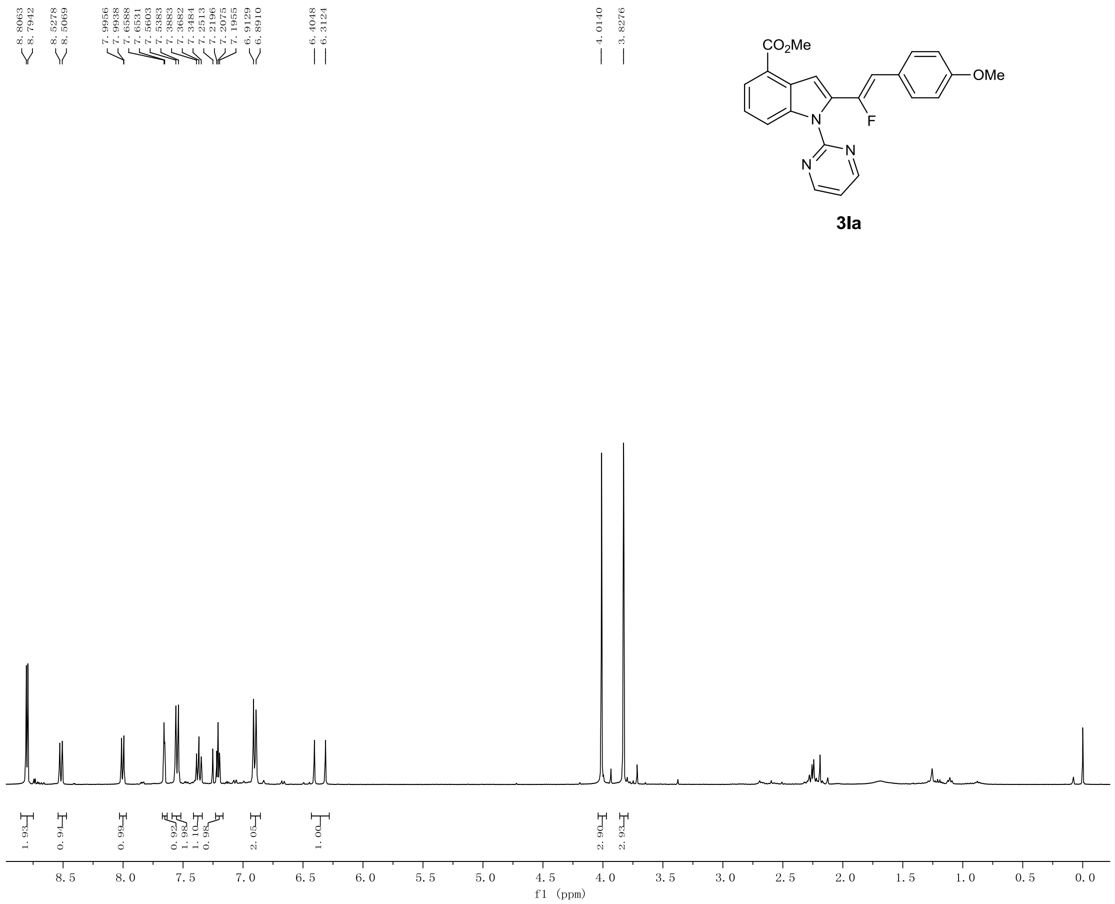

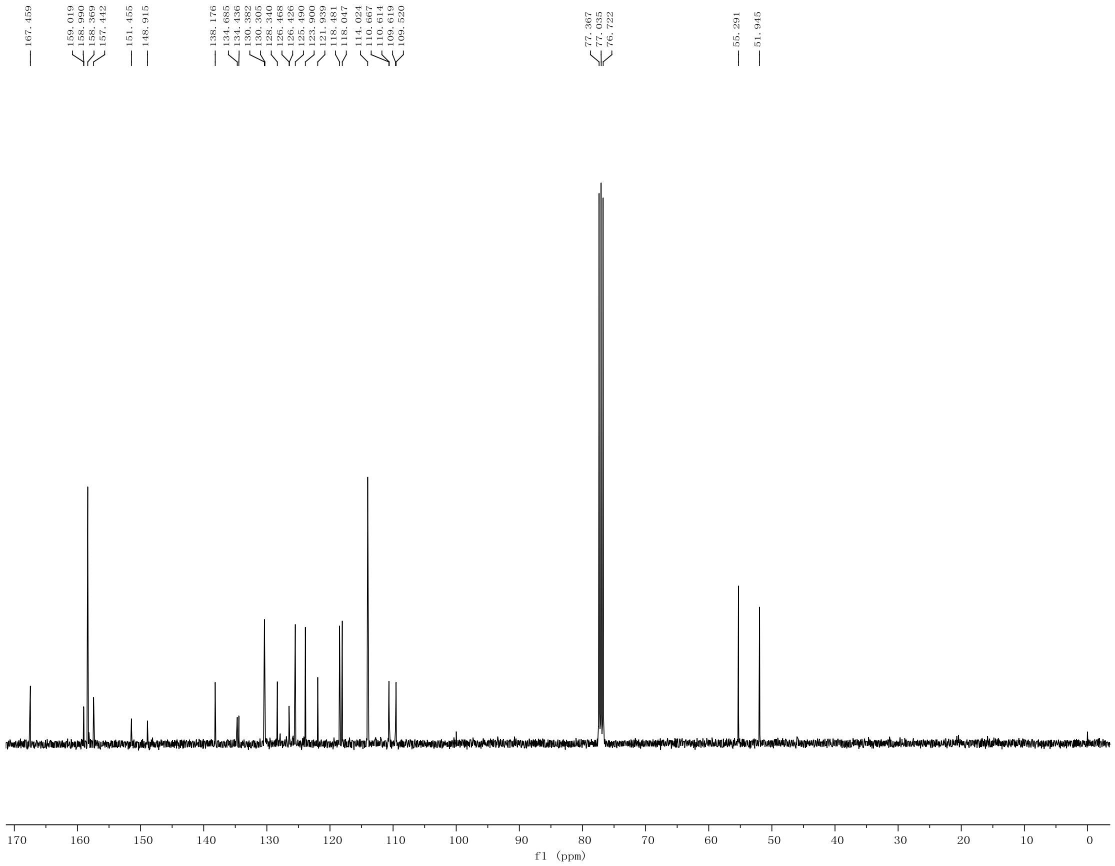

Supplementary Figure 32. ^{1}H and ^{13}C NMR spectra for product 3la

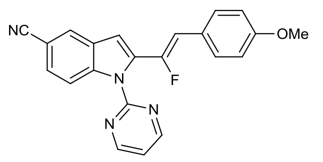

3ma

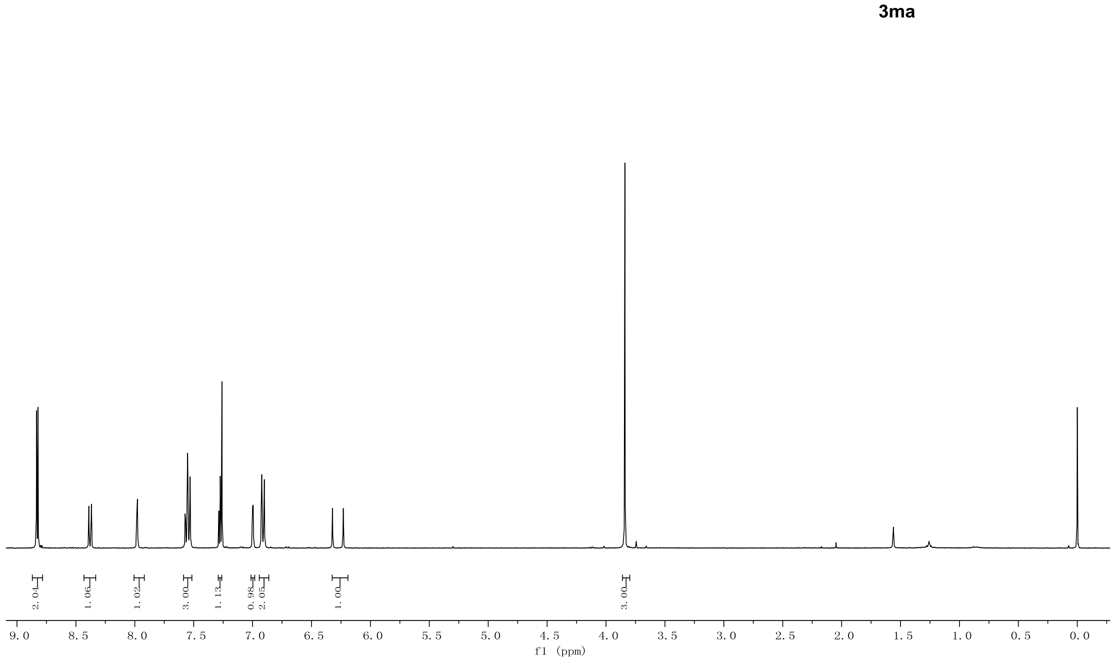

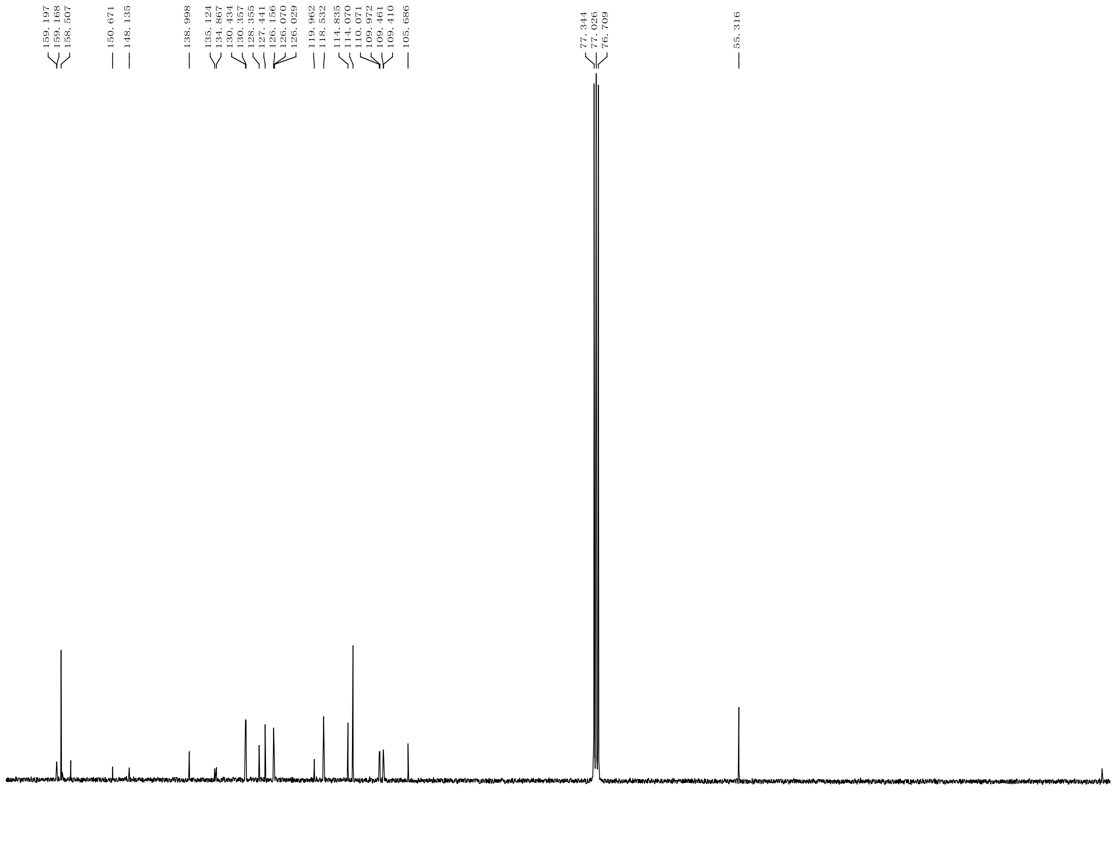

Supplementary Figure 33. ^{1}H and ^{13}C NMR spectra for product 3ma

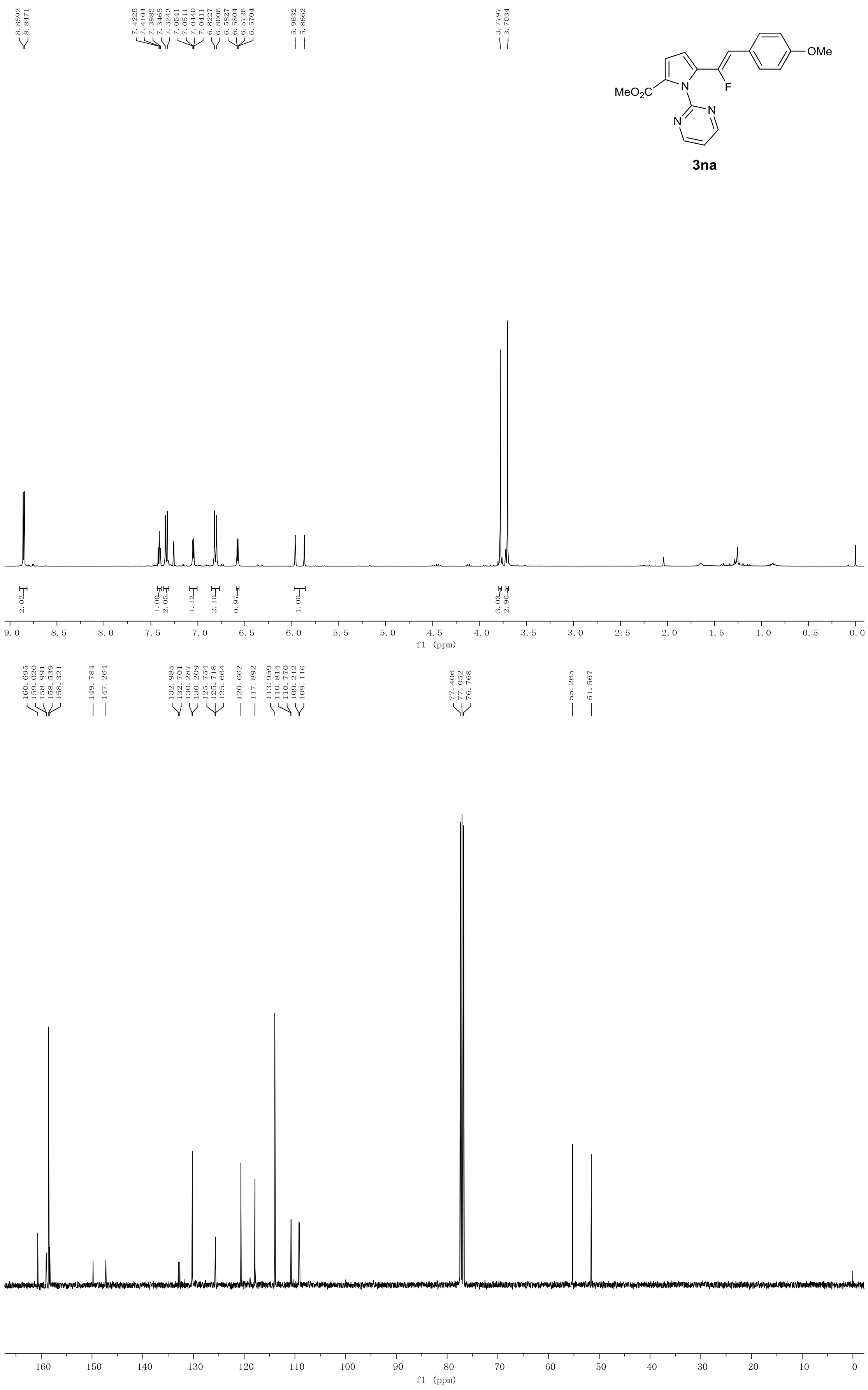

Supplementary Figure 34. ^{1}H and ^{13}C NMR spectra for product 3na

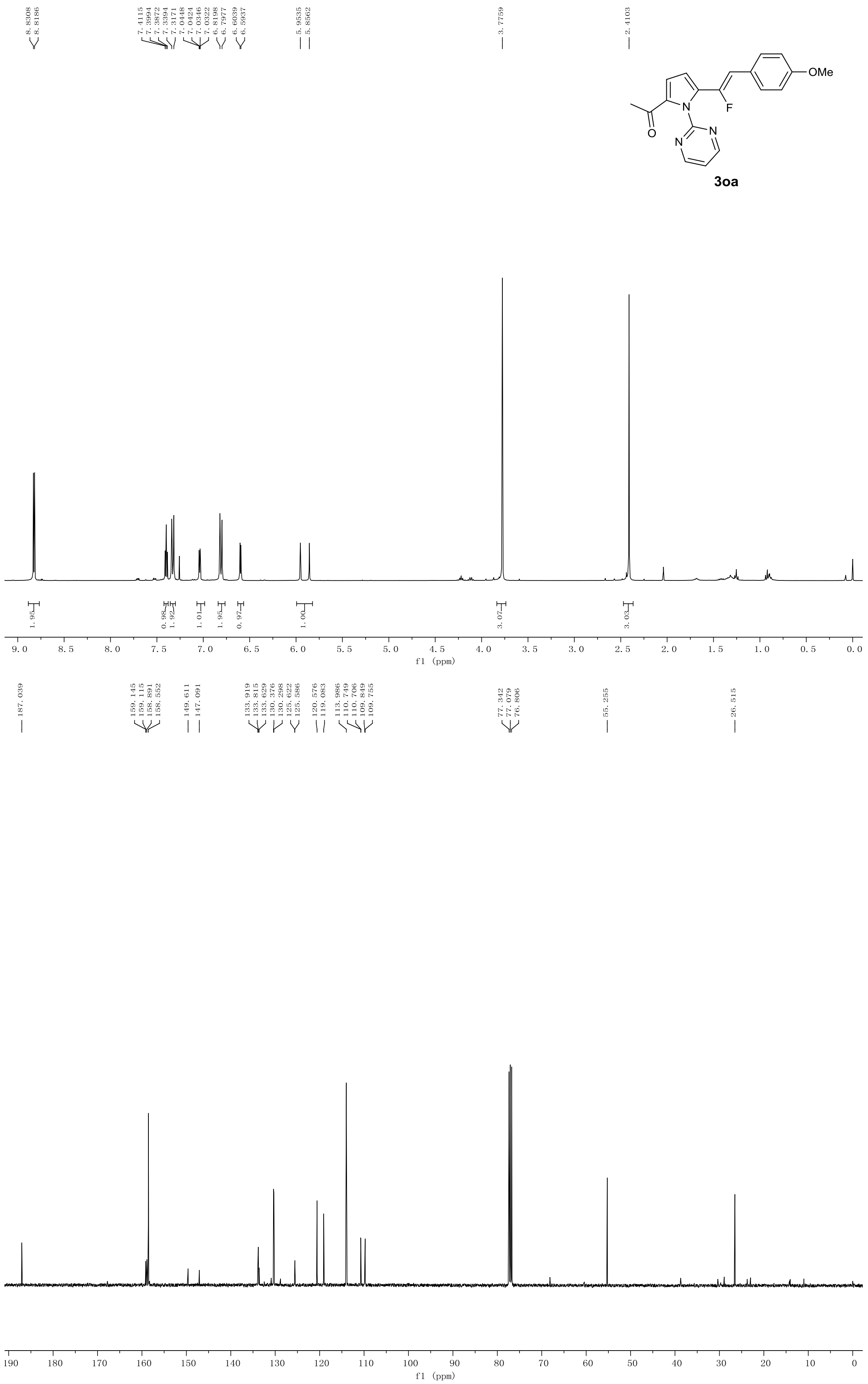

Supplementary Figure 35. ^{1}H and ^{13}C NMR spectra for product 3oa

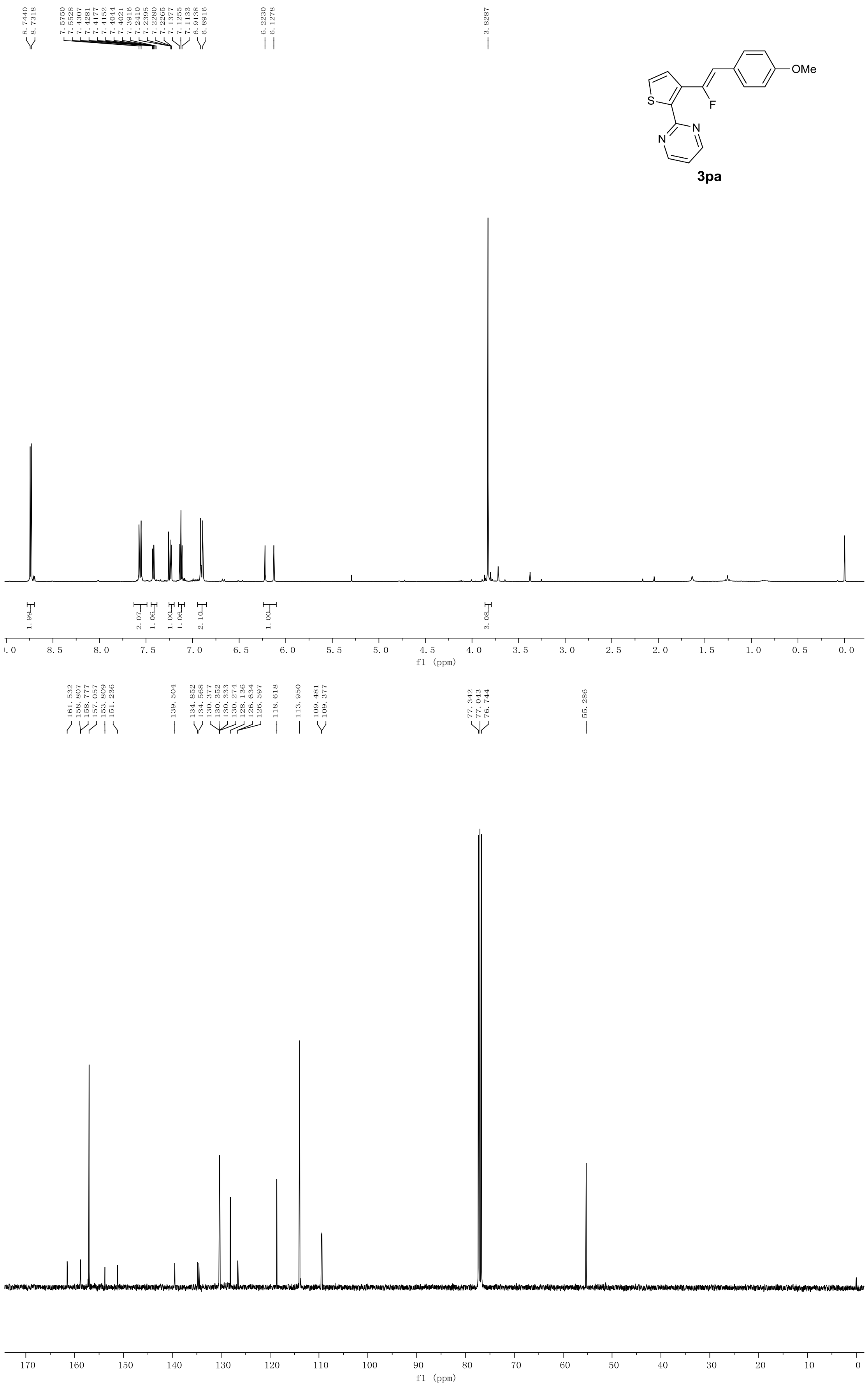

Supplementary Figure 36. ^{1}H and ^{13}C NMR spectra for product 3pa

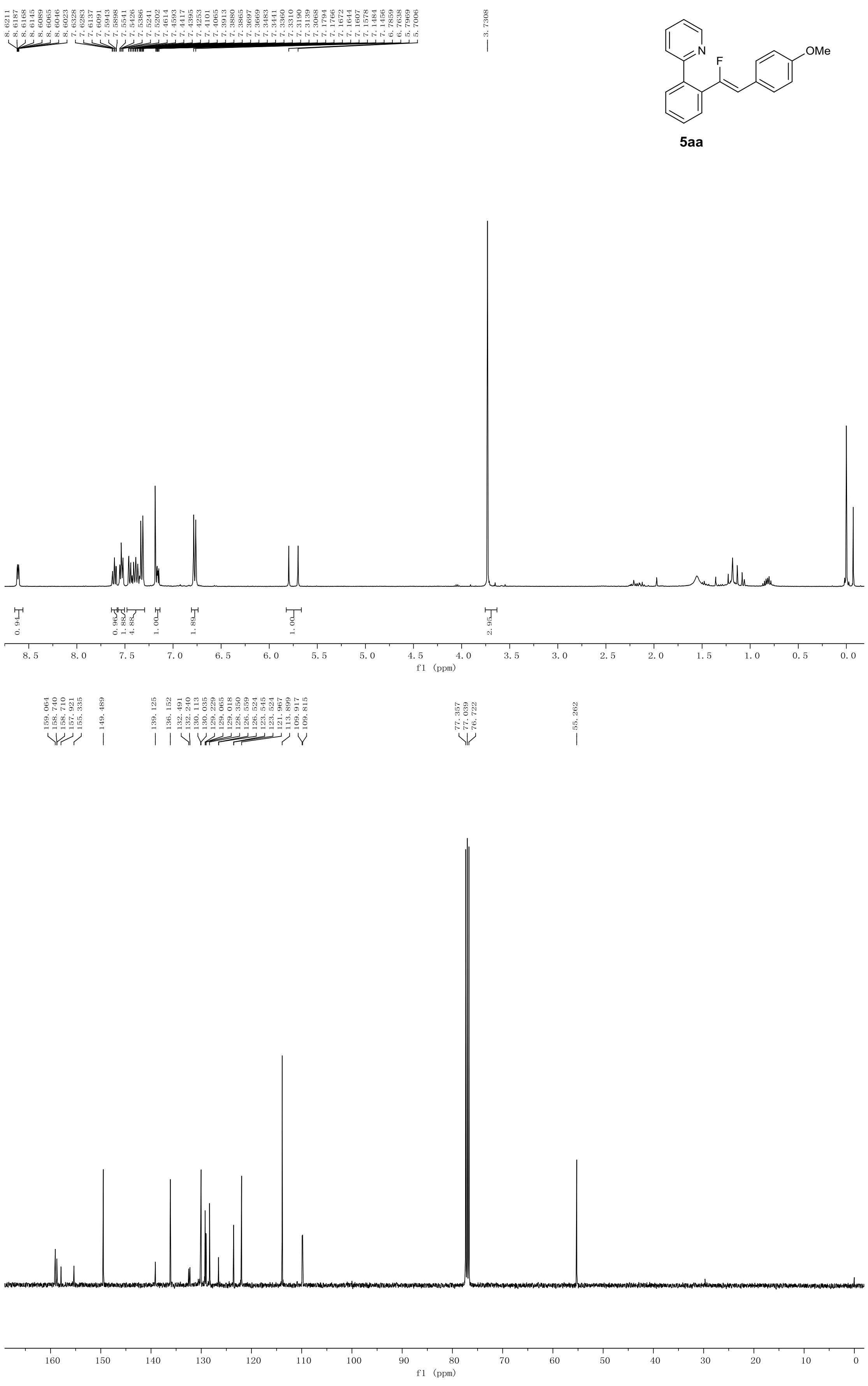

Supplementary Figure 37. ^{1}H and ^{13}C NMR spectra for product 5aa

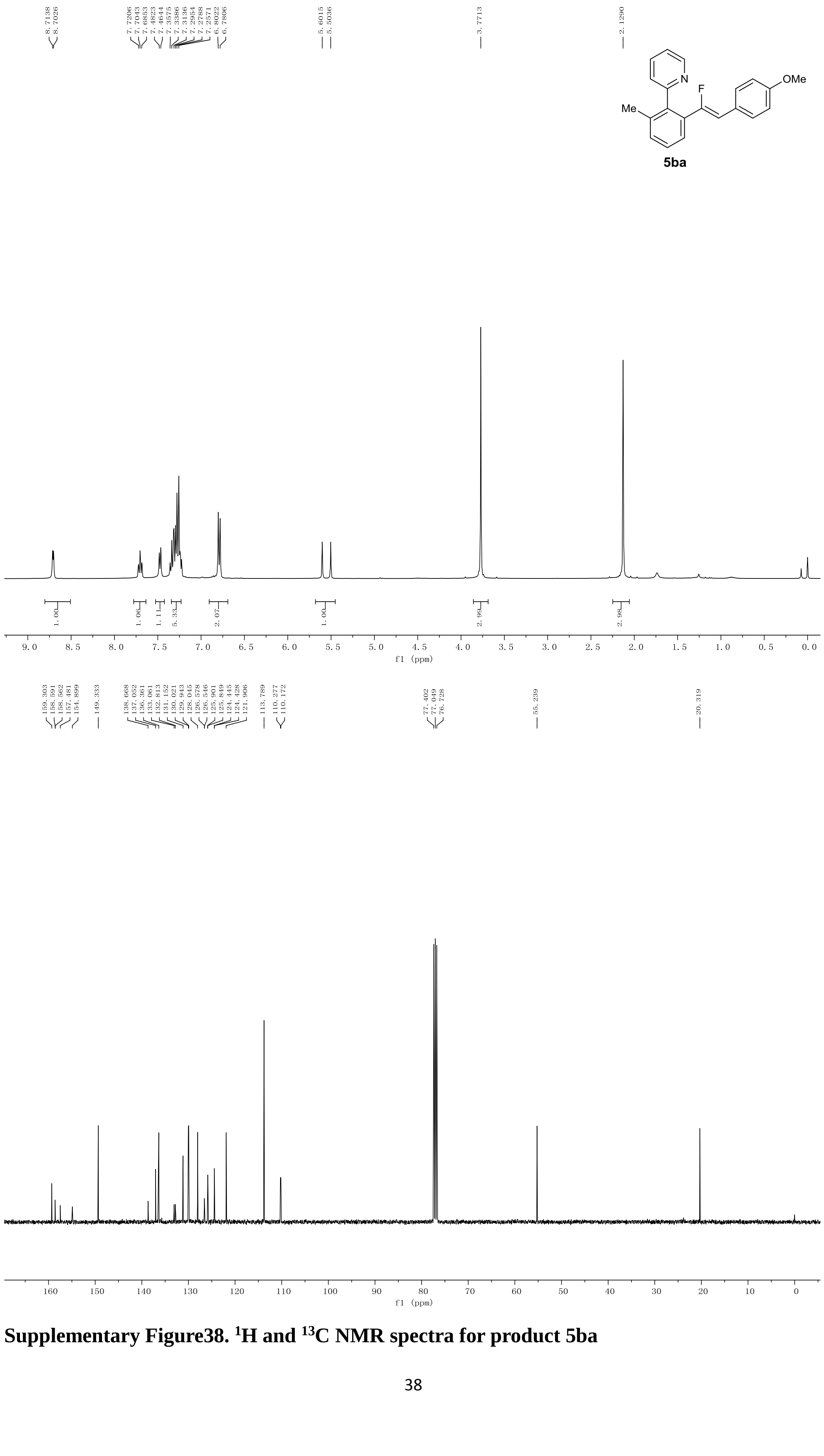

Supplementary Figure38. ^{1}H and ^{13}C NMR spectra for product 5ba

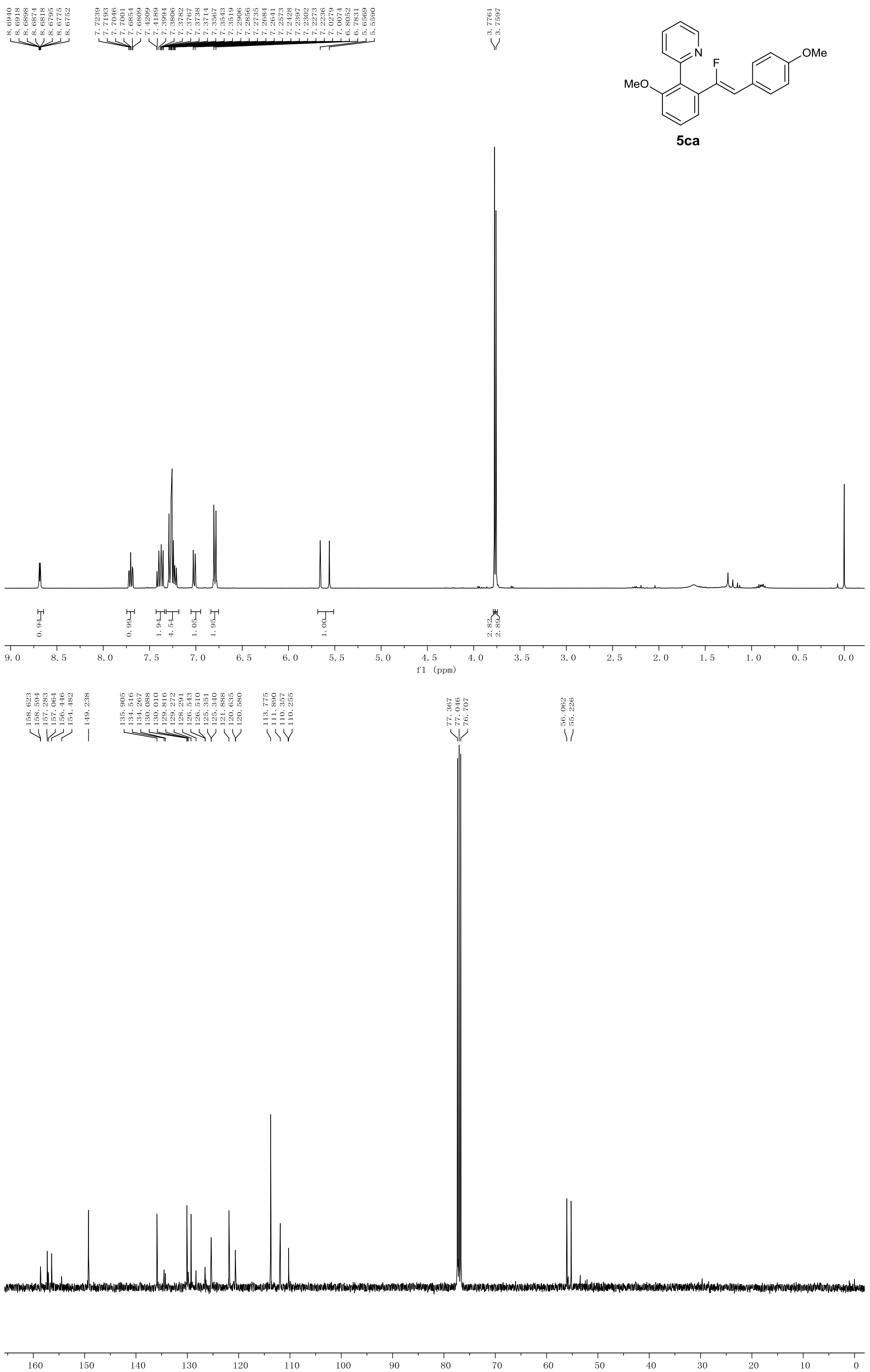

Supplementary Figure 39. ^{1}H and ^{13}C NMR spectra for product 5ca

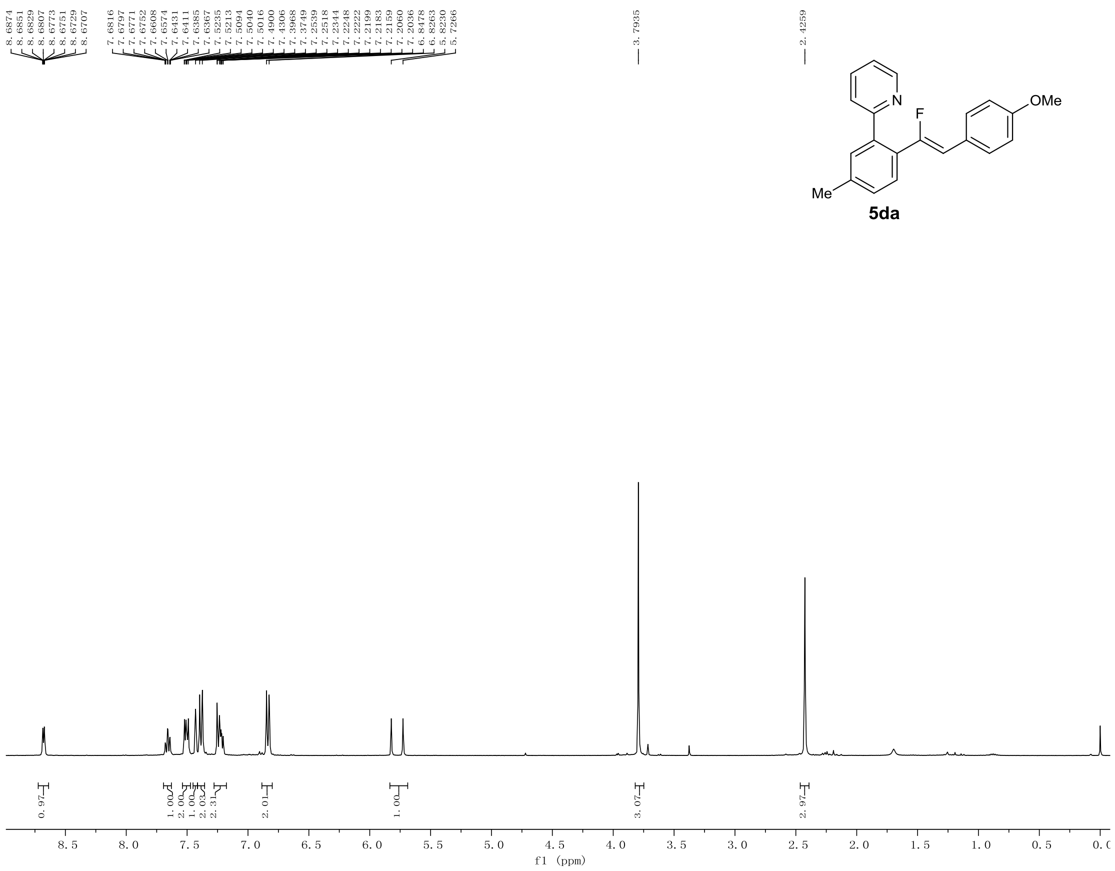

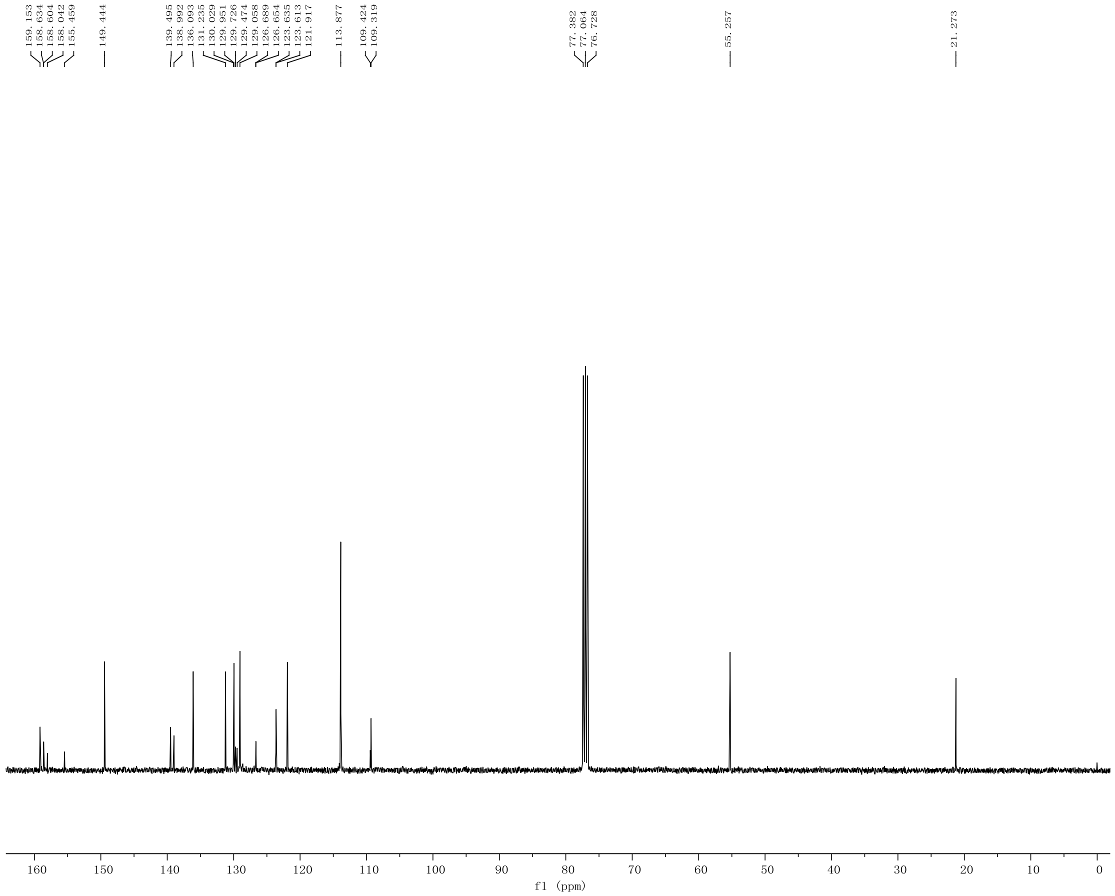

Supplementary Figure 40. ^{1}H and ^{13}C NMR spectra for product 5da

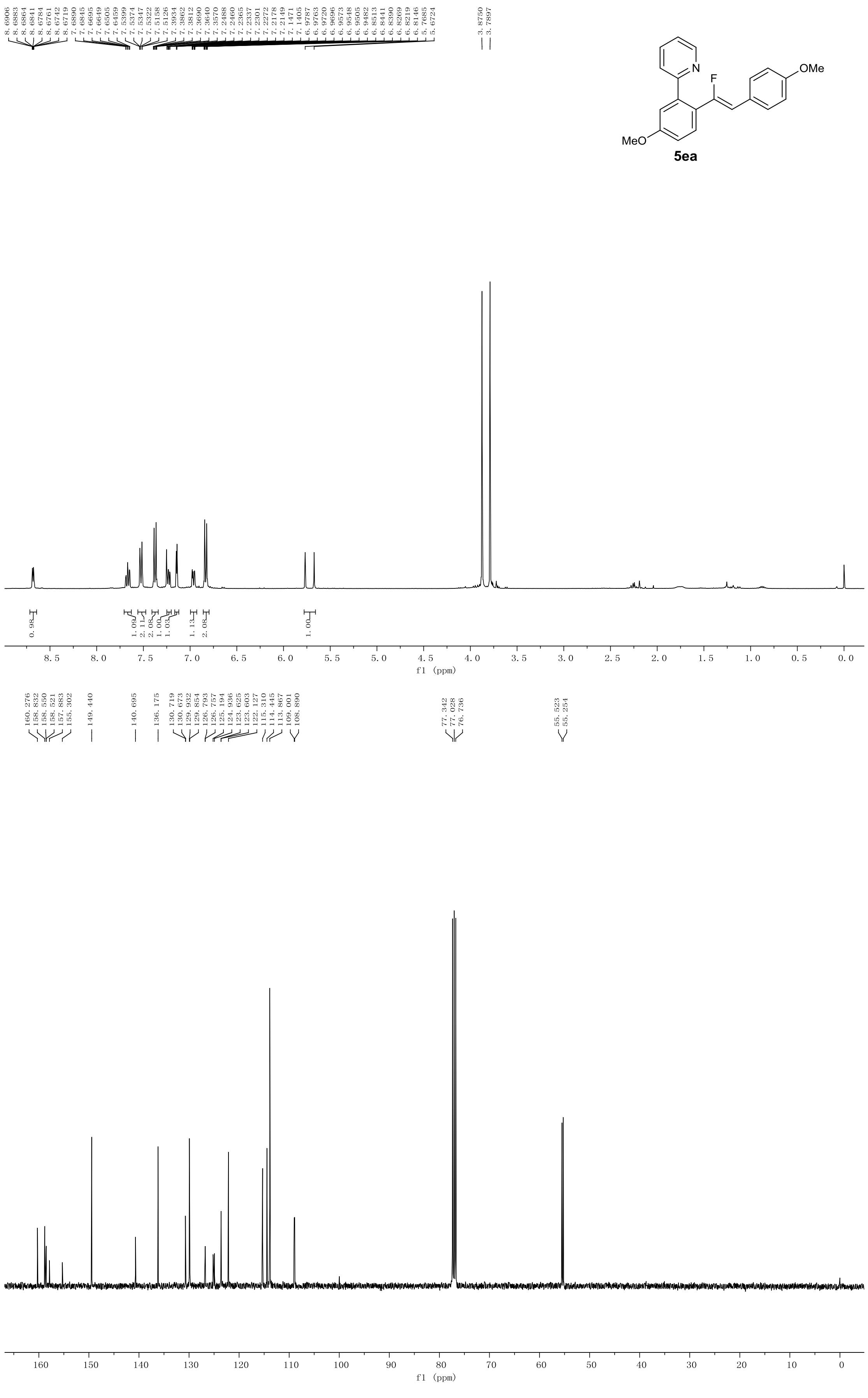

Supplementary Figure 41. ^{1}H and ^{13}C NMR spectra for product 5ea

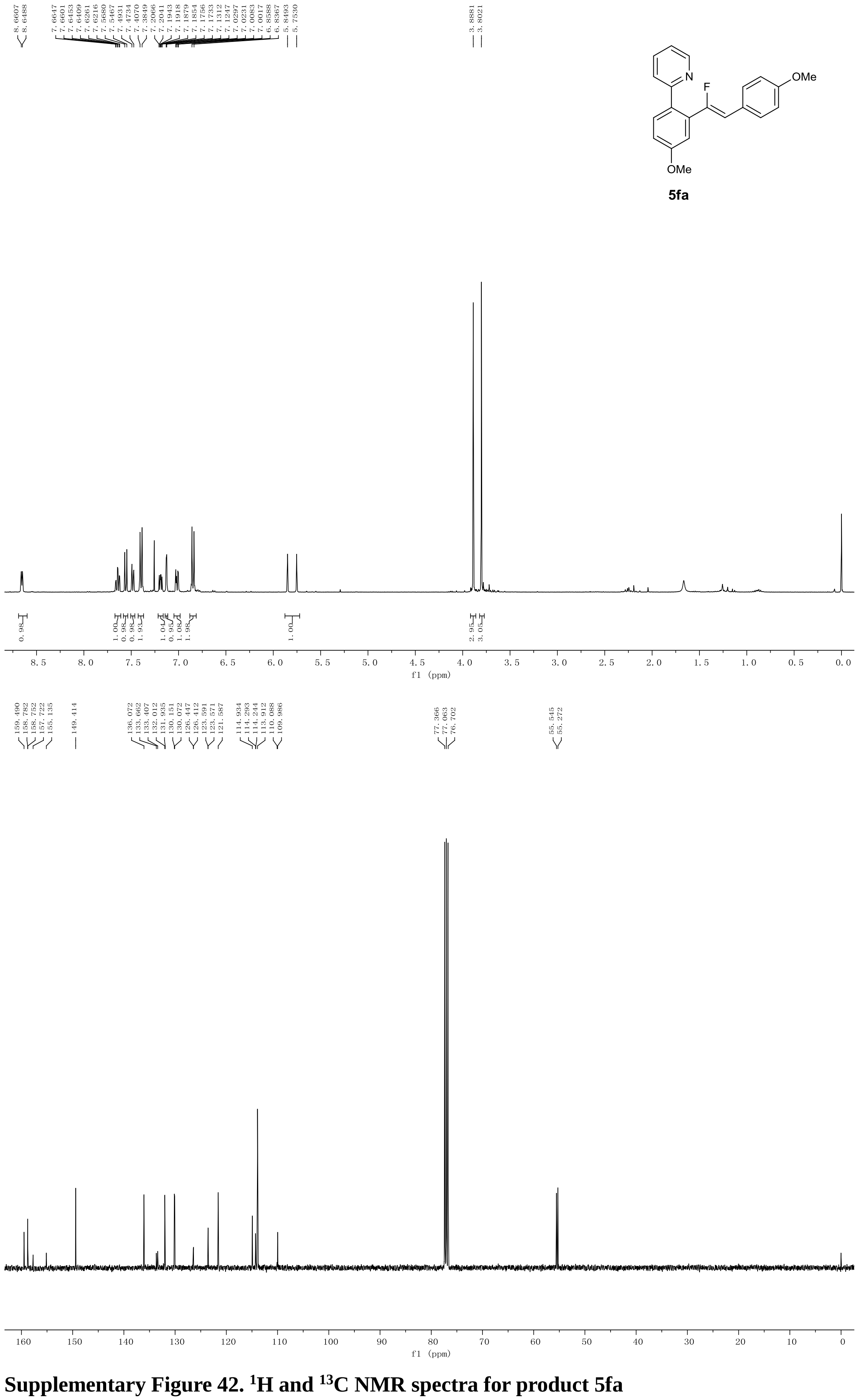

Supplementary Figure 42. ^{1}H and ^{13}C NMR spectra for product 5fa

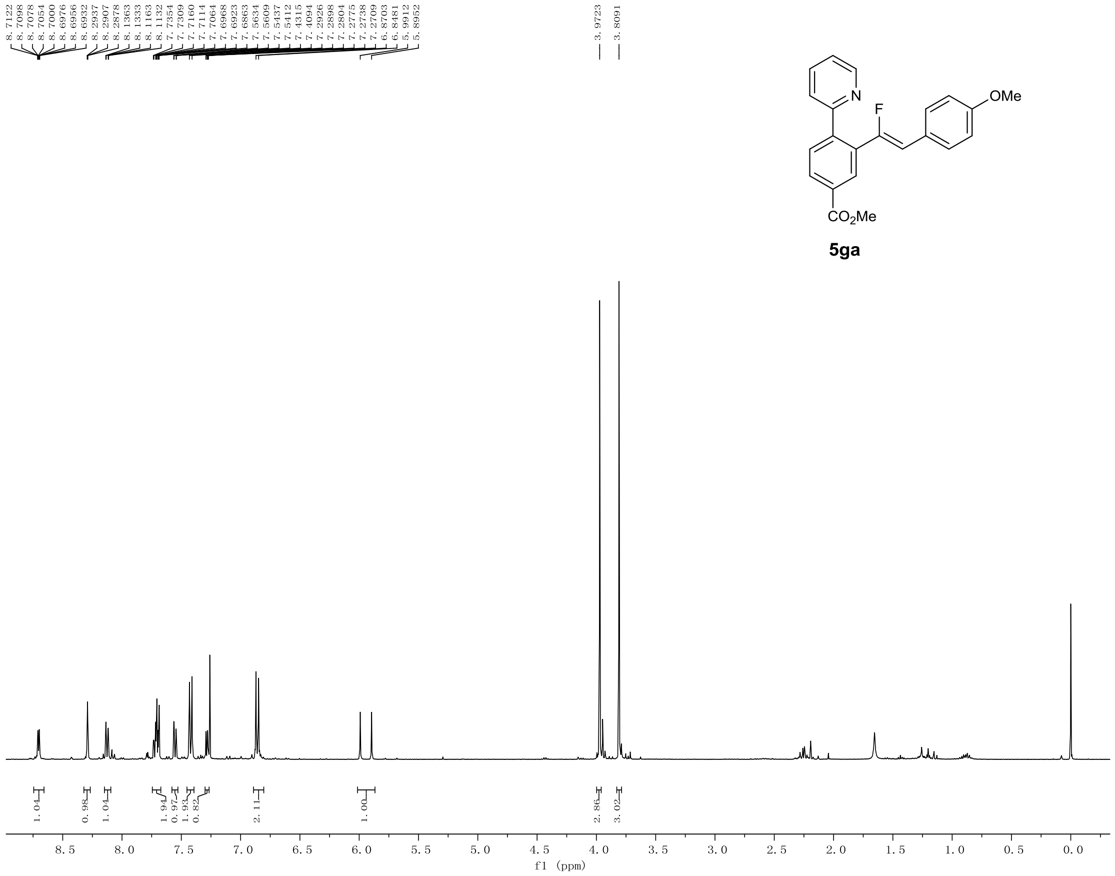

5ga

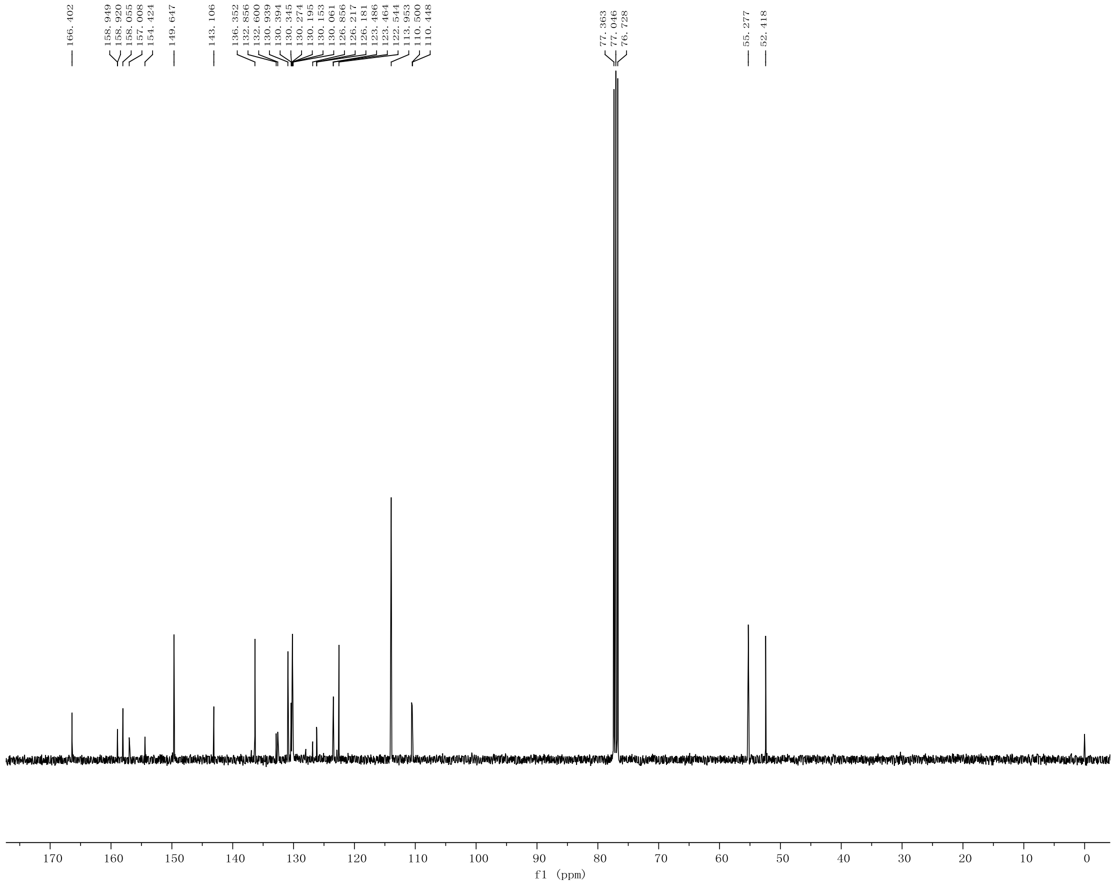

Supplementary Figure 43. ^{1}H and ^{13}C NMR spectra for product 5ga

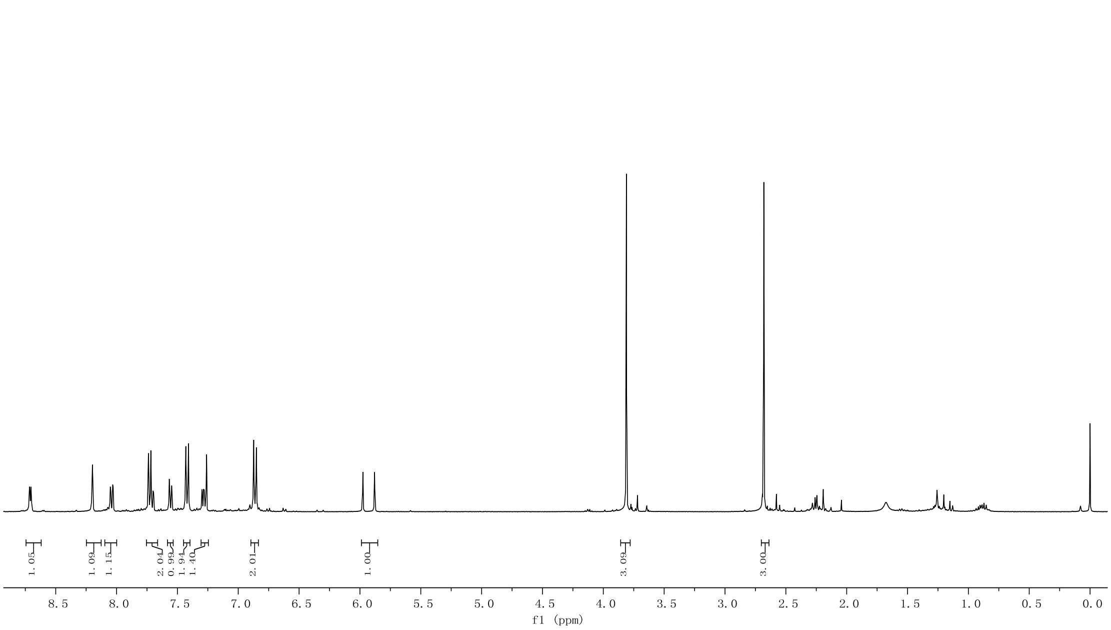

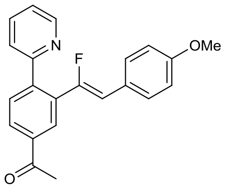

5ha

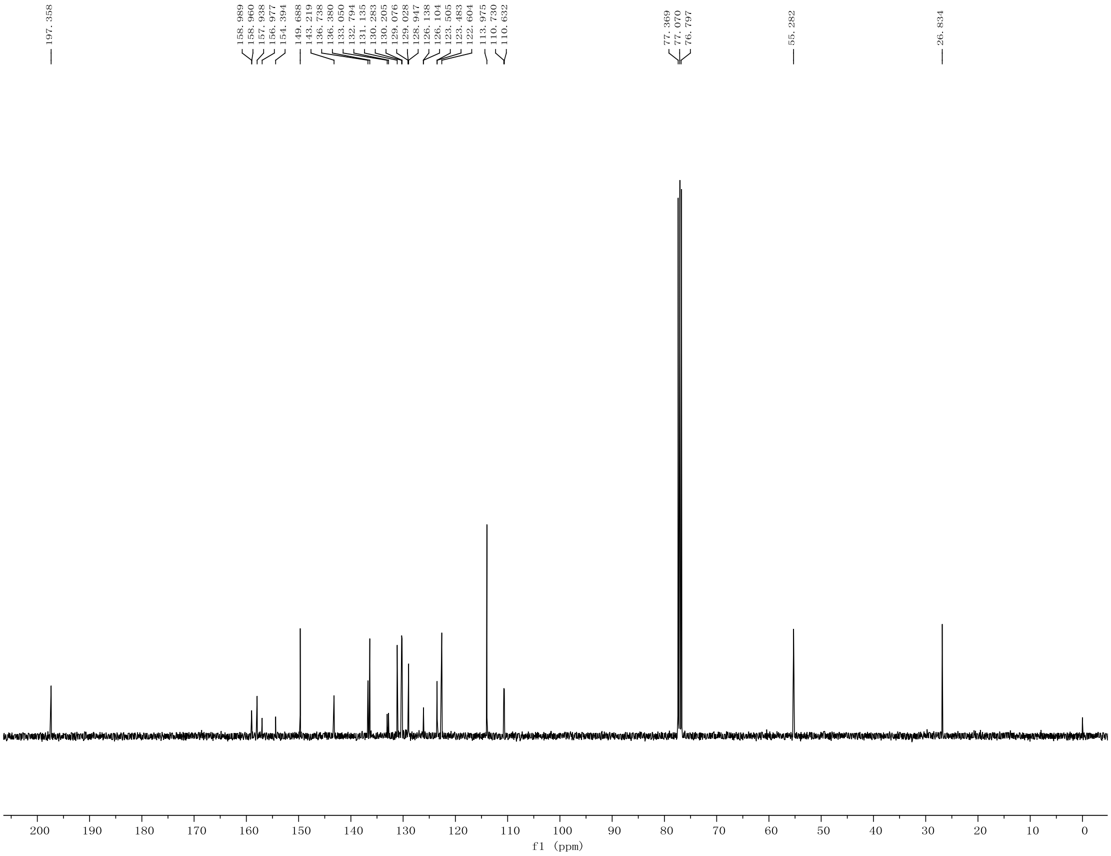

Supplementary Figure 44. ^{1}H and ^{13}C NMR spectra for product 5ha

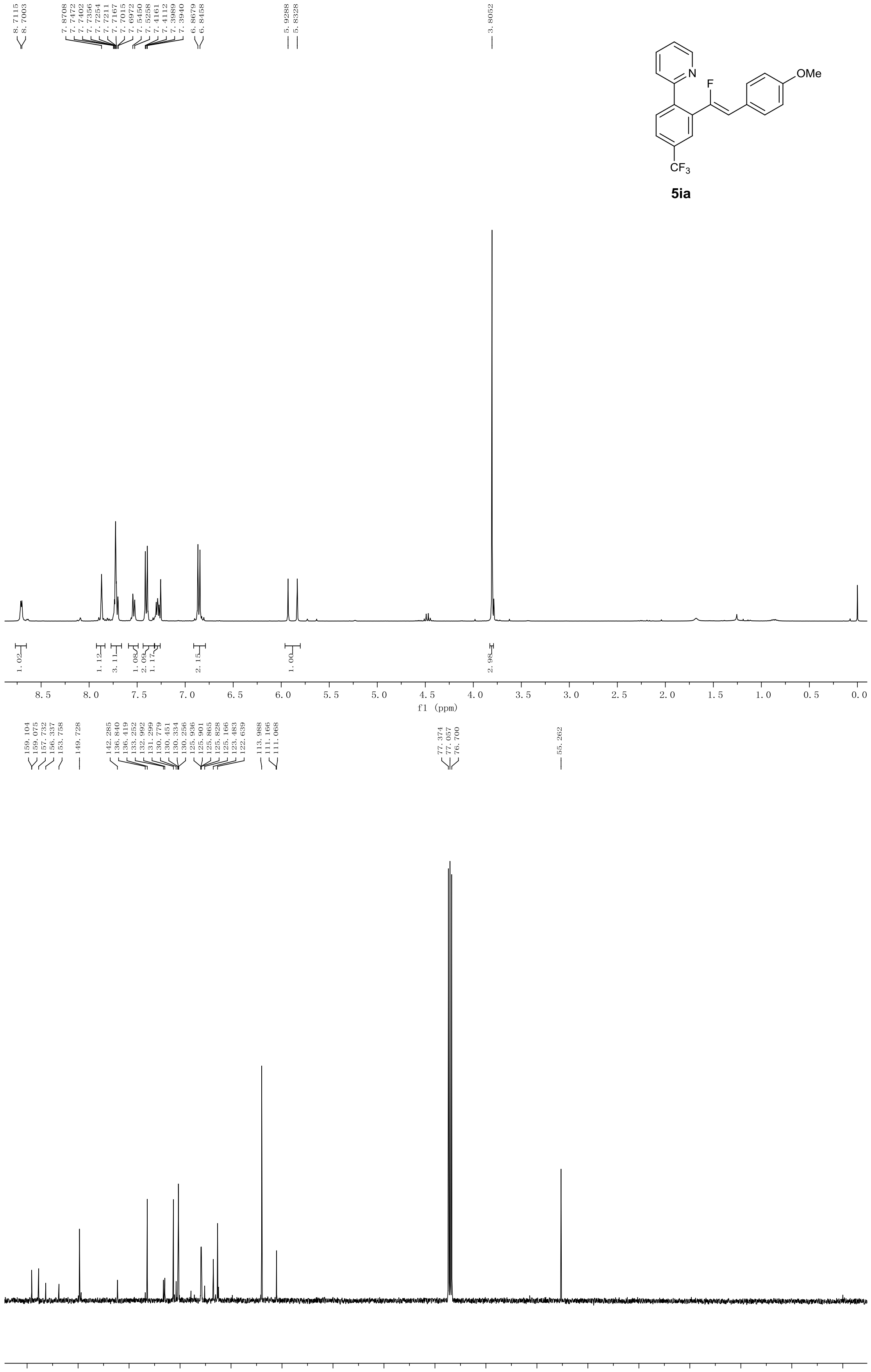

Supplementary Figure 45. ^{1}H and ^{13}C NMR spectra for product 5ia

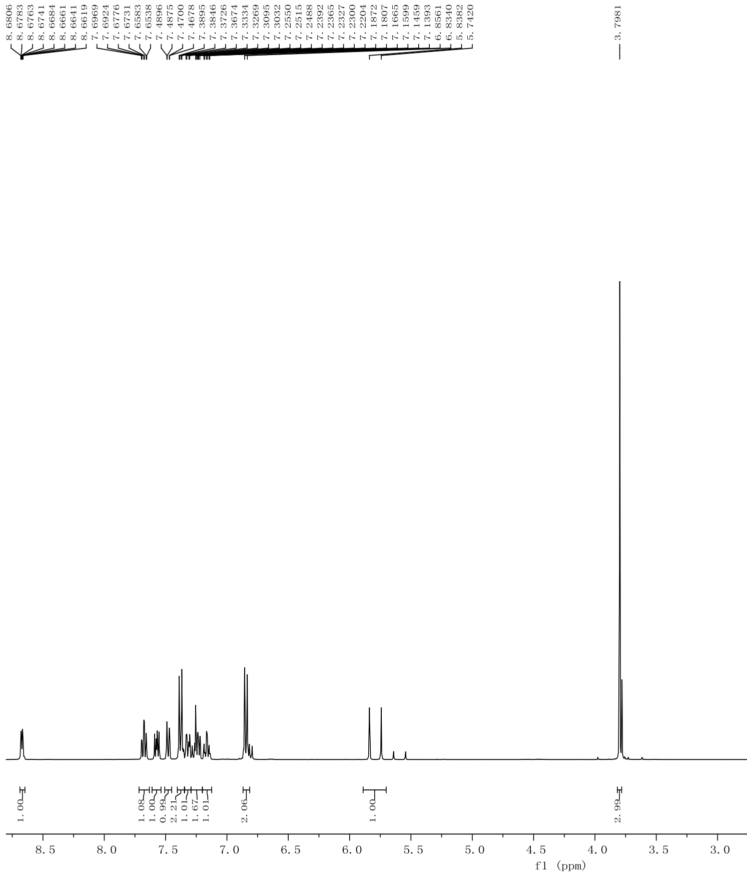

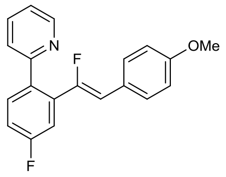

5ja, trace of di - product

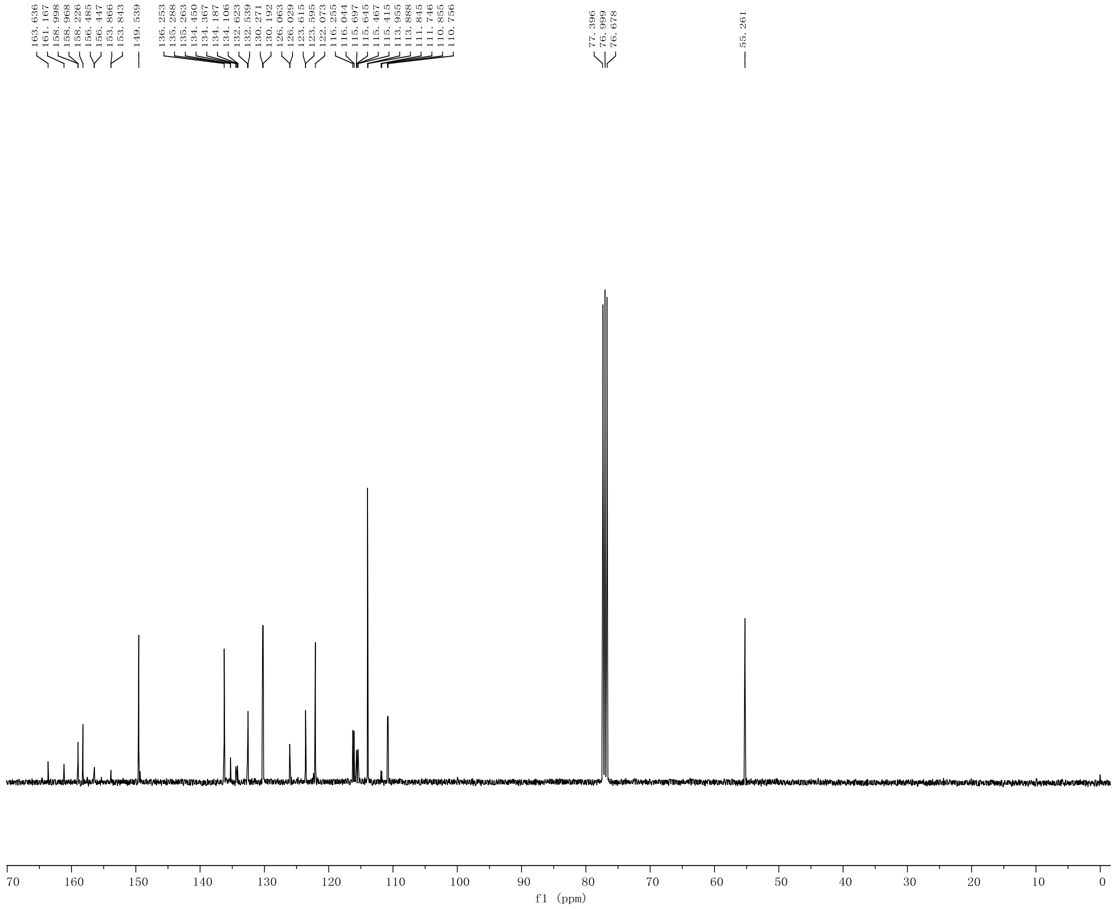

Supplementary Figure 46. ^{1}H and ^{13}C NMR spectra for product 5ja

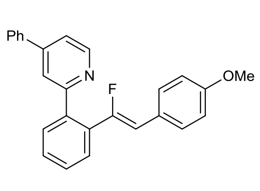

5ka

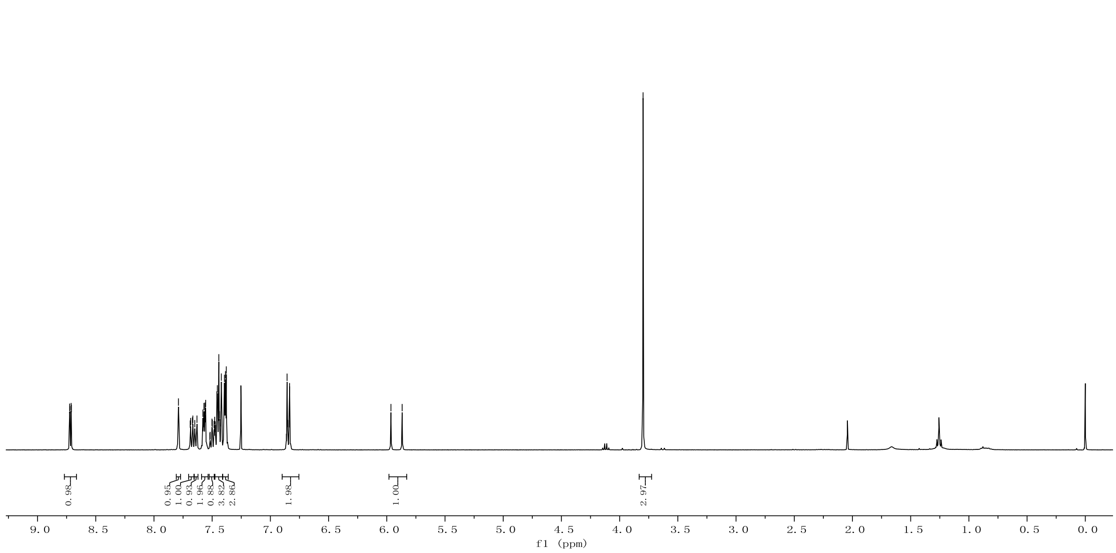

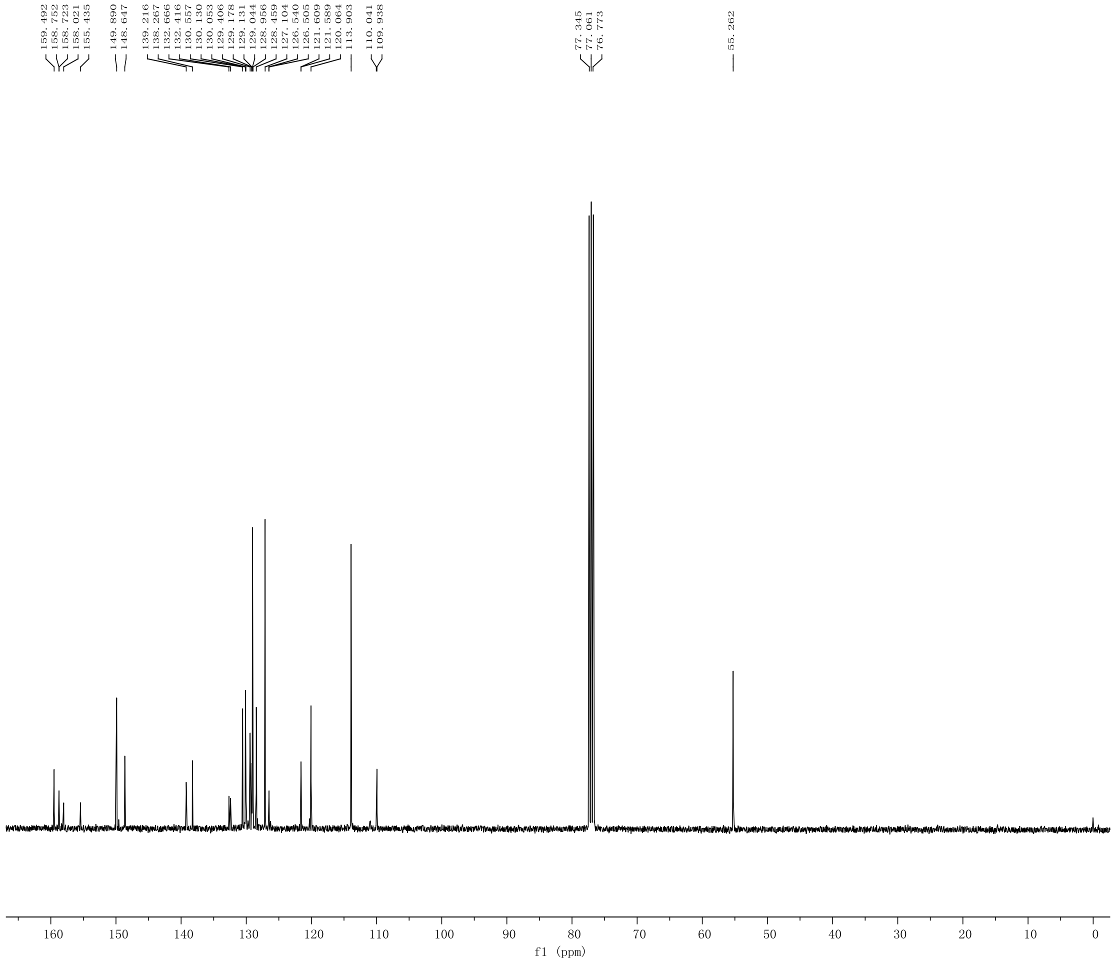

Supplementary Figure 47. ^{1}H and ^{13}C NMR spectra for product 5ka

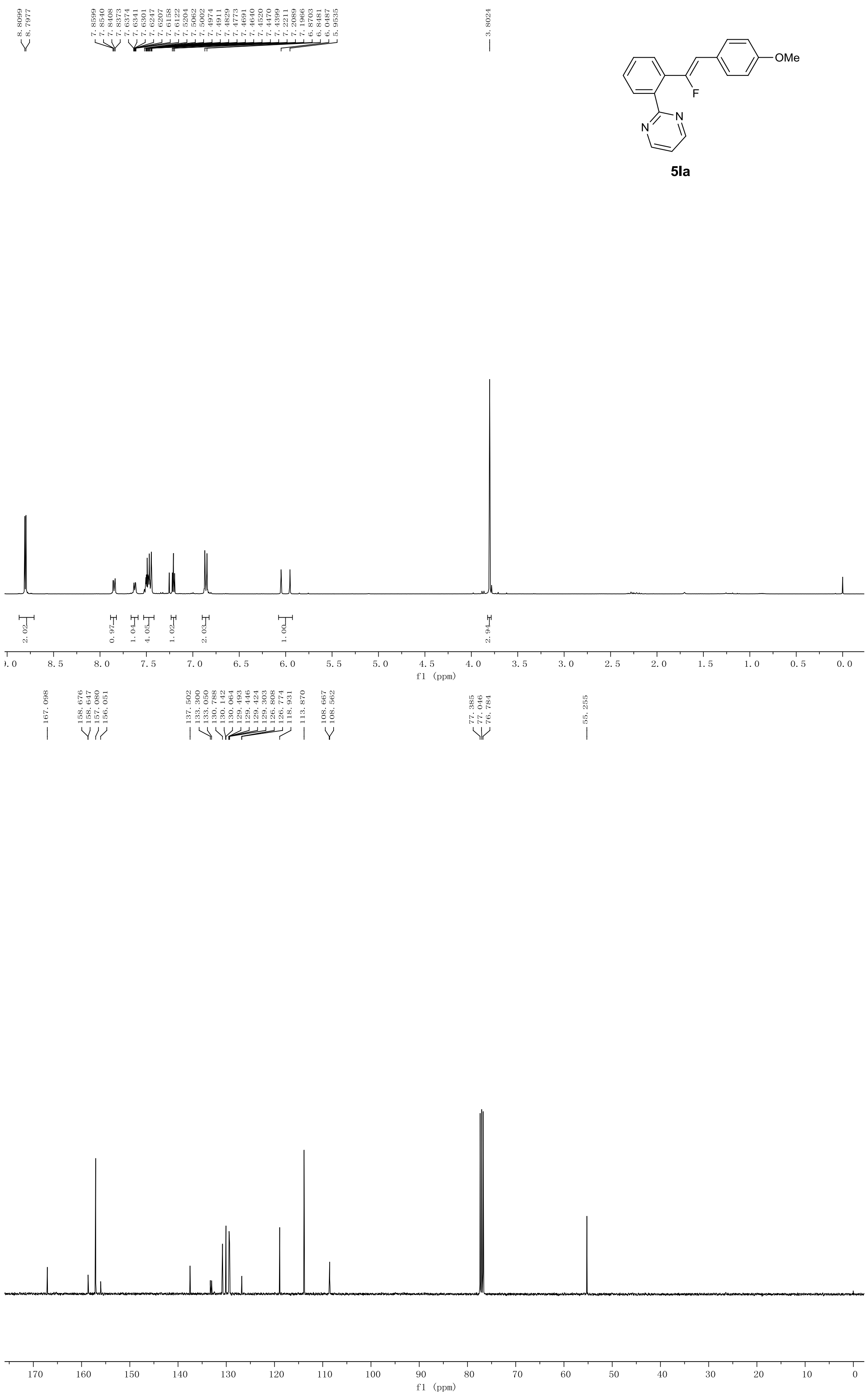

Supplementary Figure 48. ^{1}H and ^{13}C NMR spectra for product 5la

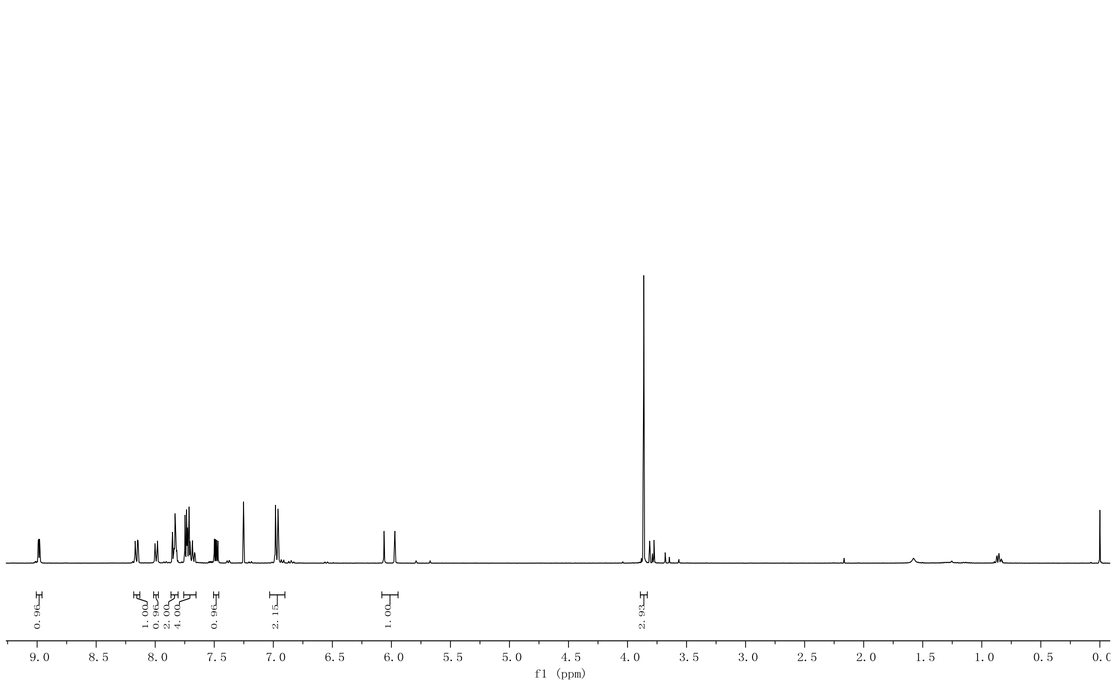

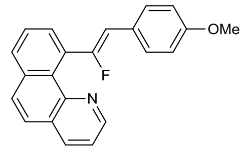

5ma

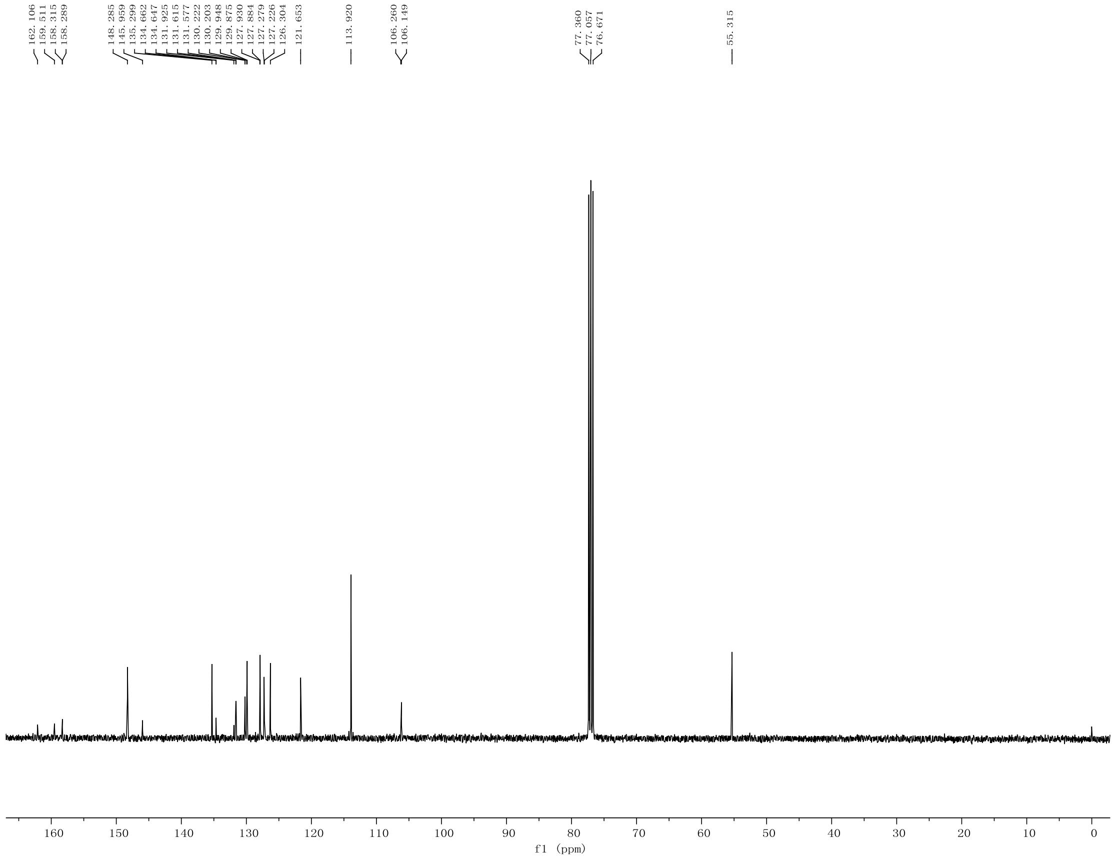

Supplementary Figure 49. ^{1}H and ^{13}C NMR spectra for product 5ma

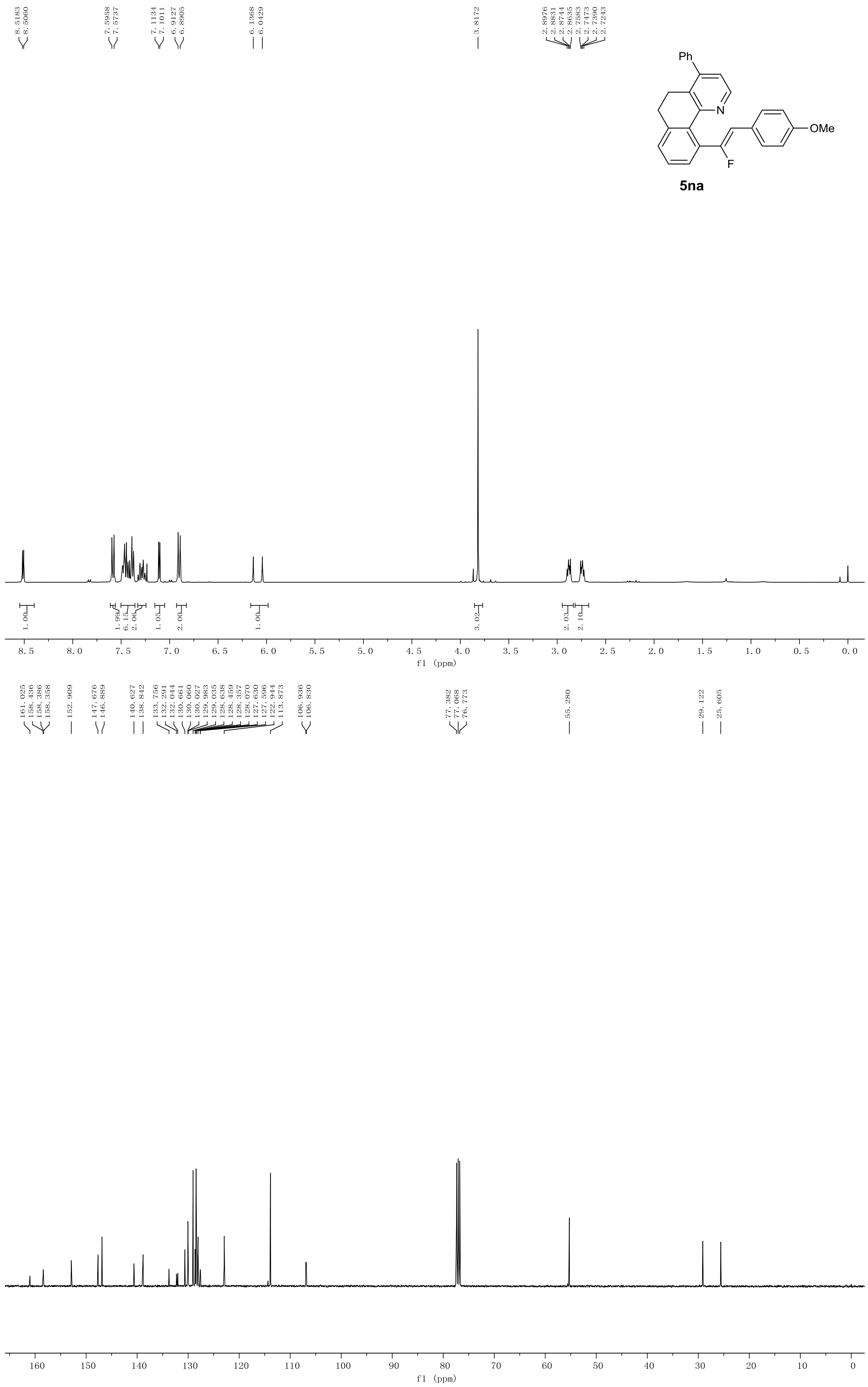

Supplementary Figure 50. ^{1}H and ^{13}C NMR spectra for product 5na

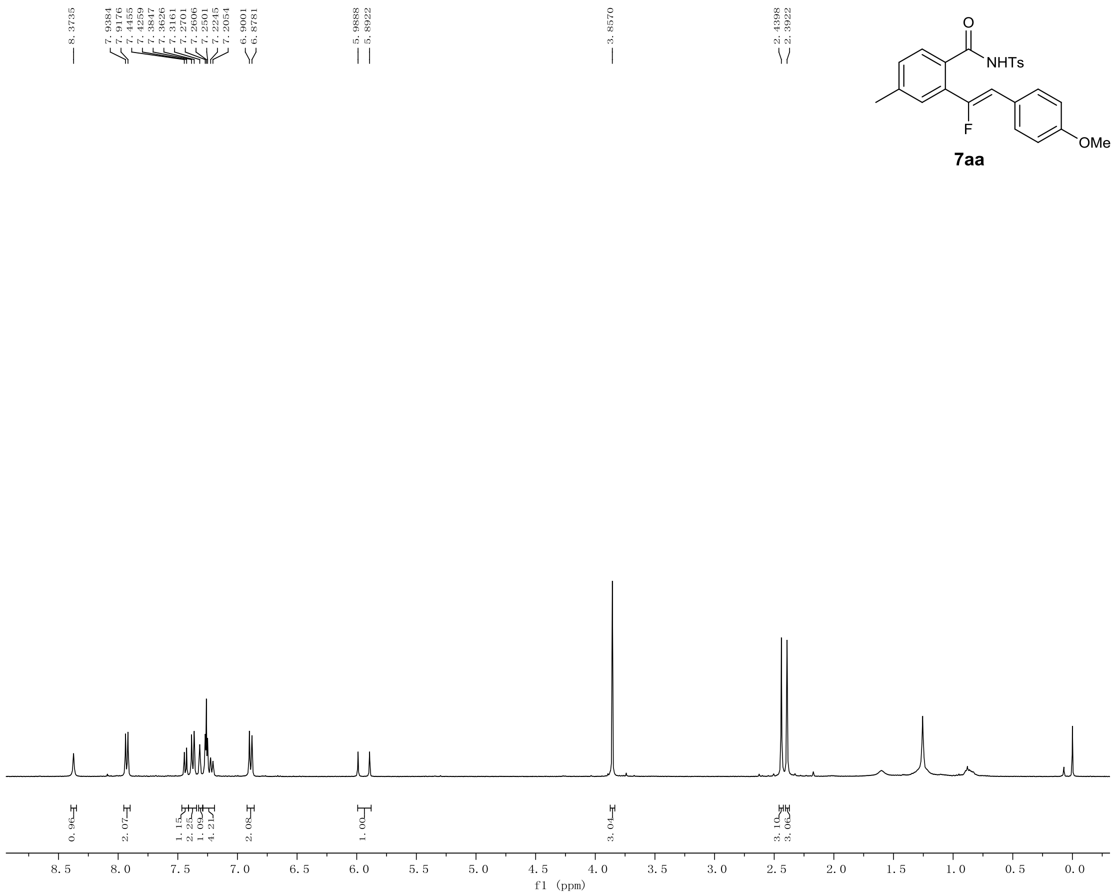

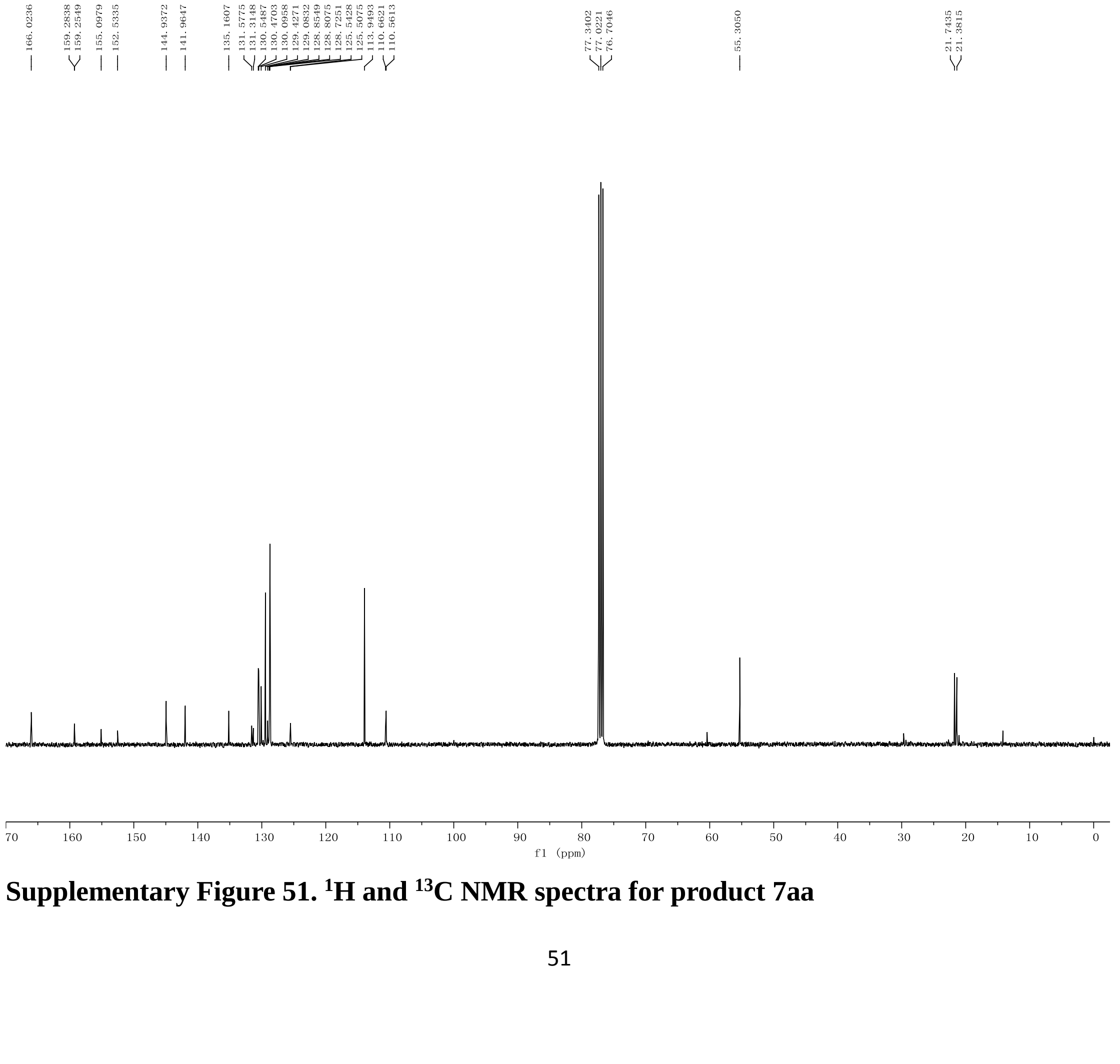

Supplementary Figure 51. ^{1}H and ^{13}C NMR spectra for product 7aa

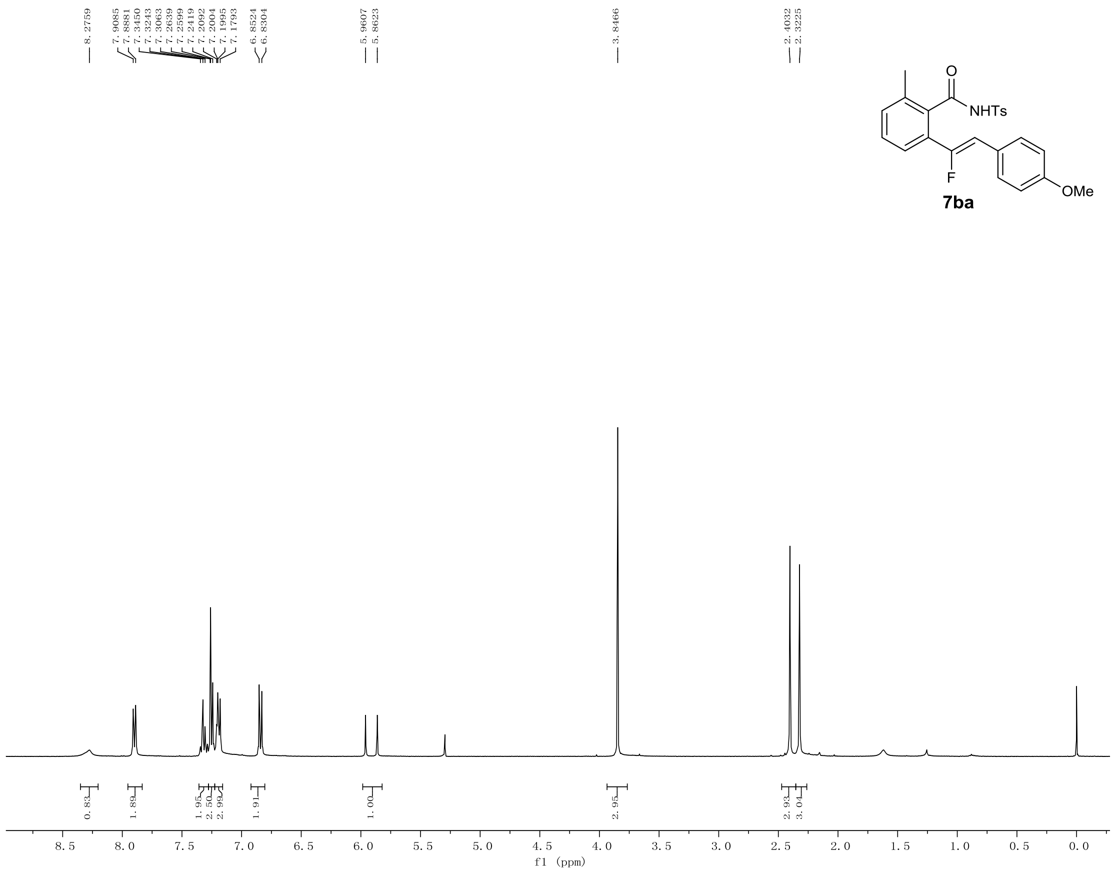

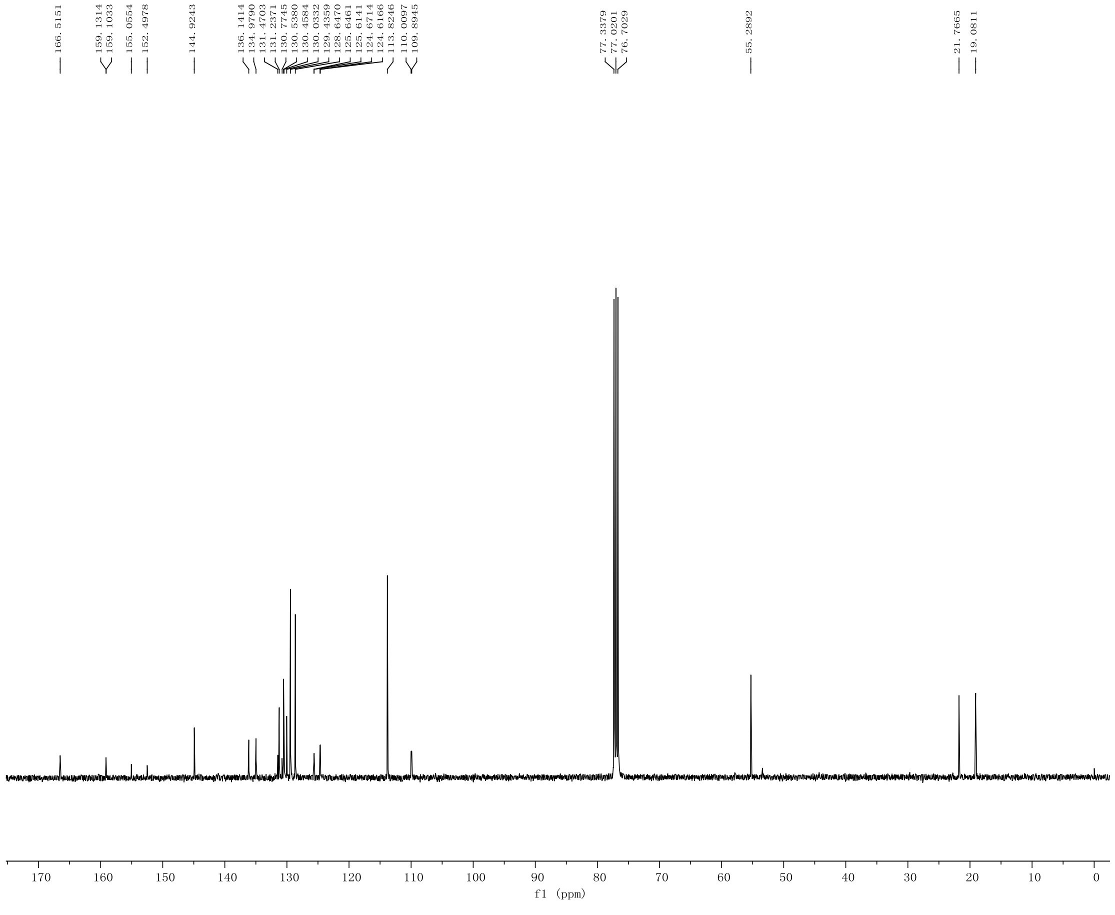

Supplementary Figure 52. ^{1}H and ^{13}C NMR spectra for product 7ba

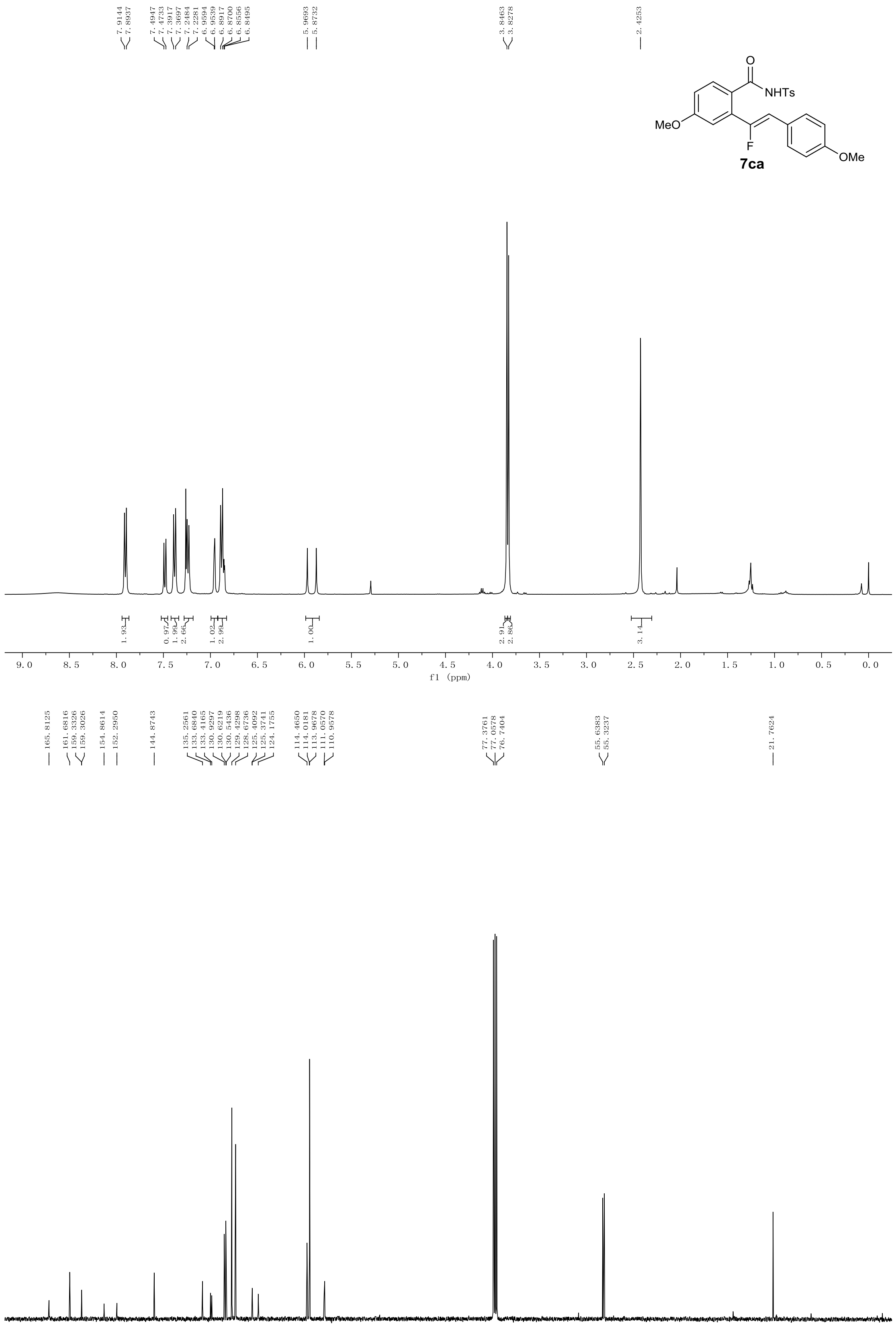

Supplementary Figure 53. ^{1}H and ^{13}C NMR spectra for product 7ca

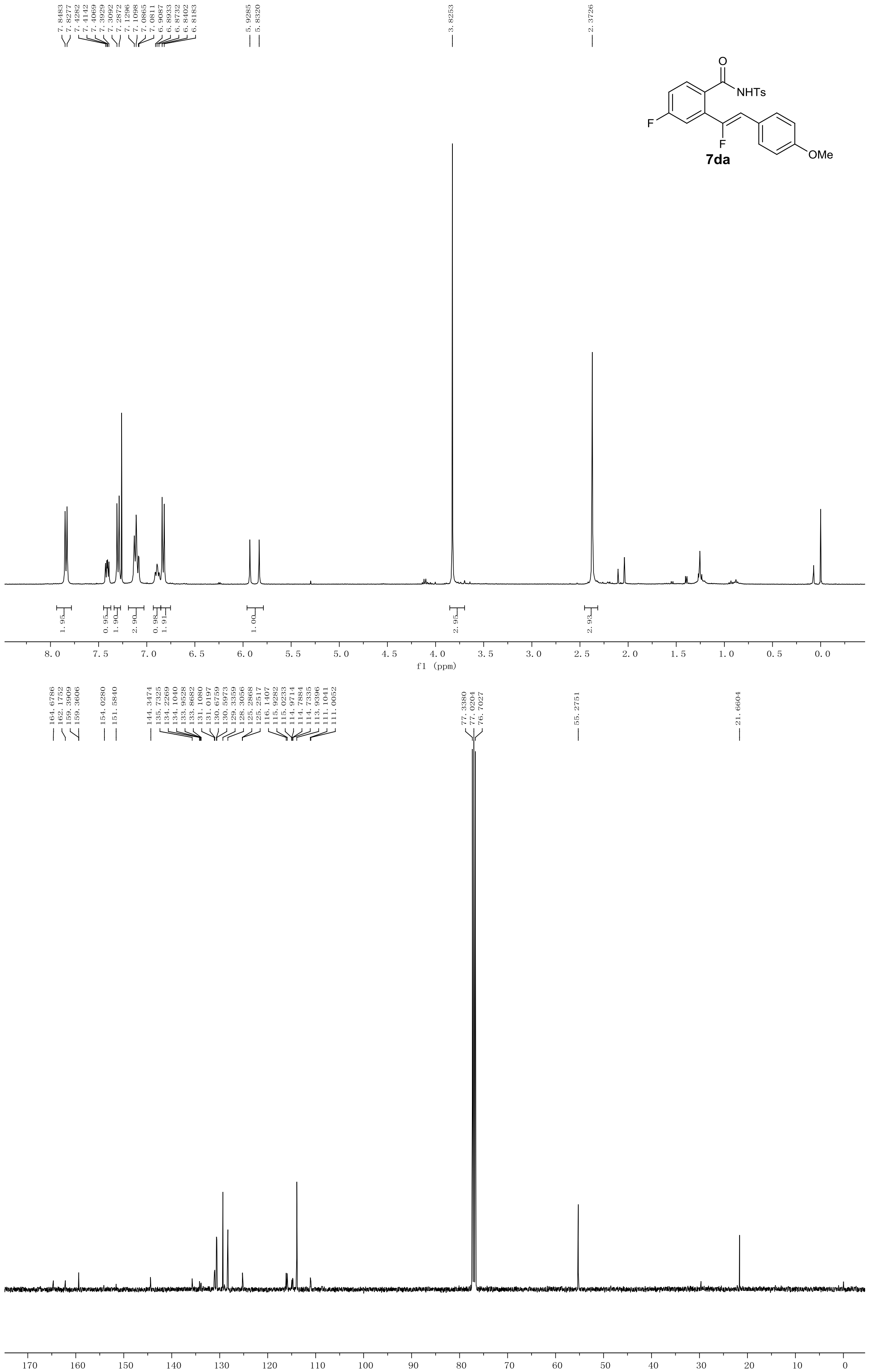

Supplementary Figure 54. ^{1}H and ^{13}C NMR spectra for product 7da

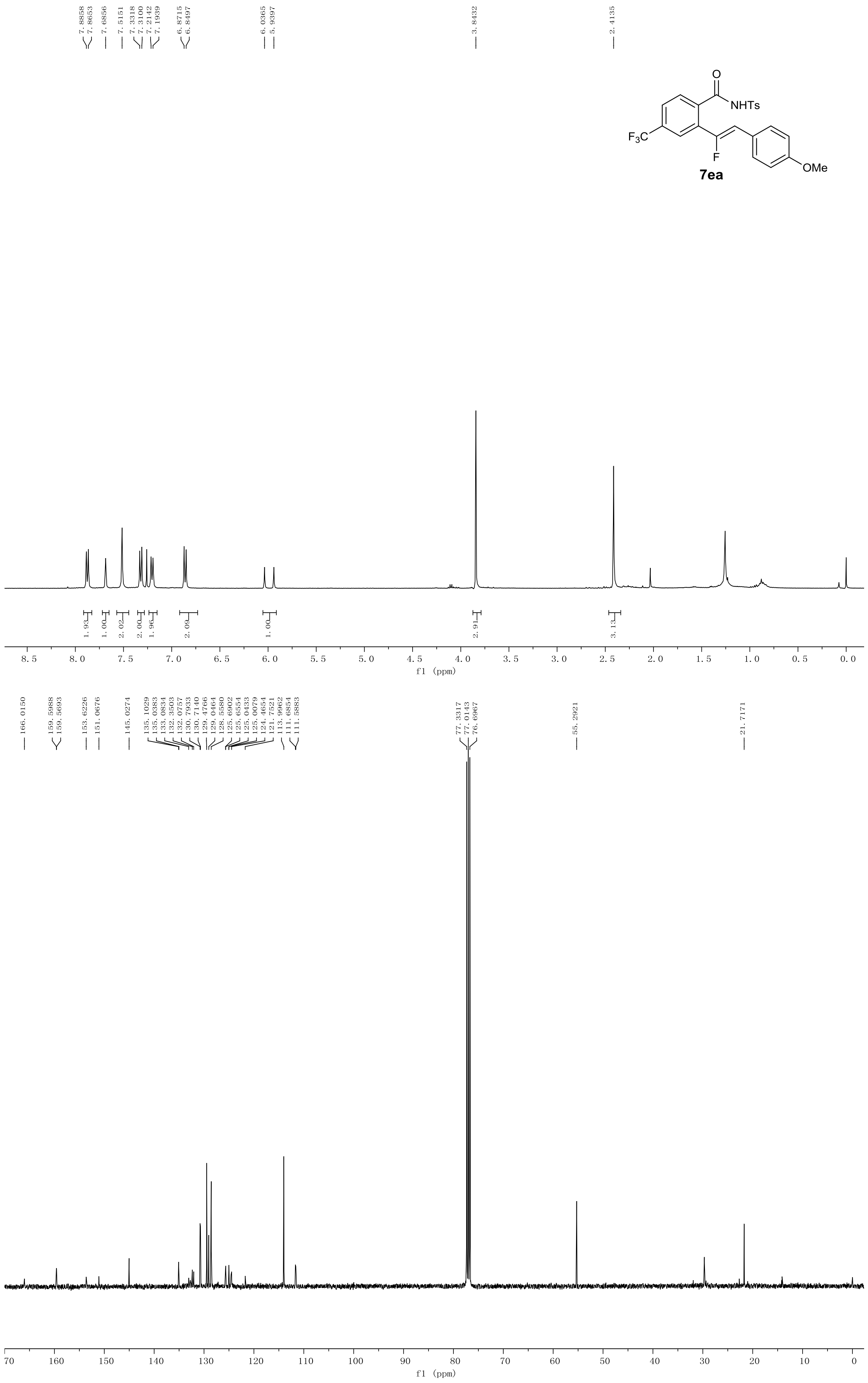

Supplementary Figure 55. ^{1}H and ^{13}C NMR spectra for product 7ea

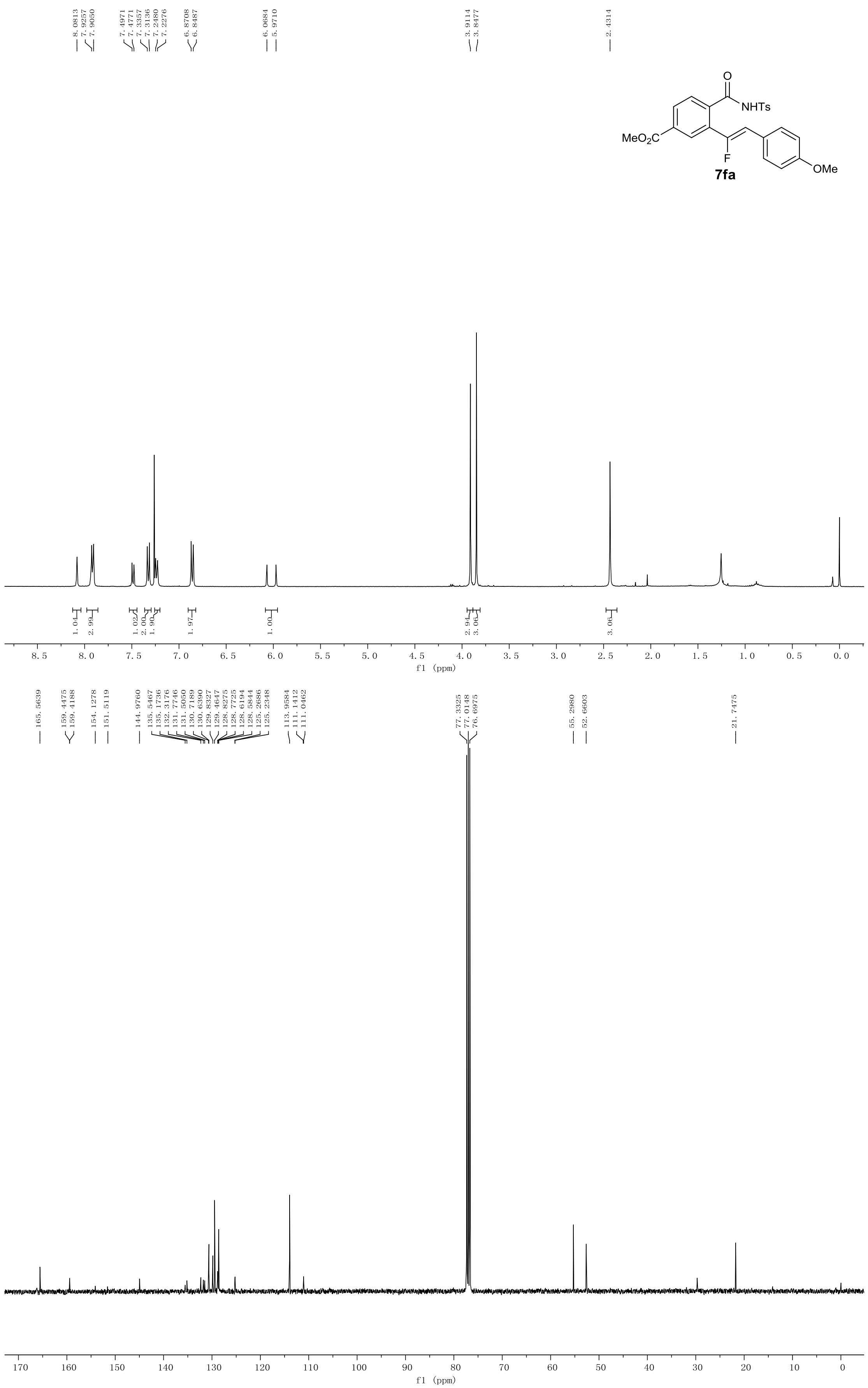

Supplementary Figure 56. ^{1}H and ^{13}C NMR spectra for product 7fa

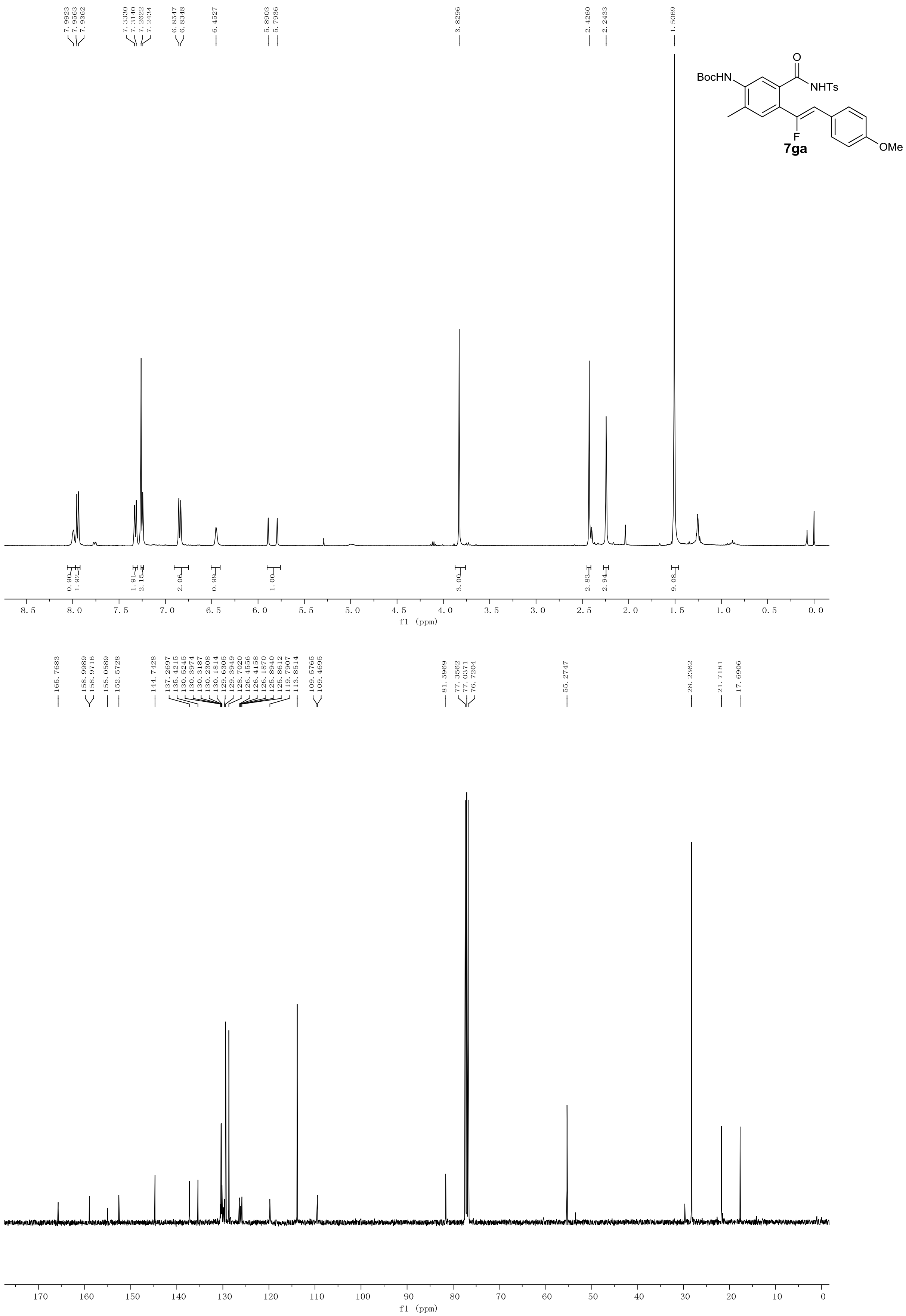

Supplementary Figure 57. ^{1}H and ^{13}C NMR spectra for product 7ga

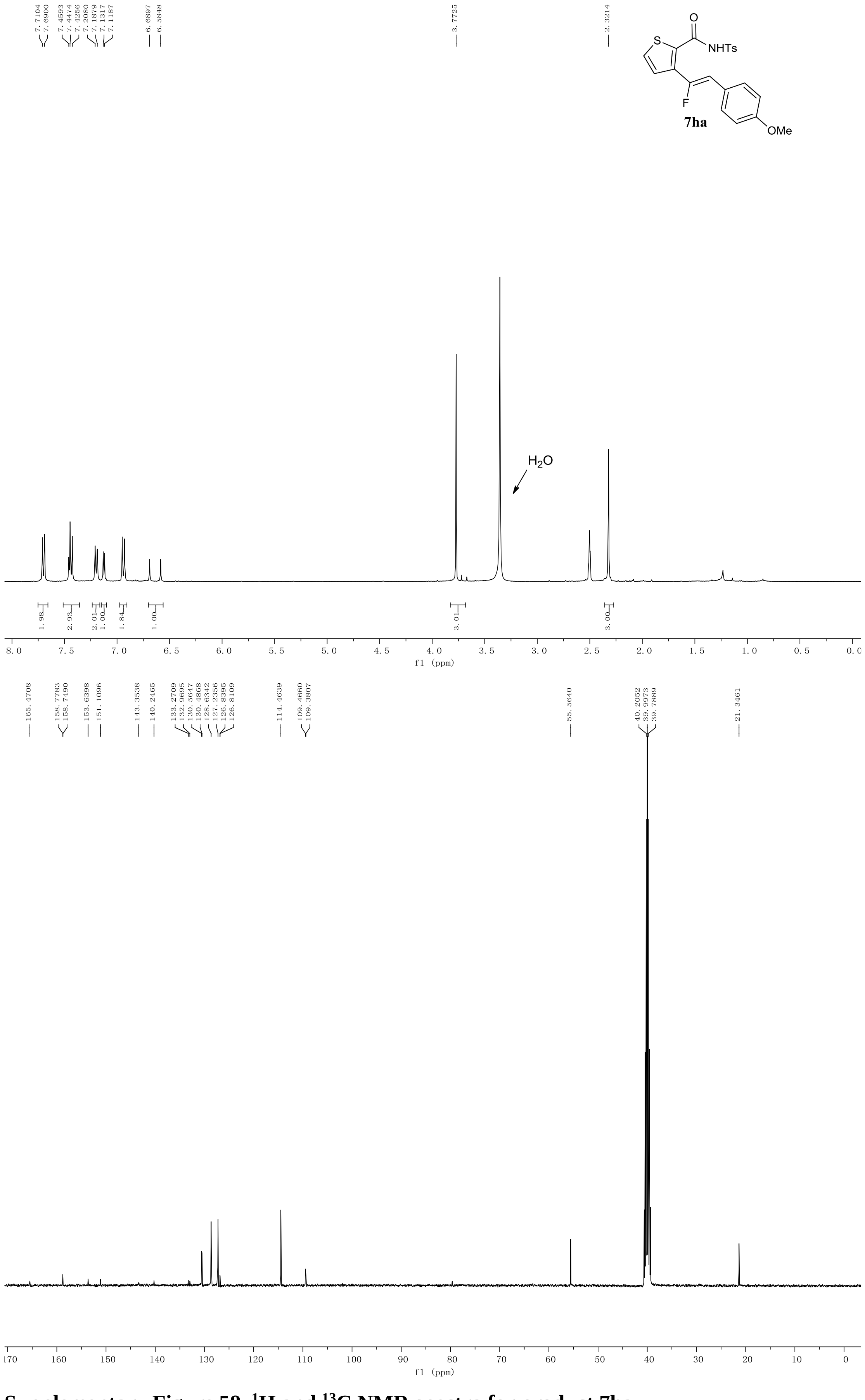

Supplementary Figure 58. ^{1}H and ^{13}C NMR spectra for product 7ha

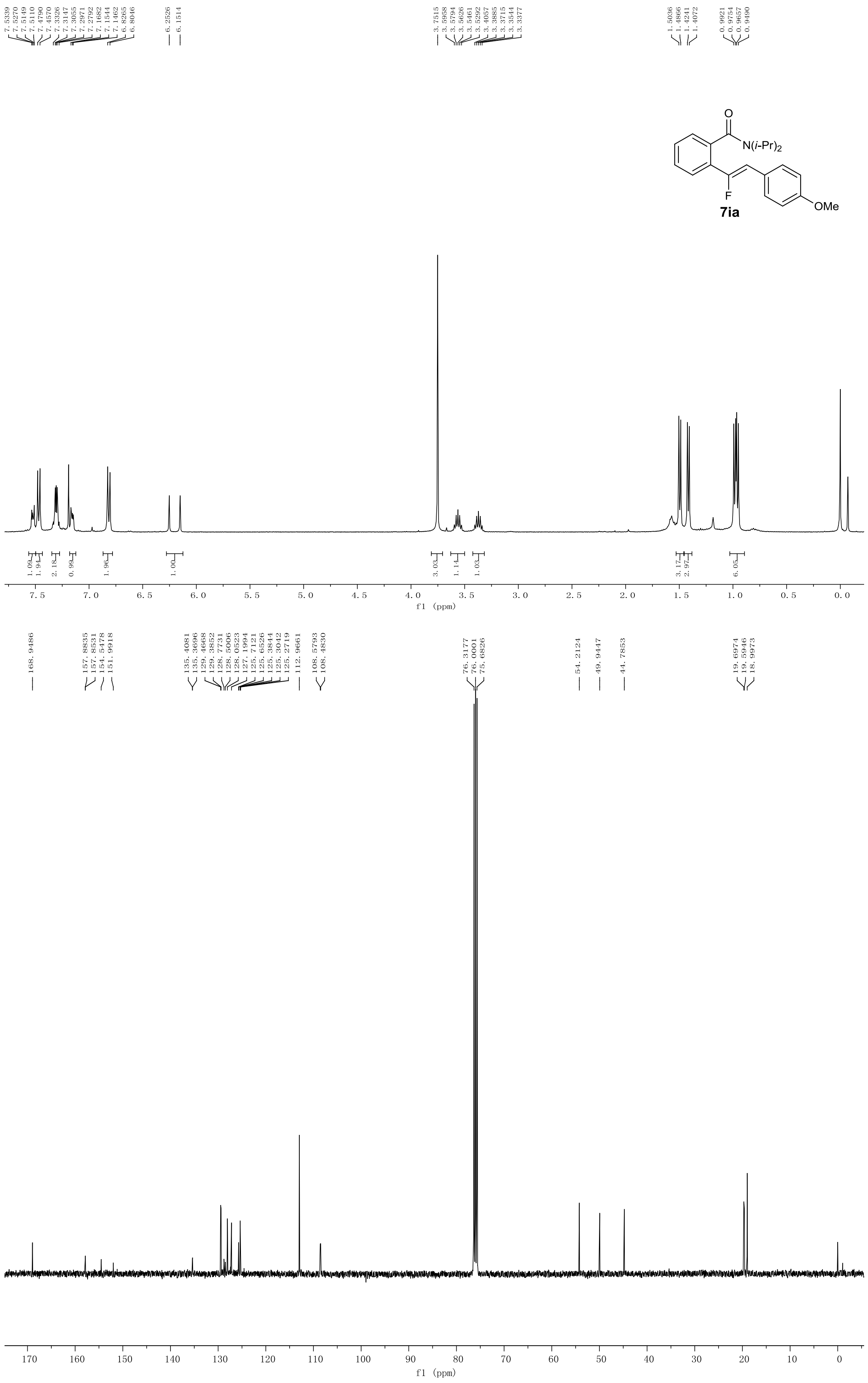

Supplementary Figure 59. ^{1}H and ^{13}C NMR spectra for product 7ia

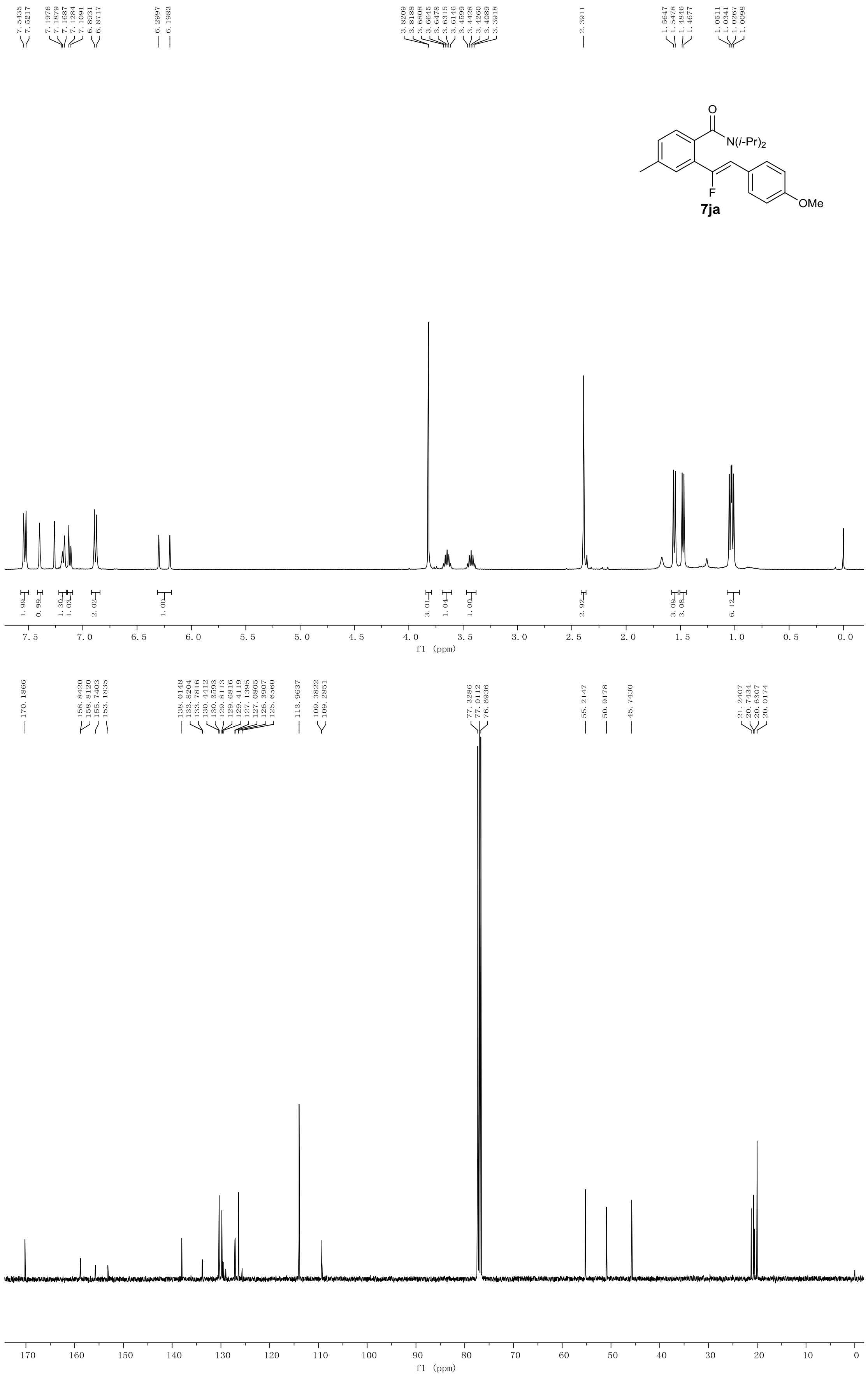

Supplementary Figure 60. ^{1}H and ^{13}C NMR spectra for product 7ja

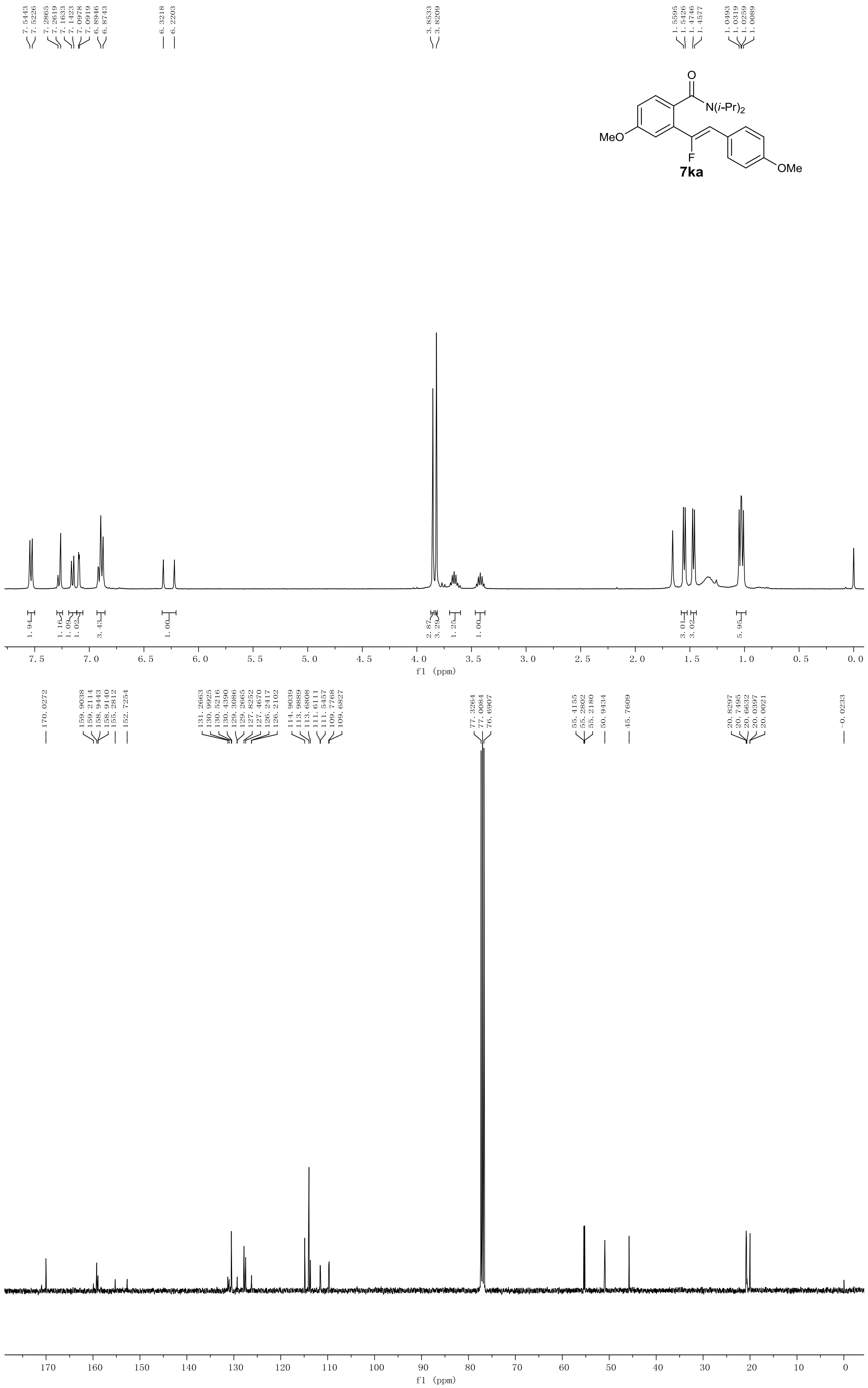

Supplementary Figure 61. ^{1}H and ^{13}C NMR spectra for product 7ka

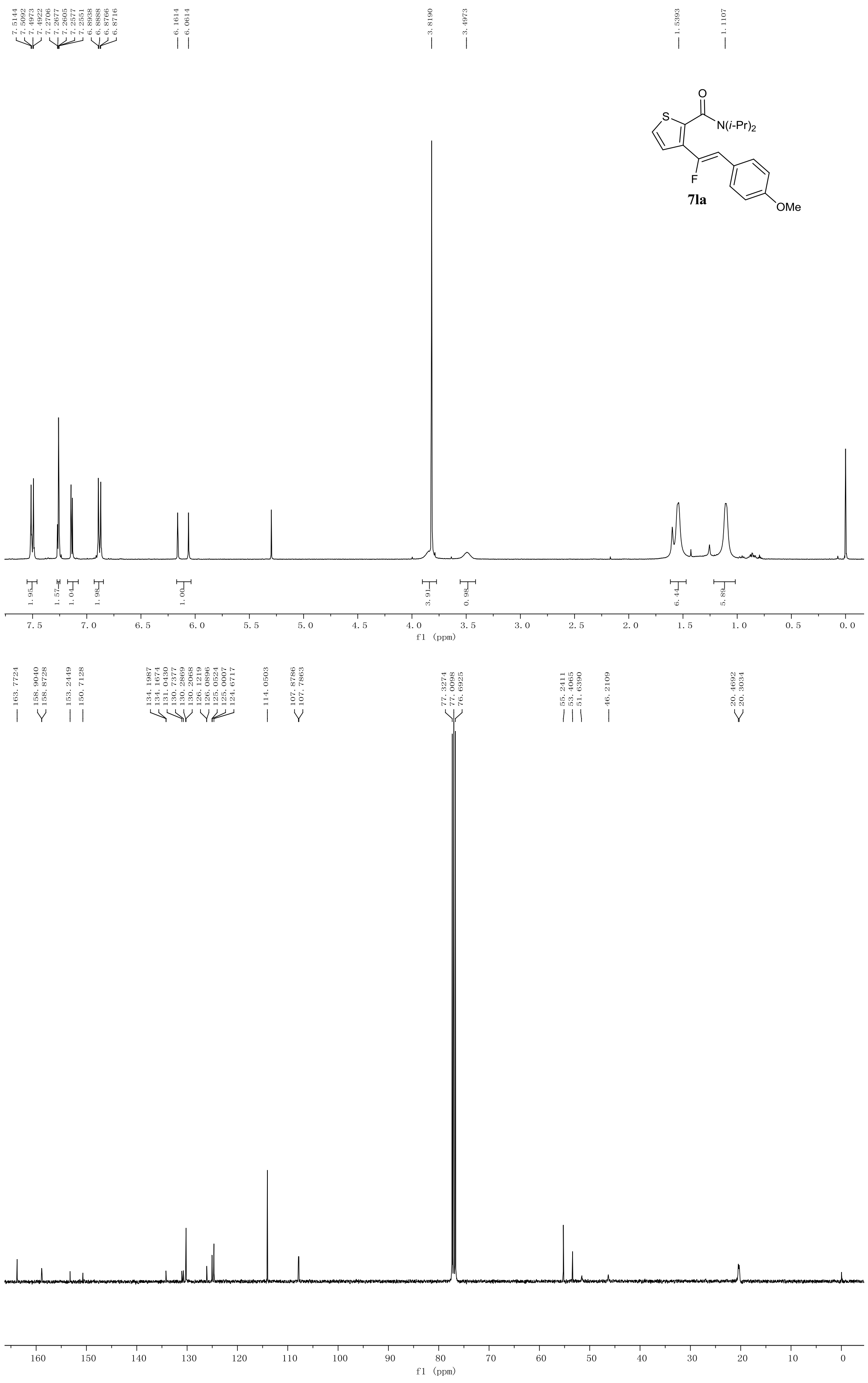

Supplementary Figure 62. ^{1}H and ^{13}C NMR spectra for product 7la

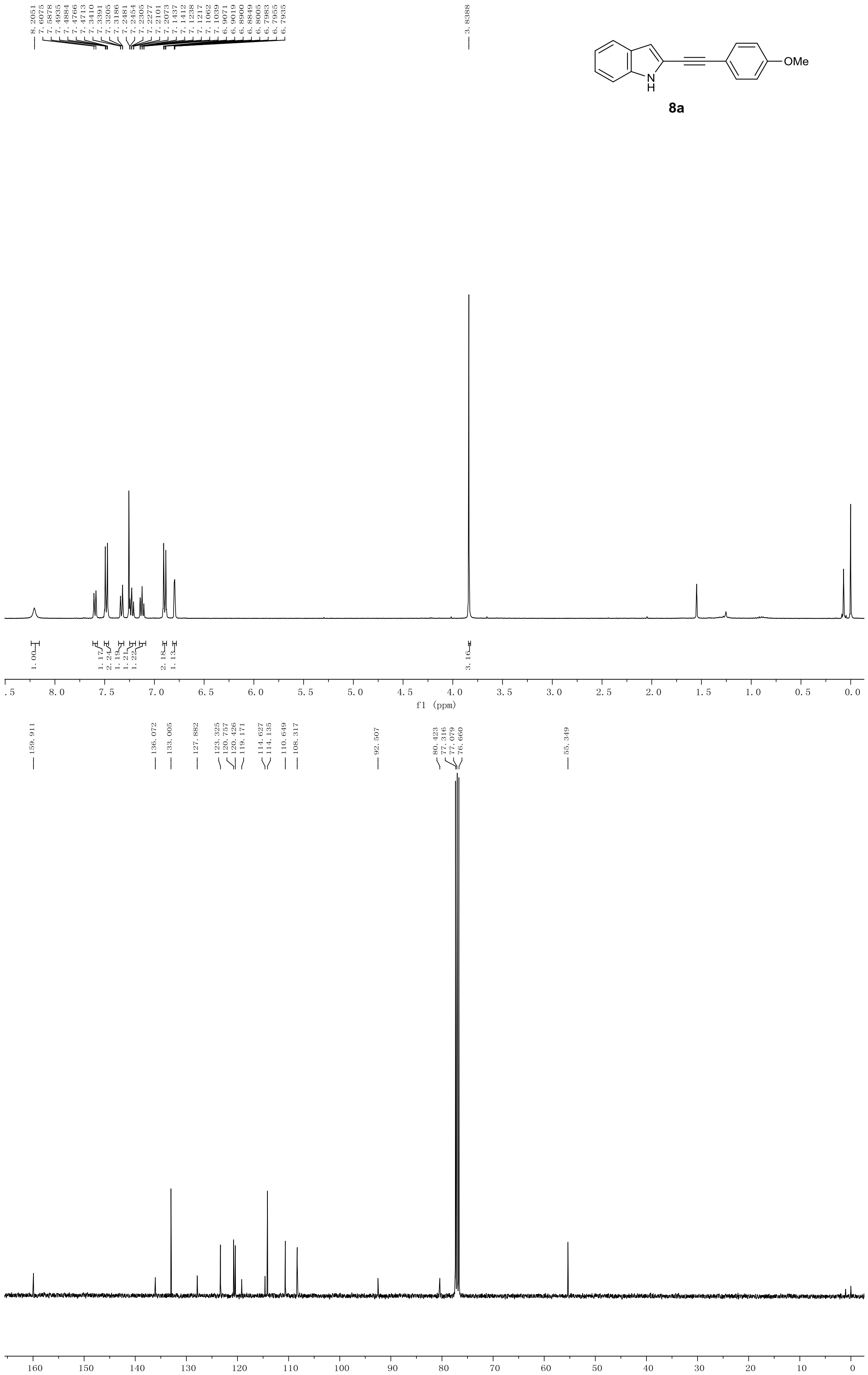

Supplementary Figure 63. ^{1}H and ^{13}C NMR spectra for product 8a

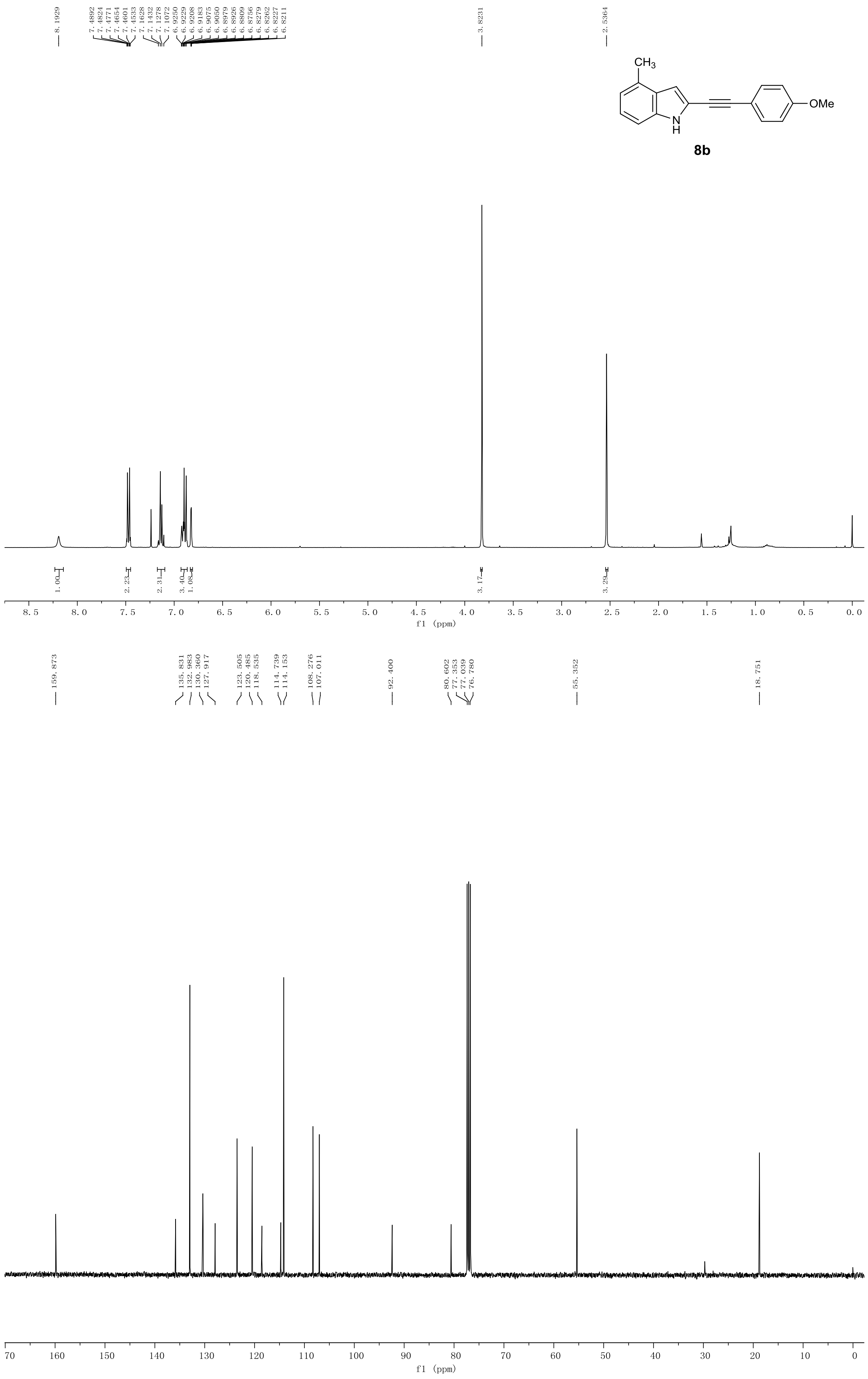

Supplementary Figure 64. ^{1}H and ^{13}C NMR spectra for product 8b

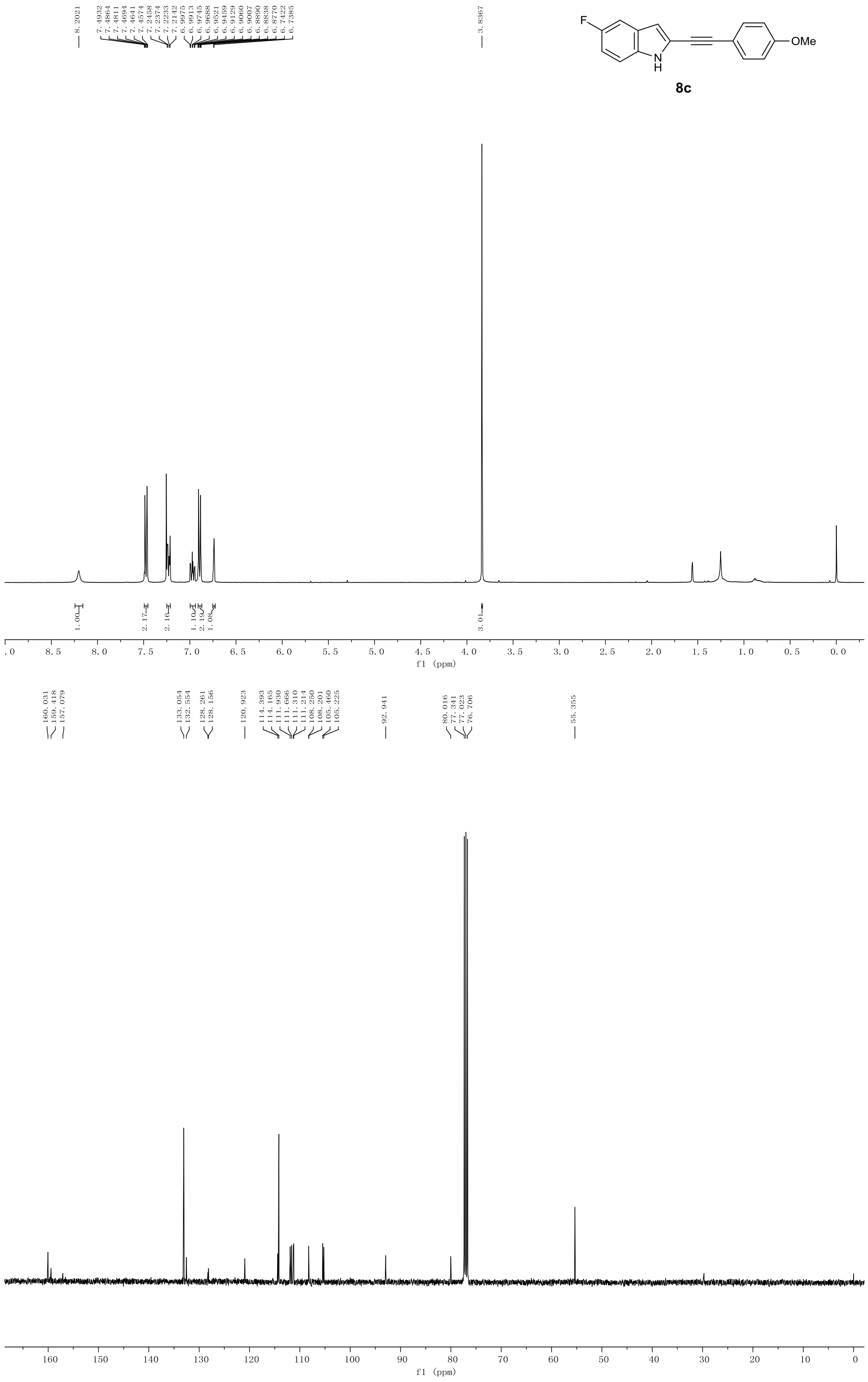

Supplementary Figure 65. ^{1}H and ^{13}C NMR spectra for product 8c

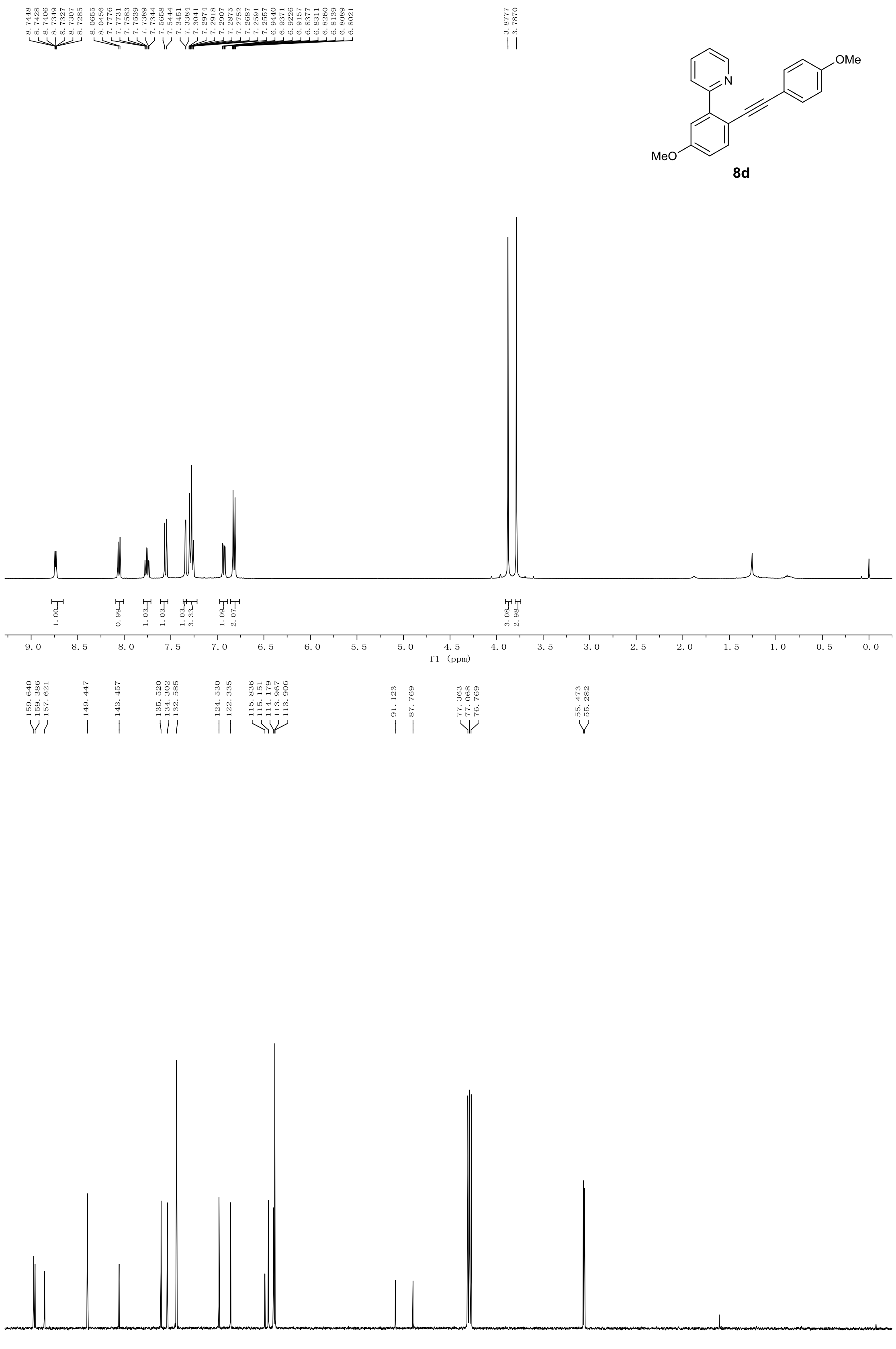

Supplementary Figure 66. ^{1}H and ^{13}C NMR spectra for product 8d

Supplementary Table

Table 1: Solvent screening

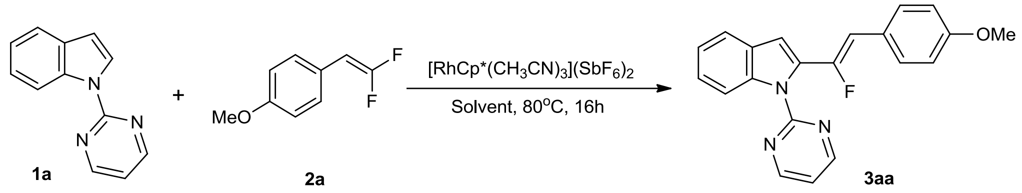

Entry	Solvent	Yield(%)
1	CF_3CH_2OH	45%[a]
2	MeOH	89%[a]
3	THF	75%[b]
4	Acetone	71%[b]
5	DCE	75%[b]
6	Cl-Ph	29%[b]

[a]Isolated yield.
[b]Yield was determined by ^{1}H NMR using mesitylene as internal standard.

Procedure: An oven-dried 10 ml Schlenk tube was charged with **1a** (0.1 mmol), $[RhCp^*(CH_3CN)_3](SbF_6)_2$ (0.004 mmol), **2a** (0.15 mmol) in sequence, followed by adding anhydrous solvent (0.5ml) through syringe and stirring at 80 ^oC for 16 h, then removal of the solvent in vacuo and purification of the residue by silica gel column chromatography afforded the desired product **3aa**.

Table 2: Base screening

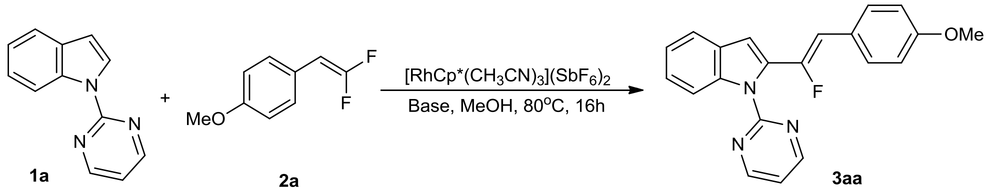

Entry	Base	Yield (%)[a]
1	$NaHCO_3$	NR
2	CsF	75%
3	Na_2CO_3	NR
4	2,6-di-tert-butyl-4-methylpyridine	NR

[a]Isolated yield.

Procedure: An oven-dried 10 ml Schlenk tube was charged with **1a** (0.1 mmol),

$[RhCp^*(CH_3CN)_3](SbF_6)_2$ (0.004 mmol), Base (0.2 mmol), **2a** (0.15 mmol) in sequence, followed by adding anhydrous MeOH (0.5ml) through syringe and stirring at 80 °C for 16 h, then removal of the solvent in vacuo and purification of the residue by silica gel column chromatography afforded the desired product **3aa**.

Table 3: Catalyst screening

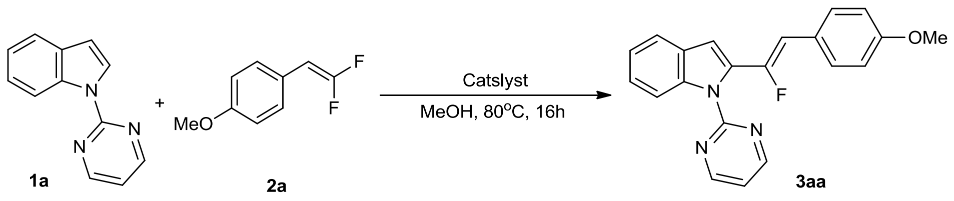

Entry	Catalyst	Yield (%)[a]
1	$[Cp^*Rh(CH_3CN)_3](SbF_6)_2$	89%
2	$[Cp^*RhCl_2]_2$	trace
3	$[Cp^*RhCl_2]_2$	85%[b]
4	$[RuCl_2(p\text{-cymene})]_2$	NR[b]
5	$Pd(OAc)_2$	NR

[a]Isolated yield. [b]Adding 10 mol% of $AgSbF_6$.

Procedure: An oven-dried 10 ml Schlenk tube was charged with **1a** (0.1 mmol), Catalyst (0.004 mmol), **2a** (0.15 mmol) in sequence, followed by adding anhydrous MeOH (0.5ml) and stirring at 80 °C for 16 h, then removal of the solvent in vacuo and purification of the residue by silica gel column chromatography afforded the desired product **3aa**.

Supplementary Methods

General Information

$[Cp^*Rh(CH_3CN)_3](SbF_6)_2$, *t*-BuOK, anhydrous MeOH, CF_3CH_2OH and THF were purchased from commercial suppliers and used as received unless otherwise noted. All reactions were carried out under air without extra protection. Reactions were monitored through thin layer chromatography [Merck 60 F254 precoated silica gel plate (0.2 mm thickness)]. Subsequent to elution, spots were visualized using UV radiation (254 nm) on Spectroline Model ENF-24061/F 254 nm. Further visualization was possible using basic solution of potassium permanganate as stain. Flash chromatography was performed using Merck silica gel 60 with distilled solvents. HRMS spectra were recorded on a Waters Q-Tof Permier Spectrometer. ^1H NMR and ^{13}C NMR spectra were recorded using Bruker Avance 400 MHz spectrometers. Chemical shifts for ^1H NMR spectra are reported as δ in units of parts per million (ppm) downfield from $SiMe_4$ (δ 0.0) and relative to the signal of $SiMe_4$ (δ 0.00, singlet). Multiplicities were given as: s (singlet); d (doublet); t (triplet); q (quartet); dd (doublets of doublet); ddd (doublets of doublets of doublet); td (triplet of doublet); m (multiplets) and etc. Coupling constants are reported as a *J* value in Hz. Carbon

nuclear magnetic resonance spectra (^{13}C NMR) are reported as δ in units of parts per million (ppm) downfield from $SiMe_4$ (δ 0.0) and relative to the signal of chloroform-*d* (δ 77.00, triplet).

General procedure for preparation of substrates

4n was purchased from commercial supplier. Difluoroolefins **2a**[1], **2b**[2], **2c**[1], **2d-2i**[2], **2j**[1], **2k-2u**[2], Indole derivatives **1a-1o**[3], **1p**[4], arylpyridines **4a-4j**[5], **4k**[6], **4l-4m**[7], **4o**[6] were prepared according to the reported methods.

General reaction precdure for 2a, 2c, 2j syntheses:

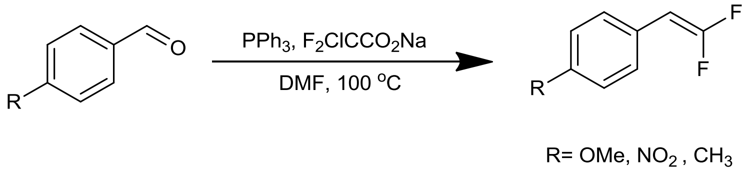

Procedure: Sodium 2-chloro-2,2-difluoroacetate (3.05g, 20 mmol) was added in the mixture of corresponding aldehyde (10 mmol) and triphenyl phosphine (5.25 g, 20 mmol) in DMF (20 ml) , the reaction was heated at 100 °C and kepted at this temperature until no further evolution of CO_2 was observed. Then water was added to the reaction slowly and the mixture was extracted with Et_2O, dried over Na_2SO_4. After evaporating solvent at cooled-water bath, the residue was subjected to column chromatography on silica gel to deliver the product.

General procedure for the C-H α-fluoroalkenylation of arenes

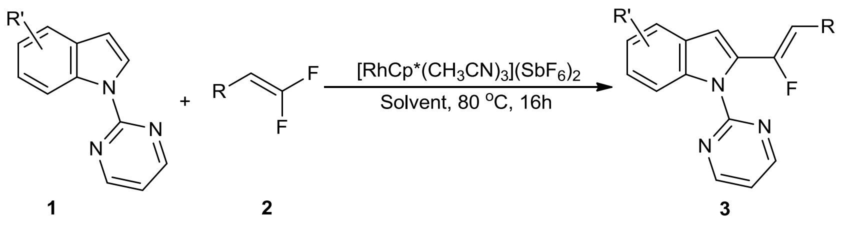

Procedure A: An oven-dried 10 ml Schlenk tube was charged with **1** (0.1 mmol), $[RhCp^*(CH_3CN)_3](SbF_6)_2$ (3.3 mg, 0.004 mmol), **2** (0.15 mmol) in sequence, followed by adding anhydrous MeOH (0.5ml) through syringe and stirring at 80 °C for 16 h, then removal of the solvent in vacuo and purification of the residue by silica gel column chromatography afforded the desired product **3**.

Procedure B: An oven-dried 10 ml Schlenk tube was charged with **1** (0.1 mmol), $[RhCp^*(CH_3CN)_3](SbF_6)_2$ (3.3 mg, 0.004 mmol), **2** (0.15 mmol) in sequence, followed by adding anhydrous CF_3CH_2OH (0.5ml) through syringe and stirring at 80 °C for 16 h, then removal of the solvent in vacuo and purification of the residue by silica gel column chromatography afforded the desired product **3**.

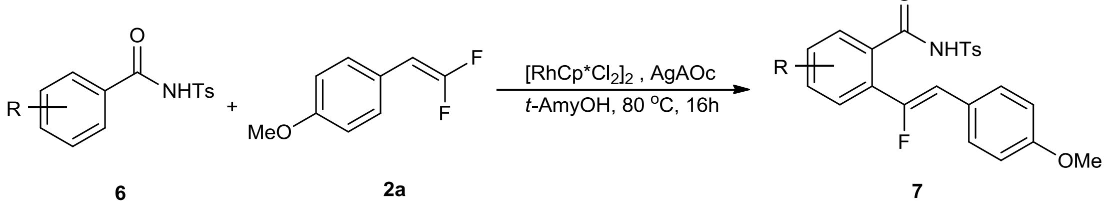

Procedure C: An oven-dried 10 ml Schlenk tube was charged with **6** (0.1 mmol),

[RhCp*Cl2]2 (1.5 mg, 0.0025 mmol), AgOAc (3.3 mg, 0.020 mmol), **2a** (0.15 mmol) in sequence, followed by adding anhydrous *t*-Amyl alcohol (0.5mL) through syringe and stirring at 80 °C for 16 h. The reaction was quenched with water and the resulting solution was extracted with DCM, dried over Na_2SO_4. After removing the solvent in vacuo, the resulted residue was purified through silica gel column chromatography to afford the desired product **7**.

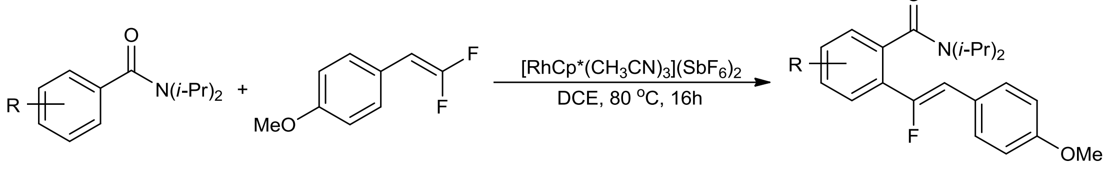

6 **2a** **7**

Procedure D: An oven-dried 10 ml Schlenk tube was charged with **6** (0.1 mmol), $[RhCp^*(CH_3CN)_3](SbF_6)_2$ (3.3 mg, 0.004 mmol), **2a** (0.15 mmol) in sequence, followed by adding anhydrous 1,2-bichloroethane (0.5mL) through syringe and stirring at 80 °C for 16 h, then removal the solvent in vacuo and purification of the resuide by silica gel column chromatography afforded the desired product **7**.

Comptition reaction for pyridine substrates

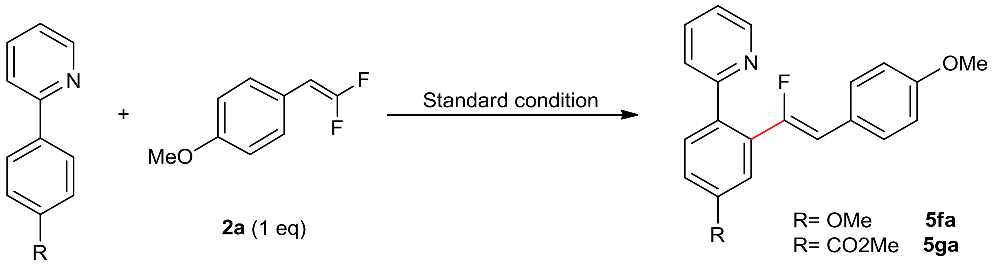

4f/4g (1:1) **5fa/5ga** (2.7:1)

Procedure: To a 10 ml Schlenk tube was successively charged with $[RhCp^*(CH_3CN)_3](SbF_6)_2$ (0.004 mmol), **4f** (0.1 mmol), **4g** (0.1 mmol) and **2a** (0.1 mmol) in sequence, followed by adding anhydrous MeOH (0.5ml) through syringe and stirring at 80 °C for 16 h, Removal of the solvent in vacuo afforded the crude products, the ration of 5fa/5ga was determined by ^{1}H NMR.

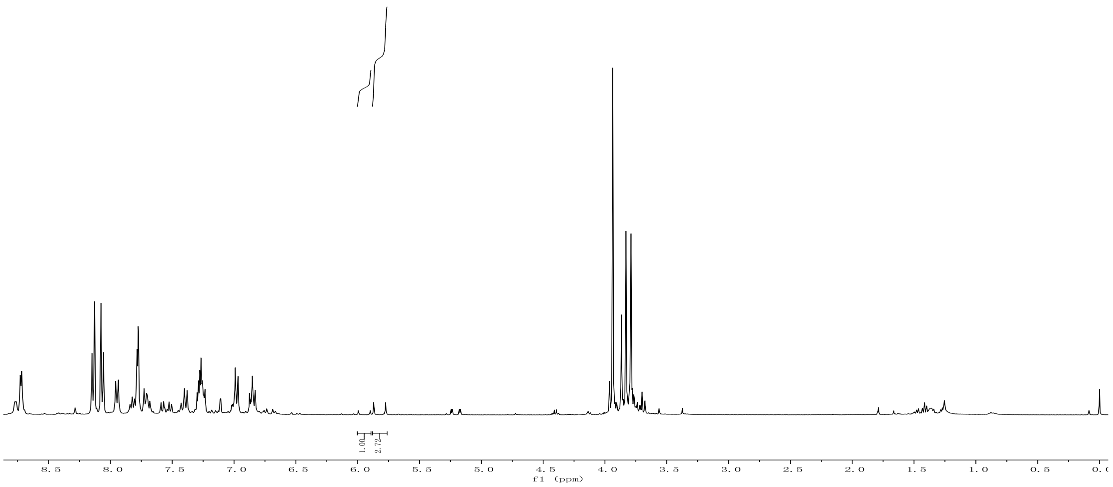

Control experiments

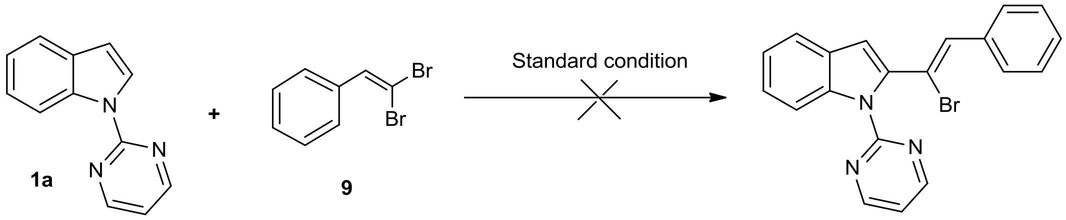

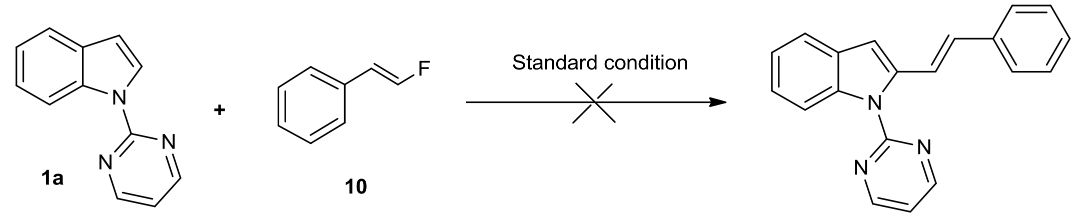

Procedure: To a 10 ml Schlenk tube was successively charged with $[RhCp^*(CH_3CN)_3](SbF_6)_2$ (0.004 mmol), **1a** (0.1 mmol), **9** (0.15 mmol) or **10** (0.15 mmol) in sequence, followed by adding anhydrous MeOH (0.5ml) through syringe and stirring at 80 °C for 16 h, Removal of the solvent in vacuo afforded the crude products, the crude product was analysed by ^1H NMR.

Application for alkyne synthesis

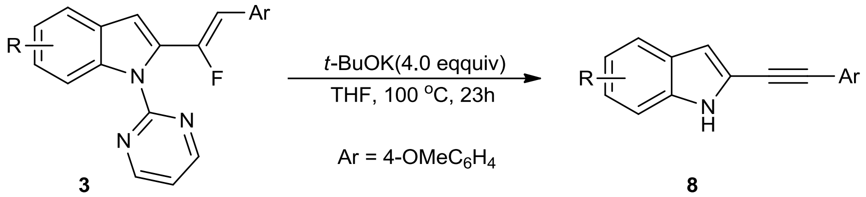

Procedure: To a 10 ml Schlenk tube was charged with *t*-BuOK (68.9 mg, 0.4 mmol) and **3** or **5** (0.1 mmol) followed by adding anhydrous THF (1.0 ml) through syringe and then closed tightly. After stirring at 100 °C for 23 hours, saturated ammonium chloride (2 ml) was added and the resulting mixture was extracted with dichloromethane (2x5 ml). Removal of the solvent in vacuo and purification of the residue by silica gel column chromatography afforded the desired product **8**.

Characterization of structurally novel compounds

(*Z*)-2-(1-Fluoro-2-(4-methoxyphenyl)vinyl)-1-(pyrimidin-2-yl)-1*H*-indole (3aa): Procedure A, white solid, m.p. 128-130. ^{1}H NMR (400 MHz, $CDCl_3$): δ 8.78 (d, *J* = 4.8 Hz, 2H), 8.34 (d, *J* = 8.4 Hz, 1H), 7.63 (d, *J* = 7.7 Hz, 1H), 7.52 - 7.56 (m, 2H), 7.33 (t, *J* = 8.0 Hz, 1H), 7.24 (t, *J* = 7.0 Hz ,1H), 7.16 (t, *J* = 4.7 Hz, 1H), 6.96 (d, *J* = 2.3 Hz, 1H), 6.86 – 6.92 (m, 2H), 6.24 (d, *J* = 36.6 Hz, 1H), 3.82 (s, 3H); ^{19}F NMR (376 MHz, $CDCl_3$): δ -103.7 (dd, J_1 = 36.6 Hz, J_2 = 1.9 Hz, 1F); ^{13}C NMR (100 MHz, $CDCl_3$): δ 158.8 (d, *J* = 2.8 Hz), 158.2, 157.7, 150.7 (d, *J* = 255.2 Hz), 137.5, 132.8, 132.5, 130.2 (d, *J* = 7.7 Hz), 128.6, 126.6 (d, *J* = 4.2 Hz), 124.8, 122.4, 121.1, 117.5, 114.0, 113.9, 110.4 (d, *J* = 5.3 Hz), 108.6 (d, *J* = 10.3 Hz), 55.3; HRMS (ESI) calcd. For $C_{21}H_{16}FN_3O$ $[M+H]^+$: 346.1356, found: 346.1355.

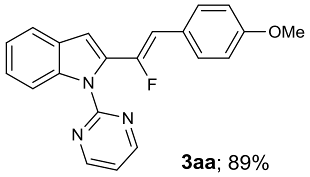

3aa; 89%

(*Z*)-2-(1-Fluoro-2-phenylvinyl)-1-(pyrimidin-2-yl)-1*H*-indole (3ab): Procedure A, white solid, m.p. 107-108. ^{1}H NMR (400 MHz, $CDCl_3$): δ 8.77 (d, *J* = 4.8 Hz, 2H), 8.36 (d, *J* = 8.4 Hz, 1H), 7.64 (d, *J* = 7.8 Hz, 1H), 7.60 (d, *J* = 7.4 Hz, 2H), 7.32-7.39 (m, 3H), 7.22-7.29 (m, 2H), 7.16 (t, *J* = 4.7 Hz, 1H), 7.00 (d, *J* = 2.4 Hz, 1H), 6.28 (d, *J* = 36.3 Hz, 1H); ^{19}F NMR (376 MHz, $CDCl_3$): δ -100.5 (dd, J_1 = 37.4 Hz, J_2 = 2.2 Hz, 1F); ^{13}C NMR (100 MHz, $CDCl_3$): δ 158.3, 157.6, 152.1 (d, *J* = 258.8 Hz), 137.6, 133.9 (d, *J* = 4.3 Hz), 132.5, 132.2, 128.9 (d, *J* = 7.6 Hz), 128.6, 128.5, 127.3 (d, *J* = 2.2 Hz), 124.9, 122.5, 121.2, 117.5, 114.0, 110.9 (d, *J* = 5.3 Hz), 109.0 (d, *J* = 10.0 Hz); HRMS (ESI) calcd. For $C_{20}H_{14}FN_3$ $[M+Na]^+$: 338.1069, found: 338.1068.

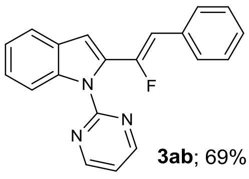

3ab; 69%

(*Z*)-2-(1-Fluoro-2-(p-tolyl)vinyl)-1-(pyrimidin-2-yl)-1*H*-indole (3ac): Procedure A, colorless oil. ^{1}H NMR (400 **MHz, $CDCl_3$):** δ 8.75 (d, *J* = 4.8 Hz, 2H), 8.34 (d, *J* = 8.4 Hz, 1H), 7.63 (d, *J* = 7.8 Hz, 1H), 7.48 (d, *J* = 7.6 Hz, 2H), 7.33 (t, *J* = 7.4 Hz, 1H), 7.24 (t, *J* = 6.8 Hz, 1H), 7.11-7.18 (m, 3H), 6.97 (d, *J* = 2.4 Hz, 1H), 6.24 (d, *J* = 36.1 Hz, 1H), 2.35 (s, 3H); **^{19}F NMR (376 MHz, $CDCl_3$):** δ -101.6 (dd, J_1 = 36.1 Hz, J_2 = 2.0 Hz, 1F); **^{13}C NMR (100 MHz, $CDCl_3$):** δ 158.2, 157.7, 151.5 (d, *J* = 258.1 Hz), 137.6, 137.2 (d, *J* = 2.3 Hz), 132.7, 132.4, 131.0 (d, *J* = 4.2 Hz), 129.3, 128.8 (d, *J* = 7.6 Hz), 128.5, 124.9, 122.4, 121.2, 117.5, 114.0, 110.7 (d, *J* = 5.3 Hz), 109.0 (d, *J* = 10.3 Hz), 21.3; HRMS (ESI) calcd. For $C_{21}H_{16}FN_3$ $[M+H]^+$: 330.1407, found: 330.1407.

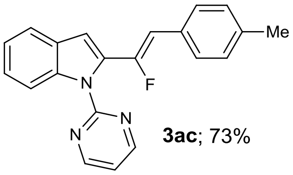

3ac; 73%

(*Z*)-2-(1-Fluoro-2-(m-tolyl)vinyl)-1-(pyrimidin-2-yl)-1*H*-indole (3ad): Procedure A, white solid, m.p. 119-121. ^{1}H NMR (400 MHz, $CDCl_3$): δ 8.76 (d, *J* = 4.8 Hz, 2H), 8.35 (d, *J* = 8.4 Hz, 1H), 7.63 (d, *J* = 7.8 Hz, 1H), 7.40 (d, *J* = 8.4 Hz, 2H), 7.34 (t, *J* = 8.8 Hz, 1H), 7.25 (t, *J* = 7.8 Hz, 2H), 7.14 (t, *J* = 5.3 Hz, 1H), 7.07 (d, *J* = 7.5 Hz, 1H), 6.98 (d, *J* = 2.1

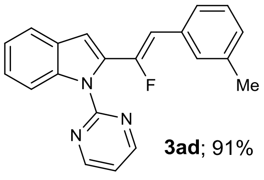

3ad; 91%

Hz, 1H), 6.24 (d, J = 36.5 Hz, 1H), 2.36 (s, 3H); ^{19}F NMR (376 MHz, $CDCl_3$): δ -100.5 (dd, J_1 = 36.5 Hz, J_2 = 2.4 Hz, 1F); ^{13}C NMR (100 MHz, $CDCl_3$): δ 158.3, 157.7, 151.9 (d, J = 257.9 Hz), 138.1, 137.6, 133.8 (d, J = 4.3 Hz), 132.7, 132.4, 129.5 (d, J = 7.5 Hz), 128.5, 128.4, 128.1 (d, J = 2.2 Hz), 126.0 (d, J = 7.6 Hz), 124.9, 122.5, 121.2, 117.5, 114.0, 110.8 (d, J = 5.3 Hz), 109.1 (d, J = 10.0 Hz), 21.5; HRMS (ESI) calcd. For $C_{21}H_{16}FN_3$ [M+H]$^+$: 330.1407, found: 330.1410.

(*Z*)-2-(1-Fluoro-2-(2-methoxyphenyl)vinyl)-1-(pyrimidin-2-yl)-1*H*-indole (3ae):

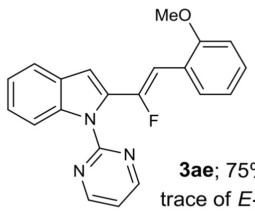

3ae; 75%
trace of *E*-product

Procedure A, white solid, m.p. 94-96. ^{1}H NMR (400 MHz, $CDCl_3$): δ 8.79 (d, J = 4.8 Hz, 2H), 8.34 (d, J = 8.3 Hz, 1H), 7.88 (dd, J_1 = 7.8 Hz, J_2 = 1.7 Hz, 1H), 7.64 (d, J = 7.7 Hz, 1H), 7.34 (t, J = 9.0 Hz, 1H), 7.21-7.27 (m, 2H), 7.16 (t, J = 5.2 Hz,1H), 7.02 (d, J = 2.3 Hz, 1H), 6.96 (t, J = 7.4 Hz,1H), 6.90 (d, J = 8.3 Hz, 1H), 6.72 (d, J = 37.9 Hz, 1H), 3.86 (s, 3H); ^{19}F NMR (376 MHz, $CDCl_3$): δ -102.4 (dd, J_1 = 37.9 Hz, J_2 = 1.8 Hz, 1F); ^{13}C NMR (100 MHz, $CDCl_3$): δ 158.2, 158.0, 157.7, 156.4, 152.0 (d, J = 259.0 Hz), 137.6, 133.0, 132.7, 129.7 (d, J = 13.6 Hz), 128.5 (d, J = 12.4 Hz), 124.7, 122.7 (d, J = 11.6 Hz), 122.4, 121.2, 120.6, 117.5, 113.9, 110.7 (d, J = 5.2 Hz), 110.5, 102.5 (d, J = 8.2 Hz), 55.5; HRMS (ESI) calcd. For $C_{21}H_{16}FN_3O$ [M+H]$^+$: 346.1356 , found: 346.1358.

(*Z*)-4-(2-Fluoro-2-(1-(pyrimidin-2-yl)-1*H*-indol-2-yl)vinyl)-*N*,*N*-dimethylaniline

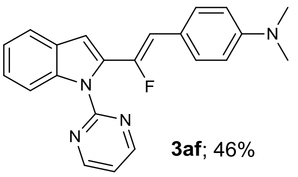

3af; 46%

(3af): Procedure A, colorless oil. ^{1}H NMR (400 MHz, $CDCl_3$): δ 8.77 (d, J = 4.8 Hz, 2H), 8.30 (d, J = 8.4 Hz, 1H), 7.62 (d, J = 7.8 Hz, 1H), 7.48 (d, J = 8.9 Hz, 2H), 7.31 (t, J = 7.5 Hz, 1H), 7.23 (t, J = 7.4 Hz, 1H), 7.14 (t, J = 4.8 Hz, 1H), 6.92 (d, J = 2.2 Hz, 1H), 6.69 (d, J = 8.9 Hz, 2H), 6.18 (d, J = 37.4 Hz, 1H), 2.97 (s, 6H); ^{19}F NMR (376 MHz, $CDCl_3$): δ -106.8 (dd, J_1 = 37.4 Hz, J_2 = 2.0 Hz, 1F); ^{13}C NMR (100 MHz, $CDCl_3$): δ 158.2, 158.1, 157.8, 149.6 (d, J = 2.3 Hz), 149.5 (d, J = 250.9 Hz), 137.5, 133.3, 133.0, 130.0 (d, J = 7.6 Hz), 128.7, 124.5, 122.3, 122.2 (d, J = 4.2 Hz), 120.9, 117.5, 113.7, 112.2, 109.8 (d, J = 5.2Hz), 109.3 (d, J = 10.6 Hz), 40.4; HRMS (ESI) calcd. For $C_{22}H_{19}FN_4$ [M+H]$^+$: 359.1672, found: 359.1673.

(*Z*)-2-(1-Fluoro-2-(4-(trifluoromethyl)phenyl)vinyl)-1-(pyrimidin-2-yl)-1*H*-indole

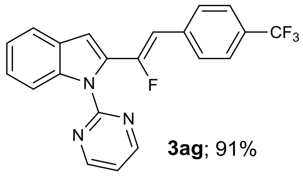

3ag; 91%

(3ag): Procedure A, white solid, m.p. 100-102. ^{1}H NMR (400 MHz, $CDCl_3$): δ 8.76 (d, J = 4.8 Hz, 2H), 8.40 (d, J = 8.4 Hz, 1H), 7.63-7.69 (m, 3H), 7.60 (s, 1H), 7.58 (s, 1H), 7.37 (t, J = 7.8 Hz,1H), 7.26 (t, J = 7.9 Hz, 1H), 7.02 (d, J = 2.3 Hz, 1H), 6.30 (d, J = 35.3 Hz, 1H); ^{19}F NMR (376 MHz, $CDCl_3$): δ -62.5 (s, 3F), -97.0 (d, J = 35.3 Hz, 1F); ^{13}C NMR (100 MHz, $CDCl_3$): δ 158.3, 158.1, 157.5, 153.7 (d, J = 260.7 Hz), 137.7, 137.4, 131.8, 131.6, 128.9 (d, J = 8.0 Hz), 128.4, 125.5 (m), 122.7, 121.4, 117.6, 114.3, 111.7 (d, J = 5.5 Hz), 107.7 (d, J = 9.9 Hz); HRMS (ESI) calcd. For $C_{21}H_{13}F_4N_3$ [M+H]$^+$: 384.1124, found: 384.1115.

Methyl-(*Z*)-4-(2-fluoro-2-(1-(pyrimidin-2-yl)-1*H*-indol-2-yl)vinyl)benzoate (3ah):

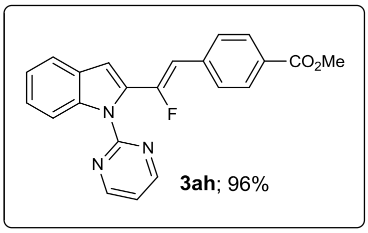

Procedure A, white solid, m.p. 145-147. ^{1}H NMR (400 MHz, $CDCl_3$): δ 8.76 (d, *J* = 4.8 Hz, 2H), 8.43 (d, *J* = 8.4 Hz, 1H), 8.01 (d, *J* = 8.4 Hz, 2H), 7.61-7.68 (m, 3H), 7.37 (t, *J* = 8.2 Hz, 1H), 7.26 (t, *J* = 7.7 Hz,1H), 7.15 (t, *J* = 4.8 Hz, 1H), 7.02 (d, *J* = 2.1 Hz, 1H), 6.31 (d, *J* = 35.6 Hz, 1H), 3.90 (s, 3H); ^{19}F NMR (376 MHz, $CDCl_3$): δ -96.6 (dd, J_1 = 35.6 Hz, J_2 = 2.1 Hz, 1F); ^{13}C NMR (100 MHz, $CDCl_3$): δ 166.8, 158.2, 157.5, 153.7 (d, *J* = 262.8Hz), 138.5 (d, *J* = 4.4 Hz), 137.7, 131.9, 131.7, 129.8, 128.6 (d, *J* = 7.9 Hz), 128.5 (d, *J* = 2.4 Hz), 128.4, 125.3, 122.6, 121.4, 117.6, 114.2, 111.6 (d, *J* =5.5 Hz), 108.2 (d, *J* = 9.8 Hz), 52.1; HRMS (ESI) calcd. For $C_{22}H_{16}FN_3O_2$ $[M+H]^+$: 374.1305, found: 374.1297.

(*Z*)-1-(4-(2-Fluoro-2-(1-(pyrimidin-2-yl)-1*H*-indol-2-yl)vinyl)phenyl)ethan-1-one

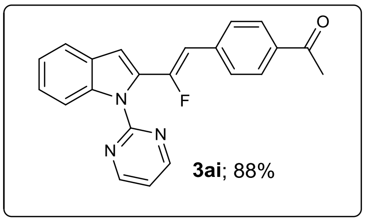

(3ai): Procedure A, white solid, m.p. 143-145. ^{1}H NMR (400 MHz, $CDCl_3$): δ 8.77 (d, *J* = 4.8 Hz, 2H), 8.39 (d, *J* = 8.4 Hz, 1H), 794 (d, *J* = 8.4 Hz, 2H), 7.62-7.69 (m, 3H), 7.37 (t, *J* = 7.5 Hz, 1H), 7.26 (t, *J* = 7.8 Hz, 1H), 7.17 (t, *J* = 4.5 Hz, 1H), 7.04 (d, *J* = 2.2 Hz, 1H), 6.23 (d, *J* = 35.6 Hz, 1H), 2.60 (s, 3H); ^{19}F NMR (376 MHz, $CDCl_3$): δ -96.3 (dd, J_1 = 35.6 Hz, J_2 = 2.2 Hz, 1F); ^{13}C NMR (100 MHz, $CDCl_3$): δ 197.5, 158.2, 158.1, 157.5, 153.8 (d, *J* = 263.8 Hz), 138.7 (d, *J* = 4.4 Hz), 137.8, 135.5, 131.9, 131.7, 128.8 (d, *J* = 8.0 Hz), 128.6, 128.4, 125.3, 122.7, 121.4, 117.6, 114.2, 111.7 (d, *J* = 5.5 Hz), 108.1 (d, *J* = 9.8 Hz), 26.6; HRMS (ESI) calcd. For $C_{22}H_{16}FN_3O$ $[M+H]^+$: 358.1356, found: 358.1353.

(*Z*)-2-(1-Fluoro-2-(4-nitrophenyl)vinyl)-1-(pyrimidin-2-yl)-1*H*-indole (3aj):

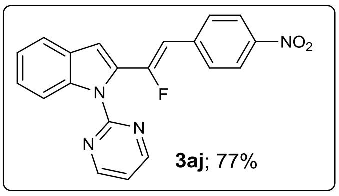

Procedure A, yellow solid, m.p. 137-138. ^{1}H NMR (400 MHz, $CDCl_3$): δ 8.79 (d, *J* = 4.8 Hz, 2H), 8.42 (dd, J_1 = 8.5 Hz, J_2 = 0.8 Hz, 1H), 8.20 (d, *J* = 8.9 Hz, 2H), 7.71 (d, *J* = 8.9 Hz, 2H), 7.66 (d, *J* = 7.8 Hz, 1H), 7.40 (td, J_1 = 7.2 Hz, J_2 = 1.1 Hz, 1H), 7.28 (td, J_1 = 7.2 Hz, J_2 = 1.1 Hz, 1H), 7.20 (t, *J* = 4.8 Hz, 1H), 7.07 (d, *J* = 2.5 Hz, 1H), 6.35 (d, *J* = 34.8 Hz, 1H); ^{19}F NMR (376 MHz, $CDCl_3$): δ -94.0 (dd, J_1 = 34.8 Hz, J_2 = 1.6 Hz, 1F); ^{13}C NMR (100 MHz, $CDCl_3$): δ 158.3, 157.4, 154.9 (d, *J* = 265.6 Hz), 146.2 (d, *J* = 3.1 Hz), 140.5 (d, *J* = 4.4 Hz), 137.8, 131.4, 131.1, 129.2 (d, *J* = 8.4 Hz), 128.3, 125.6, 123.9, 122.8, 121.5, 117.7, 114.4, 112.3 (d, *J* = 5.7 Hz), 107.1 (d, *J* = 9.6 Hz); HRMS (ESI) calcd. For $C_{20}H_{13}FN_4O_2$ $[M+H]^+$: 361.1101, found: 361.1104.

(*Z*)-4-(2-Fluoro-2-(1-(pyrimidin-2-yl)-1*H*-indol-2-yl)vinyl)benzonitrile (3ak):

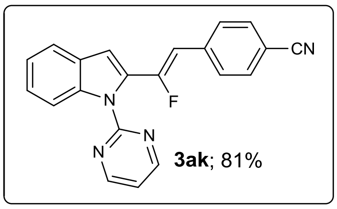

Procedure A, white solid, m.p. 120-121. ^{1}H NMR (400 MHz, $CDCl_3$): δ 8.78 (d, *J* = 4.8 Hz, 2H), 8.41 (d, *J* = 8.4 Hz, 1H), 7.59-7.70 (m, 5H), 7.39 (t, *J* = 7.5 Hz, 1H), 7.29 (d, *J* = 7.6 Hz, 1H), 7.19 (t, *J* = 4.8 Hz, 1H), 7.04 (d, *J* = 2.0 Hz, 1H), 6.28 (d, *J* = 35.0 Hz, 1H); ^{19}F NMR (376 MHz, $CDCl_3$): δ -94.9 (dd, J_1 = 35.0 Hz, J_2 = 1.3 Hz, 1F); ^{13}C NMR (100 MHz, $CDCl_3$): δ 158.3, 157.4, 154.5 (d, *J* = 262.4 Hz), 138.5 (d, *J* =

4.4 Hz), 137.7, 132.3, 131.5, 131.2, 129.1 (d, J = 8.3 Hz), 128.3, 125.5, 122.8, 121.4, 119.0, 117.6, 114.3, 112.0 (d, J = 5.6 Hz), 110.3 (d, J = 2.9 Hz), 107.4 (d, J = 9.7 Hz); HRMS (ESI) calcd. For $C_{21}H_{13}FN_4$ $[M+H]^+$: 341.1202, found: 341.1192.

(*Z*)-2-(1-Fluoro-2-(4-fluorophenyl)vinyl)-1-(pyrimidin-2-yl)-1*H*-indole (3al):

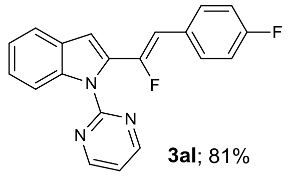
3al; 81%

Procedure A, colorless oil. ^{1}H NMR (400 MHz, $CDCl_3$): δ 8.78 (d, J = 4.8 Hz, 2H), 8.37 (d, J = 8.4 Hz, 1H), 7.64 (d, J = 8.2 Hz, 1H), 7.55-7.60 (m, 2H), 7.36 (t, J = 7.2 Hz, 1H), 7.26 (t, J = 8.2 Hz, 1H), 7.18 (t, J = 5.0 Hz, 1H), 7.05 (t, J = 8.9 Hz, 2H), 6.98 (d, J = 2.4 Hz, 1H), 6.25 (d, J = 35.9 Hz, 1H); ^{19}F NMR (376 MHz, $CDCl_3$): δ -101.5 (d, J = 35.9 Hz, 1F), -113.8 (m, 1F); ^{13}C NMR (100 MHz, $CDCl_3$): δ 163.0 (d, J = 3.4 Hz), 160.6 (d, J = 3.3 Hz), 158.2, 157.6, 151.7 (dd, J_1 = 256.6 Hz, J_2 = 2.6 Hz), 137.6, 132.3, 132.0, 130.5(t), 130.0 (t), 128.5, 125.0, 122.5, 121.2, 117.6, 115.6, 115.4, 114.1, 111.0 (d, J = 5.4 Hz), 107.9 (d, J = 10.3 Hz); HRMS (ESI) calcd. For $C_{20}H_{13}F_2N_3$ $[M+H]^+$: 341.1156, found: 341.1157.

(*Z*)-2-(2-(3-Chlorophenyl)-1-fluorovinyl)-1-(pyrimidin-2-yl)-1*H*-indole (3am):

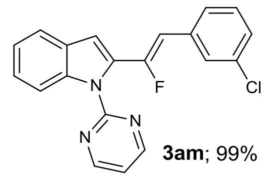
3am; 99%

Procedure A, white solid, m.p. 123-125. ^{1}H NMR (400 MHz, $CDCl_3$): δ 8.77 (d, J = 4.8 Hz, 2H), 8.38 (d, J = 8.4 Hz, 1H), 7.64 (d, J = 7.8 Hz, 1H), 7.62 (s, 1H), 7.43 (d, J = 7.5 Hz, 1H), 7.36 (t, J = 7.8 Hz, 1H), 7.20-7.30 (m, 3H), 7.15 (t, J = 4.6 Hz, 1H), 6.98 (d, J = 2.4 Hz, 1H), 6.22 (d, J = 35.4 Hz, 1H); ^{19}F NMR (376 MHz, $CDCl_3$): δ -98.1 (dd, J_1 = 35.4 Hz, J_2 = 2.0 Hz, 1F); ^{13}C NMR (100 MHz, $CDCl_3$): δ 158.3, 157.5, 153.1(d, J = 259.9 Hz), 137.7, 135.6 (d, J = 4.2 Hz), 134.4, 131.9, 131.7, 129.7, 128.6 (d, J = 8.8 Hz), 128.4, 127.3 (d, J = 2.1 Hz), 127.0 (d, J = 7.2 Hz), 125.2, 122.6, 121.3, 117.6, 114.2, 111.3 (d, J = 5.5 Hz), 107.8 (d, J = 9.9 Hz); HRMS (ESI) calcd. For $C_{20}H_{13}ClFN_3$ $[M+Na]^+$: 372.0680, found: 372.0680.

(*Z*)-2-(2-(4-Bromophenyl)-1-fluorovinyl)-1-(pyrimidin-2-yl)-1*H*-indole (3an):

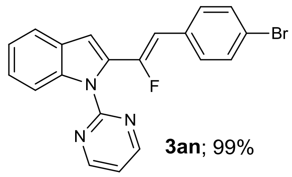
3an; 99%

Procedure A, white solid, m.p. 112-114. ^{1}H NMR (400 MHz, $CDCl_3$): δ 8.77 (d, J = 4.8 Hz, 2H), 8.37 (d, J = 8.3 Hz, 1H), 7.65 (d, J = 7.8 Hz, 1H), 7.43-7.50 (m, 4H), 7.36 (t, J = 8.4 Hz,1H), 7.26 (t, J = 7.8 Hz, 1H), 7.17 (t, J = 5.3 Hz, 1H), 7.00 (d, J = 2.0 Hz, 1H), 6.22 (d, J = 35.7 Hz, 1H); ^{19}F NMR (376 MHz, $CDCl_3$): δ -99.1 (dd, J_1 = 35.7 Hz, J_2 = 2.1 Hz, 1F); ^{13}C NMR(100 MHz, $CDCl_3$): δ 158.3, 157.6, 152.5 (d, J = 258.2 Hz), 137.6, 132.8 (d, J = 4.2 Hz), 132.1, 131.9, 131.7, 131.4, 130.3 (d, J = 8.0 Hz), 128.4, 125.2, 122.6, 121.3, 121.1 (d, J = 3.4 Hz), 117.6, 114.1, 111.2 (d, J = 5.4 Hz), 107.9 (d, J = 10.1 Hz); HRMS (ESI) calcd. For $C_{20}H_{13}BrFN_3$ $[M+H]^+$: 394.0355, found: 394.0336.

(*Z*)-2-(1-Fluoro-2-(2-fluorophenyl)vinyl)-1-(pyrimidin-2-yl)-1*H*-indole (3ao):

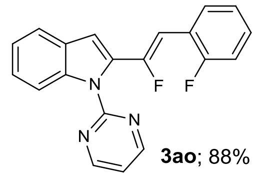
3ao; 88%

Procedure A, white solid, m.p. 136-138. ^{1}H NMR (400 MHz, $CDCl_3$): δ 8.68 (d, J = 4.8 Hz, 2H), 8.29 (d, J = 8.4 Hz, 1H), 7.82 (t, J = 7.8 Hz, 1H), 7.56 (d, J = 7.7 Hz, 1H), 7.27 (t, J = 7.7 Hz, 1H), 7.10-7.20 (m, 2H), 694-7.08 (m, 4H), 6.43 (d, J = 36.2 Hz, 1H); ^{19}F NMR (376 MHz,

$CDCl_3$): δ -98.9 (d, J = 35.7 Hz, 1F), -116.5(m, 1F); ^{13}C NMR (100 MHz, $CDCl_3$): δ 160.9, 158.5, 158.2, 157.6, 153.2 (d, J = 258.4 Hz), 137.7, 131.9, 129.9 (dd, J_1 = 13.7 Hz, J_2 = 2.5 Hz), 128.7 (dd, J_1 = 8.4 Hz, J_2 = 1.9 Hz), 128.5, 125.1, 124.1 (d, J = 3.6 Hz), 122.6, 121.7 (dd, J_1 = 11.9 Hz, J_2 = 4.4 Hz), 121.3, 117.6, 115.4, 115.1, 114.1, 111.4 (d, J = 5.4 Hz), 100.3 (dd, J_1 = 9.2 Hz, J_2 = 7.5 Hz); HRMS (ESI) calcd. For $C_{20}H_{13}F_2N_3$ $[M+H]^+$: 334.1156, found: 334.1152.

(*Z*)-2-(1-Fluoro-2-(4-iodophenyl)vinyl)-1-(pyrimidin-2-yl)-1*H*-indole (3ap):

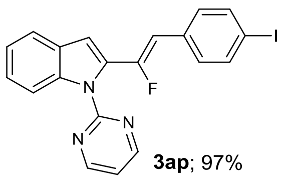

3ap; 97%

Procedure A, white solid, m.p. 130-132. ^1H NMR (400 MHz, $CDCl_3$): δ 8.76 (d, J = 4.8 Hz, 2H), 8.37 (dd, J_1 = 8.4 Hz, J_2 = 0.8 Hz, 1H), 7.67 (d, J = 8.5 Hz, 2H), 7.64 (d, J = 7.7 Hz, 1H), 7.36 (t, J = 8.1 Hz, 1H), 7.32(d, J = 8.5 Hz, 2H), 7.23-7.28 (m, 1H), 7.15 (t, J = 4.8 Hz,1H), 6.99 (d, J = 2.0 Hz,1H), 6.19 (d, J = 35.7 Hz,1H); ^{19}F NMR (376 MHz, $CDCl_3$): δ -98.6 (dd, J_1 = 35.7 Hz, J_2 = 2.1 Hz, 1F); ^{13}C NMR (100 MHz, $CDCl_3$): δ 158.2, 157.5, 152.8 (d, J = 260.4 Hz), 137.6, 133.3 (d, J = 4.3 Hz), 132.1, 131.9, 130.5 (d, J = 7.9 Hz), 128.4, 125.2, 122.6, 121.3, 117.6, 114.2, 111.3 (d, J = 5.5 Hz), 108.0 (d, J = 10.0 Hz), 92.6 (d, J = 3.3 Hz); HRMS (ESI) calcd. For $C_{20}H_{13}FIN_3$ $[M+H]^+$: 442.0216, found:442.0233.

(*Z*)-2-(2-(Benzo[d][1,3]dioxol-5-yl)-1-fluorovinyl)-1-(pyrimidin-2-yl)-1*H*-indole

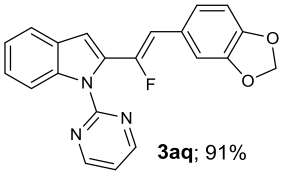

3aq; 91%

(3aq): Procedure A, white solid, m.p. 104-106. ^1H NMR (400 MHz, $CDCl_3$): δ 8.78 (d, J = 4.8 Hz, 2H), 8.34 (dd, J_1 = 8.4 Hz, J_2 = 0.8 Hz, 1H), 7.63 (d, J = 7.7 Hz, 1H), 7.34 (t, J = 7.2 Hz,1H), 7.21-7.28 (m, 2H), 7.17 (t, J = 4.6 Hz, 1H), 6.99 (dd, J_1 = 8.1 Hz, J_2 = 1.6 Hz, 1H), 6.96 (dd, J_1 = 2.5 Hz, J_2 = 0.6 Hz, 1H), 6.80 (d, J = 8.1 Hz, 1H), 6.20 (d, J = 35.9 Hz, 1H), 5.7 (s, 2H); ^{19}F NMR (376 MHz, $CDCl_3$): δ -102.6 (dd, J_1 = 35.9 Hz, J_2 = 2.1 Hz, 1F); ^{13}C NMR (100 MHz, $CDCl_3$): δ 158.2, 158.1, 157.6, 150.9 (d, J = 257.2 Hz), 147.8, 146.8, 137.5, 132.5, 132.3, 128.5, 128.0 (d, J = 4.3 Hz), 124.8, 123.2 (d, J = 6.9 Hz), 122.5, 121.1, 117.5, 114.0, 110.6 (d, J = 5.3 Hz), 108.8 (d, J = 10.2 Hz), 108.3; HRMS (ESI) calcd. For $C_{21}H_{14}FN_3O_2$ $[M+H]^+$: 360.1148, found: 360.1140.

(*Z*)-2-(1-Fluoro-2-(naphthalen-2-yl)vinyl)-1-(pyrimidin-2-yl)-1*H*-indole (3ar):

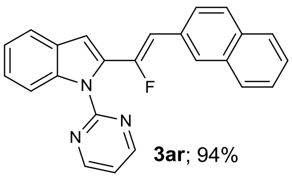

3ar; 94%

Procedure A, colorless oil. ^1H NMR (400 MHz, $CDCl_3$): δ 8.77 (d, J = 4.8 Hz, 2H), 8.38 (d, J = 8.4 Hz, 1H), 8.03 (s, 1H), 7.72-7.84 (m, 4H), 7.65 (d, J = 7.7 Hz, 1H), 7.42-7.48 (m, 2H), 7.36 (t, J = 7.7 Hz, 1H), 7.26 (t, J = 7.7 Hz, 1H), 7.14 (t, J = 5.1 Hz, 1H), 7.04 (d, J = 2.2 Hz, 1H), 6.44 (d, J = 36.3 Hz, 1H); ^{19}F NMR (376 MHz, $CDCl_3$): δ -100.1 (dd, J_1 = 36.3 Hz, J_2 = 2.1 Hz, 1F); ^{13}C NMR (100 MHz, $CDCl_3$): δ 158.3, 157.7, 152.4 (d, J = 260.3 Hz), 137.7, 133.5, 132.6, 131.4 (d, J = 4.4 Hz), 128.5, 128.1, 128.0, 127.9, 127.6, 126.8 (d, J = 7.7 Hz), 126.1 (d, J = 14.9 Hz), 125.0, 122.5, 121.3, 117.6, 114.1, 111.0 (d, J = 5.4 Hz), 109.2 (d, J =9.9 Hz); HRMS (ESI) calcd. For $C_{24}H_{16}FN_3$ $[M+H]^+$: 366.1407, found: 366.1407.

(*Z*)-2-(1-Fluoro-2-(thiophen-2-yl)vinyl)-1-(pyrimidin-2-yl)-1*H*-indole (3as):

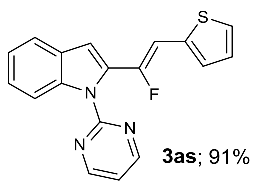

3as; 91%

Procedure A, colorless oil. ^{1}H NMR (400 MHz, $CDCl_3$): δ 8.75 (d, *J* = 4.8 Hz, 2H), 8.36 (d, *J* = 8.4 Hz, 1H), 7.62 (d, *J* = 7.8 Hz, 1H), 7.35 (t, *J* = 7.5 Hz,1H), 7.29 (d, *J* = 5.0 Hz, 1H), 7.25 (t, *J* = 7.5 Hz, 1H), 7.16 (d, *J* = 3.2 Hz, 1H), 7.13 (t, *J* = 4.6 Hz, 1H), 7.01-7.05 (m, 1H), 6.93 (d, *J* = 2.4 Hz, 1H), 6.58 (d, *J* = 35.1 Hz, 1H); ^{19}F NMR (376 MHz, $CDCl_3$): δ -98.3(d, *J* = 35.1 Hz, 1F); ^{13}C NMR (100 MHz, $CDCl_3$): δ 158.2, 157.6, 150.6(d, *J* = 256.3 Hz), 137.7, 136.2 (d, *J* = 5.8 Hz), 131.7, 131.4, 128.5, 127.2 (d, *J* = 3.9 Hz), 126.9, 126.0 (d, *J* = 9.3 Hz), 125.0, 122.6, 121.2, 117.6, 114.1, 110.8 (d, *J* = 5.3 Hz), 103.6 (d, *J* = 14.4 Hz); HRMS (ESI) calcd. For $C_{18}H_{12}FN_3S$ $[M+H]^+$: 322.0814, found: 322.0811.

(*Z*)-2-(2-(Benzo[b]thiophen-2-yl)-1-fluorovinyl)-1-(pyrimidin-2-yl)-1*H*-indole

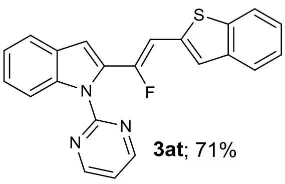

3at; 71%

(3at): Procedure A, white solid, m.p. 183-184. ^{1}H NMR (400 MHz, $CDCl_3$): δ 8.76 (d, *J* = 4.8 Hz, 2H), 8.39 (d, *J* = 8.4 Hz, 1H), 7.78 (d, *J* = 8.5 Hz, 1H), 7.73 (dd, J_1 = 6.7 Hz, J_2 = 1.4 Hz, 1H), 7.64 (d, *J* = 7.8 Hz, 1H), 7.22-7.39 (m, 5H), 7.15 (t, *J* = 4.9 Hz, 1H), 7.02 (d, *J* = 1.8 Hz, 1H), 6.64 (d, *J* = 34.7 Hz, 1H); ^{19}F NMR (376 MHz, $CDCl_3$): δ -96.3 (d, *J* = 34.7Hz, 1F); ^{13}C NMR (100 MHz, $CDCl_3$): δ 158.3, 157.5, 152.1 (d, *J* = 259.9 Hz), 140.3 (d, *J* = 7.7 Hz), 139.3, 137.8, 136.4 (d, *J* = 6.2 Hz), 131.4, 131.2, 128.5, 125.3, 124.4 (d, *J* = 6.7 Hz), 123.6 (d, *J* = 3.2 Hz), 123.3 (d, *J* = 1.5 Hz), 122.6, 122.1, 121.4, 117.6, 115.1, 114.2, 111.4 (d, *J* = 5.4 Hz), 103.9 (d, *J* = 13.7 Hz); HRMS (ESI) calcd. For $C_{22}H_{14}FN_3S$ $[M+H]^+$: 372.0971, found: 372.0965.

(*Z*)-2-(1-Fluoro-4-phenylbut-1-en-1-yl)-1-(pyrimidin-2-yl)-1*H*-indole (3au):

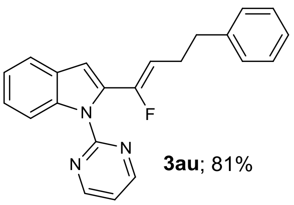

3au; 81%

Procedure A, white solid, m.p. 79-81. ^{1}H NMR (400 MHz, $CDCl_3$): δ 8.73 (d, *J* = 4.8 Hz, 2H), 8.31 (d, *J* = 8.4 Hz, 1H), 7.59 (d, *J* = 7.7 Hz, 1H), 7.17-7.34 (m, 7H), 7.11 (t, *J* = 4.6 Hz, 1H), 6.79 (d, *J* = 2.5 Hz, 1H), 5.34 (dt, J_1 = 34.5 Hz, J_2 = 7.4 Hz, 1H), 2.81 (t, *J* = 7.3 Hz, 2H), 2.59 (q, *J* = 7.9 Hz, 2H); ^{19}F NMR (376 MHz, $CDCl_3$): δ -105.9 (dd, J_1 = 34.5 Hz, J_2 = 1.0 Hz, 1F); ^{13}C NMR (100 MHz, $CDCl_3$): δ 158.1, 157.6, 151.7 (d, *J* = 246.6 Hz), 141.6, 137.3, 132.2, 131.9, 128.4 (d, *J* = 15.0 Hz), 125.9, 124.6, 122.3, 121.0, 117.4, 114.0, 110.4 (d, *J* = 5.3 Hz), 108.8 (d, *J* = 17 Hz), 35.4 (d, *J* = 1.5 Hz), 26.0 (d, *J* = 2.4 Hz); HRMS (ESI) calcd. For $C_{22}H_{18}FN_3$ $[M+H]^+$: 344.1563, found: 344.1562.

(*Z*)-2-(1-Fluoro-2-(4-methoxyphenyl)vinyl)-3-methyl-1-(pyrimidin-2-yl)-1*H*-indole (3ba):

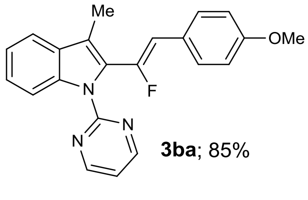

3ba; 85%

Procedure A, white solid, m.p. 145-147. ^{1}H NMR (400 MHz, $CDCl_3$): δ 8.71 (d, *J* = 4.8 Hz, 2H), 8.42 (d, *J* = 8.4 Hz, 1H), 7.61 (d, *J* = 7.8 Hz, 1H), 7.57 (d, *J* = 8.8 Hz, 1H), 7.36 (t, *J* = 8.4 Hz,1H), 7.26 (t, *J* = 7.7 Hz, 1H), 7.06 (t, *J* = 5.7 Hz, 1H), 6.90 (d, *J* = 8.8 Hz, 2H), 5.98 (d, *J* = 37.0 Hz, 1H), 3.82 (s, 3H), 2.47 (d, *J* = 2.7 Hz, 3H); ^{19}F NMR (376 MHz, $CDCl_3$): δ -98.5 (dd, J_1 = 37.0 Hz, J_2 = 2.3 Hz, 1F);

^{13}C NMR (100 MHz, $CDCl_3$): δ 158.8 (d, J = 2.8 Hz), 158.1, 157.7, 149.1 (d, J = 256.8 Hz), 136.6, 130.2 (d, J = 7.7 Hz), 129.9, 128.6 (d, J = 5.2 Hz), 128.4, 126.7 (d, J = 4.4 Hz), 125.2, 122.0, 119.6, 119.1 (d, J = 5.7 Hz), 116.9, 114.2, 114.0, 111.1 (d, J = 11.3 Hz), 55.3, 10.0; HRMS (ESI) calcd. For $C_{22}H_{18}FN_3O$ $[M+H]^+$: 360.1512, found: 360.1504.

(*Z*)-2-(1-Fluoro-2-(4-methoxyphenyl)vinyl)-4-methyl-1-(pyrimidin-2-yl)-1*H*-indole (3ca): Procedure A, white solid, m.p. 158-159. ^{1}H NMR (400 MHz, $CDCl_3$): δ 8.75 (d, J = 4.8 Hz, 2H), 8.15 (d, J = 8.4 Hz, 1H), 7.53 (d, J = 8.8 Hz, 2H), 7.23 (t, J = 9.4 Hz,1H), 7.12 (t, J = 5.0 Hz,1H), 7.03 (d, J = 7.2 Hz, 1H), 6.99 (d, J = 0.5 Hz, 1H), 6.89 (d, J = 8.8 Hz, 2H), 6.24 (d, J = 36.7 Hz, 1H), 3.81 (s, 3H), 2.57 (s, 3H); ^{19}F NMR (376 MHz, $CDCl_3$): δ -103.5 (dd, J_1 = 36.7 Hz, J_2 = 2.0 Hz, 1F); ^{13}C NMR (100 MHz, $CDCl_3$): δ 158.8, 158.2, 157.7, 150.8 (d, J = 257.1 Hz), 137.4, 132.2, 131.9, 130.6, 130.2 (d, J = 7.7 Hz), 128.3, 126.7 (d, J = 3.6 Hz), 124.9, 122.7, 117.5, 113.9, 111.5, 109.0 (d, J = 5.2 Hz), 108.5 (d, J = 10.6 Hz), 55.3, 18.6; HRMS (ESI) calcd. For $C_{22}H_{18}FN_3O$ $[M+H]^+$: 360.1512, found: 360.1511.

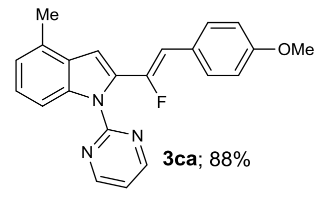

3ca; 88%

(*Z*)-2-(1-Fluoro-2-(4-methoxyphenyl)vinyl)-5-methyl-1-(pyrimidin-2-yl)-1*H*-indole (3da): Procedure A, white solid, m.p. 187-189. ^{1}H NMR (400 MHz, $CDCl_3$): δ 8.75 (d, J = 4.8 Hz, 2H), 8.23 (d, J = 8.6 Hz, 1H), 7.54 (d, J = 8.8 Hz, 2H), 7.40 (s, 1H), 7.15 (dd, J_1 = 8.6 Hz, J_2 = 1.3 Hz, 1H), 7.12 (t, J = 4.6 Hz, 1H), 6.82-6.94 (m, 3H), 6.21 (d, J = 36.6 Hz, 1H), 3.82 (s, 3H), 2.45 (s, 3H); ^{19}F NMR (376 MHz, $CDCl_3$): δ -103.2 (dd, J_1 = 36.6 Hz, J_2 = 2.1 Hz, 1F); ^{13}C NMR (100 MHz, $CDCl_3$): δ 158.8 (d, J = 2.8 Hz), 158.2, 157.7, 150.8 (d, J = 256.5 Hz), 135.9, 132.7, 132.4, 131.8, 130.2 (d, J = 7.7 Hz), 128.8, 126.7 (d, J = 4.2 Hz), 126.3, 120.8, 117.3, 113.9, 113.8, 110.4 (d, J = 5.3 Hz), 108.4 (d, J = 10.5 Hz), 55.3, 21.4; HRMS (ESI) calcd. For $C_{22}H_{18}FN_3O$ $[M+H]^+$: 360.1512, found: 360.1515.

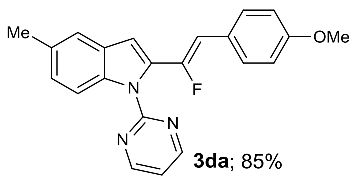

3da; 85%

(*Z*)-2-(1-Fluoro-2-(4-methoxyphenyl)vinyl)-5-methoxy-1-(pyrimidin-2-yl)-1*H*-indole (3ea): Procedure A, white solid, m.p. 173-174. ^{1}H NMR (400 MHz, $CDCl_3$): δ 8.74 (d, J = 4.8 Hz, 2H), 8.28 (d, J = 9.0 Hz, 1H), 7.54 (d, J = 8.8 Hz, 2H), 7.11 (t, J = 5.3 Hz,1H), 7.06 (d, J = 2.5 Hz, 1H), 6.97 (dd, J_1 = 9.1 Hz, J_2 = 2.6 Hz, 1H), 6.87-6.92 (m, 3H), 6.21(d, J = 36.5 Hz, 1H), 3.86 (s, 3H), 3.82 (s, 3H); ^{19}F NMR (376 MHz, $CDCl_3$): δ -103.1 (dd, J_1 = 36.5 Hz, J_2 = 2.0 Hz, 1F); ^{13}C NMR (100 MHz, $CDCl_3$): δ 158.8 (d, J = 2.8 Hz), 158.1, 157.6, 155.7, 150.7 (d, J = 256.4 Hz), 133.1, 132.9, 132.5, 130.2 (d, J = 7.7 Hz), 129.3, 126.6 (d, J = 4.3 Hz), 117.3, 115.2, 114.4, 113.9, 110.4 (d, J = 5.3 Hz), 108.5 (d, J = 10.6 Hz), 102.8, 55.7, 55.3; HRMS (ESI) calcd. For $C_{22}H_{18}FN_3O_2$ $[M+H]^+$: 376.1461, found: 376.1468.

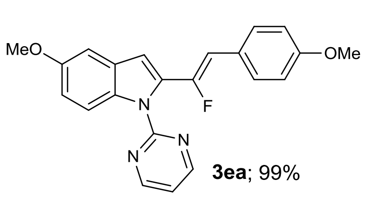

3ea; 99%

(*Z*)-2-(1-Fluoro-2-(4-methoxyphenyl)vinyl)-7-methy

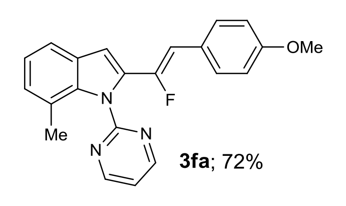

3fa; 72%

l-1-(pyrimidin-2-yl)-1*H*-indole (3fa): Procedure A, white solid, m.p. 130-131. ^{1}H NMR (400 MHz, $CDCl_3$): δ 8.85 (d, *J* = 4.9 Hz, 2H), 7.51 (d, *J* = 7.8 Hz, 1H), 7.39 (d, *J* = 4.8 Hz, 2H), 7.35 (t, *J* = 4.7 Hz, 1H), 7.10 (t, *J* = 7.4 Hz, 1H), 7.00 (d, *J* = 7.2 Hz, 1H), 6.95 (d, *J* = 1.4 Hz, 1H), 6.80-6.85 (m, 2H), 6.05 (d, *J* = 38.3 Hz, 1H), 3.79 (s, 3H), 1.94 (s, 3H); ^{19}F NMR (376 MHz, $CDCl_3$): δ -108.9 (d, *J* = 38.3 Hz, 1F); ^{13}C NMR (100 MHz, $CDCl_3$): δ 159.5, 158.9 (d, *J* = 3.0 Hz), 158.4, 149.6 (d, *J* = 254.6 Hz), 137.5, 133.8, 133.6, 130.2 (d, *J* = 7.7 Hz), 128.5, 126.8, 126.1 (d, *J* = 3.8 Hz), 121.9, 121.7, 120.1, 119.3, 113.9, 109.5 (d, *J* = 11.1 Hz), 107.1 (d, *J* = 4.5 Hz), 55.3, 19.6; HRMS (ESI) calcd. For $C_{22}H_{18}FN_3O$ $[M+H]^+$: 360.1512, found: 360.1507.

(*Z*)-2-(1-Fluoro-2-(4-methoxyphenyl)vinyl)-1-(pyrimidin-2-yl)-1*H*-indole-5-carbonitrile (3ga): Procedure A, white solid, m.p. 166-168. ^{1}H NMR (400 MHz, $CDCl_3$): δ 8.76 (d, *J* = 4.8 Hz, 2H), 8.13 (dd, J_1 = 10.7 Hz, J_2= 2.4 Hz, 1H), 7.47-7.58 (m, 3H), 7.16 (t, *J* = 4.7 Hz,1H), 7.00 (td, J_1 = 8.9 Hz, J_2 = 2.4 Hz, 1H), 6.85-6.94 (m, 3H), 6.20 (d, *J* = 36.4 Hz, 1H), 3.82 (s, 3H); ^{19}F NMR (376 MHz, $CDCl_3$): δ -103.3 (dd, J_1 = 36.4 Hz, J_2 = 2.1 Hz, 1F), -116.5 (m, 1F); ^{13}C NMR (100 MHz, $CDCl_3$): δ 162.5, 160.1, 158.9 (d, *J* = 2.5 Hz), 158.3, 157.5, 150.4 (d, *J* = 255.0 Hz), 137.7 (d, *J* = 13.0 Hz), 133.1 (dd, J_1 = 26.0 Hz, J_2 = 4.14 Hz), 130.2 (d, *J* = 7.7 Hz), 126.5 (d, *J* = 4.2 Hz), 124.9, 121.8 (d, *J* = 9.9 Hz), 117.7, 114.0, 111.2, 110.9, 110.3 (d, *J* = 5.5 Hz), 108.6 (d, *J* = 10.6 Hz), 101.5, 101.2, 55.3; HRMS (ESI) calcd. For $C_{21}H_{15}F_2N_3O$ $[M+H]^+$: 364.1261, found: 364.1265.

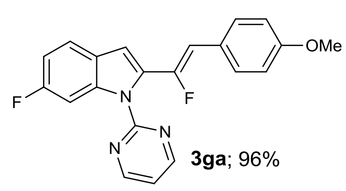

3ga; 96%

(*Z*)-5-Fluoro-2-(1-fluoro-2-(4-methoxyphenyl)vinyl)-1-(pyrimidin-2-yl)-1*H*-indole (3ha): Procedure A, white solid, m.p. 167-168. ^{1}H NMR (400 MHz, $CDCl_3$): δ 8.75 (d, *J* = 4.9 Hz, 2H), 8.31 (dd, J_1 = 9.2 Hz, J_2 = 4.6 Hz, 1H), 7.50-7.58 (m, 2H), 7.26 (dd, J_1 = 8.8 Hz, J_2 = 2.5 Hz, 1H), 7.15 (t, *J* = 4.6 Hz, 1H), 7.06 (td, J_1 = 8.4 Hz, J_2 = 2.6 Hz, 1H), 6.86-6.93 (m, 3H), 6.22 (d, *J* = 36.6 Hz, 1H), 3.82 (s, 3H); ^{19}F NMR (376 MHz, $CDCl_3$): δ -104.0 (dd, J_1 = 36.6 Hz, J_2 = 2.1 Hz, 1F), -121.3 (m, 1F); ^{13}C NMR (100 MHz, $CDCl_3$): δ 160.3, 158.9 (d, *J* = 2.8 Hz), 158.2, 157.9, 157.5, 150.3 (d, *J* = 255.3 Hz), 134.3, 134.0 (d, *J* = 3.3 Hz), 130.3 (d, *J* = 7.7 Hz), 129.2 (d, *J* = 10.1 Hz), 126.4 (d, *J* = 4.3 Hz), 117.7, 115.2 (d, *J* = 8.9 Hz), 114.0, 112.8, 112.6, 110.0 (t), 109.1 (d, *J* = 10.3 Hz), 106.1, 105.9, 55.3; HRMS (ESI) calcd. For $C_{21}H_{15}F_2N_3O$ $[M+H]^+$: 364.1261, found: 364.1269.

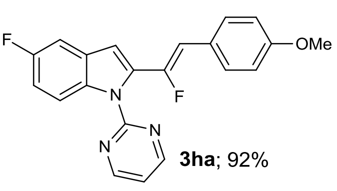

3ha; 92%

(*Z*)-5-Bromo-2-(1-fluoro-2-(4-methoxyphenyl)vinyl)-1-(pyrimidin-2-yl)-1*H*-indole (3ia): Procedure A, white solid, m.p. 159-161. ^{1}H NMR (400 MHz, $CDCl_3$): δ 8.76 (d, *J* = 4.8 Hz, 2H), 8.22 (d, *J* = 8.9 Hz, 1H), 7.7 (d, *J* = 1.8 Hz, 1H), 7.52 (d, *J* = 8.8 Hz, 2H), 7.40 (dd, J_1 = 8.9 Hz, J_2 = 1.8 Hz, 1H), 7.16 (t, *J* = 4.5 Hz, 1H), 6.81-6.93 (m, 3H), 6.23 (d, *J* = 38.6 Hz, 1H), 3.82 (s, 3H); ^{19}F NMR (376 MHz, $CDCl_3$): δ -104.2 (dd, J_1 = 38.6 Hz, J_2 = 1.7 Hz, 1F); ^{13}C NMR (100 MHz, $CDCl_3$): δ 159.0 (d, *J* = 2.9 Hz), 158.3, 157.4, 150.1 (d, *J* = 255.7 Hz), 136.2, 133.9, 133.6,

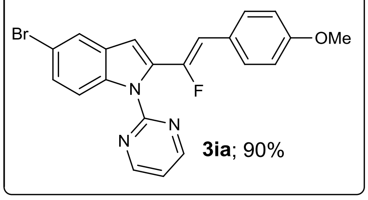

3ia; 90%

130.3 (d, *J* = 7.9 Hz), 127.5, 126.4 (d, *J* = 4.2 Hz), 123.5, 117.8, 115.6 (d, *J* = 7.3 Hz), 114.0, 109.3 (d, *J* = 5.3 Hz), 109.2, 55.3; HRMS (ESI) calcd. For $C_{21}H_{15}BrFN_3O$ $[M+H]^+$: 424.0461, found: 424.0454.

(*Z*)-6-Bromo-2-(1-fluoro-2-(4-methoxyphenyl)vinyl)-1-(pyrimidin-2-yl)-1*H*-indole

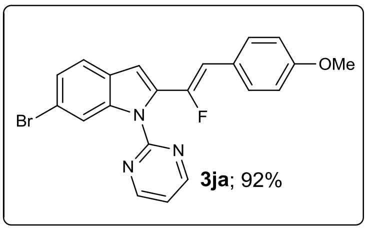

(3ja): Procedure A, white solid, m.p. 191-192. ^1H NMR (400 MHz, $CDCl_3$): δ 8.78 (d, *J* = 4.8 Hz, 2H), 8.55 (d, *J* = 1.1 Hz, 1H), 7.53 (d, *J* = 8.8 Hz, 2H), 7.47 (d, *J* = 8.4 Hz, 1H), 7.35 (dd, J_1 = 8.4 Hz, J_2 = 1.7 Hz, 1H), 7.19 (t, *J* = 5.3 Hz, 1H), 6.86-6.93 (m, 3H), 6.22 (d, *J* = 36.5 Hz, 1H), 3.83 (s, 3H); ^{19}F NMR (376 MHz, $CDCl_3$): δ -104.2 (dd, J_1 = 36.5 Hz, J_2 = 2.2 Hz, 1F); ^{13}C NMR (100 MHz, $CDCl_3$): δ 158.9 (d, *J* = 2.9 Hz), 158.3, 157.3, 150.2 (d, *J* = 255.1 Hz), 138.1, 133.3, 133.1, 130.2 (d, *J* = 7.7 Hz), 127.4, 126.4 (d, *J* = 4.3 Hz), 125.7, 122.1, 118.4, 117.8, 117.1, 114.0, 110.0 (d, *J* = 5.2 Hz), 109.1 (d, *J* = 10.3 Hz), 55.3; HRMS (ESI) calcd. For $C_{21}H_{15}BrFN_3O$ $[M+H]^+$: 424.0461, found: 424.0448.

(*Z*)-4-Chloro-2-(1-fluoro-2-(4-methoxyphenyl)vinyl)-1-(pyrimidin-2-yl)-1*H*-indol

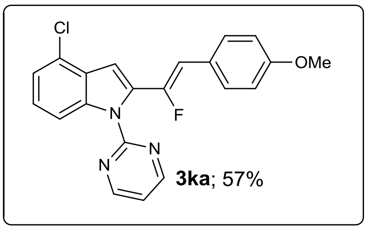

e (3ka): Procedure A, white solid, m.p. 169-170. ^1H NMR (400 MHz, $CDCl_3$): δ 8.78 (d, *J* = 4.8 Hz, 2H), 8.21 (t, *J* = 4.6 Hz,1H), 7.54 (d, *J* = 8.8 Hz, 2H), 7.24 (d, *J* = 5.5 Hz, 2H), 7.20 (t, *J* = 5.0 Hz, 1H), 7.07 (d, *J* = 1.9 Hz, 1H), 687-6.92 (m, 2H), 6.29 (d, *J* = 36.7 Hz, 1H), 3.83 (s, 3H); ^{19}F NMR (376 MHz, $CDCl_3$): δ -104.7 (dd, J_1 = 36.7 Hz, J_2= 2.3 Hz, 1F); ^{13}C NMR (100 MHz, $CDCl_3$): δ 159.0 (d, *J* = 2.9 Hz), 158.3, 157.4, 150.1 (d, *J* = 254.6 Hz), 138.1, 133.4, 133.2, 130.3 (d, *J* = 7.7 Hz), 128.6, 127.4, 126.4 (d, *J* = 4.2 Hz), 126.2, 125.2, 122.1, 118.0, 114.0, 112.5, 109.4 (d, *J* = 10.1 Hz), 108.1 (d, *J* = 5.3 Hz), 55.3; HRMS (ESI) calcd. For $C_{21}H_{15}ClFN_3O$ $[M+H]^+$: 380.0966, found: 380.0969.

Methyl-(*Z*)-2-(1-fluoro-2-(4-methoxyphenyl)vinyl)-1-(pyrimidin-2-yl)-1*H*-indole-

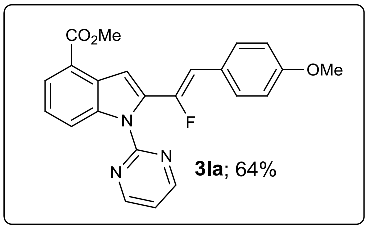

4-carboxylate (3la): Procedure A, white solid, m.p. 153-154. ^1H NMR (400 MHz, $CDCl_3$): δ 8.80 (d, *J* = 4.8 Hz, 2H), 8.52 (d, *J* = 8.8 Hz, 1H), 8.00 (d, *J* = 0.7 Hz, 1H), 7.65 (d, *J* = 2.3 Hz, 1H), 7.54 (d, *J* = 8.8 Hz, 2H), 7.37 (t, *J* = 7.9 Hz, 1H), 7.21 (t, *J* = 5.3 Hz, 1H), 6.90 (d, *J* = 8.8 Hz, 2H), 6.36 (d, *J* = 36.9 Hz, 1H), 4.01 (s, 3H), 3.83 (s, 3H); ^{19}F NMR (376 MHz, $CDCl_3$): δ -105.2 (dd, J_1 = 36.9 Hz, J_2 = 1.7 Hz, 1F); ^{13}C NMR (100 MHz, $CDCl_3$): δ 167.4, 159.0 (d, *J* = 2.9 Hz), 158.3, 157.4, 150.2 (d, *J* = 257.7 Hz), 138.2, 134.7, 134.4, 130.3 (d, *J* = 7.7 Hz), 128.3, 126.4 (d, *J* = 4.2 Hz), 125.5, 123.9, 121.9, 119.5, 118.0, 114.0, 110.6 (d, *J* = 5.3 Hz), 109.6 (d, *J* = 9.9 Hz), 55.3, 51.9; HRMS (ESI) calcd. For $C_{23}H_{18}FN_3O_3$ $[M+H]^+$: 404.1410, found: 404.1420.

(Z)-2-(1-Fluoro-2-(4-methoxyphenyl)vinyl)-1-(pyrimidin-2-yl)-1H-indole-5-carbo

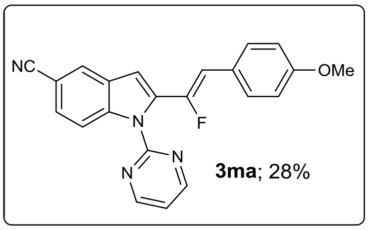

nitrile (3ma): Procedure A, white solid, m.p. 212-214. ^1H NMR (400 MHz, $CDCl_3$): δ 8.83 (d, *J* = 4.8 Hz,

2H), 8.38 (d, J = 8.8 Hz, 1H), 7.98 (d, J = 1.0 Hz, 1H), 7.52-7.58 (m, 3H), 7.26 (t, J = 4.6 Hz, 1H), 6.99 (d, J = 1.8 Hz, 1H), 6.91 (d, J = 8.7 Hz, 2H), 6.27 (d, J = 36.6 Hz, 1H), 3.84 (s, 3H); ^{19}F NMR (376 MHz, $CDCl_3$): δ -105.4 (dd, J_1 = 36.6 Hz, J_2 = 1.9 Hz, 1F); ^{13}C NMR (100 MHz, $CDCl_3$): δ 159.1 (d, J = 2.9 Hz), 158.5, 149.4 (d, J = 255.1 Hz), 138.9, 135.1, 134.9, 130.4 (d, J = 7.8 Hz), 128.3, 127.4, 126.2, 126.0 (d, J = 4.2 Hz), 119.9, 118.5, 114.8, 114.1, 110.0 (d, J = 9.8 Hz), 109.4 (d, J = 5.1 Hz), 105.7, 55.3; HRMS (ESI) calcd. For $C_{22}H_{15}FN_4O$ $[M+H]^+$: 371.1308, found: 371.1316.

Methyl-(*Z*)-5-(1-fluoro-2-(4-methoxyphenyl)vinyl)-1-(pyrimidin-2-yl)-1*H*-pyrrole -2-carboxylate (3na): Procedure B, white solid , m.p. 119-120. ^{1}H NMR (400 MHz, $CDCl_3$): δ 8.85 (d, J = 4.8 Hz, 2H), 7.41 (t, J = 4.8 Hz, 1H), 7.33 (d, J = 8.9 Hz, 2H), 7.05 (dd, J_1 = 4.0 Hz, J_2 = 1.2 Hz, 1H), 6.81 (d, J = 8.8 Hz, 2H), 6.80 (dd, J_1 = 4.0 Hz, J_2 = 0.9 Hz, 1H), 5.92 (d, J = 38.8 Hz, 1H), 3.78 (s, 3H), 3.70 (s, 3H); ^{19}F NMR (376 MHz, $CDCl_3$): δ -112.0 (d, J = 38.8 Hz, 1F); ^{13}C NMR (100 MHz, $CDCl_3$): δ 160.7, 159.0 (d, J = 3.0 Hz), 158.5, 158.3, 148.5 (d, J = 253.8 Hz), 132.9, 132.7, 130.2 (d, J = 7.8 Hz), 125.7 (d, J = 3.6 Hz), 125.6, 120.7, 117.9, 113.9, 110.8 (d, J = 4.3 Hz), 109.2 (d, J = 9.6 Hz), 55.3, 51.6; HRMS (ESI) calcd. For $C_{19}H_{16}FN_3O_3$ $[M+H]^+$: 354.1254, found: 354.1247.

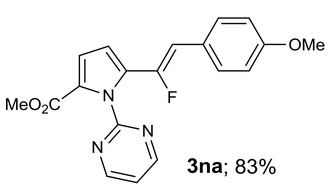

3na; 83%

(*Z*)-1-(5-(1-Fluoro-2-(4-methoxyphenyl)vinyl)-1-(pyrimidin-2-yl)-1*H*-pyrrol-2-yl) ethan-1-one (3oa): Procedure B, yellow solid, m.p. 150-152. ^{1}H NMR (400 MHz, $CDCl_3$): δ 8.80 (d, J = 4.9 Hz, 2H), 7.40 (t, J = 4.8 Hz, 1H), 7.32 (d, J = 8.9 Hz, 2H), 7.04 (dd, J_1 = 4.1 Hz, J_2 = 0.9 Hz, 1H), 6.81 (d, J = 8.8 Hz, 2H), 6.59 (d, J = 4.1 Hz, 1H), 5.91 (d, J = 38.9 Hz, 1H), 3.78 (s, 3H), 2.41 (s, 3H); ^{19}F NMR (376 MHz, $CDCl_3$): δ -112.8 (d, J = 38.9 Hz, 1F); ^{13}C NMR (100 MHz, $CDCl_3$): δ 187.0, 159.1 (d, J = 3.0 Hz), 158.9, 158.5, 148.4 (d, J = 254.4 Hz), 133.9, 133.8, 133.6, 130.3 (d, J = 7.8 Hz), 125.6 (d, J = 3.6 Hz), 120.6, 119.1, 114.0, 110.7 (d, J = 4.2 Hz), 109.8 (d, J = 9.4 Hz), 55.3, 26.5; HRMS (ESI) calcd. For $C_{19}H_{16}FN_3O_2$ $[M+H]^+$: 338.1305, found: 338.1313.

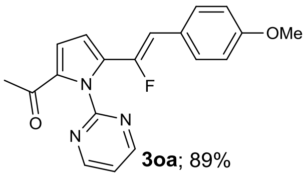

3oa; 89%

(*Z*)-2-(3-(1-Fluoro-2-(4-methoxyphenyl)vinyl)thiophen-2-yl)pyrimidine (3pa): Procedure A, colorless oil. ^{1}H NMR (400 MHz, $CDCl_3$): δ 8.74 (d, J = 4.9 Hz, 2H), 7.56 (d, J = 8.9 Hz, 2H), 7.42 (dd, J_1 = 5.2 Hz, J_2 = 1.0 Hz, 1H), 7.23 (dd, J_1 = 5.2 Hz, J_2 = 0.6 Hz, 1H), 7.13 (t, J = 4.9 Hz, 1H), 6.90 (d, J = 8.9 Hz, 2H), 6.17 (d, J = 38.1 Hz, 1H), 3.83 (s, 3H); ^{19}F NMR (376 MHz, $CDCl_3$): δ -62.6 (s, 3F), -100.5 (d, J = 38.1 Hz, 1F); ^{13}C NMR (100 MHz, $CDCl_3$): δ 161.5, 158.8 (d, J = 2.9 Hz), 157.0, 152.6 (d, J = 257.3 Hz), 139.5, 134.8, 134.6, 130.4(d, J = 2.5 Hz), 130.4 (d, J = 5.9 Hz), 128.1, 126.6 (d, J = 3.8 Hz), 118.6, 113.9, 109.4 (d, J = 10.3 Hz), 55.3; HRMS (ESI) calcd. For $C_{17}H_{13}FN_2OS$ $[M+H]^+$: 313.0811, found: 313.0807.

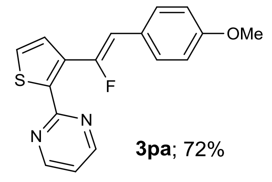

3pa; 72%

(*Z*)-2-(2-(1-Fluoro-2-(4-methoxyphenyl)vinyl)phenyl)pyridine (5aa): Procedure A,

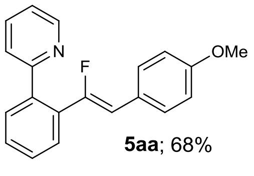

5aa; 68%

colorless oil. ^{1}H NMR (400 MHz, $CDCl_3$): δ 8.61 (dq, J_1 = 4.5 Hz, J_2 = 0.9 Hz, 1H), 7.61 (td, J_1 = 7.8 Hz, J_2 = 1.9 Hz, 1H), 7.50-7.57 (m, 2H), 7.29-7.47 (m, 5H), 7.16 (ddd, J_1 = 8.0 Hz, J_2 = 4.8 Hz, J_3 = 1.2 Hz, 1H), 6.77 (d, J = 8.8 Hz, 2H), 5.75 (d, J = 38.5 Hz, 1H), 3.73 (s, 3H); ^{19}F NMR (376 MHz, $CDCl_3$): δ -100.6 (d, J = 38.5 Hz, 1F); ^{13}C NMR (100 MHz, $CDCl_3$): δ 159.0, 158.7 (d, J = 2.9 Hz), 156.6 (d, J = 260.2 Hz), 149.5, 139.1, 136.1, 132.5, 132.2, 130.1 (d, J = 7.8 Hz), 129.2, 129.0 (d, J = 4.7 Hz), 128.3, 126.5 (d, J = 3.5 Hz), 123.5 (d, J = 2.1 Hz), 121.9, 113.9, 109.9 (d, J = 10.3 Hz), 55.3; HRMS (ESI) calcd. For $C_{20}H_{16}FNO$ $[M+H]^+$: 306.1294, found: 306.1297.

(*Z*)-2-(2-(1-Fluoro-2-(4-methoxyphenyl)vinyl)-6-methylphenyl)pyridine (5ba):

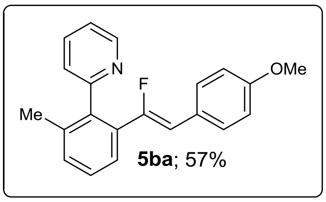

Procedure A, colorless oil. ^{1}H NMR (400 MHz, $CDCl_3$): δ 8.70 (d, J = 4.5 Hz, 1H), 7.69 (t, J = 6.5 Hz, 1H), 7.47 (d, J = 7.1 Hz, 1H), 7.21-7.37 (m, 4H), 6.78 (d, J = 8.6 Hz, 2H), 5.54 (d, J = 39.2 Hz, 1H), 3.77 (s, 3H), 2.13 (s, 3H); ^{19}F NMR (376 MHz, $CDCl_3$): δ -100.9 (d, J = 39.2 Hz, 1F); ^{13}C NMR (100 MHz, $CDCl_3$): δ 159.3, 158.5 (d, J = 2.9 Hz), 156.2 (d, J = 259.1 Hz), 149.3, 138.7, 137.0, 136.4, 133.0, 132.8, 131.1, 130.0 (d, J = 7.8 Hz), 128.0, 126.5 (d, J = 3.3 Hz), 125.9 (d, J = 5.2 Hz), 124.4 (d, J = 1.7 Hz), 121.9, 113.8, 110.2 (d, J = 10.5 Hz), 55.2, 20.3; HRMS (ESI) calcd. For $C_{21}H_{18}FNO$ $[M+H]^+$: 320.1451, found: 320.1452.

(*Z*)-2-(2-(1-Fluoro-2-(4-methoxyphenyl)vinyl)-6-methoxyphenyl)pyridine (5ca):

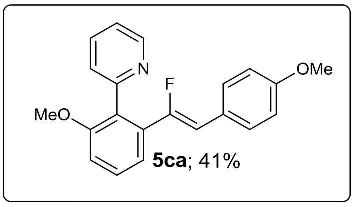

Procedure A, colorless oil. ^{1}H NMR (400 MHz, $CDCl_3$): δ 8.68 (dq, J_1 = 4.5 Hz, J_2 = 0.9 Hz, 1H), 7.70 (td, J_1 = 7.8 Hz, J_2 = 1.9 Hz, 1H), 7.34-7.43 (m, 2H), 7.18-7.32 (m, 4H), 7.01 (d, J = 8.3 Hz, 1H), 6.79 (d, J = 8.8 Hz, 2H), 5.60 (d, J = 39.7 Hz, 1H), 3.78 (s, 3H), 3.76 (s, 3H); ^{19}F NMR (376 MHz, $CDCl_3$): δ -102.3 (d, J = 39.7 Hz, 1F); ^{13}C NMR (100 MHz, $CDCl_3$): δ 158.6 (d, J = 2.9 Hz), 157.3, 156.4, 155.8 (d, J = 259.2 Hz), 149.2, 135.9, 134.5, 134.3, 130.0 (d, J = 7.8 Hz), 129.8, 129.3, 128.3, 126.5 (d, J = 3.3 Hz), 125.3 (d, J = 11.3 Hz), 121.9, 120.6 (d, J = 5.5Hz), 113.8, 111.9, 110.3 (d, J = 10.3 Hz), 56.1, 55.2; HRMS (ESI) calcd. For $C_{21}H_{18}FNO_2$ $[M+H]^+$: 336.1400, found: 336.1398.

(*Z*)-2-(2-(1-Fluoro-2-(4-methoxyphenyl)vinyl)-5-methylphenyl)pyridine (5da):

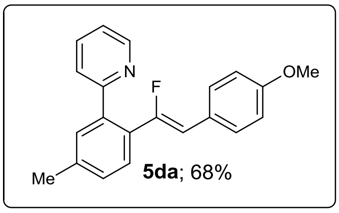

Procedure A, colorless oil. ^{1}H NMR (400 MHz, $CDCl_3$): δ 8.68 (dq, J_1 = 4.8 Hz, J_2 = 0.9 Hz, 1H), 7.66 (tdd, J_1 = 7.8 Hz, J_2 = 1.9 Hz, J_3 = 0.9 Hz, 1H), 7.47-7.54 (m, 2H), 7.43 (s, 1H), 7.39 (d, J = 8.0 Hz, 2H), 7.18-7.28 (m, 2H), 6.83 (d, J = 8.5 Hz, 2H), 5.77 (d, J = 37.3 Hz, 1H), 3.79 (s, 3H), 2.42 (s, 3H); ^{19}F NMR (376 MHz, $CDCl_3$): δ -101.0 (d, J = 37.3 Hz, 1F); ^{13}C NMR (100 MHz, $CDCl_3$): δ 159.1, 158.6 (d, J = 3.0 Hz), 157.0 (d, J = 260.6 Hz), 149.4, 139.5, 138.8, 136.1, 131.2, 130.0 (d, J = 7.8 Hz), 129.7, 129.4, 129.0(t), 126.6 (d, J = 3.5 Hz), 123.6 (d, J = 2.2 Hz), 121.9, 113.9, 109.4 (d, J = 10.6 Hz), 55.3, 21.3; HRMS (ESI) calcd. For $C_{21}H_{18}FNO$ $[M+H]^+$: 320.1451, found: 320.1449.

(*Z*)-2-(2-(1-Fluoro-2-(4-methoxyphenyl)vinyl)-5-methoxyphenyl)pyridine (5ea):

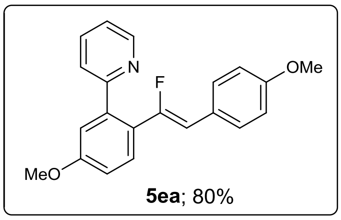

5ea; 80%

Procedure A, colorless oil. ^{1}H NMR (400 MHz, $CDCl_3$): δ 8.68 (dq, J_1 = 4.8 Hz, J_2 = 0.9 Hz, 1H), 7.67 (td, J_1 = 8.3 Hz, J_2 = 1.4 Hz, 1H), 7.52 (dq, J_1 = 8.6 Hz, J_2 = 1.0 Hz, 2H), 7.34-7.41 (m, 2H), 7.20-7.25 (m, 1H), 7.14 (d, *J* = 3.1 Hz, 1H), 6.97 (ddd, J_1 = 8.3 Hz, J_2 = 2.9 Hz, J_3 = 1.0 Hz, 1H), 6.79-6.83 (m, 2H), 5.72 (d, *J* = 38.4 Hz, 1H), 3.87 (s, 3H), 3.79 (s, 3H); ^{19}F NMR (376 MHz, $CDCl_3$): δ -98.8 (d, *J* = 38.4 Hz, 1F); ^{13}C NMR (100 MHz, $CDCl_3$): δ 160.3, 158.8, 158.5 (d, *J* = 2.9 Hz), 156.7 (d, *J* = 260.4 Hz), 149.4, 104.7, 136.2, 130.7 (d, *J* = 4.6 Hz), 129.9 (d, *J* = 7.8 Hz), 126.7 (d, *J* = 3.6 Hz), 125.2, 124.9, 123.6 (d, *J* = 3.5 Hz), 122.1, 115.3, 114.4, 113.9, 109.0 (d, *J* = 11.1 Hz), 55.5, 55.3; HRMS (ESI) calcd. For $C_{21}H_{18}FNO_2$ $[M+H]^+$: 336.1400, found: 336.1396.

(*Z*)-2-(2-(1-Fluoro-2-(4-methoxyphenyl)vinyl)-4-methoxyphenyl)pyridine (5fa):

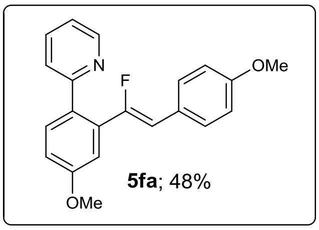

5fa; 48%

Procedure B, colorless oil. ^{1}H NMR (400 MHz, $CDCl_3$): δ 8.65 (d, *J* = 4.8 Hz, 1H), 7.64 (td, J_1 = 7.8 Hz, J_2 = 1.8 Hz, 1H), 7.55 (d, *J* = 8.5 Hz, 1H), 7.48 (d, *J* = 7.9 Hz, 1H), 7.39 (d, *J* = 8.8 Hz, 2H), 7.19 (ddd, J_1 = 12.4 Hz, J_2 = 4.9 Hz, J_3 = 1.0 Hz,1H), 7.13 (d, *J* = 2.6 Hz, 1H), 7.02 (dd, J_1 = 8.6 Hz, J_2 = 2.6 Hz, 1H), 6.85 (d, *J* = 8.8 Hz, 2H), 5.80 (d, *J* = 38.5 Hz, 1H), 3.89 (s, 3H), 3.80 (s, 3H); ^{19}F NMR (376 MHz, $CDCl_3$): δ -100.7 (d, *J* = 38.5 Hz, 1F); ^{13}C NMR (100 MHz, $CDCl_3$): δ 159.5, 158.7 (d, *J* = 3.0 Hz), 156.4 (d, *J* = 260.4 Hz), 149.4, 136.1, 133.7, 133.4, 132.0 (d, *J* = 7.8Hz), 130.1 (d, *J* = 7.8 Hz), 126.4 (d, *J* = 3.5 Hz), 123.5 (d, *J* = 2.1 Hz), 121.6, 114.9, 114.2 (d, *J* = 4.9 Hz), 113.9, 110.0 (d, *J* = 10.2 Hz), 55.5, 55.3; HRMS (ESI) calcd. For $C_{21}H_{18}FNO_2$ $[M+H]^+$: 336.1400, found: 336.1400.

Methyl (*Z*)-3-(1-fluoro-2-(4-methoxyphenyl)vinyl)-4-(pyridin-2-yl)benzoate (5ga):

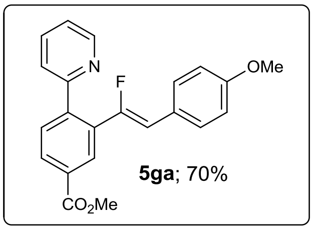

5ga; 70%

Procedure B, colorless oil. ^{1}H NMR (400 MHz, $CDCl_3$): δ 8.70 (dq, J_1 = 4.9 Hz, J_2 = 0.8 Hz, 1H), 8.29 (t, *J* = 1.5 Hz, 1H), 8.12 (dd, J_1 = 7.7 Hz, J_2 = 1.8 Hz, 1H), 7.67-7.74 (m, 2H), 7.55 (dd, J_1 = 8.1 Hz, J_2 = 1.3 Hz, 1H), 7.42 (d, *J* = 8.8 Hz, 2H), 7.26-7.30 (m, 1H), 6.86 (d, *J* = 8.9 Hz, 2H), 5.95 (d, *J* = 38.7 Hz, 1H), 3.97 (s, 3H), 3.81 (s, 3H); ^{19}F NMR (376 MHz, $CDCl_3$): δ -101.6 (d, *J* = 38.7 Hz, 1F); ^{13}C NMR (100 MHz, $CDCl_3$): δ 166.4, 158.9 (d, *J* = 2.5 Hz), 158.0, 155.7 (d, *J* = 259.6 Hz), 149.6, 143.1, 136.3, 132.8, 132.6, 130.9, 130.3 (d, *J* = 5.1 Hz), 130.2, 130.1 (d, *J* = 4.2 Hz), 130.0, 126.8, 126.2 (d, *J* = 3.8 Hz), 123.4 (d, *J* = 2.8 Hz), 122.5, 113.9, 110.5 (d, *J* = 10.5 Hz), 55.3, 52.4; HRMS (ESI) calcd. For $C_{22}H_{18}FNO_3$ $[M+H]^+$: 364.1349, found: 364.1351.

(*Z*)-1-(3-(1-Fluoro-2-(4-methoxyphenyl)vinyl)-4-(pyridin-2-yl)phenyl)ethan-1-one

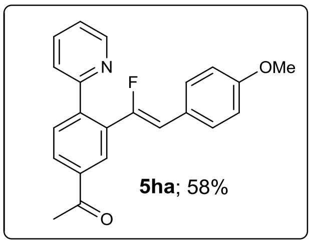

5ha; 58%

(5ha): Procedure B, colorless oil. ^{1}H NMR (400 MHz, $CDCl_3$): δ 8.71 (dq, J_1 = 4.9 Hz, J_2 = 0.9 Hz, 1H), 8.20 (s, 1H), 8.04 (dd, J_1 = 7.6 Hz, J_2 = 1.5 Hz, 1H), 7.73 (d, *J* = 8.0 Hz, 2H), 7.56 (dd, J_1 = 8.3 Hz, J_2 = 0.9 Hz, 1H), 7.42 (d, *J* =

8.8 Hz, 2H), 7.24-7.31(m, 1H), 6.86 (d, *J* = 8.5 Hz, 2H), 5.93 (d, *J* = 38.3 Hz, 1H), 3.81 (s, 3H), 2.68 (s, 3H); ^{19}F NMR (376 MHz, $CDCl_3$): δ -101.5 (d, *J* = 38.3 Hz, 1F); ^{13}C NMR (100 MHz, $CDCl_3$): δ 197.4, 158.9 (d, *J* = 3.0 Hz), 157.9, 153.3 (d, *J* = 258.9 Hz), 149.7, 143.2, 136.7, 136.4, 133.0, 132.8, 131.1, 130.2 (d, *J* = 9.2 Hz), 129.0 (d, *J* = 4.8 Hz), 128.9, 126.1 (d, *J* = 3.4 Hz), 123.5 (d, *J* = 2.2 Hz), 122.6, 114.0, 110.7 (d, *J* = 9.8 Hz), 55.3, 26.8; HRMS (ESI) calcd. For $C_{22}H_{18}FNO_2$ $[M+H]^+$: 348.1400, found: 348.1403.

(*Z*)-2-(2-(1-Fluoro-2-(4-methoxyphenyl)vinyl)-4-(trifluoromethyl)phenyl)pyridine (5ia): Procedure B, colorless oil. ^{1}H NMR (400 MHz, $CDCl_3$): δ 8.70 (d, *J* = 4.8 Hz, 2H), 7.87 (s, 1H), 7.68-7.76 (m, 3H), 7.53 (d, *J* = 8.2 Hz, 1H), 7.37-7.44 (m, 2H), 7.26-7.32 (m, 1H), 6.85 (d, *J* = 8.8 Hz, 2H), 5.89 (d, *J* = 38.3 Hz, 1H), 3.80 (s, 3H); ^{19}F NMR (376 MHz, $CDCl_3$): δ -62.6 (s, 3F), -102.4 (d, *J* = 38.3 Hz, 1F); ^{13}C NMR (100 MHz, $CDCl_3$): δ 159.1 (d, *J* = 2.9 Hz), 157.7, 155.1 (d, *J* = 257.5 Hz), 149.7, 142.3, 136.8, 136.4, 133.2, 133.0, 131.3, 130.8, 130.4, 130.3 (d, *J* = 7.8 Hz), 125.9(m), 125.2, 123.5, 122.6, 113.9, 111.1 (d, *J* = 9.7 Hz), 55.3; HRMS (ESI) calcd. For $C_{21}H_{15}F_4NO$ $[M+H]^+$: 374.1168, found: 374.1174.

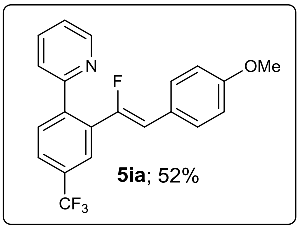

5ia; 52%

(*Z*)-2-(4-Fluoro-2-(1-fluoro-2-(4-methoxyphenyl)vinyl)phenyl)pyridine (5ja): Procedure A, colorless oil. ^{1}H NMR (400 MHz, $CDCl_3$): δ 8.67 (dq, J_1 = 4.7 Hz, J_2 = 1.0 Hz, 1H), 7.68 (td, J_1 = 7.8 Hz, J_2 = 2.1 Hz, 1H), 7.57 (dd, J_1 = 8.5 Hz, J_2 = 5.6 Hz, 1H), 7.48 (dd, J_1 = 8.1 Hz, J_2 = 1.0 Hz, 1H), 7.36-7.40 (m, 2H), 7.32 (dd, J_1 = 9.8 Hz, J_2 = 2.5 Hz, 1H), 7.19-7.29 (m, 1H), 7.16 (td, J_1 = 7.9 Hz, J_2 = 2.5 Hz, 1H), 6.84 (d, *J* = 8.7 Hz, 2H), 5.79 (d, *J* = 38.4 Hz,1H), 3.80 (s, 3H); ^{19}F NMR (376 MHz, $CDCl_3$): δ -102.5 (d, *J* = 38.4 Hz, 1F), -113.4 (m, 1F); ^{13}C NMR (100 MHz, $CDCl_3$): δ 162.4 (d, *J* = 249.3 Hz), 158.9 (d, *J* = 3.0 Hz), 158.2, 156.4 (d, *J* = 3.9 Hz), 153.8 (d, *J* = 2.4 Hz), 149.5, 136.3, 135.2 (d, *J* = 2.5 Hz), 134.3 (dd, J_1 = 25.7 Hz, J_2 = 7.1 Hz), 132.6 (d, *J* = 8.4 Hz), 130.2 (d, *J* = 7.9 Hz), 126.0 (d, *J* = 3.5 Hz), 123.6 (d, *J* = 1.9Hz), 122.1, 116.2, 116.0, 115.5 (dd, J_1 = 23.5 Hz, J_2 = 5.3 Hz), 113.9, 110.8 (d, *J* = 9.9 Hz), 55.3; HRMS (ESI) calcd. For $C_{20}H_{15}F_2NO$ $[M+H]^+$: 324.1200, found: 324.1202.

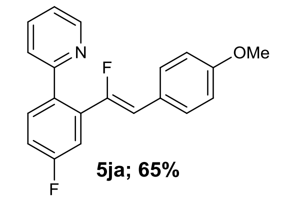

5ja; 65%

(*Z*)-2-(2-(1-Fluoro-2-(4-methoxyphenyl)vinyl)phenyl)-4-phenylpyridine (5ka): Procedure A, yellow solid, m.p. 108-110. ^{1}H NMR (400 MHz, $CDCl_3$): δ 8.72 (dd, J_1 = 5.2 Hz, J_2 = 0.9 Hz, 1H), 7.79 (s, 1H), 7.67(dd, J_1 = 7.8 Hz, J_2 = 2.2 Hz, 1H), 7.64 (d, *J* = 7.6 Hz, 1H), 7.54-7.58(m, 2H), 7.47-7.52 (m,1H), 7.41-7.46 (m, 4H), 7.36-7.40 (m, 3H), 6.84 (d, *J* = 8.8 Hz, 2H), 5.92 (d, *J* = 38.5 Hz, 1H), 3.79 (s, 3H); ^{19}F NMR (376 MHz, $CDCl_3$): δ -99.9 (d, *J* = 38.5 Hz, 1F); ^{13}C NMR (100 MHz, $CDCl_3$): δ 159.5, 158.7 (d, *J* = 2.9 Hz), 156.9 (d, *J* = 258.6 Hz), 149.9, 148.6, 139.2, 138.3, 132.6, 132.4, 130.5, 130.1 (d, *J* = 7.7 Hz), 129.4, 129.1 (d, *J* = 4.7 Hz), 129.0 (d, *J* = 8.8 Hz), 128.5, 127.1, 126.5 (d, *J* = 3.5 Hz), 121.6 (d, *J* = 2.1 Hz), 120.1, 113.9, 110.0 (d, *J* = 10.3 Hz), 55.3; HRMS (ESI) calcd. For $C_{26}H_{20}FNO$ $[M+H]^+$: 382.1607, found: 382.1601.

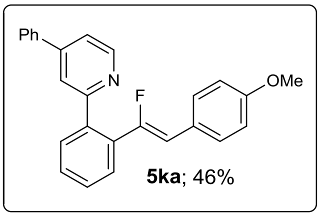

5ka; 46%

(*Z*)-2-(2-(1-Fluoro-2-(4-methoxyphenyl)vinyl)phenyl)pyrimidine (5la): Procedure A, white solid, m.p. 121-122. ^{1}H NMR (400 MHz, $CDCl_3$): δ 8.80 (d, *J* = 4.9 Hz, 2H), 7.82-7.88 (m, 1H), 7.59-7.66 (m, 1H), 7.42-7.53 (m, 4H), 7.21 (t, *J* = 4.9 Hz, 1H), 6.86 (d, *J* = 10.9 Hz, 2H), 6.00 (d, *J* = 38.1 Hz, 1H), 3.80 (s, 3H); ^{19}F NMR (376 MHz, $CDCl_3$): δ -99.3 (d, *J* = 38.1 Hz, 1F); ^{13}C NMR (100 MHz, $CDCl_3$): δ 167.1, 158.6 (d, *J* = 3.0 Hz), 157.1, 156.0, 137.5, 133.3, 133.0, 130.8, 130.1 (d, *J* = 7.8 Hz), 129.5 (d, *J* = 4.8 Hz), 129.4 (d, *J* = 12.1 Hz), 126.8 (d, *J* = 3.4 Hz), 118.9, 113.9, 108.6 (d, *J* = 10.4 Hz), 55.3; HRMS (ESI) calcd. For $C_{19}H_{15}FN_2O$ $[M+H]^+$: 307.1247, found: 307.1248.

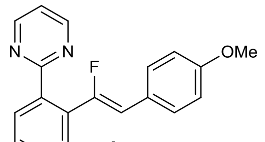

5la; 73%

(*Z*)-10-(1-Fluoro-2-(4-methoxyphenyl)vinyl)benzo[*h*]quinolone (5ma): Procedure A, colorless oil. ^{1}H NMR (400 MHz, $CDCl_3$): δ 8.98 (dd, J_1 = 4.3 Hz, J_2 = 1.9 Hz, 1H), 8.16 (dd, J_1 = 8.1 Hz, J_2 = 1.8 Hz, 1H), 7.99 (d, *J* = 8.0 Hz, 1H), 7.81-7.87 (m, 2H), 7.65-7.76 (m, 4H), 7.48 (dd, J_1 = 8.0 Hz, J_2 = 4.2 Hz, 1H), 6.89-7.03 (m, 2H), 6.01 (d, *J* = 36.7 Hz, 1H), 3.86 (s, 3H); ^{19}F NMR (376 MHz, $CDCl_3$): δ -90.1 (d, *J* = 38.1 Hz, 1F); ^{13}C NMR (100 MHz, $CDCl_3$): δ 160.8 (d, *J* = 259.5 Hz), 158.3 (d, *J* = 2.6 Hz), 148.3, 146.0, 135.3, 134.6 (d, *J* = 1.5 Hz), 131.9, 131.6 (d, *J* = 3.9 Hz), 130.2 (d, *J* = 1.9 Hz), 129.9 (d, *J* = 7.4 Hz), 127.9, 127.8, 127.2 (d, *J* = 5.3 Hz), 126.3, 121.6, 113.9, 106.2 (d, *J* = 11.1 Hz), 55.3; HRMS (ESI) calcd. For $C_{22}H_{16}FNO$ $[M+H]^+$: 330.1294, found: 330.1293.

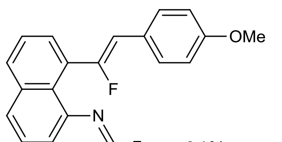

5ma; 84%

(*Z*)-10-(1-Fluoro-2-(4-methoxyphenyl)vinyl)-4-phenyl-5,6-dihydrobenzo[*h*] quino-lone (5na): Procedure A, greenish solid, m.p. 132-133.^{1}H NMR (400 MHz, $CDCl_3$): δ 8.51 (d, *J* = 5.0 Hz, 1H), 7.58 (d, *J* = 9.4 Hz, 2H), 7.36-7.50 (m, 6H), 7.24-7.33 (m, 2H), 7.10 (d, *J* = 5.2 Hz, 1H), 6.90 (d, *J* = 9.0 Hz, 2H), 6.09 (d, *J* = 37.9 Hz, 1H), 3.82 (s, 3H), 2.88 (m, 2H), 2.74 (m, 2H); ^{19}F NMR (376 MHz, $CDCl_3$): δ -95.5 (d, *J* = 37.9 Hz, 1F); ^{13}C NMR (100 MHz, $CDCl_3$): δ 160.7 (d, *J* = 258.9 Hz), 158.3 (d, *J* = 2.7 Hz), 152.9, 147.7, 146.9, 140.6, 138.8, 133.7, 132.3, 132.0, 130.0 (d, *J* = 3.3 Hz), 129.9, 129.0, 128.6, 128.4, 128.3, 128.0, 127.6 (d, *J* = 3.4 Hz), 122.9, 113.9, 106.9 (d, *J* = 10.6Hz), 55.3, 29.1, 25.6; HRMS (ESI) calcd. For $C_{28}H_{22}FNO$ $[M+H]^+$: 408.1764, found: 408.1761.

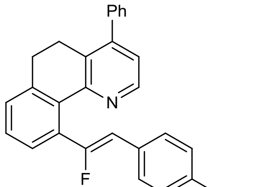

5na; 83%

(*Z*)-2-(1-Fluoro-2-(4-methoxyphenyl)vinyl)-4-methyl-*N*-tosylbenzamide (7aa): Procedure C, light yellow viscous oil. ^{1}H NMR (400 MHz, $CDCl_3$): δ 8.37 (s, 1H), 7.93 (d, *J* = 8.4 Hz, 2H), 7.44 (d, *J* = 7.7 Hz, 1H), 7.37 (d, *J* = 8.8 Hz, 2H), 7.32 (s, 1H), 7.19-7.29 (m, 3H), 6.89 (d, *J* = 9.1 Hz, 2H), 5.95 (d, *J* = 38.6 Hz, 1H), 3.86 (s, 3H), 2.44 (s, 3H), 2.39 (s, 3H); ^{19}F

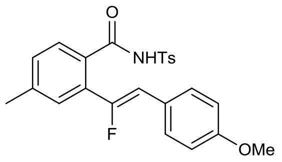

7aa; 68%

NMR (376 MHz, $CDCl_3$): δ -102.7 (d, J_1 = 38.6 Hz, 1F); ^{13}C NMR (100 MHz, $CDCl_3$): δ 166.0, 159.2 (d, J = 10.8 Hz), 153.9 (d, J = 256.7 Hz), 144.9, 141.9, 135.2, 131.4 (d, J = 27.6 Hz), 130.5 (d, J = 8.2 Hz), 130.1, 129.4, 129.1, 128.8 (d, J = 4.5 Hz), 128.7, 125.5 (d, J = 4.7 Hz), 113.9, 110.6 (d, J = 12.6 Hz), 55.3, 21.7, 21.4; HRMS (ESI) calcd. For $C_{24}H_{22}FNO_4S$ $[M+H]^+$: 440.1332, found: 440.1328.

(*Z*)-2-(1-Fluoro-2-(4-methoxyphenyl)vinyl)-6-methyl-*N*-tosylbenzamide (7ba):

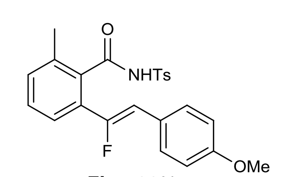

7ba; 66%

Procedure C, white solid, m.p. 85-87. ^1H NMR (400 MHz, $CDCl_3$): δ 8.27 (s, 1H), 7.90 (d, J = 9.0 Hz, 2H), 7.28-7.36 (m, 2H), 7.22-7.35 (m, 2H), 7.16-7.22 (m, 3H), 6.84 (d, J = 8.8 Hz, 2H), 5.91 (d, J = 37.1 Hz, 1H), 3.85 (s, 3H), 2.40 (s, 3H), 2.32 (s, 3H); ^{19}F NMR (376 MHz, $CDCl_3$): δ -105.4 (d, J = 37.1 Hz, 1F); ^{13}C NMR (100 MHz, $CDCl_3$): δ 166.5, 159.1 (d, J = 3.2 Hz), 153.7 (d, J = 264.2 Hz), 144.9, 136.1, 134.9, 131.5, 131.2, 130.5 (d, J = 11.1 Hz), 130.0, 129.4, 128.6, 125.6 (d, J = 4.1 Hz), 124.6 (d, J = 5.2 Hz),113.8, 110.0 (d, J = 12.1 Hz), 55.3, 21.7, 19.1; HRMS (ESI) calcd. For $C_{24}H_{22}FNO_4S$ $[M+H]^+$: 440.1332, found: 440.1342.

(*Z*)-2-(1-Fluoro-2-(4-methoxyphenyl)vinyl)-4-methoxy-*N*-tosylbenzamide (7ca):

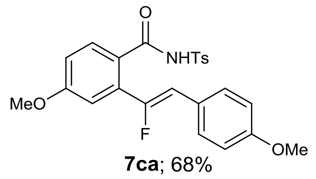

7ca; 68%

Procedure C, light yellow viscous oil. ^1H NMR (400 MHz, $CDCl_3$): δ 7.90 (d, J = 7.7 Hz, 2H), 7.48 (d, J = 7.7 Hz,1H), 7.38 (d, J = 8.8 Hz, 2H), 7.23 (d, J = 7.7 Hz, 2H), 6.95 (d, J = 2.1 Hz, 1H), 6.83-6.92 (m, 3H), 5.93 (d, J = 39.9 Hz, 1H), 3.84 (s, 3H), 3.83 (s, 3H), 2.43 (s, 3H); ^{19}F NMR (376 MHz, $CDCl_3$): δ -102.3 (d, J_1 = 39.9 Hz, 1F); ^{13}C NMR (100 MHz, $CDCl_3$): δ 165.8, 161.7, 159.3 (d, J = 3.5 Hz), 153.7 (d, J = 253.5 Hz), 144.9, 135.3, 133.5 (d, J = 25.4 Hz), 130.9, 130.6 (d, J = 8.2 Hz), 129.4, 128.7, 125.4 (d, J = 4.1 Hz), 124.2, 114.5, 113.9, 110.1 (d, J = 9.5 Hz), 55.6, 55.3, 21.8; HRMS (ESI) calcd. For $C_{24}H_{22}FNO_5S$ $[M+H]^+$: 456.1281, found: 456.1277.

(*Z*)-4-Fluoro-2-(1-fluoro-2-(4-methoxyphenyl)vinyl)-*N*-tosylbenzamide

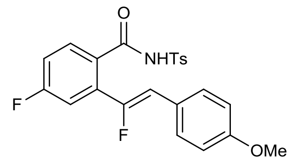

7da; 52%

(7da): Procedure C, light yellow viscous oil. ^1H NMR (400 MHz, $CDCl_3$): δ 7.84 (d, J = 7.3 Hz, 2H), 7.41 (q, J = 5.9 Hz, 1H), 7.30 (d, J = 8.0 Hz, 2H), 7.05-7.16 (m, 3H), 6.89 (t, J = 10.1 Hz, 1H), 6.83 (d, J = 8.7 Hz, 2H), 5.88 (d, J = 38.5 Hz, 1H), 3.82 (s, 3H), 2.37 (s, 3H); ^{19}F NMR (376 MHz, $CDCl_3$): δ -104.6 (s, 1F), -107.9 (s, 1F); ^{13}C NMR (100 MHz, $CDCl_3$): δ 164.7, 162.2, 159.4 (d, J = 5.8 Hz), 152.8 (d, J = 255.6 Hz), 144.4, 135.7, 134.2 (d, J = 8.9 Hz), 133.9 (d, J = 9.4 Hz), 131.1 (d, J = 9.4 Hz), 130.6 (d, J = 8.9 Hz), 129.3, 128.3, 125.2 (d, J = 4.4 Hz), 116.1, 115.9, 115.0 (d, J = 5.2 Hz), 114.8 (d, J = 6.5 Hz), 113.9, 111.1 (d, J = 10.9 Hz), 55.3, 21.7; HRMS (ESI) calcd. For $C_{23}H_{19}F_2NO_4S$ $[M+H]^+$: 444.1081, found: 444.1087.

(*Z*)-2-(1-Fluoro-2-(4-methoxyphenyl)vinyl)-*N*-tosyl-4-(trifluoromethyl)benzamide

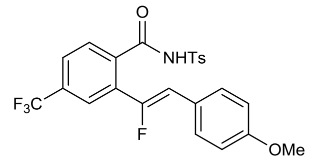

7ea; 52%

(7ea): Procedure C, light yellow viscous oil. ^1H NMR (400 MHz, $CDCl_3$): δ 7.87 (d, J = 8.2 Hz, 2H), 7.68 (s, 1H), 7.51 (s, 2H), 7.32 (d, J = 8.2 Hz, 2H), 7.21 (d, J = 8.5 Hz, 2H), 6.86 (d, J = 8.2 Hz, 2H), 5.99 (d, J = 39.2

Hz, 1H), 3.84 (s, 3H), 2.41 (s, 3H); ^{19}F NMR (376 MHz, $CDCl_3$): δ -63.2 (s, 3F), -106.2 (d, J = 39.2 Hz, 1F); ^{13}C NMR (100 MHz, $CDCl_3$): δ 166.0, 159.6 (d, J = 2.7 Hz), 152.2 (d, J = 257.3 Hz), 145.0, 135.1 (d, J = 6.5 Hz), 133.0, 132.3, 132.0, 130.7 (d, J = 8.2 Hz), 129.5, 129.0, 128.6, 125.7 (d, J = 3.0 Hz), 124.6 (m), 121.8, 113.9, 111.7 (d, J = 9.6 Hz), 55.3, 21.7; HRMS (ESI) calcd. For $C_{24}H_{19}F_4NO_4S$ $[M+H]^+$: 494.1049, found: 494.1053.

Methyl-(*Z*)-3-(1-fluoro-2-(4-methoxyphenyl)vinyl)-4-(tosylcarbamoyl)benzoate

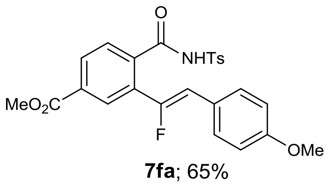

7fa; 65%

(7fa): Procedure C, light yellow solid, m.p. 94-96. ^{1}H NMR (400 MHz, $CDCl_3$): δ 8.08 (s, 1H), 7.92 (d, J = 8.7 Hz, 3H), 7.49 (d, J = 7.9 Hz, 1H), 7.32 (d, J = 8.6 Hz, 2H), 7.24 (d, J = 7.4 Hz, 2H), 6.86 (d, J = 9.2 Hz, 2H), 6.01 (d, J = 37.3 Hz, 1H), 3.91 (s, 3H), 3.84 (s, 3H), 2.43 (s, 3H); ^{19}F NMR (376 MHz, $CDCl_3$): δ 105.6 (d, J = 37.3 Hz, 1F); ^{13}C NMR (100 MHz, $CDCl_3$): δ 165.6, 159.4 (d, J = 2.8 Hz), 153.1 (d, J = 261.9 Hz), 144.9, 135.5, 135.2, 132.3, 131.7, 131.5, 130.7 (d, J = 9.8 Hz), 129.8, 129.5, 128.8 (d, J = 5.80 Hz), 128.6 (d, J = 4.0 Hz), 125.3 (d, J = 3.5 Hz), 113.9, 111.1 (d, J = 10.3 Hz), 55.3, 52.7, 21.7; HRMS (ESI) calcd. For $C_{25}H_{22}FNO_6S$ $[M+H]^+$: 484.1230, found: 484.1239.

***tert*-Butyl-(*Z*)-(4-(1-fluoro-2-(4-methoxyphenyl)vinyl)-2-methyl-5-(tosylcarbamoyl)phenyl)carbamate (7ga):**

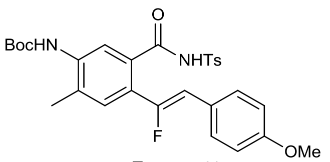

7ga; 50%

Procedure C, light yellow viscous oil. ^{1}H NMR (400 MHz, $CDCl_3$): δ 7.99 (s, 1H), 7.94 (d, J = 8.2 Hz, 2H), 7.32 (d, J = 7.3 Hz, 2H), 7.25 (d, J = 7.5 Hz, 2H), 6.84 (d, J = 10.0 Hz, 2H), 6.45 (s, 1H), 5.84 (d, J = 38.1 Hz, 1H), 3.83 (s, 3H), 2.43 (s, 3H), 2.24 (s, 3H), 1.51 (s, 6H); ^{19}F NMR (376 MHz, $CDCl_3$): δ -103.2 (dq, J_1 = 38.1 Hz, J_2 = 6.9 Hz 1F); ^{13}C NMR (100 MHz, $CDCl_3$): δ 165.8, 158.9 (d, J = 2.5 Hz), 153.7 (d, J = 246.4 Hz), 144.7, 137.3, 135.4, 130.5, 130.3 (d, J = 8.0 Hz), 130.2 (d, J = 5.1 Hz), 129.6, 129.4, 128.7, 126.4 (d, J = 4.5 Hz), 126.2, 125.8 (d, J = 2.8 Hz), 119.8, 113.8, 109.5 (d, J = 11.0 Hz), 81.6, 55.3, 28.2, 21.7, 17.7; HRMS (ESI) calcd. For $C_{29}H_{31}FN_2O_6S$ $[M+H]^+$: 555.1965, found: 555.1966.

(*Z*)-5-(1-Fluoro-2-(4-methoxyphenyl)vinyl)-*N*-tosylthiophene-2-carboxamide

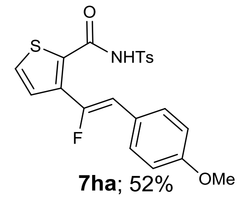

7ha; 52%

(7ha) : Procedure C, white solid, m.p. 79-80. ^{1}H NMR (400 MHz, DMSO-d_6): δ 7.70 (d, J = 7.5 Hz, 2H), 7.36-7.51 (m, 3H), 7.19 (d, J = 7.2 Hz, 2H), 7.13 (d, J = 5.2 Hz, 1H), 6.94 (d, J = 8.9 Hz, 2H), 6.64 (d, J = 41.6 Hz, 1H), 3.78 (s, 3H), 2.32 (s, 3H); ^{19}F NMR (376 MHz, DMSO-d_6): δ 101.2 (s 1F); ^{13}C NMR (100 MHz, DMSO-d_6): δ 165.5, 158.7 (d, J = 2.7 Hz), 152.3 (d, J = 256.7 Hz), 143.3, 140.2, 133.2, 132.9, 130.5 (d, J = 8.9 Hz), 128.6, 127.2, 126.8 (d, J = 2.8 Hz), 114.5, 109.4 (d, J = 9.1 Hz), 55.6, 21.3; HRMS (ESI) calcd. For $C_{21}H_{18}FNO_4S_2$ $[M+H]^+$: 432.0740, found: 432.0753.

(*Z*)-2-(1-Fluoro-2-(4-methoxyphenyl)vinyl)-*N*,*N*-diisopropylbenzamide (7ia):

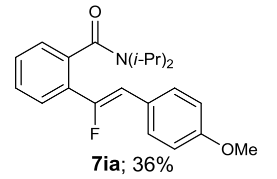

7ia; 36%

Procedure D, white solid, m.p. 102-104. ^{1}H NMR (400 MHz, $CDCl_3$): δ 7.49- 7.56 (m, 1H), 7.43- 7.49 (m, 2H), 7.27- 7.35

(m, 2H), 7.2- 7.8 (m, 1H), 6.82 (d, *J* = 8.7 Hz, 2H), 6.20 (d, *J* = 40.5 Hz, 1H), 3.75 (s, 3H), 3.50- 3.63 (m, 1H), 3.31- 3.42 (m, 1H), 1.49 (d, *J* = 7.1 Hz, 3H), 1.41 (d, *J* = 7.1 Hz, 3H), 0.97 (q, *J* = 6.8 Hz, 6H); ^{19}F NMR (376 MHz, $CDCl_3$): δ -106.8 (d, *J* = 40.5 Hz, 1F); ^{13}C NMR (100 MHz, $CDCl_3$): δ 168.9, 157.8 (d, *J* = 3.0 Hz), 153.4 (d, *J* = 257.0 Hz), 135.4 (d, *J* = 3.8 Hz), 129.4 (d, *J* = 8.2 Hz), 128.6 (d, *J* = 28.1 Hz), 128.0, 127.2, 125.7 (d, *J* = 6.4 Hz), 125.4, 125.3 (d, *J* = 3.1 Hz), 112.9, 108.5 (d, *J* = 11.3 Hz), 54.2, 49.9, 44.8, 19.6 (d, *J* = 11.4 Hz), 19.0; HRMS (ESI) calcd. For $C_{22}H_{26}FNO_2$ $[M+H]^+$: 356.2026, found: 356.2035.

(*Z*)-2-(1-Fluoro-2-(4-methoxyphenyl)vinyl)-*N*,*N*-diisopropyl-4-methylbenzamide

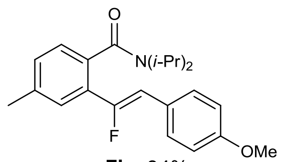

7ja; 34%

(7ja): Procedure D, white solid, m.p. 123-124. ^{1}H NMR (400 MHz, $CDCl_3$): δ 7.53 (d, *J* = 9.9 Hz, 2H), 7.39 (s, 1H), 7.14-7.22 (m, 1H), 7.09-7.14 (m, 1H), 6.88 (d, *J* = 8.5 Hz, 2H), 6.24 (d, *J* = 39.9 Hz, 1H), 3.82 (d, *J* = 0.8 Hz, 3H), 3.60-3.69 (m, 1H), 3.38-3.47 (m, 1H), 2.39 (s, 3H), 1.55 (d, *J* = 6.4 Hz, 3H), 1.48 (d, *J* = 6.4 Hz, 3H), 1.03 (q, *J* = 6.4 Hz, 6H); ^{19}F NMR (376 MHz, $CDCl_3$): δ -106.7 (d, *J* = 39.9 Hz, 1F); ^{13}C NMR (100 MHz, $CDCl_3$): δ 170.2, 158.8 (d, *J* = 4.6 Hz), 154.5 (d, *J* = 257.5 Hz), 138.0, 133.8 (d, *J* = 5.2 Hz), 130.4 (d, *J* = 8.2 Hz), 129.8, 129.5 (d, *J* = 26.1 Hz), 129.4, 127.1 (d, *J* = 6.9 Hz), 125.6, 113.9, 109.3 (d, *J* = 9.7 Hz), 55.2, 50.9, 45.7, 21.2, 20.7 (d, *J* = 11.8 Hz), 20.0; HRMS (ESI) calcd. For $C_{23}H_{28}FNO_2$ $[M+H]^+$: 370.2182, found: 370.2192.

(*Z*)-2-(1-Fluoro-2-(4-methoxyphenyl)vinyl)-*N*,*N*-diisopropyl-4-methoxybenzamid e (7ka): Procedure D, colorless viscous oil. ^{1}H NMR (400 MHz, $CDCl_3$): δ 7.53 (d, *J* = 10.7 Hz, 2H), 7.27 (d, *J* = 9.3 Hz, 1H), 7.15 (d, *J* = 7.8 Hz, 1H), 7.09 (d, *J* = 3.0 Hz, 1H), 6.85-6.93 (m, 3H), 6.27 (d, *J* = 40.2 Hz, 1H), 3.85 (s, 3H), 3.82 (s, 3H), 3.59-3.70 (m, 1H), 3.37-3.46 (m, 1H), 1.55 (d, *J* = 7.7 Hz, 3H), 1.46 (d, *J* = 7.7 Hz, 3H), 1.03 (q, *J* = 7.7 Hz, 6H); ^{19}F NMR (376 MHz, $CDCl_3$): δ -107.0 (d, *J* = 40.2 Hz, 1F); ^{13}C NMR (100 MHz, $CDCl_3$): δ 170.0, 159.2, 158.9 (d, *J* = 2.9 Hz), 154.2 (d, *J* = 255.5 Hz), 131.3, 130.9, 130.5 (d, *J* = 7.6 Hz), 129.3 (d, *J* = 8.6 Hz), 127.8, 127.5, 126.2 (d, *J* = 3.1 Hz), 114.9, 113.9, 113.7, 111.6 (d, *J* = 10.8 Hz), 109.7 (d, *J* = 11.1 Hz), 55.4, 55.2, 50.9, 45.8, 20.8, 20.7 (d, *J* = 8.4 Hz), 20.0 (d, *J* = 3.7 Hz); HRMS (ESI) calcd. For $C_{23}H_{28}FNO_3$ $[M+H]^+$: 386.2131, found: 386.2140.

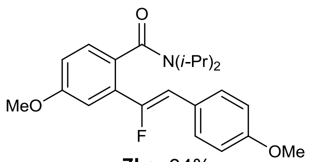

7ka; 34%

(*Z*)-5-(1-Fluoro-2-(4-methoxyphenyl)vinyl)-*N*,*N*-diisopropylthiophene-2-carboxa mide (7la): Procedure D, colorless viscous oil. ^{1}H NMR (400 MHz, $CDCl_3$): δ 7.50 (d, *J* = 8.8 Hz, 2H), 7.25-7.27 (m, 1H), 7.14 (d, *J* = 5.2 Hz, 1H), 6.88 (d, *J* = 8.8 Hz, 2H), 6.11 (d, *J* = 40.7 Hz, 1H), 3.82 (s, 4H), 3.49 (brs, 1H), 1.54 (s, 6H), 1.11 (s, 6H); ^{19}F NMR (376 MHz, $CDCl_3$): δ -109.2 (d, *J* = 40.7 Hz, 1F); ^{13}C NMR (100 MHz, $CDCl_3$): δ 163.8, 158.9 (d, *J* = 5.1 Hz), 152.1 (d, *J* = 255.4 Hz), 134.1 (d, *J* = 5.6 Hz), 131.0, 130.7,

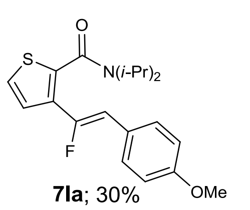

7la; 30%

130.2 (d, J = 8.3 Hz), 126.1 (d, J = 4.3 Hz), 125.0 (d, J = 6.0 Hz), 124.6, 114.0, 107.8 (d, J = 9.3 Hz), 55.2, 53.4, 51.7, 46.3, 20.4 (d, J = 18.1 Hz); HRMS (ESI) calcd. For $C_{20}H_{24}FNO_2S$ [M+H]$^+$: 362.1590, found: 362.1595.

2-((4-Methoxyphenyl)ethynyl)-1*H*-indole (8a):

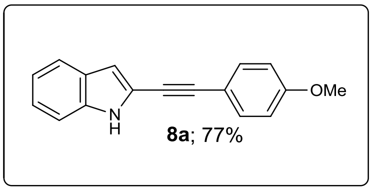

White solid, m.p. 168-169. ^{1}H NMR (400 MHz, $CDCl_3$): δ 8.21 (s, 1H), 7.59 (d, J = 7.6 Hz, 1H), 7.46-7.50 (m, 2H), 7.33 (dd, J_1 = 8.6 Hz, J_2 = 0.8 Hz, 1H), 7.23 (td, J_1 = 6.8 Hz, J_2 = 1.2 Hz, 1H), 7.12 (td, J_1 = 7.5 Hz, J_2 = 1.1 Hz, 1H), 6.89 (m, 2H), 6.80 (dd, J_1 = 2.1 Hz, J_2 = 0.9 Hz, 1H), 3.84 (s, 3H); ^{13}C NMR (100 MHz, $CDCl_3$): δ 159.9, 136.1, 133.0, 127.9, 123.3, 120.7, 120.4, 119.2, 114.6, 114.1, 110.6, 108.3, 92.5, 80.4, 55.3; HRMS (ESI) calcd. For $C_{17}H_{13}NO$ [M+H]$^+$: 248.1075, found: 248.1081.

2-((4-Methoxyphenyl)ethynyl)-4-methyl-1*H*-indolen (8b): White solid, m.p. 112-114. ^{1}H NMR (400 MHz, $CDCl_3$): δ 8.19 (s, 1H), 7.47 (m, 2H), 7.09-7.17 (m, 2H), 6.86-6.92 (m, 3H), 6.82 (dd, J_1 = 2.1 Hz, J_2 = 0.7 Hz, 1H), 3.82 (s, 3H), 2.54 (s, 3H); ^{13}C NMR (100 MHz, $CDCl_3$): δ 159.8, 135.8, 132.9, 130.4, 127.9, 123.5, 120.5, 118.5, 114.7, 114.2, 108.3, 107.0, 92.4, 80.6, 55.3, 18.7; HRMS (ESI) calcd. For $C_{18}H_{15}NO$ [M+H]$^+$: 262.1232, found: 262.1233.

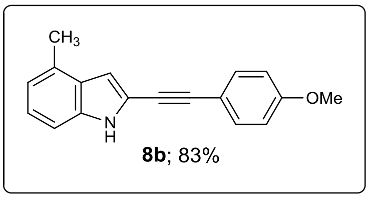

5-Fluoro-2-((4-methoxyphenyl)ethynyl)-1*H*-indole (8c):White solid, m.p. 177-179. ^{1}H NMR (400 MHz, $CDCl_3$): δ 8.20 (s, 1H), 7.44-7.53 (m, 2H), 7.21-7.25 (m, 2H), 6.97 (td, J_1 = 8.9 Hz, J_2 = 2.3 Hz, 1H), 6.86-6.92 (m, 2H), 6.74 (d, J = 1.5 Hz, 1H), 3.84 (s, 3H); ^{19}F NMR (376 MHz, $CDCl_3$): δ -123.8 (m, 1F); ^{13}C NMR (100 MHz, $CDCl_3$): δ 160.0, 158.2 (d, J = 236.7 Hz),133.0, 132.5, 128.2 (d, J = 2.1 Hz), 120.9, 114.4, 114.2, 111.7 (d, J = 26.5 Hz), 111.3 (d, J = 10.0 Hz), 108.2 (d, J = 5.0 Hz), 105.3 (d, J = 22.7 Hz), 92.9, 80.0, 55.3; HRMS (ESI) calcd. For $C_{17}H_{12}FNO$ [M+H]$^+$: 266.0981, found: 266.0979.

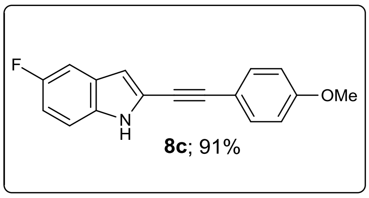

2-(5-Methoxy-2-((4-methoxyphenyl)ethynyl)phenyl)pyridine (8d): Greennish oil. ^{1}H NMR (400 MHz, $CDCl_3$): δ 8.74 (dq, J_1 = 4.8 Hz, J_2 = 1.0 Hz, 1H), 8.06 (d, J = 7.9 Hz, 1H), 7.56 td, J_1 = 7.7 Hz, J_2 = 1.8 Hz, 1H), 7.56 d, J = 8.6 Hz, 1H), 7.34 (d, J = 2.7 Hz, 1H), 7.23-7.32 (m, 3H), 6.93 (dd, J_1 = 8.6 Hz, J_2 = 2.7 Hz, 1H), 6.78-6.85 (m, 2H), 3.87 (s, 3H), 3.79 (s, 3H); ^{13}C NMR (100 MHz, $CDCl_3$): δ 159.6, 159.4, 157.5, 149.4, 143.4, 135.5, 134.3, 132.5, 124.5,122.3, 115.8, 115.1, 114.2, 113.9, 113.8, 91.1, 87.8, 55.4 ,55.3; HRMS (ESI) calcd. For $C_{21}H_{17}NO_2$ [M+H]$^+$: 316.1338, found: 316.1340.

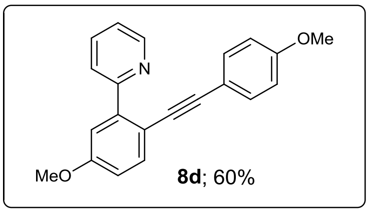

Supplementary References

1. Riss, P. J. & Aigbirhio, F. I. A simple, rapid procedure for nucleophilic radiosynthesis of aliphatic [^{18}F]trifluoromethyl groups. *Chem. Commun.* **47,** 11873-11875 (2011).
2. Zheng, J., Cai, J. & Xiao, J. C. Synthesis and decarboxylative Wittig reaction of difluoromethylene phosphobetaine. *Chem. Commun.* **49,** 7513-7515 (2013).
3. Lu, M. Z., Lu, P., Xu, Y. H. & Loh, T. P. Mild Rh(III)-catalyzed direct C−H bond arylation of (hetero)arenes witharylsilanes in aqueous media. *Org. Lett.* **16,** 2614-2617 (2014).
4. Billiingsley, K. & Buchwald, S. L. Highly efficient monophosphine-based catalyst for the palladium-catalyzed Suzuki-Miyaura reaction of heteroaryl halides and heteroaryl horonic acids andesters. *J. Am. Chem. Soc.* **129,** 3358-3366 (2007).
5. Mizuno, H., Takaya, J. & Iwasawa, N. Rhodium(I)-catalyzed direct carboxylation of arenes with CO_2 via chelation-assisted C-H bond activation. *J. Am. Chem. Soc.* **133,** 1251-1253 (2011).
6. Wei, Y. & Yoshikai, N. Modular pyridine synthesis from oximes and enals throughsynergistic copper/iminium catalysis. *J. Am. Chem. Soc.* **135,** 3756-3759 (2013).
7. Zheng, X. J., Song, B. R. & Xu, B. Eur. Palladium-catalyzed regioselective C–H bond ortho-acetoxylation of arylpyrimidines. *J. Org. Chem.* **23,** 4376-4380 (2010).
8. Hwang, G. T., Son, H. S. & Kim, B. H. Synthesis and photophysical studies of bis-enediynes as tunable fluorophores. *J. Am. Chem. Soc.* **125,** 11241-11248 (2003).
9. Furuya, T. & Ritter, T. Fluorination of boronic acids mediated by silver(I) triflate *Org. Lett.* **11,** 2860-2863 (2009).
